# Divergent Kinetic/Thermodynamic Selectivity in Palladium(II)-Mediated C–H Activation to Form 5- and 6‑Membered Palladacycles

**DOI:** 10.1021/jacs.5c02735

**Published:** 2025-07-15

**Authors:** Philip S. Zhou, Kyana M. Sanders, Ilia A. Guzei, Djamaladdin G. Musaev, Shannon S. Stahl

**Affiliations:** † Department of Chemistry, 5228 University of Wisconsin—Madison, Madison, Wisconsin 53706, United States; ‡ Cherry L. Emerson Center for Scientific Computation, 1371 Emory University, Atlanta, Georgia 30322, United States

## Abstract

Palladium-catalyzed C–H functionalization proceeds via metallacycle formation and often favors 6-membered palladacycles, contrasting the typical preference for 5-membered chelates in transition-metal complexes. The present study probes the origin of this behavior by comparing the reactivity of benzoate and phenylacetate substrates in stoichiometric and catalytic reactions with MPAA-ligated Pd­(OAc)_2_ (MPAA = mono-*N*-protected amino acid). Stoichiometric competition studies show that 6-membered palladacycle formation is kinetically favored, while the 5-membered palladacycle is thermodynamically favored. Density functional theory (DFT) calculations reveal that the transition state for 5-membered palladacycle formation is inhibited by a higher distortion energy. Replacement of the α-MPAA ligand with a more flexible β-MPAA derivative lowers the transition state energy of benzoate C–H activation and improves the catalytic performance.

## Graphical abstract

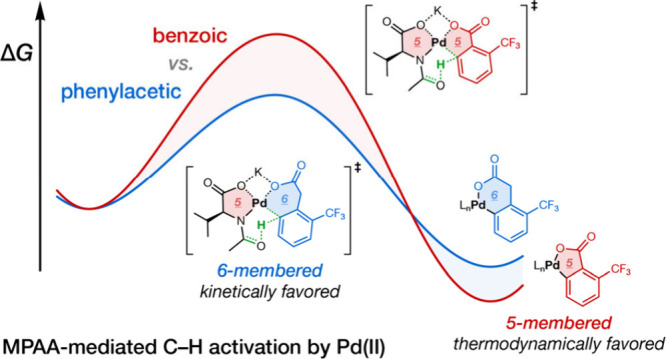

Chelate-directed palladium­(II)-catalyzed C–H functionalization reactions are versatile synthetic methods in organic chemistry.^1–6^ These reactions typically form a palladacycle intermediate that can undergo various transformations, resulting in a net functionalization of the C–H bond (Figure 1A). Yu and co-workers have highlighted the ability of mono-*N*-protected amino acid (MPAA) ligands to promote C–H functionalization in diverse catalytic transformations,^1,3,5,7^ among which benzoic and phenylacetic acids are prototypical substrates. Comparison of their reactivity in Pd-catalyzed *ortho*-butylation (Figure 1B)^8^ reveals that these reactions favor formation of 6- rather than 5-membered palladacycles. This selectivity, replicated in complementary arylation^9,10^ and olefination reactions,^11–13^ conflicts with the long-recognized preference for transition-metal complexes to form 5-membered chelates (Figure 1C)^14^ and raises questions about the origin of selectivity. Recent studies of directed C­(sp^3^)–H functionalization show that site-selectivity can be modulated by ancillary ligands^16,17^ and/or transient directing groups;^18–21^ however, the basis for selectivity in now-classical Pd­(OAc)_2_/MPAA-catalyzed C­(sp^2^)–H functionalization reactions has not been elucidated. Here, we use a series of benzoate and phenylacetate derivatives to probe stoichiometric and catalytic C­(sp^2^)–H functionalization reactions (Figure 1D). The collective data reveal a thermodynamic and kinetic preference for formation of 5- and 6-membered palladacycles, respectively.^22^ Density functional theory (DFT) calculations show the MPAA ligand imposes conformational constraints on the C–H activation transition states, disfavoring the 5-membered palladacycle. These insights provide the basis for modification of the MPAA ligand to enable a more effective reactivity with benzoic acid substrates.

**Figure 1:**
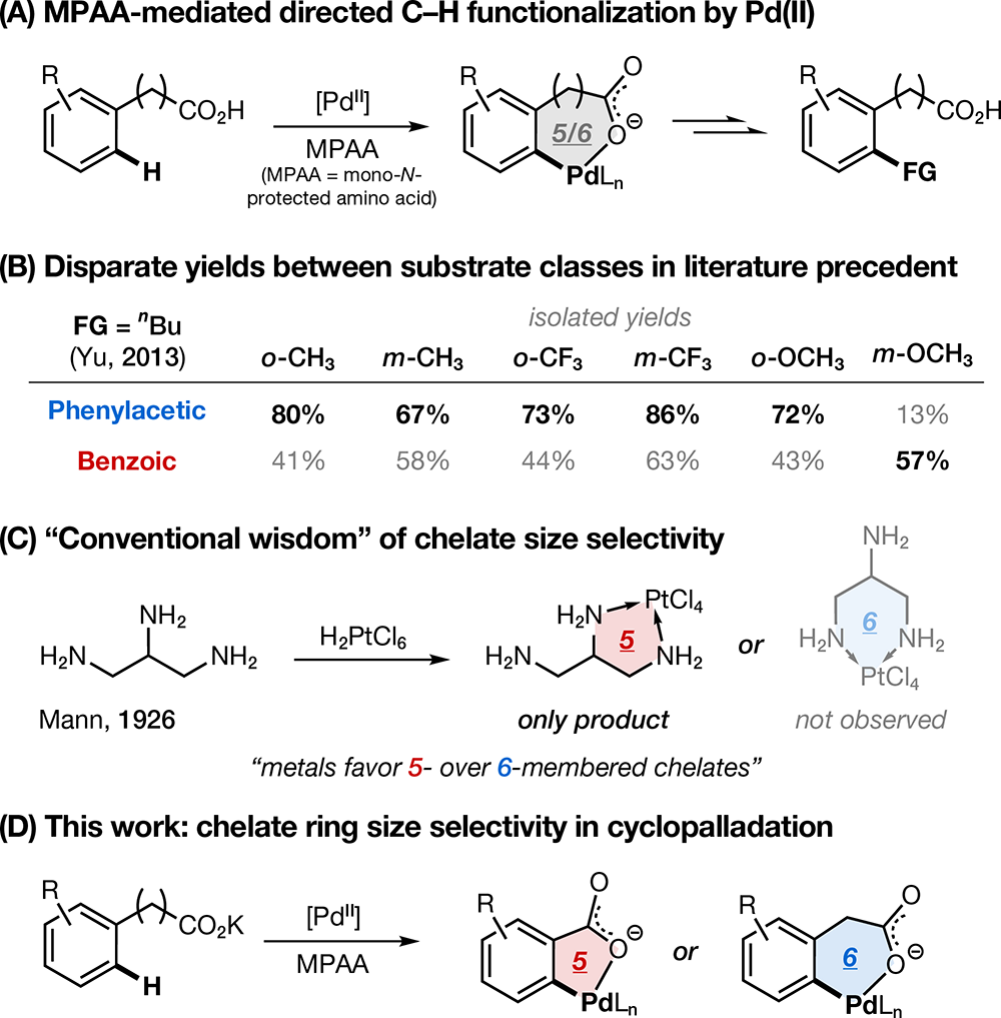
(A) Pd^II^-catalyzed C–H functionalization of benzoic and phenylacetic acid substrates mediated by MPAA ligands. (B) Data for Pd-catalyzed *n*-butylation. (C) Historical data favor 5- over 6-membered metallacycles. (D) Focus of the present study.

In a recent study of Pd­(OAc)_2_/MPAA-catalyzed C–H arylation,^10^ we noted improved reactivity of phenylacetates relative to benzoates, similar to the observations highlighted in Figure 1B.^8^ The present study was initiated with a systematic comparison of representative benzoic and phenylacetic acid substrates to gain further insight into their reactivity difference. As shown in Figure 2, phenylacetic acids undergo nearly complete substrate conversion, albeit with some forming diarylation products (Supporting Information, section 6d). In contrast, benzoic acids exhibit poor-to-moderate reactivity. Initial efforts to examine the origin of this reactivity difference focused on the C–H activation step, probing the stoichiometric cyclopalladation of *o*-CF_3_ benzoate (**1a**) and phenylacetate (**1b**) in the presence of 10% Pd­(OAc)_2_/*N*-acetyl-l-valine (Ac-Val-OH). C–H activation to afford the palladacycle proceeds smoothly in ^*t*^AmylOH at 40 °C, as evident by ^19^F NMR spectroscopy (Figure 3A). The spectra of **1a-Pd** and **1b-Pd** reveal an ensemble of peaks reflecting a dynamic mixture of palladacycle species with different ancillary ligation and/or oligomerization.^23,24^ Addition of pyridine causes this mixture to converge into a single pyridine-coordinated palladacycle (**1a-Pd-py** or **1b-Pd-py**). Analogs bearing 4-methylpyridine ligands were characterized by X-ray crystallography, and the structures reveal a planar geometry in the 5-membered palladacycle and a nonplanar “boat” configuration of the 6-membered palladacycle (Figure 3B).

**Figure 2:**
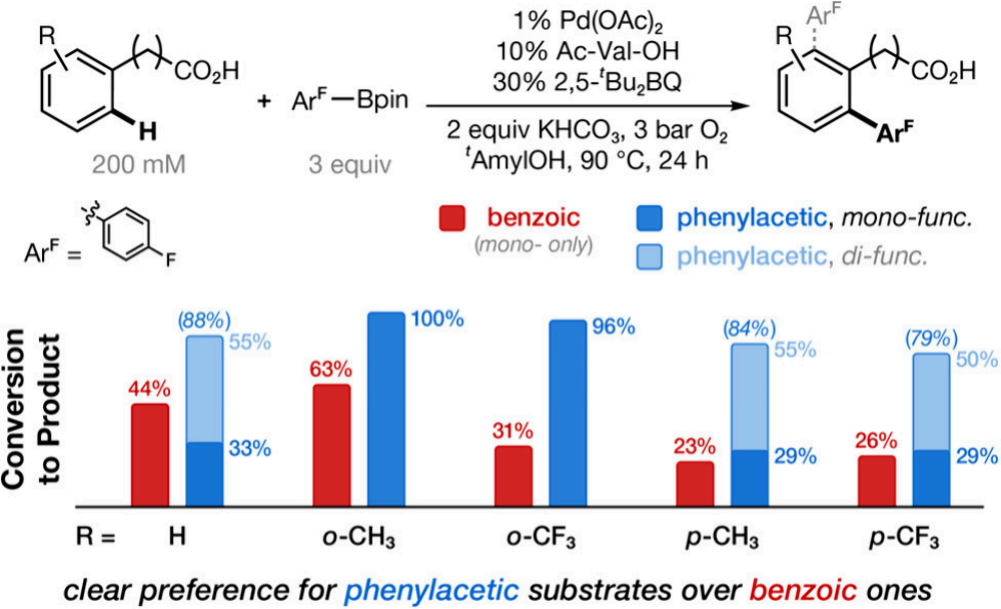
Yield comparison of benzoic and phenylacetic acid substrates in the *ortho*-arylation reaction. Conversion was established by ^19^F­{^1^H} NMR of reaction mixtures.

**Figure 3:**
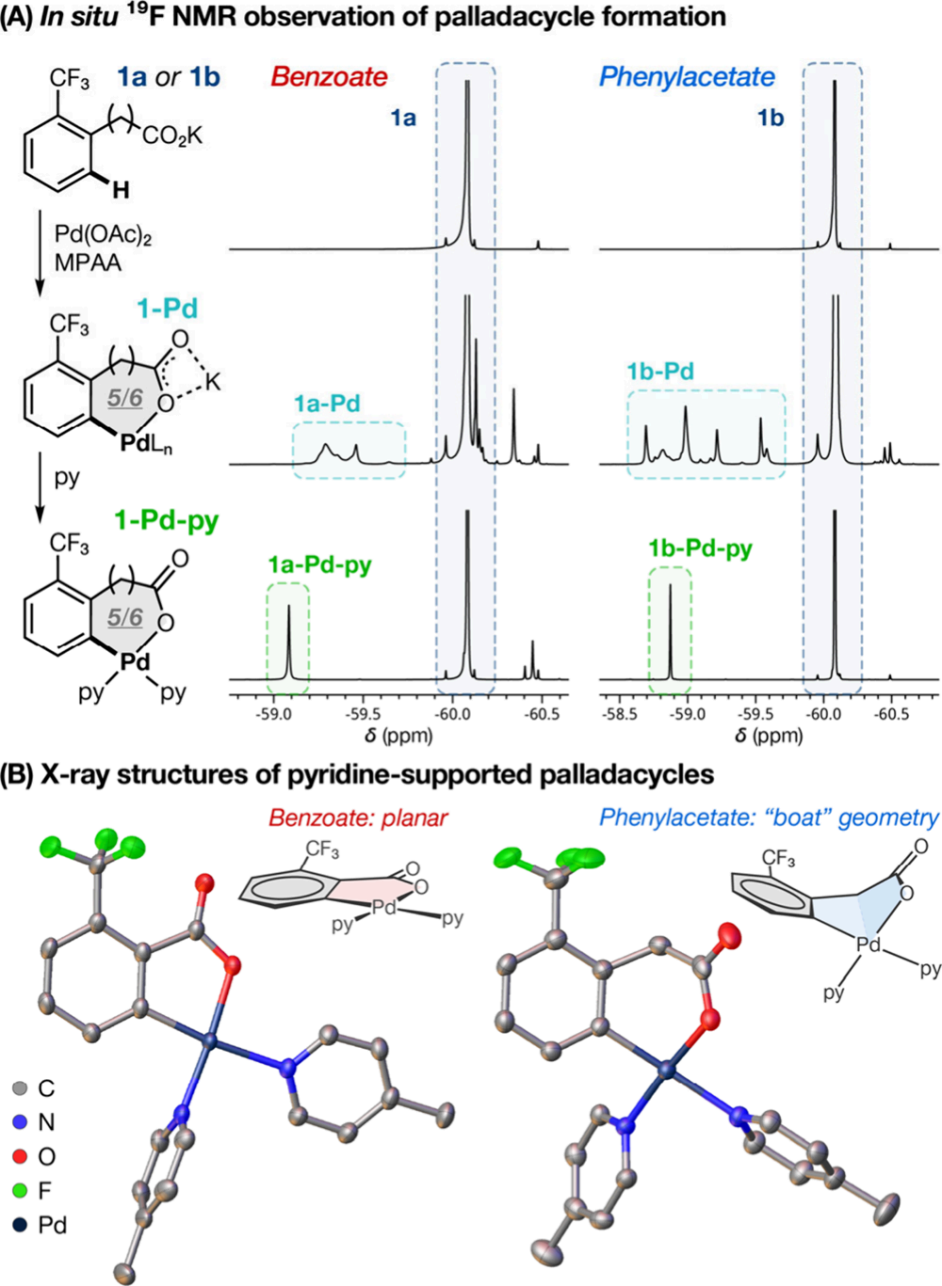
(A) ^19^F NMR spectra of species observed in the C–H activation of *o*-CF_3_ benzoate (**1a**) and phenylacetate (**1b**). Reaction conditions: 50 mM **1a**/**1b**, 5 mM Pd­(OAc)_2_, 5 mM Ac-Val-OH, ^*t*^AmylOH, 40 °C. (B) X-ray crystal structures of **1a-Pd-** ^**4Me**^ **py** and **1b-Pd-** ^**4Me**^ **py**. Thermal ellipsoids displayed at 50% probability; H atoms are omitted for clarity.

The cyclopalladation kinetics of seven different substrates were then analyzed by ^19^F NMR spectroscopy (Figure 4). The time course data reveal significant differences in C–H activation rates, with notable distinctions between benzoic and phenylacetic acids. For example, a 45-fold rate difference is observed between the cyclopalladation of *o*-CF_3_ benzoate (**1a**) and its phenylacetate counterpart (**1b**). Collectively, phenylacetates react 4- to 86-fold faster than benzoates (see Supporting Information section 3b for derivation of initial rates). In general, faster rates are observed with more electron-rich substrates, while steric effects can alter this trend (**3a** versus **4a**). Phenylacetates are intrinsically more electron-rich than benzoates, raising the question of whether electronic effects could rationalize the rate difference between the substrate classes. To address this question, DFT methods were used to calculate Mulliken charges on the *H* atom undergoing activation and the *O* atoms of the chelating carboxylate (see Supporting Information section 7d). The latter property is expected to correlate with binding of the substrate to Pd^II^. Analysis of rate correlations for each substrate class with these two Mulliken charges confirms that more electron-rich substrates exhibit faster rates. Comparison of benzoates and phenylacetates with similar electronic properties, however, reveals that phenylacetates always exhibit faster rates than benzoates, indicating that the chelate ring size is the major factor leading to differences in the reaction rates between the two substrate classes.

**Figure 4:**
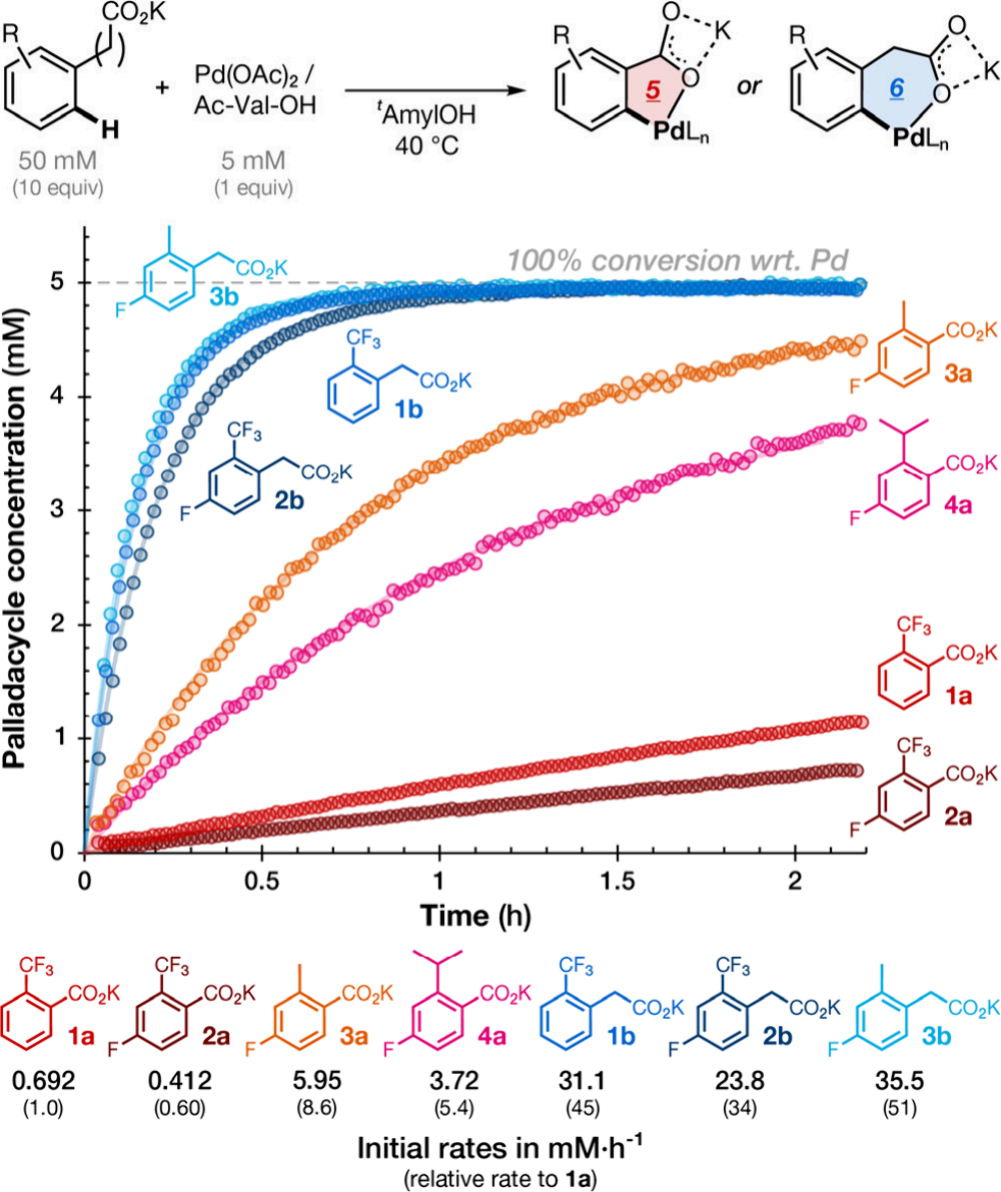
C–H activation time courses of various substrates and a comparison of rate constants determined by fitting the kinetic traces (see Supporting Information section 3b).

The reactivities of benzoate and phenylacetate substrates **3a** and **3b** were then compared in an intermolecular competition experiment (Figure 5). A 1:1 mixture of **3a** and **3b** was combined with 10% Pd­(OAc)_2_/Ac-Val-OH (relative to the total amount of both substrates) and monitored by ^19^F NMR spectroscopy. The reaction conditions closely resemble those used for the time course experiments in Figure 4. Reactivity was observed at 25 °C immediately upon injecting Pd­(OAc)_2_ into the reaction mixture, rapidly generating a mixture of 6-membered palladacycles **3b-Pd**, similar to **1b-Pd** in Figure 3A. Complete formation of **3b-Pd** was observed within 20 min. Upon raising the temperature to 70 °C, the **3b-Pd** species disappeared over approximately 1.5 h, with concomitant formation of the 5-membered palladacycles **3a-Pd**. These observations indicate that C–H activation of **3b** is reversible and that the formation of **3a-Pd** is favored at elevated temperatures. This behavior is attributed to a kinetic preference for formation of the 6-membered palladacycles but a thermodynamic preference for the 5-membered palladacycle.

**Figure 5:**
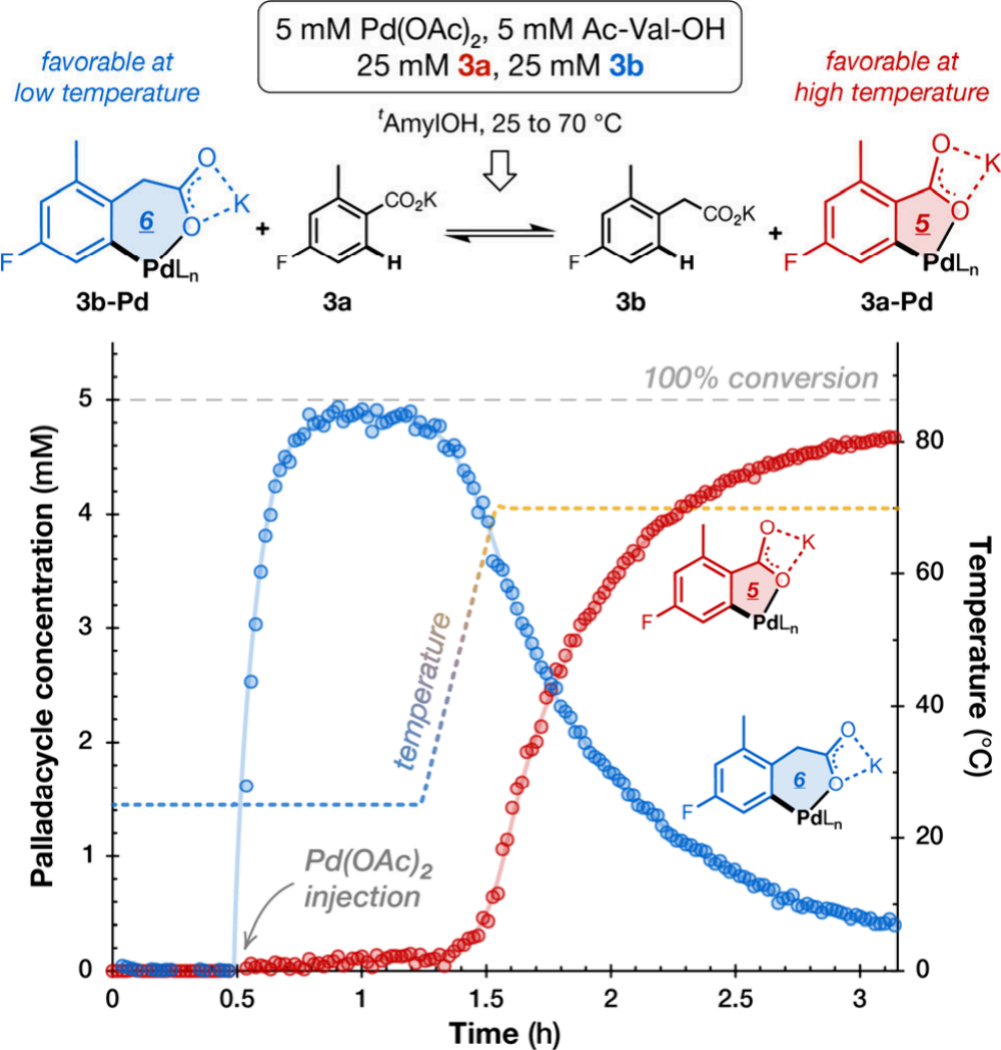
Intramolecular competition experiment showing a switch of selectivity between 5- and 6-membered palladacycle products at different temperatures.

DFT calculations were then used to gain further insights into these experimental results and to identify factors contributing to reactivity and selectivity, using **1a** and **1b** as the model benzoate and phenylacetate substrates, respectively, and with Ac-Val-OH as the MPAA ligand to match the experimental data. Structures were calculated at the B3LYP-D3­(BJ) level of theory and 6-31G­(d,p) basis sets for all atoms except Pd, for which the Lanl2dz basis sets were utilized (Supporting Information section 7a). Leveraging insights from previous studies of Pd/MPAA-catalyzed C–H activation,^25–37^ we initiated the energetic analysis of C–H activation with the [κ^2^-(*N,O*)-MPAA]–Pd–Sub complex, **Sub-5** and **Sub-6** (Figure 6B). A low energy pathway for concerted metalation–deprotonation (CMD) of the substrate aromatic C–H bond employs the carbonyl in the *N*-acyl fragment of the MPAA ligand as a Brønsted base to promote formation of the palladacycle, **PdCycle-5** or **PdCycle-6**. Transition states **TS-5** and **TS-6** associated with these pathways were also calculated. The results show that C–H activation has a lower kinetic barrier for the phenylacetate substrate (**TS-6**) relative to the benzoate (**TS-5**), while the 5-membered palladacycle **PdCycle-5** derived from C–H activation of the benzoate is thermodynamically favored relative to the 6-membered palladacycle **PdCycle-6**. These results are consistent with the experimental observations.

**Figure 6:**
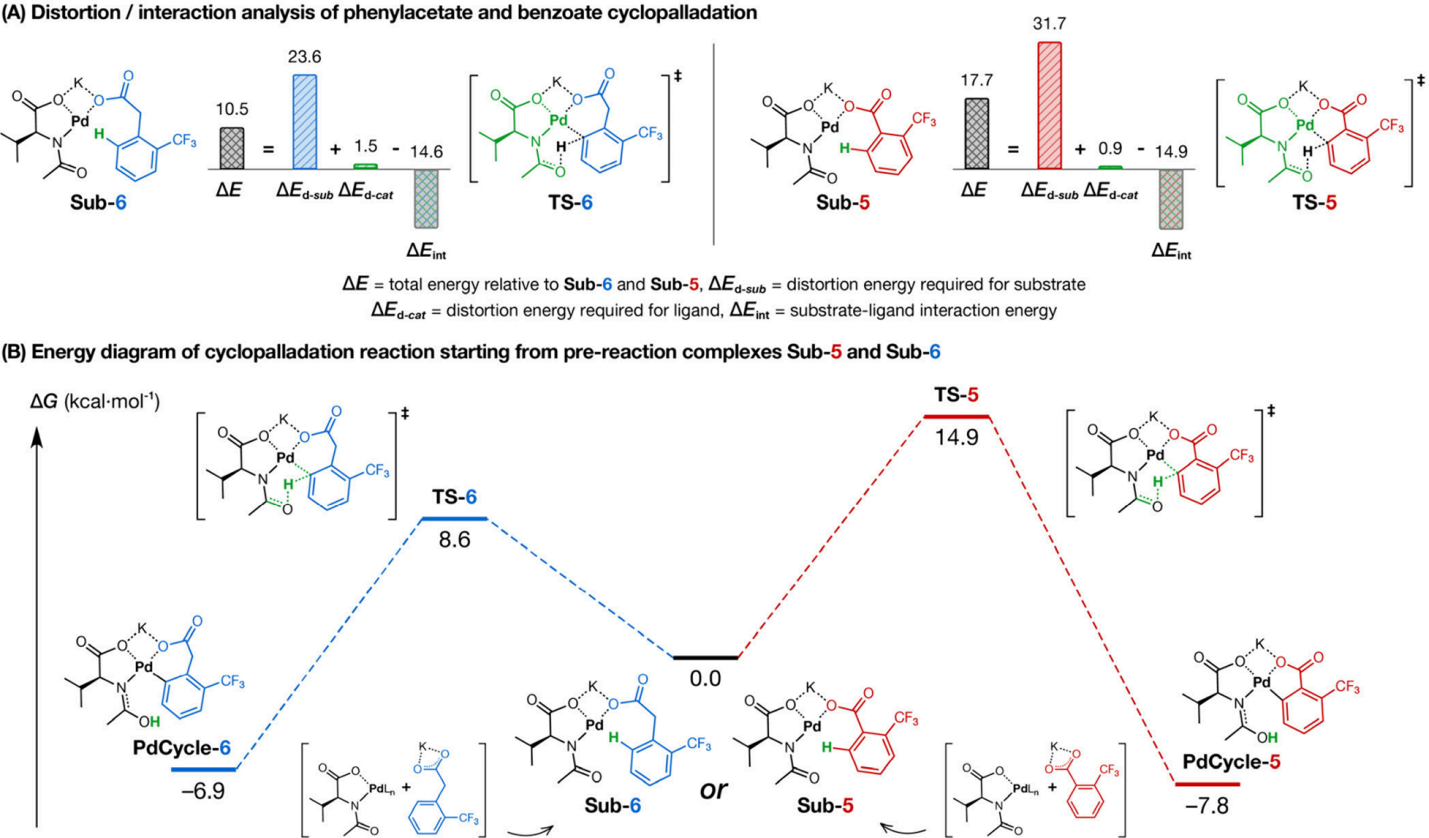
(A) Distortion/interaction analysis of transition states **TS-5** and **TS-6**. (B) Computed free energy diagram of the cyclopalladation of **Sub-5** and **Sub-6** with **L1**. See Supporting Information section 7a for computation details.

Further insights into the origin of kinetic versus thermodynamic selectivity were gained from distortion/interaction analysis of the prereaction complexes and transition states of both substrates. Each structure was computationally partitioned into two subunits: *sub* (blue or red in Figure 6A), corresponding to the substrate fragment; and *cat* (green in Figure 6A), corresponding to the catalytic fragment, including both the MPAA ligand and the Pd center. This analysis shows that the energy required for distortion of the *cat* fragment (Δ*E*_d‑*cat*_) is very small relative to that of the *sub* fragment (Δ*E*_d‑*sub*_). Comparison of the Δ*E*_d‑*sub*_ values shows that the benzoate substrate experiences a significantly higher distortion energy (Δ*E*_d‑*sub*_ = 31.7 and 23.6 kcal·mol^–1^ for benzoate and phenylacetate, respectively, with ΔΔ*E*_d‑*sub*_ = 8.1 kcal·mol^–1^). The *sub*–*cat* interaction energies (Δ*E*_int_) have similarly favorable magnitudes in both transition states and, thus, do not differentiate between benzoate and phenylacetate. These results show that the difference between the C–H activation barriers for benzoate and phenylacetate largely arises from structural distortion of the substrate in the transition state. Specifically, the benzoate transition state requires the arene ring to rotate out of its preferred coplanar arrangement with the carboxylate group to enable the carbon atom of the C–H bond to interact with Pd^II^. The more flexible phenylacetate incurs less energetic cost in forming the transition state structure (lower Δ*E*_d‑*sub*_).

Recent studies that show the chelate ring-size of MPAA-like ligands^15,16,17^ or transient directing groups^18–21^ can alter site-selectivity in aliphatic C­(sp^3^)–H functionalization reactions reveal a cooperative relationship between the size of the two chelates present in the transition state—one from the C–H substrate and one from the ancillary ligand or transient directing group. Although these insights have not been adapted to arene C­(sp^2^)–H activation, they raise the possibility that benzoate reactivity could be improved by using a conformationally more flexible MPAA ligand that would allow better alignment of the reacting C–H bond and the carbonyl of MPAA. To test this hypothesis, we prepared and evaluated a series of homologous β-MPAA ligands in stoichiometric C–H activation and catalytic arylation reactions. Palladacycle formation was analyzed by ^19^F NMR spectroscopy following addition of pyridine (cf. Figure 3), and catalytic arylation reactions were performed and analyzed by ^19^F­{^1^H} NMR spectroscopy (see Supporting Information sections 5 and 6).

β^3^-MPAA ligands showed enhanced efficacy for both C–H activation (Table S2) and catalytic arylation (Figure 7A) of benzoates relative to their α-MPAA analogs. When Ac-α-l-Val-OH (**L1**) and Ac-β^3^-l-Val-OH (**L2**) ligands were compared in the arylation of the original set of benzoate substrates, **L2** led to substantially improved arylation yields compared to those observed with **L1**. DFT computational studies indicate that the benzoate C–H activation barrier is slightly lower with the more flexible β-MPAA ligand **L2** compared to that of the α-MPAA ligand **L1** (Figure 7B). Distortion/interaction analysis (Figure 7C) shows that the transition state with **L2** has reduced substrate distortion energy than that with **L1** (ΔΔ*E*_d‑*sub*_ = 1.9 kcal·mol^–1^; see Supporting Information section 7d). Neither **L1** nor **L2** contributes significantly to transition state distortion energies. Instead, the different conformations of these ligands result in a different orientation of the *N*-acetyl carbonyl group, which imposes a preferred geometry on the approaching arene C–H bond that manifests as a distortion energy in the C–H substrate. This constraint by **L1** is more pronounced due to its own rigidity. The relatively small rate effect is magnified by factors that improve the product selectivity with **L2**. Reactions with *o-* and *p-*CF_3_ benzoates are readily analyzed by ^19^F­{^1^H} NMR spectroscopy, and the data show that use of **L2** contributes to reduced side product formation,^38^ in addition to improved conversion/rate (Figure 7D; see Figures S5 and S6 in the Supporting Information for spectra). Collectively, these results show how modification of the MPAA ligand enables improved reactivity with benzoate substrates. They also have mechanistic implications for the ongoing discovery of new ligands that support other classes of C–H functionalization reactions.^6^

**Figure 7:**
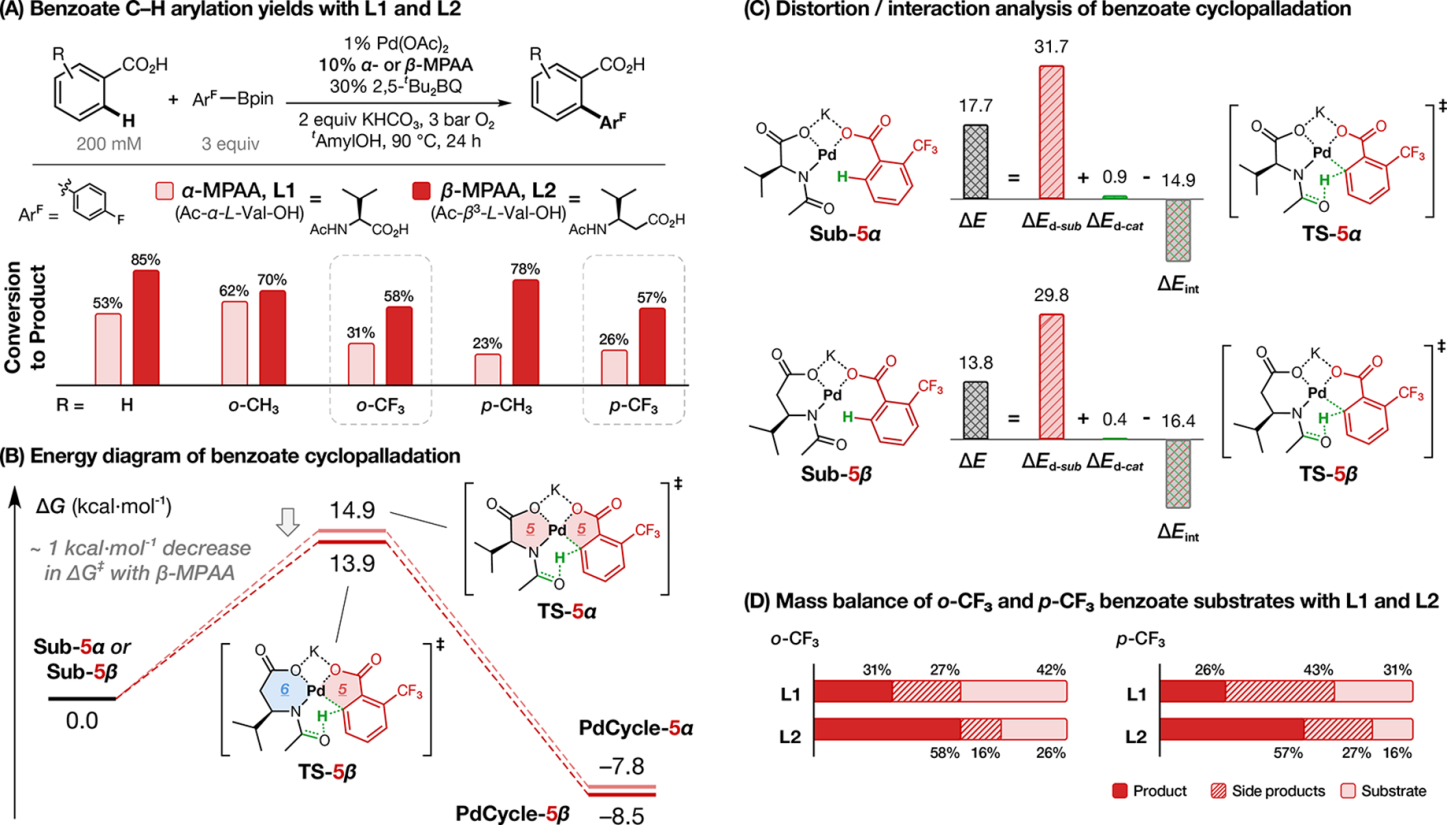
(A) Yield comparison of benzoate substrates in *ortho*-arylation reactions using both α-MPAA (**L1**) and β-MPAA (**L2**) ligands. (B) Computed free energy diagram of the cyclopalladation of **1a** with **L1** and **L2**. (C) Distortion/interaction analysis of transition states **TS-5α** and **TS-5β** (see Supporting Information sections 6 and 7). (D) Mass balance analysis of benzoate substrates with –CF_3_ substituents, using both **L1** and **L2**.

In summary, this study provides rare direct insights into kinetic versus thermodynamic factors that influence the outcome of directed C–H activation/functionalization reactions. C–H activation is reversible and kinetically favors 6-membered palladacycle formation, while the 5-membered palladacycle is thermodynamically favored. These results align with the historical data indicating a prevalence of 5-membered chelates in transition-metal complexes. The rigidity of 5-membered rings, however, leads to higher distortion energies in the transition state for C–H activation by α-MPAA-ligated Pd species. Less unfavorable distortion in 6-membered transition states explains why phenylacetates often undergo more efficient cyclopalladation and functionalization than benzoates. Benzoate reactivity improves significantly when using a more flexible β-MPAA ligand, which supports more effective C–H activation/functionalization. These insights highlight the importance of substrate–ligand pairing in Pd/MPAA-catalyzed C–H functionalization reactions and provide the basis for rational ligand design to exploit the inherent ring-size selectivity in such reactions.

## Supplementary Material



## Notes

Experimental procedures, additional data, computation details, and characterization data. (PDF)
