# Supplementary material for: Divergent Kinetic/Thermodynamic Selectivity in Palladium(II)-Mediated C–H Activation to Form 5- and 6‑Membered Palladacycles
Source: J Am Chem Soc. 2025 Jul 15;147(30):26091–6. doi: 10.1021/jacs.5c02735 (PMC12376188; doi:10.1021/jacs.5c02735)

Supporting Information

Divergent Kinetic/Thermodynamic Selectivity in Palladium(II)-Mediated C–H Activation to Form 5- and 6-Membered Palladacycles

Philip S. Zhou,[a] Kyana M. Sanders,[a] Ilia A. Guzei,[a]
Djamaladdin G. Musaev,*,[b] and Shannon S. Stahl*,[a]

Email: stahl@chem.wisc.edu, dmusaev@emory.edu

[a] Department of Chemistry, University of Wisconsin–Madison, Madison, Wisconsin 53706, United States

[b] Cherry L. Emerson Center for Scientific Computation, Emory University, Atlanta, Georgia 30322, United States

Table of Contents:

1. **General experimental considerations** **S3**
2. **Synthesis and characterization of compounds** **S3**
    - 2a. Synthesis of 4-fluoro-2-isopropylbenzoic acid (4a-H) S3
    - 2b. General procedure for the synthesis of potassium benzoates and phenylacetates S3
    - 2c. General procedure for the synthesis of Ac- protected MPAA ligands S4
    - 2d. Synthesis of 5-membered palladacycles (1a-PdCycle-MePy and 1a-PdCycle-^{t}BuPy) S7
    - 2e. Synthesis of 6-membered palladacycle (1b-PdCycle-MePy) S7
    - 2f. General procedure for the synthesis of benzoic acid C–H arylation products S8
    - 2g. General procedure for the synthesis of phenylacetic acid C–H arylation products S9
3. ***In situ* NMR studies of C–H activation kinetics** **S10**
    - 3a. General experimental procedures S10
    - 3b. Initial rate measurements from NMR data S11
    - 3c. NMR spectra of *in situ* cyclopalladation rate measurements S13
4. ***In situ* NMR observation of C–H activation equilibrium** **S23**
    - 4a. General experimental procedures S23
    - 4b. NMR spectra of C–H activation equilibrium observation S24
5. **C–H activation assay with various MPAA ligands** **S27**
    - 5a. General experimental procedures S27
    - 5b. C–H activation assay results S28
6. **C–H arylation assay** **S29**
    - 6a. General experimental procedures S29
    - 6b. NMR analyses of C–H arylation product mixtures S30
    - 6c. C–H arylation assay results S32
    - 6d. C–H arylation of phenylacetic acids to minimize di-arylation products S32
7. **DFT studies** **S33**
    - 7a. Computation details S33
    - 7b. Comparison of *ortho*- and *meta*- substituted substrates S33
    - 7c. Comparison of L1 and L2 .... S33
    - 7d. Distortion/interaction analyses S34
    - 7e. Linear Free Energy Relationship (LFER) analyses S34
    - 7f. Coordinates of computed structures S34
8. **X-ray crystallography** **S47**
    - 8a. 4-Methylpyridine supported 5-membered palladacycle (1a-PdCycle-MePy) S47
    - 8b. 4-*tert*-butylpyridine supported 5-membered palladacycle (1a-PdCycle-^{t}BuPy) S55
    - 8c. 4-methylpyridine supported 6-membered palladacycle (1b-PdCycle-MePy) S64
9. **References** **S73**
10. **NMR spectra of compounds** **S74**
    - 10a. Benzoate and phenylacetate substrates (^{1}H, ^{13}Cs{^{1}H}, and ^{19}F NMR) S74
    - 10b. MPAA ligands (^{1}H and ^{13}C{^{1}H} NMR) S74
    - 10c. Pyridine-supported palladacycles (^{1}H, ^{13}C{^{1}H} and ^{19}F NMR) S74
    - 10d. Benzoic and phenylacetic acid arylation products (^{1}H, ^{13}C{^{1}H} and ^{19}F NMR) S74

1. General experimental considerations

Commercially available organic compounds and solvents were purchased from Sigma-Aldrich, Oakwood, TCI, and Combi-Blocks and used as received unless otherwise noted. Anhydrous ^{t}AmylOH purchased from Sigma-Aldrich was used for all experiments without further purification. Palladium acetate (99.98% trace metals basis) was purchased from Sigma-Aldrich. Spectroscopic analyses were performed at University of Wisconsin–Madison using the following instruments: ^{1}H, ^{13}C{^{1}H}, ^{19}F, and ^{19}F{^{1}H} NMR spectra for compound characterization and time course studies on Bruker Avance 500 and 600 MHz spectrometers; single crystal X-ray diffraction analyses on Bruker D8 Venture Photon III X-ray diffractometer; and high-resolution mass spectrometry analyses on Thermo Q Exactive™ Plus mass spectrometer.

2. Synthesis and characterization of compounds

2a. Synthesis of 4-fluoro-2-isopropylbenzoic acid (4a-H)

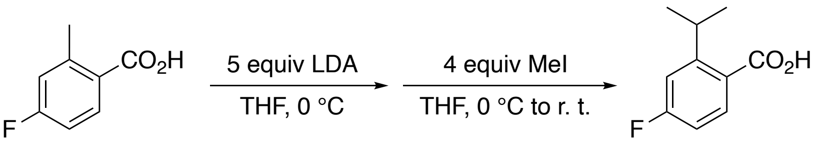

A 250 mL round bottom flask was charged with 4-fluoro-2-methylbenzoic acid (1056 mg, 8.05 mmol, 1 equiv) and 40 mL anhydrous THF, purged with N_2, and cooled to 0 °C in an ice-water bath. Lithium diisopropylamide (LDA) solution (20.0 mL 2.0 M in THF/heptane/ethylbenzene, 40.0 mmol, 5.0 equiv) was slowly added to the flask. The mixture was stirred at 0 °C for 1 h. Methyl iodide (2.00 mL, 32.0 mmol, 4.0 equiv) was dissolved in 10 mL hexane and added to the reaction mixture dropwise via a syringe. The mixture was allowed to warm to room temperature and stirred for an additional 1 h, quenched by adding 20 mL H_2O, then dried *in vacuo*. The residue was dissolved in 50 mL EtOAc, washed with 2× 10 mL 2 M HCl and 2× 20 mL H_2O, then extracted with 3× 15 mL 2 M NaOH. The combined aqueous phases were acidified with concentrated HCl until pH ~1. The crude product precipitated from the solution, and the mixture was extracted with 3× 10 mL Et_2O. The combined organic phases were washed with 5 mL 2 M HCl and 2× 5 mL H_2O, dried over $MgSO_4$, and dried *in vacuo*. The pure product **4a** was obtained as a white crystalline powder (958 mg, 65 %), without further purification.

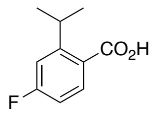

4-Fluoro-2-isopropylbenzoic acid (**4a-H**):

^{1}H NMR (600 MHz, $CDCl_3$) δ 8.01 (dd, J = 8.7, 6.1 Hz, 1H), 7.13 (dd, J = 10.7, 2.6 Hz, 1H), 6.95 (ddd, J = 8.7, 7.7, 2.6 Hz, 1H), 4.02 (heptd, J = 6.8, 1.7 Hz, 1H), 1.27 (d, J = 6.8 Hz, 6H).

^{13}C{^{1}H} NMR (151 MHz, $CDCl_3$) δ 172.34, 165.72 (d, J = 253.7 Hz), 155.30 (d, J = 7.9 Hz), 133.98 (d, J = 9.5 Hz), 123.93 (d, J = 2.9 Hz), 113.64 (d, J = 21.7 Hz), 112.80 (d, J = 21.8 Hz), 29.45 (d, J = 1.4 Hz), 23.81.

^{19}F NMR (564 MHz, $CDCl_3$) δ –105.51 (dddd, J = 10.7, 7.8, 6.1, 1.7 Hz).

HRMS (ESI) calculated: $[M–H]^-$ 181.0664, measured 181.0662.

See **Section 10a** (pages S11–S13) for NMR spectra.

2b. General procedure for the synthesis of potassium benzoates and phenylacetates

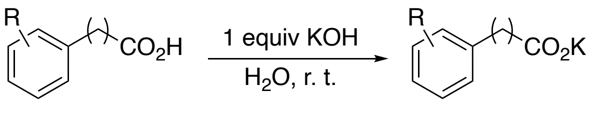

A ~ 2.5 M aqueous KOH standard solution was prepared and titrated with an aqueous potassium hydrogen phthalate standard solution with phenolphthalein indicator to obtain a concentration of 2.8857 M. Benzoic acids and phenylacetic acids were treated with 1 equivalent of the KOH standard solution, then filtered through a 0.22 µm syringe filter to remove particulate and undissolved solids. Water was removed *in vacuo*, and the potassium salts were then dried under high vacuum at 60 °C overnight to yield products as white crystalline powders, without further purification.

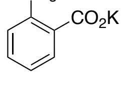

Potassium 2-(trifluoromethyl)benzoate (**1a**):

^{1}H NMR (600 MHz, $CDCl_3$) δ 7.63 (d, J = 7.9 Hz, 1H), 7.54 (d, J = 7.6 Hz, 1H), 7.43 (t, J = 7.8 Hz, 1H), 7.35 (d, J = 7.7 Hz, 1H).

^{13}C{^{1}H} NMR (151 MHz, $CDCl_3$) δ 176.86, 138.57 (q, J = 2.6 Hz), 132.23, 128.21, 126.57, 125.89 (q, J = 4.8 Hz), 124.52 (q, J = 31.5 Hz), 124.02 (q, J = 272.6 Hz).

^{19}F NMR (564 MHz, $CDCl_3$) δ -59.68.

HRMS (ESI) calculated: $[M–H]^-$ 189.0169, measured 189.0160.

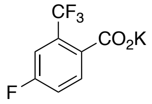

Potassium 4-fluoro-2-(trifluoromethyl)benzoate (**2a**):
^{1}H NMR (600 MHz, $CDCl_3$) δ 7.43 – 7.37 (m, 2H), 7.28 (td, J = 8.5, 2.6 Hz, 1H).
^{13}C{^{1}H} NMR (151 MHz, $CDCl_3$) δ 175.82, 161.36 (d, J = 246.1 Hz), 135.07, 129.18 (d, J = 8.3 Hz), 126.74 (qd, J = 32.7, 8.2 Hz), 123.11 (qd, J = 272.7, 3.0 Hz), 119.10 (d, J = 21.1 Hz), 113.36 (dq, J = 25.3, 5.0 Hz).
^{19}F NMR (564 MHz, $CDCl_3$) δ -60.13, -112.48 (td, J = 8.9, 5.5 Hz).
HRMS (ESI) calculated: [M–H]$^-$ 207.0075, measured 207.0069.

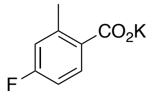

Potassium 4-fluoro-2-methylbenzoate (**3a**):
^{1}H NMR (600 MHz, D_2O) δ 7.27 (dd, J = 8.5, 6.1 Hz, 1H), 6.90 (dd, J = 10.3, 2.7 Hz, 1H), 6.85 (td, J = 8.6, 2.7 Hz, 1H), 2.28 (s, 3H).
^{13}C{^{1}H} NMR (151 MHz, D_2O) δ 178.21, 162.14 (d, J = 244.0 Hz), 137.25 (d, J = 8.3 Hz), 135.77 (d, J = 3.0 Hz), 128.20 (d, J = 8.9 Hz), 116.63 (d, J = 21.3 Hz), 112.05 (d, J = 21.4 Hz), 19.23 (d, J = 1.6 Hz).
^{19}F NMR (564 MHz, D_2O) δ -114.32 (ddd, J = 10.3, 8.8, 6.1 Hz).
HRMS (ESI) calculated: [M–H]$^-$ 153.0357, measured 153.0347.

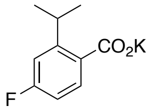

Potassium 4-fluoro-2-isopropylbenzoate (**4a**):
^{1}H NMR (600 MHz, D_2O) δ 7.16 (dd, J = 8.4, 6.1 Hz, 1H), 7.04 (dd, J = 11.2, 2.6 Hz, 1H), 6.84 (td, J = 8.7, 2.7 Hz, 1H), 3.15 (heptd, J = 6.4, 1.4 Hz, 1H), 1.11 (d, J = 6.9 Hz, 6H).
^{13}C{^{1}H} NMR (151 MHz, D_2O) δ 178.89, 162.46 (d, J = 243.1 Hz), 147.43 (d, J = 7.0 Hz), 135.68 (d, J = 3.0 Hz), 127.41, 112.19 (d, J = 10.4 Hz), 112.04 (d, J = 10.3 Hz), 30.26 (d, J = 1.6 Hz), 23.02.
^{19}F NMR (564 MHz, D_2O) δ -114.07 (dddd, J = 11.2, 8.7, 6.1, 1.8 Hz).
HRMS (ESI) calculated: [M–H]$^-$ 181.0670, measured 181.0661.

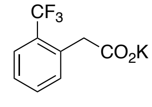

Potassium 2-(2-(trifluoromethyl)phenyl)acetate (**1b**):
^{1}H NMR (600 MHz, D_2O) δ 7.63 (d, J = 7.9 Hz, 1H), 7.50 (t, J = 7.6 Hz, 1H), 7.35 (t, J = 7.7 Hz, 1H), 7.31 (d, J = 7.7 Hz, 1H), 3.64 (s, 2H).
^{13}C{^{1}H} NMR (151 MHz, D_2O) δ 179.78, 135.25 (q, J = 1.7 Hz), 132.86, 132.22, 127.94 (q, J = 29.7 Hz), 126.87, 125.92 (q, J = 5.6 Hz), 124.59 (q, J = 273.2 Hz), 41.47 (q, J = 2.0 Hz).
^{19}F NMR (564 MHz, D_2O) δ -60.12.
HRMS (ESI) calculated: [M–H]$^-$ 203.0325, measured 203.0318.

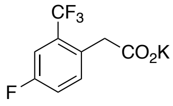

Potassium 2-(4-fluoro-2-(trifluoromethyl)phenyl)acetate (**2b**):
^{1}H NMR (600 MHz, D_2O) δ 7.39 (dd, J = 9.5, 2.8 Hz, 1H), 7.31 (dd, J = 8.6, 5.6 Hz, 1H), 7.24 (td, J = 8.5, 2.8 Hz, 1H), 3.61 (s, 2H).
^{13}C{^{1}H} NMR (151 MHz, D_2O) δ 179.58, 160.74 (d, J = 243.9 Hz), 134.87 (d, J = 8.0 Hz), 131.38 (dq, J = 3.6, 1.7 Hz), 129.54 (qd, J = 31.1, 7.7 Hz), 124.05 (qd, J = 273.2, 3.0 Hz), 118.89 (d, J = 20.9 Hz), 113.34 (dq, J = 25.3, 5.8 Hz), 40.68 (q, J = 2.0 Hz).
^{19}F NMR (564 MHz, D_2O) δ -60.77, -115.55 (td, J = 8.9, 5.6 Hz).
HRMS (ESI) calculated: [M–H]$^-$ 221.0231, measured 221.0226.

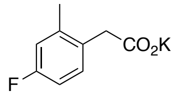

Potassium 2-(4-fluoro-2-methylphenyl)acetate (**3b**):
^{1}H NMR (600 MHz, D_2O) δ 7.06 (dd, J = 8.4, 6.1 Hz, 1H), 6.89 (dd, J = 10.2, 2.8 Hz, 1H), 6.83 (td, J = 8.7, 2.9 Hz, 1H), 3.42 (s, 2H), 2.14 (s, 3H).
^{13}C{^{1}H} NMR (151 MHz, D_2O) δ 180.69 (d, J = 1.2 Hz), 161.33 (d, J = 241.0 Hz), 139.62, 131.81 (d, J = 3.0 Hz), 131.59 (d, J = 8.5 Hz), 116.37 (d, J = 21.1 Hz), 112.23 (d, J = 20.8 Hz), 41.49, 18.70 (d, J = 1.6 Hz).
^{19}F NMR (564 MHz, D_2O) δ -117.85 (ddd, J = 10.2, 8.9, 6.1 Hz).
HRMS (ESI) calculated: [M–H]$^-$ 167.0514, measured 167.0503.

See **Section 10a** (pages S2–S25) for NMR spectra.

2c. General procedure for the synthesis of Ac- protected MPAA ligands

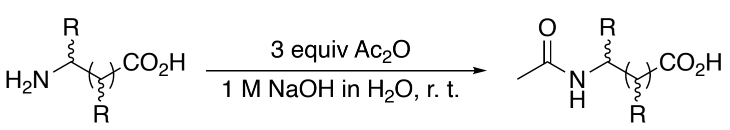

24 mL glass vials were charged with the amino acid substrates, acetic anhydride (3 equiv), and 1 M NaOH. The mixtures were stirred at room temperature overnight and acidified with concentrated HCl until pH ~1. The mixtures were extracted with EtOAc, the combined organic phases were dried over $MgSO_4$, then dried *in vacuo*. The crude products were recrystallized by dissolving in a minimal amount of MeOH and cooling to −18 °C.

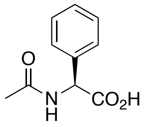

N-acetyl-*L*-phenylglycine (**Ac-*α*-*L*-Ph-OH**):
^{1}H NMR (600 MHz, MeOD-d_4) δ 7.44 – 7.39 (m, 2H), 7.37 (m, 2H), 7.35 – 7.31 (m, 1H), 5.43 (s, 1H), 2.01 (s, 3H).
^{13}C{^{1}H} NMR (151 MHz, MeOD-d_4) δ 173.73, 172.89, 138.04, 129.80, 129.38, 128.80, 58.24, 22.25.
HRMS (ESI) calculated: [M–H]$^{-}$ 192.0666, measured 192.0659.

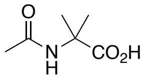

N-acetyl-*α*,*α*-dimethylglycine (**Ac-*α*-Me$_2$-OH**):
^{1}H NMR (600 MHz, MeOD-d_4) δ 1.91 (s, 3H), 1.45 (s, 6H).
^{13}C{^{1}H} NMR (151 MHz, MeOD-d_4) δ 178.00, 172.61, 56.82, 25.35, 22.53.
HRMS (ESI) calculated: [M–H]$^{-}$ 144.0666, measured 144.0656.

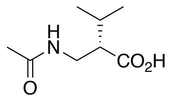

N-acetyl-β^{2}-*L*-homovaline (**Ac-β^{2}-*L*-Val-OH**):
^{1}H NMR (600 MHz, MeOD-d_4) δ 3.46 (dd, J = 13.4, 4.7 Hz, 1H), 3.27 (dd, J = 13.4, 9.5 Hz, 1H), 2.41 (ddd, J = 9.5, 6.7, 4.7 Hz, 1H), 1.91 (heptd, J = 6.8, 6.8 Hz, 1H), 1.91 (s, 3H), 0.99 (dd, J = 19.4, 6.8 Hz, 6H).
^{13}C{^{1}H} NMR (151 MHz, MeOD-d_4) δ 177.50, 173.39, 53.07, 40.36, 30.01, 22.46, 20.56, 20.35.
HRMS (ESI) calculated: [M–H]$^{-}$ 172.0979, measured 172.0969.

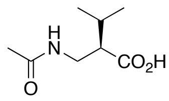

N-acetyl-β^{2}-*D*-homovaline (**Ac-β^{2}-*D*-Val-OH**):
^{1}H NMR (600 MHz, MeOD-d_4) δ 3.46 (dd, J = 13.4, 4.7 Hz, 1H), 3.27 (dd, J = 13.4, 9.5 Hz, 1H), 2.41 (ddd, J = 9.5, 6.7, 4.7 Hz, 1H), 1.91 (heptd, J = 6.8, 6.8 Hz, 1H), 1.91 (s, 3H), 0.99 (dd, J = 19.4, 6.8 Hz, 6H).
^{13}C{^{1}H} NMR (151 MHz, MeOD-d_4) δ 177.50, 173.39, 53.07, 40.36, 30.00, 22.46, 20.56, 20.35.
HRMS (ESI) calculated: [M–H]$^{-}$ 172.0979, measured 172.0970.

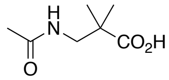

N-acetyl-2,2-dimethyl-*β*-alanine (**Ac-β^{2}-Me$_2$-OH**):
^{1}H NMR (600 MHz, MeOD-d_4) δ 3.34 (s, 2H), 1.95 (s, 3H), 1.16 (s, 6H).
^{13}C{^{1}H} NMR (151 MHz, MeOD-d_4) δ 180.23, 173.59, 47.97, 44.22, 23.53, 22.52.
HRMS (ESI) calculated: [M–H]$^{-}$ 158.0823, measured 158.0813.

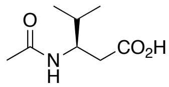

N-acetyl-β^{3}-*L*-leucine (**Ac-β^{3}-*L*-Val-OH**):
^{1}H NMR (600 MHz, MeOD-d_4) δ 4.11 (ddd, J = 8.8, 6.0, 4.8 Hz, 1H), 2.51 (dd, J = 15.3, 4.8 Hz, 1H), 2.37 (dd, J = 15.3, 8.9 Hz, 1H), 1.94 (s, 3H), 1.81 (dh, J = 13.7, 6.8 Hz, 1H), 0.92 (dd, J = 6.8, 3.9 Hz, 6H).
^{13}C{^{1}H} NMR (151 MHz, MeOD-d_4) δ 175.21, 172.84, 53.00, 37.60, 33.09, 22.58, 19.42, 18.49.
HRMS (ESI) calculated: [M–H]$^{-}$ 172.0979, measured 172.0970.

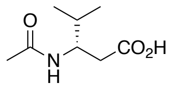

N-acetyl-β^{3}-*D*-leucine (**Ac-β^{3}-*D*-Val-OH**):
^{1}H NMR (600 MHz, MeOD-d_4) δ 4.11 (ddd, J = 8.8, 6.1, 4.8 Hz, 1H), 2.51 (dd, J = 15.3, 4.8 Hz, 1H), 2.37 (dd, J = 15.3, 8.9 Hz, 1H), 1.94 (s, 3H), 1.81 (dh, J = 13.6, 6.8 Hz, 1H), 0.92 (dd, J = 6.8, 3.9 Hz, 6H).
^{13}C{^{1}H} NMR (151 MHz, MeOD-d_4) δ 175.20, 172.84, 52.99, 37.59, 33.09, 22.58, 19.42, 18.49.
HRMS (ESI) calculated: [M–H]$^{-}$ 172.0979, measured 172.0970.

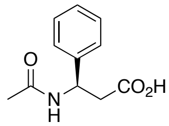

N-acetyl-β^{3}-*L*-phenylalanine (**Ac-β^{3}-*L*-Ph-OH**):
^{1}H NMR (600 MHz, MeOD-d_4) δ 7.39 – 7.28 (m, 4H), 7.27 – 7.21 (m, 1H), 5.34 (dd, J = 8.4, 6.5 Hz, 1H), 2.84 – 2.71 (m, 2H), 1.95 (s, 3H).
^{13}C{^{1}H} NMR (151 MHz, MeOD-d_4) δ 174.05, 172.37, 142.91, 129.59, 128.46, 127.62, 51.58, 41.62, 22.59.
HRMS (ESI) calculated: [M–H]$^{-}$ 206.0823, measured 206.0816.

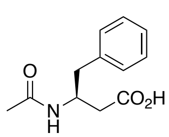

N-acetyl-β^{3}-*L*-homophenylalanine (**Ac-β^{3}-*L*-Phe-OH**):
^{1}H NMR (600 MHz, MeOD-d_4) δ 7.28 (m, 2H), 7.24 – 7.17 (m, 3H), 4.42 (ddd, J = 13.8, 7.5, 6.2 Hz, 1H), 2.89 – 2.77 (m, 2H), 2.51 – 2.39 (m, 2H), 1.85 (s, 3H).
^{13}C{^{1}H} NMR (151 MHz, MeOD-d_4) δ 174.72, 172.61, 139.46, 130.39, 129.39, 127.53, 49.40, 41.08, 39.19, 22.57.
HRMS (ESI) calculated: [M–H]$^{-}$ 220.0979, measured 220.0975.

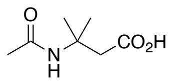

N-acetyl-3,3-dimethyl-*β*-alanine (**Ac-β^{3}-Me$_2$-OH**):
^{1}H NMR (600 MHz, MeOD-d_4) δ 2.79 (s, 2H), 1.88 (s, 3H), 1.39 (s, 6H).
^{13}C{^{1}H} NMR (151 MHz, MeOD-d_4) δ 174.78, 172.98, 52.77, 43.93, 27.44, 23.53.
HRMS (ESI) calculated: [M–H]$^{-}$ 158.0823, measured 158.0813.

See **Section 10b** (pages S26–S45) for NMR spectra.

	Amino acids		MPAA ligands	
α	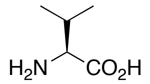	*L*-valine<br>*α*-*L*-Val-OH (CAS#: 72-18-4)	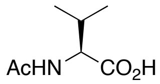	*N*-acetyl-*L*-valine*<br>Ac-*α*-*L*-Val-OH (CAS#: 96-81-1)
	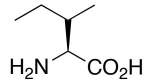	*L*-isoleucine<br>*α*-*L*-Ile-OH (CAS#: 73-32-5)	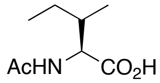	*N*-acetyl-*L*-isoleucine*<br>Ac-*α*-*L*-Ile-OH (CAS#: 3077-46-1)
	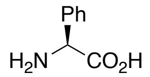	*L*-phenylglycine<br>*α*-*L*-Ph-OH (CAS#: 2935-35-5)	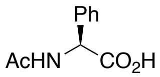	*N*-acetyl-*L*-phenylglycine<br>Ac-*α*-*L*-Ph-OH (CAS#: 42429-20-9)
	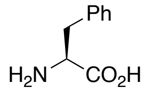	*L*-phenylalanine<br>*α*-*L*-Phe-OH (CAS#: 63-91-2)	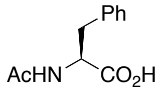	*N*-acetyl-*L*-phenylalanine*<br>Ac-*α*-*L*-Phe-OH (CAS#: 2018-61-3)
	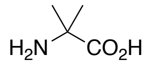	*α*,*α*-dimethylglycine<br>*α*-Me$_2$-OH (CAS#: 62-57-7)	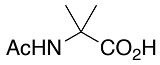	*N*-acetyl-*α*,*α*-dimethylglycine<br>Ac-*α*-Me$_2$-OH (CAS#: 62-57-7)
***β*^{2}**	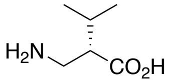	β^2-*L*-homovaline<br>β^2-*L*-Val-OH (CAS#: 203854-54-0)	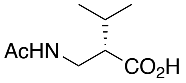	*N*-acetyl-β^2-*L*-homovaline<br>Ac-β^2-*L*-Val-OH (No CAS#)
	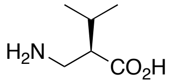	β^2-*D*-homovaline<br>β^2-*D*-Val-OH (CAS#: 210345-86-1)	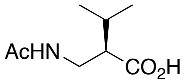	*N*-acetyl-β^2-*D*-homovaline<br>Ac-β^2-*D*-Val-OH (No CAS#)
	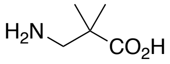	2,2-dimethyl-*β*-alanine<br>β^2-Me$_2$-OH (CAS#: 19036-43-2)	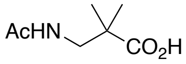	*N*-acetyl-2,2-dimethyl-*β*-alanine<br>Ac-β^2-Me$_2$-OH (CAS#: 80253-47-0)
***β*^{3}**	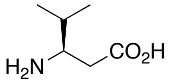	β^3-*L*-leucine<br>β^3-*L*-Val-OH (CAS#: 75992-50-6)	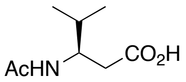	*N*-acetyl-β^3-*L*-leucine<br>Ac-β^3-*L*-Val-OH (CAS#: 220472-44-6)
	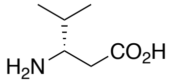	β^3-*D*-leucine<br>β^3-*D*-Val-OH (CAS#: 40469-85-0)	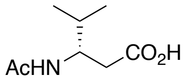	*N*-acetyl-β^3-*D*-leucine<br>Ac-β^3-*D*-Val-OH (CAS#: 162921-78-0)
	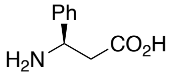	β^3-*L*-phenylalanine<br>β^3-*L*-Ph-OH (CAS#: 13921-90-9)	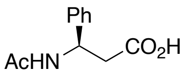	*N*-acetyl-β^3-*L*-phenylalanine<br>Ac-β^3-*L*-Ph-OH (CAS#: 117020-31-2)
	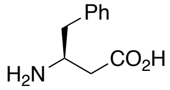	β^3-*L*-homophenylalanine<br>β^3-*L*-Phe-OH (CAS#: 26250-87-3)	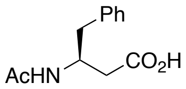	*N*-acetyl-β^3-*L*-homophenylalanine<br>Ac-β^3-*L*-Phe-OH (CAS#: 162921-79-1)
	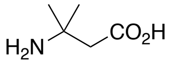	3,3-dimethyl-*β*-alanine<br>β^3-Me$_2$-OH (CAS#: 625-05-8)	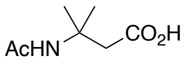	*N*-acetyl-3,3-dimethyl-*β*-alanine<br>Ac-β^3-Me$_2$-OH (CAS#: 63879-04-9)

Table S1. MPAA ligands synthesized and used in the study and their corresponding amino acids.

Compounds marked with * were purchased from Combi-Blocks.

Note: For synthesis of Ac-β^3-Me$_2$-OH, the HCl salt of β^2-Me$_2$-OH (3,3-dimethyl-*β*-alanine hydrochloride, CAS#: 2843-19-8) was used instead without modification to the procedure.

2d. Synthesis of 5-membered palladacycles (1a-PdCycle-MePy and 1a-PdCycle-^{t}BuPy)

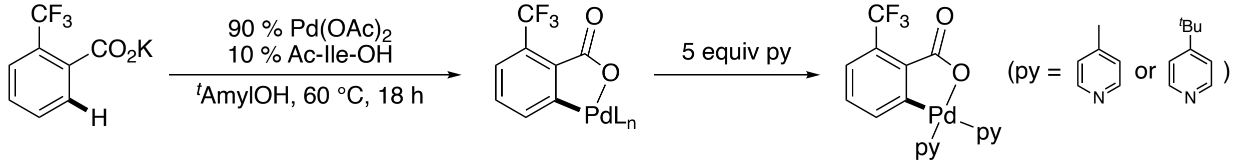

Two 24 mL glass vials were each charged with potassium 2-trifluoromethylbenzoate (292 mg, 1.28 mmol, 1 equiv), palladium acetate (259 mg, 1.15 mmol, 0.9 equiv), *N*-acetyl-*L*-isoleucine (Ac-Ile-OH, 22.2 mg, 0.13 mmol, 0.10 equiv), and 16.0 mL ^{t}AmylOH. The mixtures were stirred at 60 °C for 18 h, then cooled to r. t. To each reaction mixture was added 4-methylpyridine (596 mg, 0.62 mL, 6.40 mmol, 5 equiv) or 4-*tert*-butylpyridine (865 mg, 0.94 mL, 6.40 mmol, 5 equiv), the reaction mixtures immediately changed from bright yellow to very pale yellow color, and off-white to light gray colored precipitates formed within several minutes. The mixtures were filtered, and the solids were washed with ^{t}AmylOH then Et_2O, then dried *in vacuo* to afford products as white crystalline powder, without further purification. The synthesis procedures were repeated at smaller scales, which yielded product precipitates containing X-ray quality single crystals (see **Sections 8a** and **8b** for X-ray crystallography details).

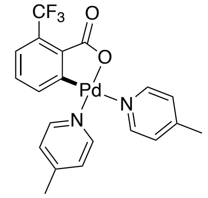

4-methylpyridine supported 5-membered palladacycle (**1a-PdCycle-MePy**):

^{1}H NMR (600 MHz, $CDCl_3$) δ 8.83 – 8.79 (m, 2H), 8.10 – 8.06 (m, 2H), 7.36 – 7.32 (m, 3H), 6.96 (d, J = 5.7 Hz, 2H), 6.87 (t, J = 7.7 Hz, 1H), 6.15 (d, J = 7.6 Hz, 1H), 2.49 (s, 3H), 2.26 (s, 3H)
^{13}C{^{1}H} NMR (151 MHz, $CDCl_3$) δ 176.58, 152.43, 151.00, 149.89, 149.14, 147.35, 139.35, 134.84, 128.80 (q, J = 31.4 Hz), 128.19, 127.49, 125.77, 123.32 (q, J = 274.7 Hz), 122.76 (q, J = 6.6 Hz), 21.35, 21.09.
^{19}F NMR (564 MHz, $CDCl_3$) δ –58.49
HRMS (ESI) calculated: $[M+H]^+$ 481.0350, measured 481.0351.

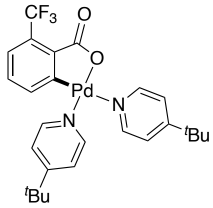

4-*tert*-butylpyridine supported 5-membered palladacycle (**1a-PdCycle-^{t}BuPy**):

^{1}H NMR (600 MHz, $CDCl_3$) δ 8.85 – 8.81 (m, 2H), 8.20 – 8.16 (m, 2H), 7.50 – 7.45 (m, 2H), 7.36 (dd, J = 7.8, 1.1 Hz, 1H), 7.21 – 7.17 (m, 2H), 6.89 (t, J = 7.7 Hz, 1H), 6.17 (dd, J = 7.7, 1.1 Hz, 1H), 1.38 (s, 9H), 1.24 (s, 9H).
^{13}C NMR (151 MHz, $CDCl_3$) δ 176.61, 163.69, 162.59, 152.49, 149.37, 147.45, 139.42, 134.88, 128.86 (q, J = 31.0 Hz), 128.22, 123.67, 123.34 (q, J = 273.8 Hz), 122.82 (q, J = 6.7 Hz), 122.08, 35.37, 34.98, 30.27, 30.22.
^{19}F NMR (564 MHz, $CDCl_3$) δ –58.37.
HRMS (ESI) calculated: $[M+H]^+$ 565.1289, measured 565.1288.

See **Section 10c** (pages S46–S53) for NMR spectra.

2e. Synthesis of 6-membered palladacycle (1b-PdCycle-MePy)

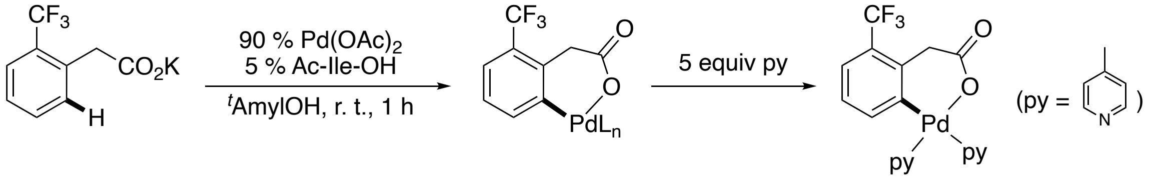

A 24 mL glass vial was charged with potassium 2-(trifluoromethyl)phenylacetate (96.7 mg, 0.40 mmol, 1 equiv), palladium acetate (81.6 mg, 0.36 mmol, 0.9 equiv), *N*-acetyl-*L*-isoleucine (Ac-Ile-OH, 3.5 mg, 0.02 mmol, 0.05 equiv), and 10.0 mL ^{t}AmylOH. The mixture was stirred at room temperature for 1 h. 4-Methylpyridine (0.20 mL, 2.00 mmol, 5 equiv) was added, the reaction mixture immediately changed from bright yellow to very pale yellow color, and a yellowish-gray colored precipitate began to form slowly. The mixture was dried *in vacuo*, and the residue was dissolved in DCM, filtered through a 0.22 µm syringe filter, and dried *in vacuo* again to afford the product as an ivory solid, without further purification. A small amount of the solid was placed in a 2 mL glass vial and dissolved in a minimal amount of DCM. Et_2O was added dropwise until a saturation point was reached. X-ray quality single crystals formed after 24 h.

The crystalline product obtained as a precipitate from the reaction mixture was revealed by NMR characterization as a 1:1 co-crystal with ^{t}AmylOH. X-ray crystallography analysis of the recrystallized single crystals showed similar characteristics, though the sample contained several different kinds of solvent molecules in the crystal lattice. See **Section 8c** for X-ray crystallography details.

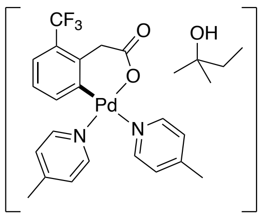

4-methylpyridine supported 6-membered palladacycle (**1b-PdCycle-MePy**):
^{1}H NMR (600 MHz, $CDCl_3$) δ 8.47 (d, J = 5.3 Hz, 2H), 8.14 (d, J = 5.4 Hz, 2H), 7.23 (d, J = 7.7 Hz, 1H), 7.19 (d, J = 5.8 Hz, 2H), 7.12 – 7.08 (m, 2H), 6.68 (t, J = 7.7 Hz, 1H), 6.50 (d, J = 7.6 Hz, 1H), 4.27 (s, 2H), 2.44 (s, 3H), 2.34 (s, 3H).
^{13}C{^{1}H} NMR (151 MHz, $CDCl_3$) δ 176.35, 152.60, 150.91, 150.03, 138.70, 127.02, 125.33, 123.55, 123.51, 121.78, 46.51, 21.31, 21.13.
^{19}F NMR (564 MHz, $CDCl_3$) δ –58.57.
HRMS (ESI) calculated: $[M+H]^+$ 495.0506, measured 495.0499.

See **Section 10c** (pages S54–S58) for NMR spectra.

2f. General procedure for the synthesis of benzoic acid C–H arylation products

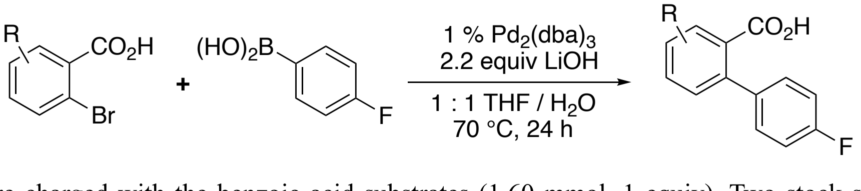

24 mL glass vials were charged with the benzoic acid substrates (1.60 mmol, 1 equiv). Two stock solutions containing 4-fluorophenylboronic acid (391 mg, 1.76 mmol, 1.1 equiv) in 6.0 mL THF and LiOH (84.3 mg, 3.52 mmol, 2.2 equiv) in 8.0 mL H_2O were added to each vial. The mixtures were stirred gently until all solids dissolved. A third stock solution containing $Pd_2(dba)_3$ (14.7 mg, 0.016 mmol as dimers, 0.01 equiv or 2 % Pd loading) in 2.0 mL THF was added to each vial. The mixtures were stirred at 70 °C for 24 h. Volatiles were removed *in vacuo*, and 10 mL of 2 M HCl was added to the residue, then extracted with EtOAc. The combined organic phases were dried over $MgSO_4$, then dried *in vacuo*. The crude products were purified using column chromatography over silica gel, using MeOH / DCM as eluents, then recrystallized using DCM / *n*-heptane to yield products as white to ivory-colored crystalline powders.

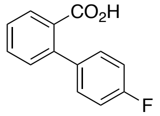

2-(4-fluorophenyl)benzoic acid (**P5-1**):
^{1}H NMR (600 MHz, $CDCl_3$) δ 11.63 (s, 1H), 7.96 (dd, J = 7.8, 1.4 Hz, 1H), 7.43 (td, J = 7.6, 1.3 Hz, 1H), 7.33 (dd, J = 7.7, 1.3 Hz, 1H), 7.30 – 7.26 (m, 2H), 7.10 – 7.04 (m, 2H).
^{13}C{^{1}H} NMR (151 MHz, $CDCl_3$) δ 173.26, 162.37 (d, J = 246.3 Hz), 142.51, 137.02 (d, J = 3.4 Hz), 132.27, 131.26, 130.87, 130.08 (d, J = 8.1 Hz), 129.09, 127.39, 115.00 (d, J = 21.5 Hz).
^{19}F NMR (564 MHz, $CDCl_3$) δ –115.34 (tt, J = 8.7, 5.3 Hz).
HRMS (ESI) calculated: $[M–H]^-$ 215.0514, measured 215.0508.

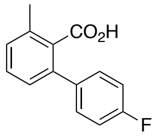

2-(4-fluorophenyl)-6-methylbenzoic acid (**P5-2**):
^{1}H NMR (600 MHz, $CDCl_3$) δ 11.06 (s, 1H), 7.40 – 7.33 (m, 3H), 7.25 – 7.23 (m, 1H), 7.18 (dd, J = 7.8, 1.2 Hz, 1H), 7.11 – 7.04 (m, 2H), 2.45 (s, 3H).
^{13}C{^{1}H} NMR (151 MHz, $CDCl_3$) δ 174.76, 162.47 (d, J = 246.8 Hz), 139.14, 136.60 (d, J = 3.3 Hz), 135.50, 132.07, 130.03 (d, J = 8.1 Hz), 129.82, 129.37, 127.43, 115.30 (d, J = 21.4 Hz), 19.88.
^{19}F NMR (564 MHz, $CDCl_3$) δ –114.91 (tt, J = 8.7, 5.3 Hz).
HRMS (ESI) calculated: $[M–H]^-$ 229.0670, measured 229.0667.

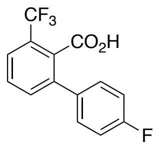

2-(4-fluorophenyl)-6-(trifluoromethyl)benzoic acid (**P5-3**):
^{1}H NMR (600 MHz, $CDCl_3$) δ 10.52 (s, 1H), 7.73 (dd, J = 7.9, 1.2 Hz, 1H), 7.61 (td, J = 7.8, 1.0 Hz, 1H), 7.55 (dd, J = 7.8, 1.2 Hz, 1H), 7.39 – 7.33 (m, 2H), 7.14 – 7.07 (m, 2H).
^{13}C{^{1}H} NMR (151 MHz, $CDCl_3$) δ 172.21, 162.88 (d, J = 248.1 Hz), 140.20, 134.66 (d, J = 3.4 Hz), 133.66, 130.31 (d, J = 8.3 Hz), 130.23 (q, J = 2.2 Hz), 129.99, 127.88 (q, J = 32.3 Hz), 125.24 (q, J = 4.8 Hz), 123.30 (q, J = 273.9 Hz), 115.61 (d, J = 21.7 Hz).
^{19}F NMR (564 MHz, $CDCl_3$) δ –59.40, –113.48 (tt, J = 8.6, 5.2 Hz).
HRMS (ESI) calculated: $[M–H]^-$ 283.0388, measured 283.0388.

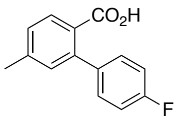

2-(4-fluorophenyl)-4-methylbenzoic acid (**P5-4**):
^{1}H NMR (600 MHz, $CDCl_3$) δ 11.77 (s, 1H), 7.89 (d, J = 8.0 Hz, 1H), 7.28 – 7.24 (m, 2H), 7.24 – 7.21 (m, 1H), 7.14 – 7.10 (m, 1H), 7.09 – 7.02 (m, 2H), 2.42 (s, 3H).
^{13}C{^{1}H} NMR (151 MHz, $CDCl_3$) δ 173.16, 162.27 (d, J = 246.1 Hz), 143.05, 142.80, 137.28 (d, J = 3.3 Hz), 132.15, 131.23, 130.05 (d, J = 8.1 Hz), 128.10, 126.07, 114.85 (d, J = 21.4 Hz), 21.50.
^{19}F NMR (564 MHz, $CDCl_3$) δ –115.60 (tt, J = 8.7, 5.3 Hz).
HRMS (ESI) calculated: $[M–H]^-$ 229.0670, measured 229.0667.

2-(4-fluorophenyl)-4-(trifluoromethyl)benzoic acid (**P5-5**):

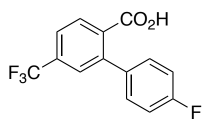

^{1}H NMR (600 MHz, $CDCl_3$) δ 11.39 (s, 1H), 8.06 (d, J = 8.2 Hz, 1H), 7.70 (dd, J = 8.3, 1.8 Hz, 1H), 7.61 (d, J = 1.9 Hz, 1H), 7.33 – 7.27 (m, 2H), 7.14 – 7.08 (m, 2H).
$^{13}C\{^1H\}$ NMR (151 MHz, $CDCl_3$) δ 172.14, 162.76 (d, J = 247.8 Hz), 143.10, 135.51 (d, J = 3.5 Hz), 133.94 (q, J = 32.9 Hz), 132.33, 131.27, 130.11 (d, J = 8.1 Hz), 128.06 (q, J = 3.7 Hz), 124.23 (q, J = 3.6 Hz), 123.05 (q, J = 273.1 Hz), 115.39 (d, J = 21.7 Hz).
^{19}F NMR (564 MHz, $CDCl_3$) δ –63.15, –113.89 (tt, J = 8.6, 5.2 Hz).
HRMS (ESI) calculated: $[M–H]^-$ 283.0388, measured 283.0387.

See **Section 10d** (pages S59–S73) for NMR spectra.

2g. General procedure for the synthesis of phenylacetic acid C–H arylation products

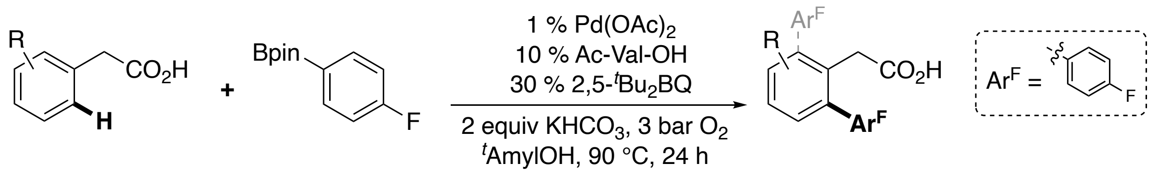

25 mL thick-walled glass vials were charged with the phenylacetic acid substrates (1.00 mmol, 1 equiv), 4-fluorophenylboronic acid pinacol ester (666 mg, 0.65 mL, 3.00 mmol, 3 equiv), and $KHCO_3$ (220 mg, 2.00 mmol, 2 equiv). Two stock solutions A and B were prepared in ^{t}AmylOH. The vials were each charged with 2.00 mL of stock solution A containing *N*-acetyl-*L*-valine (Ac-Val-OH, 15.9 mg, 0.10 mmol, 0.1 equiv) and 2,5-di-*tert*-butylbenzoquinone (66.1 mg, 0.30 mmol, 0.3 equiv), and 3.00 mL of stock solution B containing palladium acetate (2.2 mg, 0.010 mmol, 0.01 equiv). The vials were equipped with cross-shaped magnetic stir bars and sealed with crimp caps fitted with septa. O_2 lines were attached to each vial by puncturing the septa with needles, and the headspace was evacuated and filled with 3 atm O_2 six times. The vials were then heated to 90 °C and stirred vigorously for 24 h. The vials were cooled to room temperature and each charged with 2.0 mL 2 M HCl, the mixtures were then dried *in vacuo*. The crude products were purified using column chromatography over silica gel, using MeOH / DCM as eluents, then recrystallized using DCM / *n*-heptane to yield products as white to ivory-colored crystalline powders.

Note: Due to the high reactivity of phenylacetic acid substrates in the C–H arylation reaction, substrates without *ortho*-substituents produced di-arylation major products, which were isolated in lieu of their mono-arylation analogs.

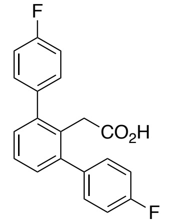

[2,6-di-(4-fluorophenyl)phenyl]acetic acid (**P6-1-2**):
^{1}H NMR (600 MHz, $CDCl_3$) δ 10.67 (s, 1H), 7.40 – 7.35 (m, 1H), 7.31 – 7.22 (m, 6H), 7.12 – 7.06 (m, 4H), 3.50 (s, 2H).
$^{13}C\{^1H\}$ NMR (151 MHz, $CDCl_3$) δ 177.53, 162.23 (d, J = 246.6 Hz), 142.55, 137.21 (d, J = 3.4 Hz), 130.69 (d, J = 8.0 Hz), 129.64, 129.49, 115.25 (d, J = 21.3 Hz), 36.43.
^{19}F NMR (564 MHz, $CDCl_3$) δ –114.88 (tt, J = 8.7, 5.4 Hz).
HRMS (ESI) calculated: $[M–H]^-$ 323.0889, measured 323.0887.

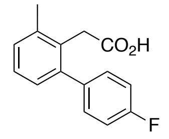

[2-(4-fluorophenyl)-6-methylphenyl]acetic acid (**P6-2**):
^{1}H NMR (600 MHz, $CDCl_3$) δ 11.28 (s, 1H), 7.28 – 7.20 (m, 4H), 7.13 – 7.05 (m, 3H), 3.62 (s, 2H), 2.35 (s, 3H).
$^{13}C\{^1H\}$ NMR (151 MHz, $CDCl_3$) δ 177.72, 162.15 (d, J = 246.2 Hz), 142.30, 137.74, 137.58 (d, J = 3.4 Hz), 130.72 (d, J = 7.9 Hz), 130.07, 129.71, 128.03, 127.24, 115.15 (d, J = 21.4 Hz), 36.01, 20.21.
^{19}F NMR (564 MHz, $CDCl_3$) δ –115.41 (tt, J = 8.7, 5.4 Hz).
HRMS (ESI) calculated: $[M–H]^-$ 243.0827, measured 243.0824.

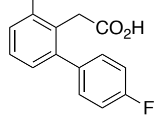

[2-(4-fluorophenyl)-6-(trifluoromethyl)phenyl]acetic acid (**P6-3**):
^{1}H NMR (600 MHz, $CDCl_3$) δ 11.12 (s, 1H), 7.72 (dd, J = 7.5, 1.8 Hz, 1H), 7.45 (t, J = 7.6 Hz, 1H), 7.42 (dd, J = 7.8, 1.8 Hz, 1H), 7.25 – 7.21 (m, 2H), 7.13 – 7.08 (m, 2H), 3.77 (s, 2H).
$^{13}C\{^1H\}$ NMR (151 MHz, $CDCl_3$) δ 176.80, 162.51 (d, J = 247.7 Hz), 144.42, 135.85 (d, J = 3.3 Hz), 133.93, 130.65 (d, J = 8.1 Hz), 129.79, 129.69 (q, J = 29.7 Hz), 127.46, 125.69 (q, J = 5.7 Hz), 124.33 (q, J = 274.0 Hz), 115.50 (d, J = 21.5 Hz), 35.21 (q, J = 2.0 Hz).
^{19}F NMR (564 MHz, $CDCl_3$) δ –59.99, –114.02 (tt, J = 8.6, 5.3 Hz).
HRMS (ESI) calculated: $[M–H]^-$ 297.0544, measured 297.0542.

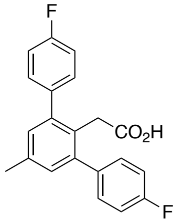

[2,6-di-(4-fluorophenyl)-4-methylphenyl]acetic acid (**P6-4-2**):

^{1}H NMR (600 MHz, $CDCl_3$) δ 10.57 (s, 1H), 7.26 (dd, J = 8.6, 5.3 Hz, 4H), 7.12 – 7.04 (m, 6H), 3.46 (s, 2H), 2.39 (s, 3H).

^{13}C{^{1}H} NMR (151 MHz, $CDCl_3$) δ 177.62, 162.17 (d, J = 246.4 Hz), 142.40, 137.35 (d, J = 3.3 Hz), 136.87, 130.65 (d, J = 8.0 Hz), 130.43, 126.46, 115.19 (d, J = 21.3 Hz), 36.02, 20.98.

^{19}F NMR (564 MHz, $CDCl_3$) δ –115.05 (tt, J = 8.6, 5.4 Hz).

HRMS (ESI) calculated: [M–H]$^-$ 337.1046, measured 337.1046.

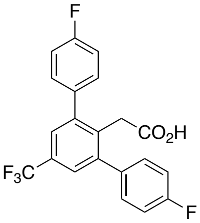

[2,6-di-(4-fluorophenyl)-4-(trifluoromethyl)phenyl]acetic acid (**P6-5-2**):

^{1}H NMR (600 MHz, $CDCl_3$) δ 10.58 (s, 1H), 7.51 (s, 2H), 7.31 – 7.26 (m, 4H), 7.16 – 7.09 (m, 4H), 3.55 (s, 2H).

^{13}C{^{1}H} NMR (151 MHz, $CDCl_3$) δ 176.72, 162.55 (d, J = 247.9 Hz), 143.35, 135.85 (d, J = 3.5 Hz), 133.55, 130.64 (d, J = 8.1 Hz), 129.50 (q, J = 32.7 Hz), 126.28 (q, J = 3.7 Hz), 123.79 (q, J = 272.6 Hz), 115.61 (d, J = 21.6 Hz), 36.40.

^{19}F NMR (564 MHz, $CDCl_3$) δ –62.56, –113.61 (tt, J = 8.6, 5.3 Hz).

HRMS (ESI) calculated: [M–H]$^-$ 391.0763, measured 391.0763.

See **Section 10d** (pages S74–S88) for NMR spectra.

3. *In situ* NMR studies of C–H activation kinetics

3a. General experimental procedures

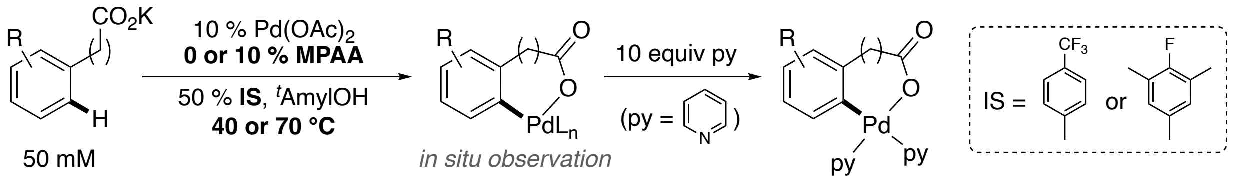

These data were recorded on a Bruker Avance III 600 MHz spectrometer with a TCI-F cryoprobe. Three stock solutions A, B, and C were prepared in volumetric flasks. For stock solutions A and B, sonication in a warm water bath was required to dissolve the solids. A screw cap NMR tube* was charged with 400 μL of stock solution A containing the potassium salt substrate (30.0 μmol, 1 equiv), MPAA ligand (3.0 μmol, 0.1 equiv), and internal standard (4-methylbenzotrifluoride for substrates with –CF_3; 2,4,6-trimethylfluorobenzene for substrates with –F only†, 15.0 μmol, 0.5 equiv) in ^{t}AmylOH. The spectrometer was set to the desired temperature, allowed to stabilize for 1 h, and calibrated to ± 0.1 °C using 100 % ethylene glycol or 80 % ethylene glycol in DMSO-d_6.[1] 200 μL of stock solution B containing palladium acetate (3.0 μmol, 0.1 equiv) in ^{t}AmylOH was injected into the NMR tube, the time of injection was recorded as the start time of the reaction. The NMR tube was shaken vigorously to allow the liquid to mix, then inserted into the spectrometer. No lock was used, and shimming was performed using the ^{t}AmylOH solvent peaks by tuning the probe to ^{1}H. The probe was then tuned to ^{19}F, and consecutive time points were recorded using the *multizg* command in Topshim. After ~ 3 h, an additional time point ("before py addition") was collected, the NMR tube was then removed from the spectrometer. 500 μL of stock solution C containing pyridine (300 μmol, 10 equiv) in acetone was added, the NMR tube was shaken vigorously to allow the liquid to mix. The reaction mixture changed from yellow or light orange colors to almost colorless. The NMR tube was returned to the spectrometer and a final spectrum ("after py addition") was collected. The data set was imported to MestReNova, the chemical shifts were referenced to the substrate peak. The concentration of the palladacycle product was determined based on integrating the ensemble of peaks consistent with palladacycle compounds with rapidly exchanging carboxylate ligands,[2] and comparing to the entire substrate and palladacycle region. After the addition of pyridine, the ensemble of palladacycles were transformed into a single palladacycle species bearing pyridine ligands, appearing as a single peak in ^{19}F NMR. The "before py addition" and "after py addition" spectra were compared against each other to confirm the quantification of palladacycles in the time course data set.

* Norell® item# S-5-600-SC-7.

† For substrates with both –CF_3 and –F substituents, 4-methylbenzotrifluoride was used as internal standard, and the data was recorded on the –CF_3 region only. ^{1}H decoupling was unavailable on the single-channel cryoprobe, and the –F region peaks appear broader and have inferior resolution and signal-to-noise ratio compared to the –CF_3 region peaks.

3b. Initial rate measurements from NMR data

The reaction rates vary greatly among different substrates. For some, an approximately linear region at the beginning (< 10% conversion) of the time course can be used to measure the initial rate. However, the reactions of some substrates are too fast, and the reactions were at > 20% conversion within a few time points. Therefore, the entirety of the time course data was used to measure the reaction rate for all substrates, by fitting to a reversible kinetics model:

$$\underset{[\text{Sub}]}{\text{Substrate}} + \underset{[\text{Pd}]}{\text{Pd(OAc)}_2} \underset{k_{-1}}{\overset{k_1}{\rightleftharpoons}} \underset{[\text{PdCycle}]}{\text{Palladacycles}}$$

Forward reaction rate $r_1 = k_1 \cdot [\text{Sub}] \cdot [\text{Pd}]$

Backward reaction rate $r_{-1} = k_{-1} \cdot [\text{PdCycle}]$

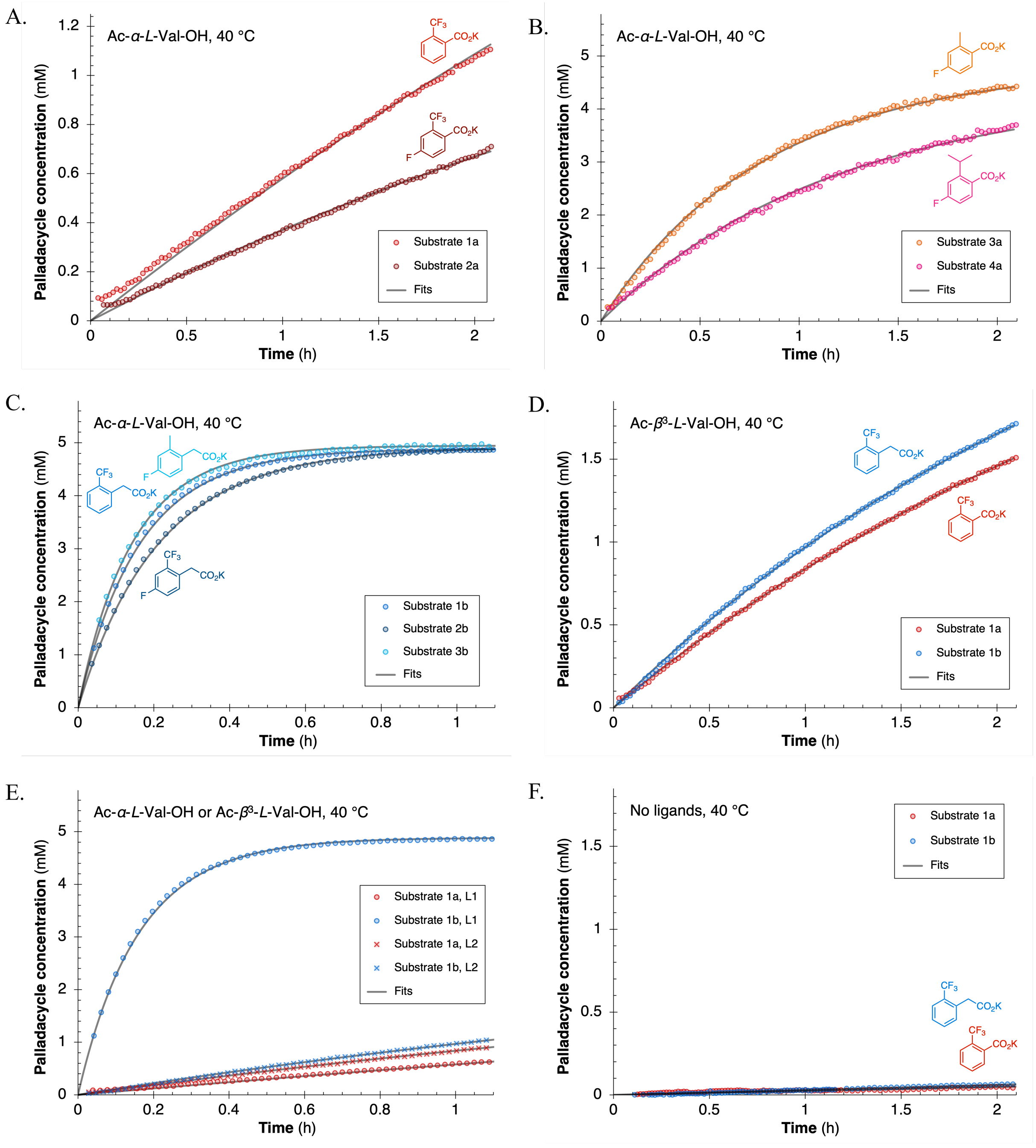

Figure S1. Experimental data sets and the fitted curves, showing a comparison of reactions between benzoate and phenylacetate substrates *using* Ac-α-*L*-Val-OH (**L1**), Ac- β^3-*L*-Val-OH (**L2**) ligand, or no ligands.

Table S2. Reaction rate constants and initial rates at 40 °C.

Substrate	Ligand	Forward rate constant k_1 ($mM^{-1} \cdot h^{-1}$)	Reverse rate constant k_{-1} ($mM^{-1} \cdot h^{-1}$)	Initial rate r_0 ($mM \cdot h^{-1}$)	Relative initial rate
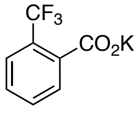 **1a**		0.002750	0.1356	0.6923	1 ×
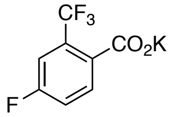 **2a**		0.001643	0.1282	0.4118	0.595 ×
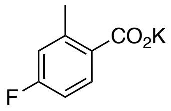 **3a**		0.02349	0.03996	5.955	8.60 ×
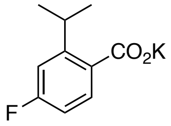 **4a**	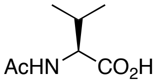 Ac-*α*-*L*-Val-OH (**L1**)	0.01468	0.08911	3.724	5.38 ×
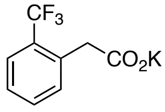 **1b**		0.1237	0.1636	31.05	44.9 ×
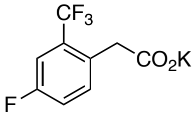 **2b**		0.09446	0.09979	23.78	34.3 ×
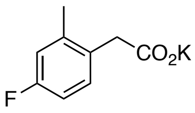 **3b**		0.1410	0.1064	35.46	51.2 ×
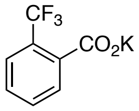 **1a**	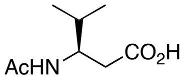 Ac-β^3-*L*-Val-OH (**L2**)	0.003841	0.09277	0.9707	1.40 ×
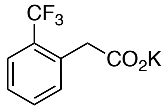 **1b**		0.004559	0.1031	1.149	1.66 ×

Alternatively, an irreversible model may also be used to fit the data:

$$\underset{[\text{Sub}]}{\text{Substrate}} + \underset{[\text{Pd}]}{\text{Pd(OAc)}_2} \xrightarrow{k} \underset{[\text{PdCycle}]}{\text{Palladacycles}} \qquad \text{Reaction rate } r = k \cdot [\text{Sub}] \cdot [\text{Pd}]$$

However, the data cannot be fitted to this model with the same quality as compared to the reversible model, especially for the phenylacetic substrates.

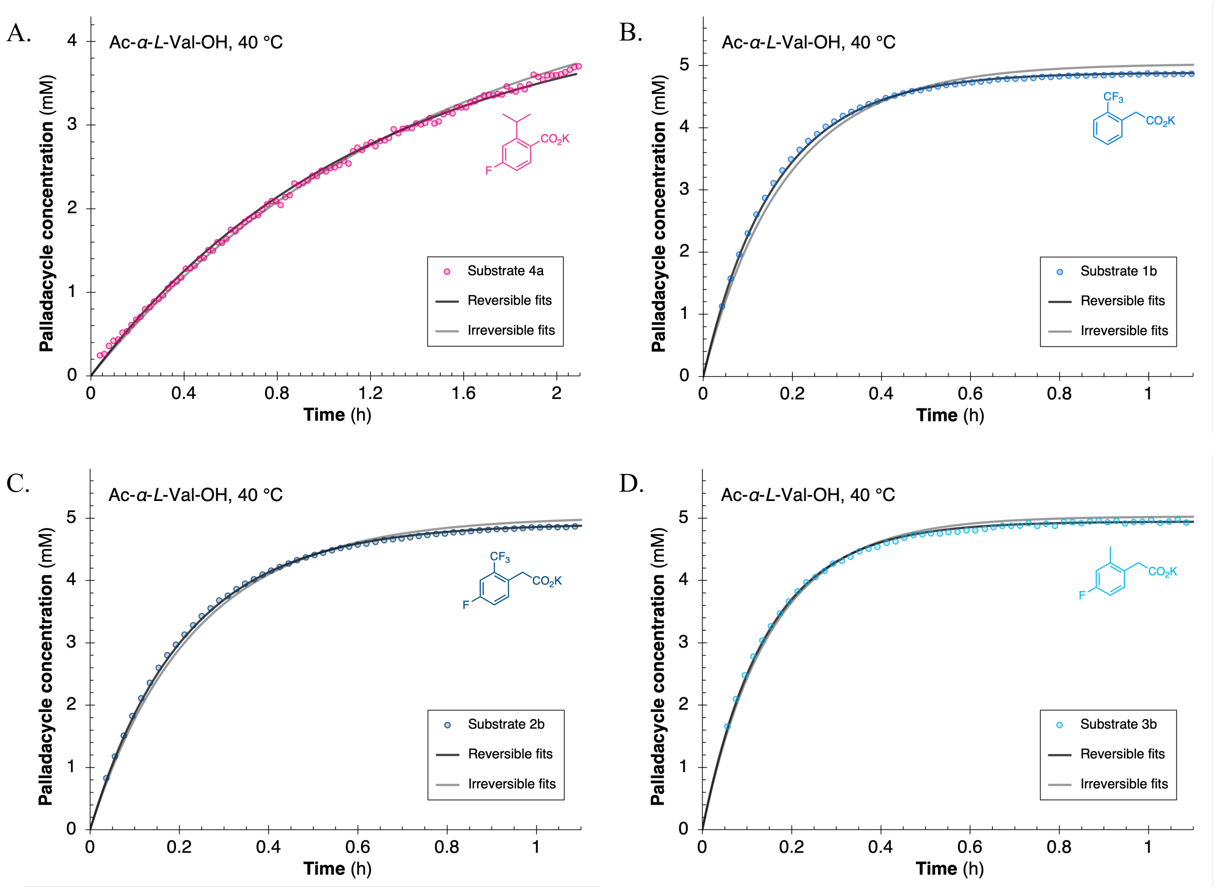

Figure S2. Comparison of data fitting qualities of both kinetic models.

3c. NMR spectra of *in situ* cyclopalladation rate measurements

(*starting from next page*)

Substrate **1a** (potassium 2-trifluoromethylbenzoate), Ac-α-L-Val-OH, 40 °C, ^{19}F NMR (^{t}AmylOH, 564.3 MHz):

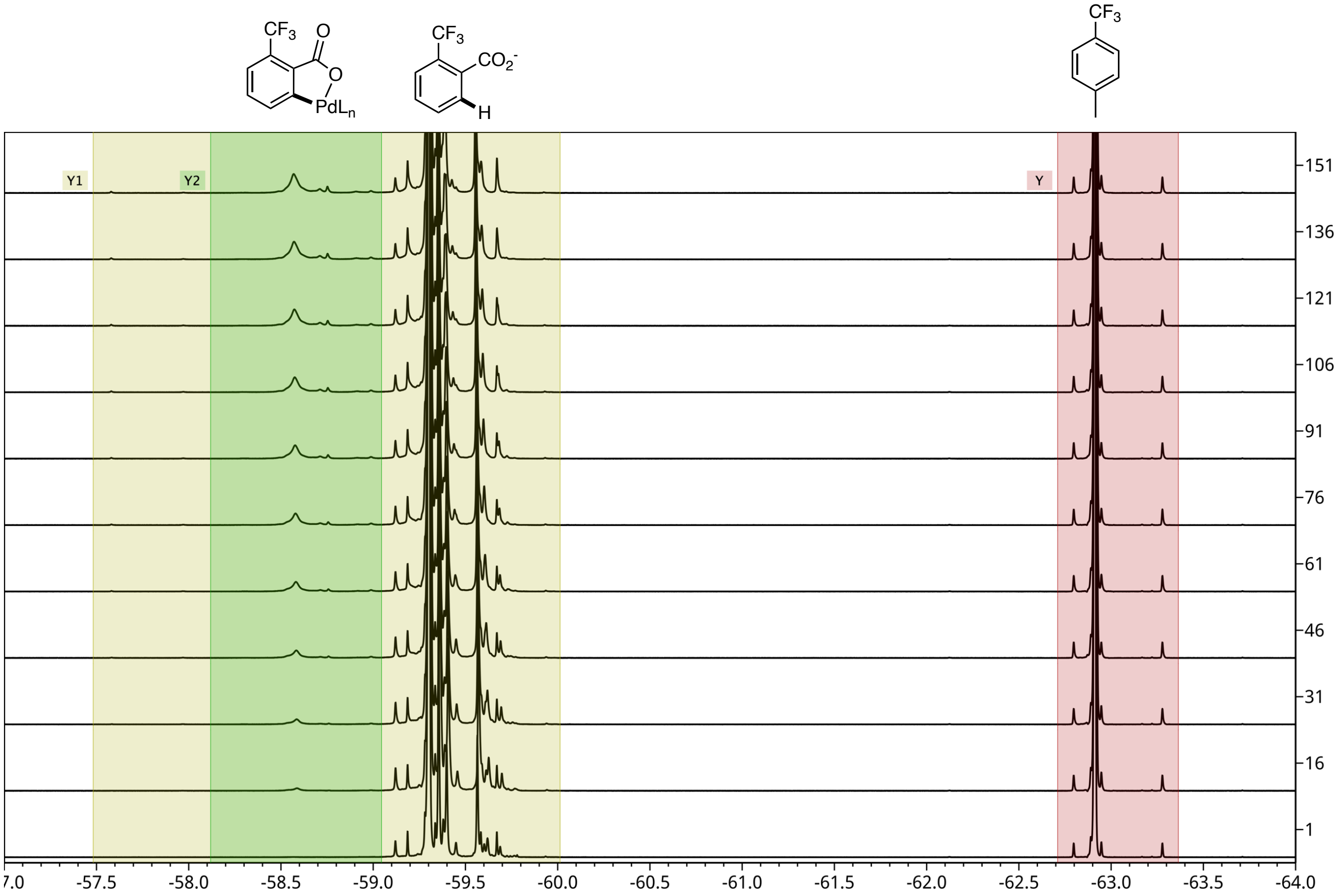

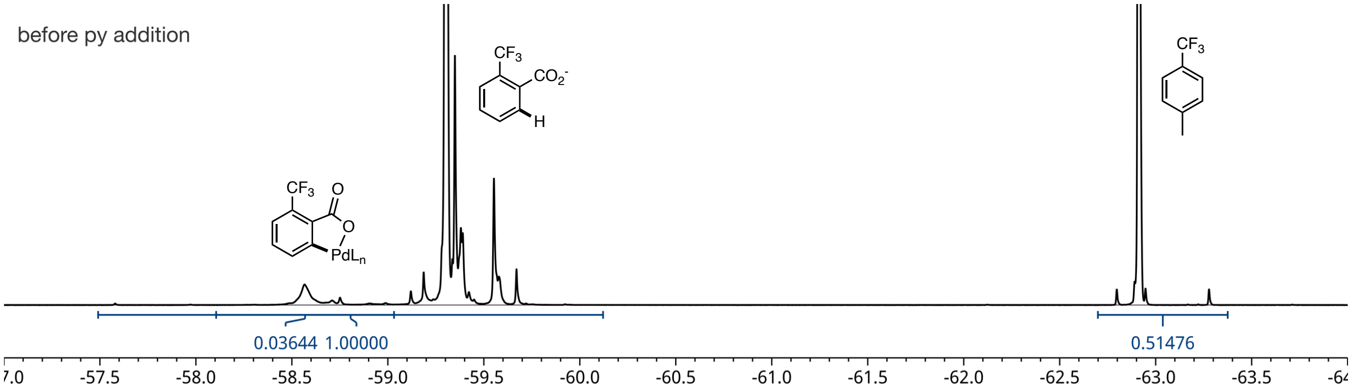

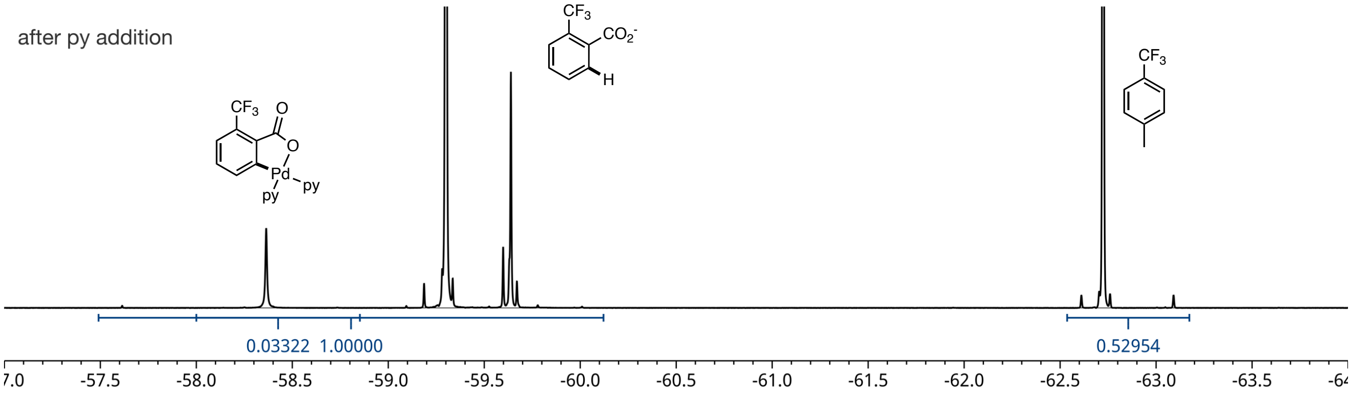

Substrate **2a** (potassium 4-fluoro-2-trifluoromethylbenzoate), Ac-*α*-*L*-Val-OH, 40 °C, ^{19}F NMR (^{t}AmylOH, 564.3 MHz):

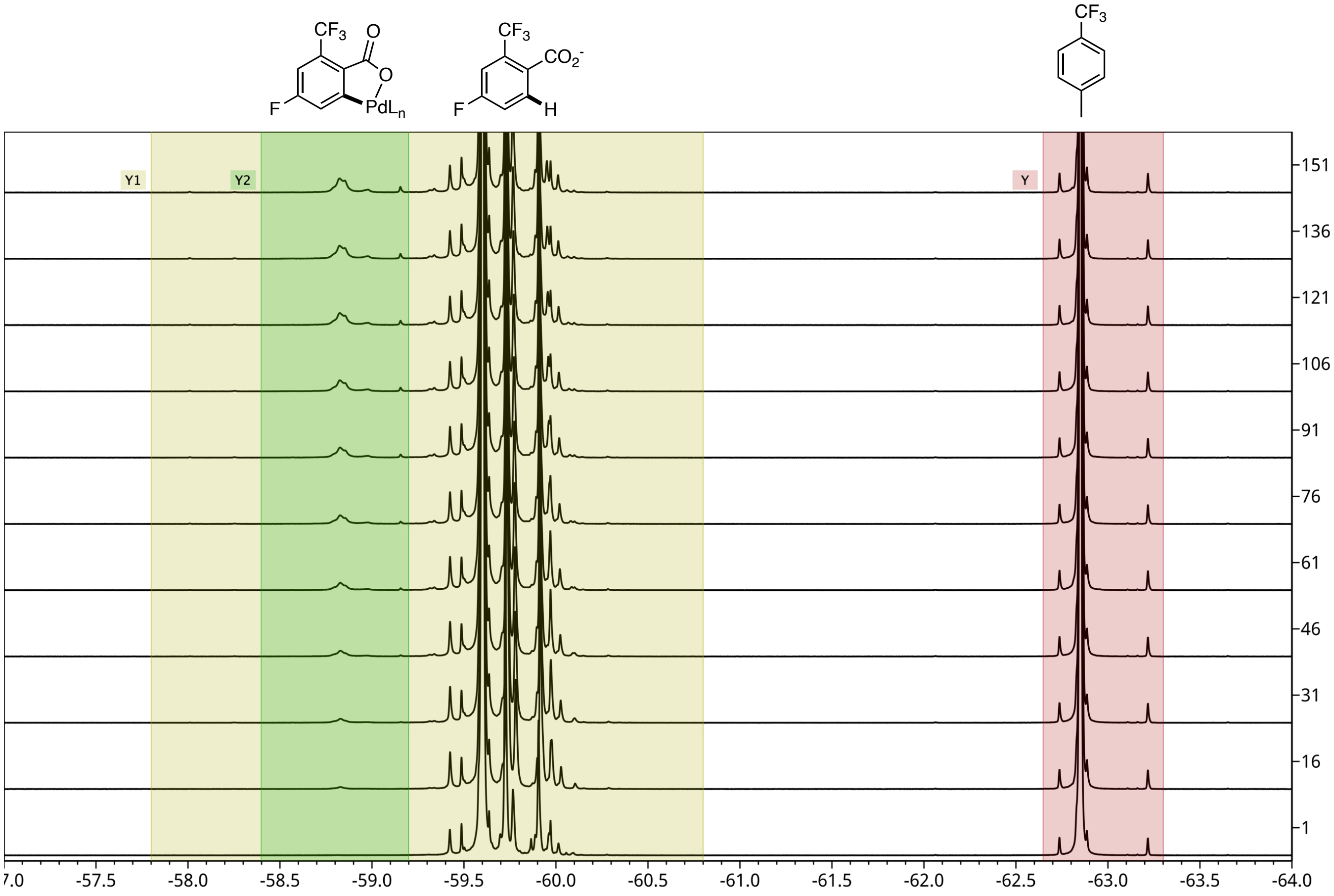

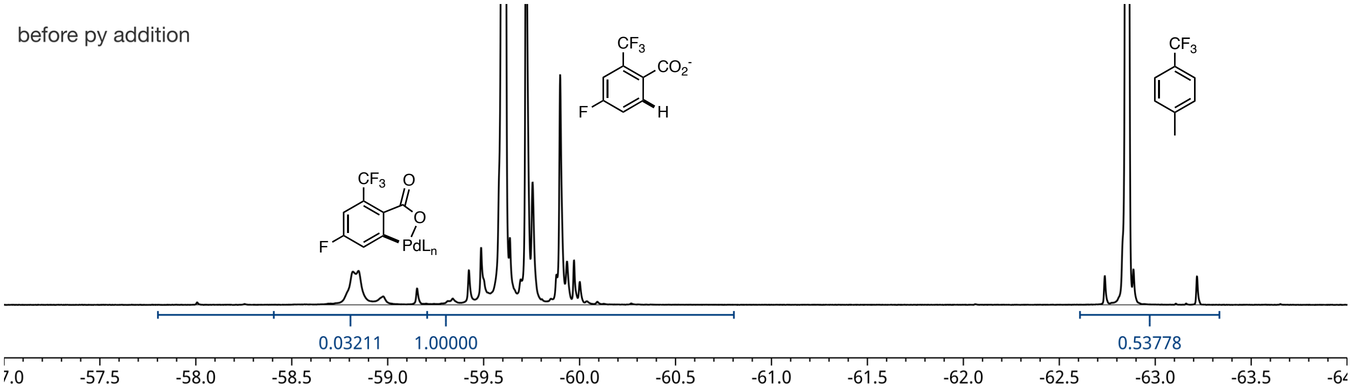

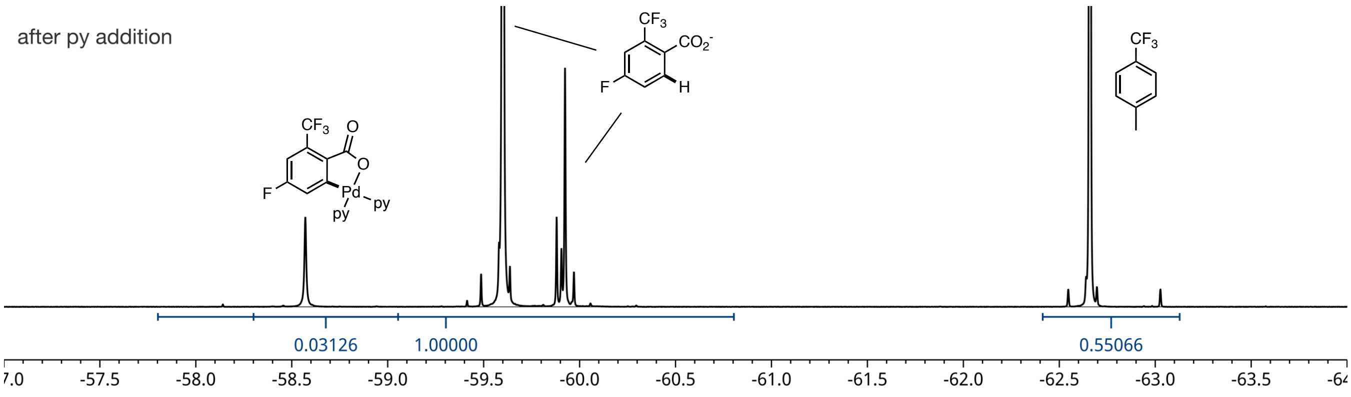

Substrate **3a** (potassium 4-fluoro-2-methylbenzoate), Ac-*α*-*L*-Val-OH, 40 °C, ^{19}F NMR (^{t}AmylOH, 564.3 MHz):

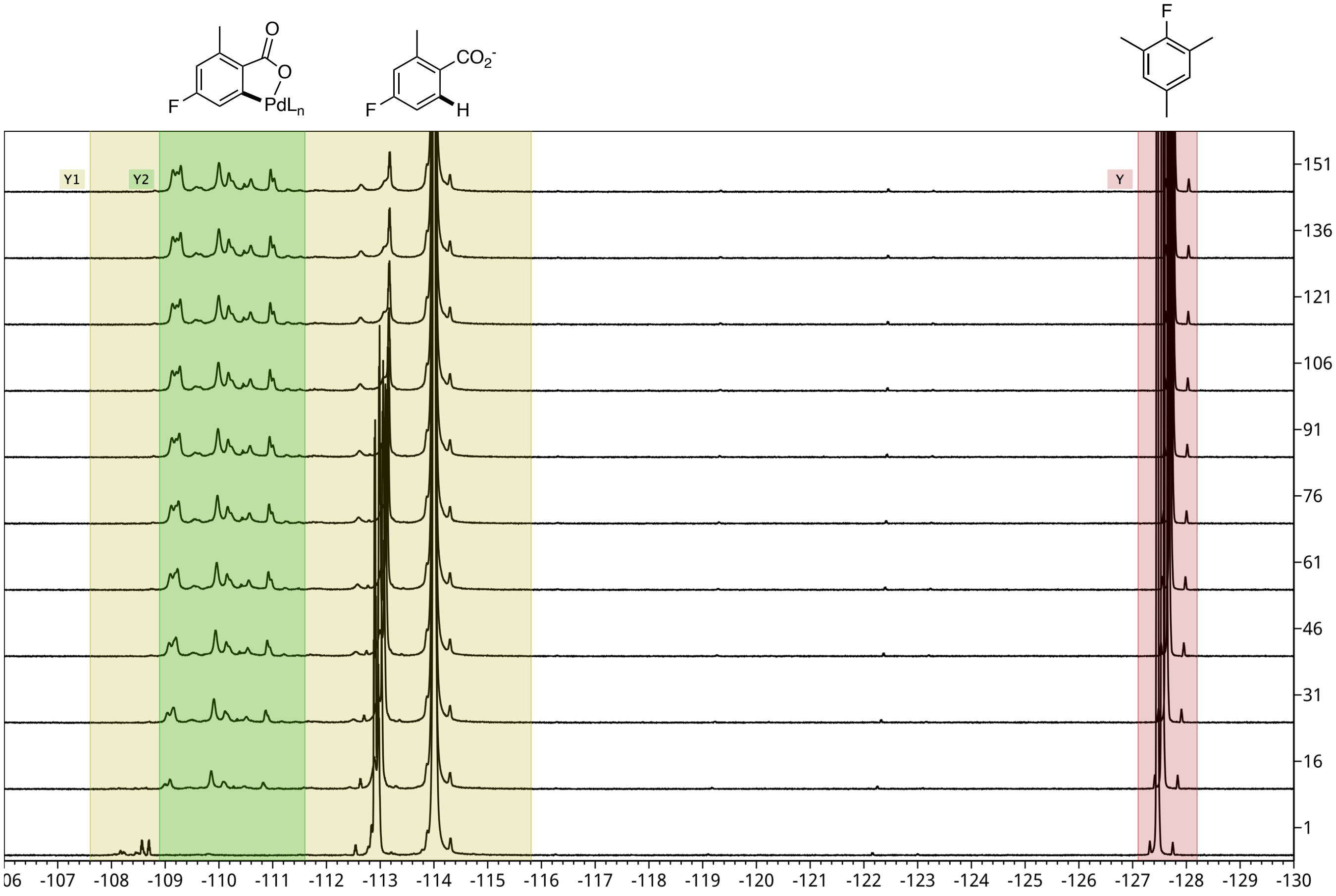

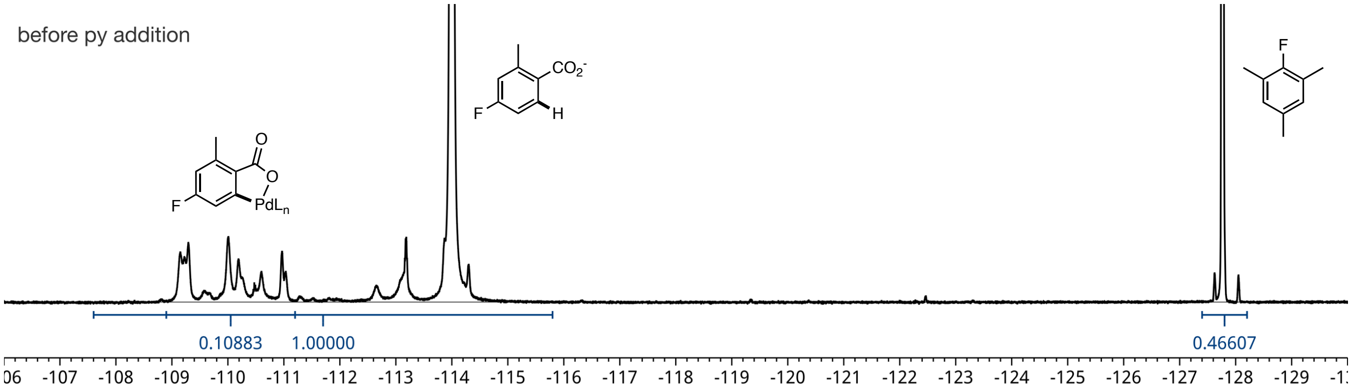

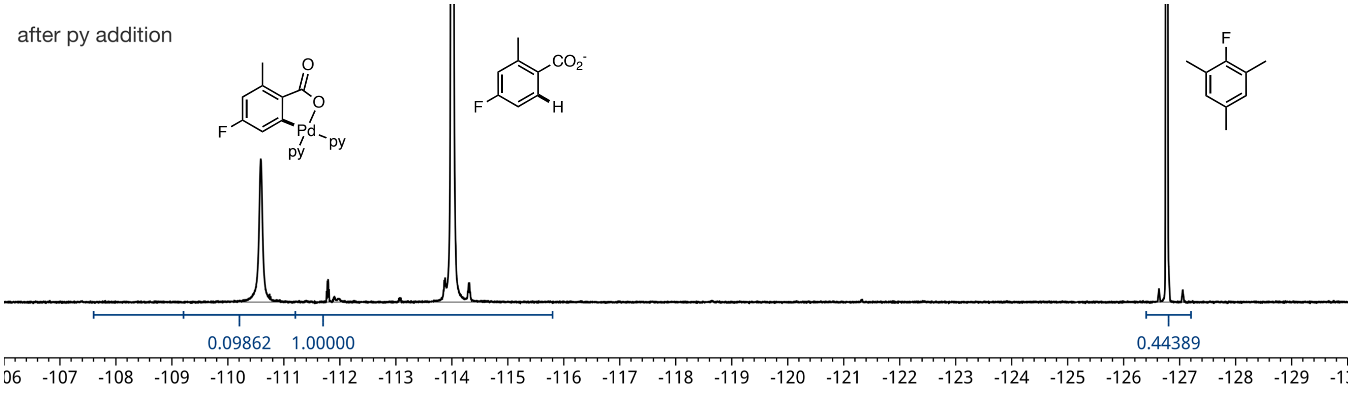

Substrate **4a** (potassium 4-fluoro-2-isopropylbenzoate), Ac-*α*-*L*-Val-OH, 40 °C, ^{19}F NMR (^{t}AmylOH, 564.3 MHz):

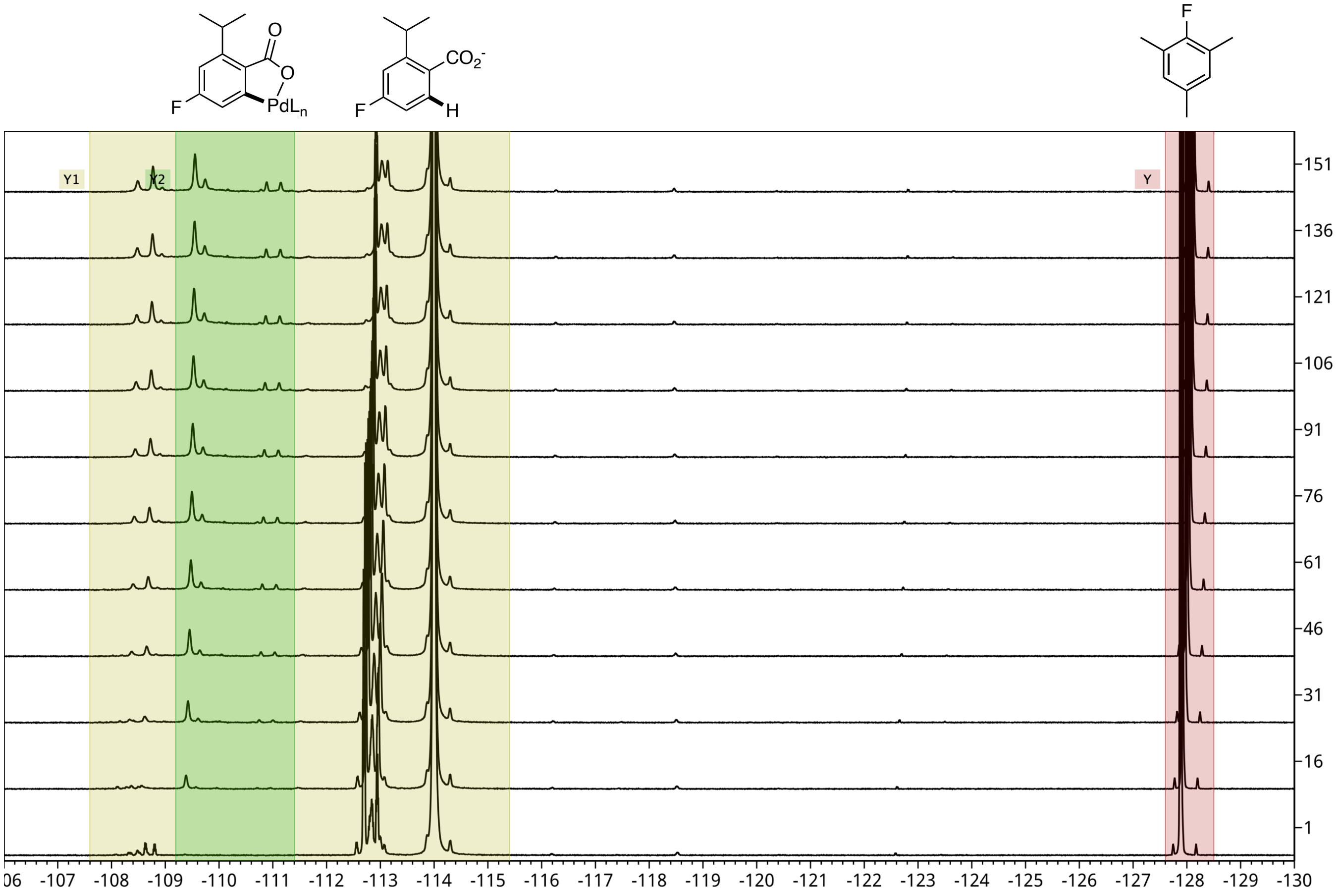

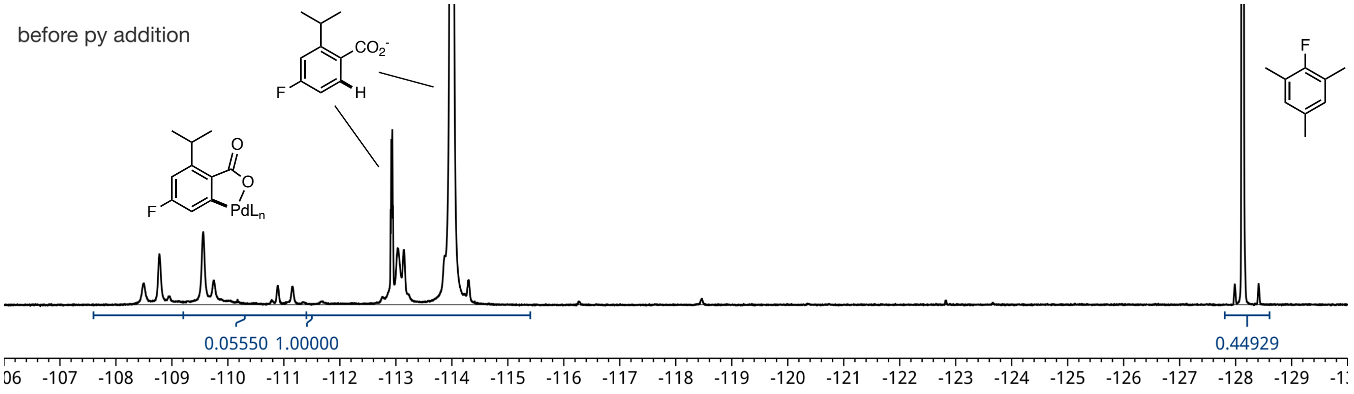

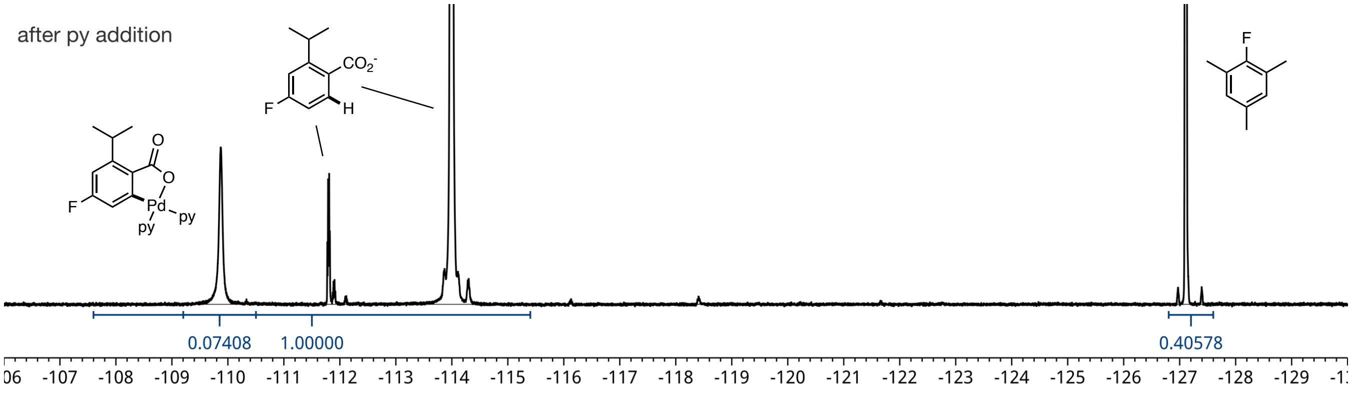

Substrate **1b** (potassium 2-(trifluoromethyl)phenylacetate), Ac-*α*-*L*-Val-OH, 40 °C, ^{19}F NMR ([t]AmylOH, 564.3 MHz):

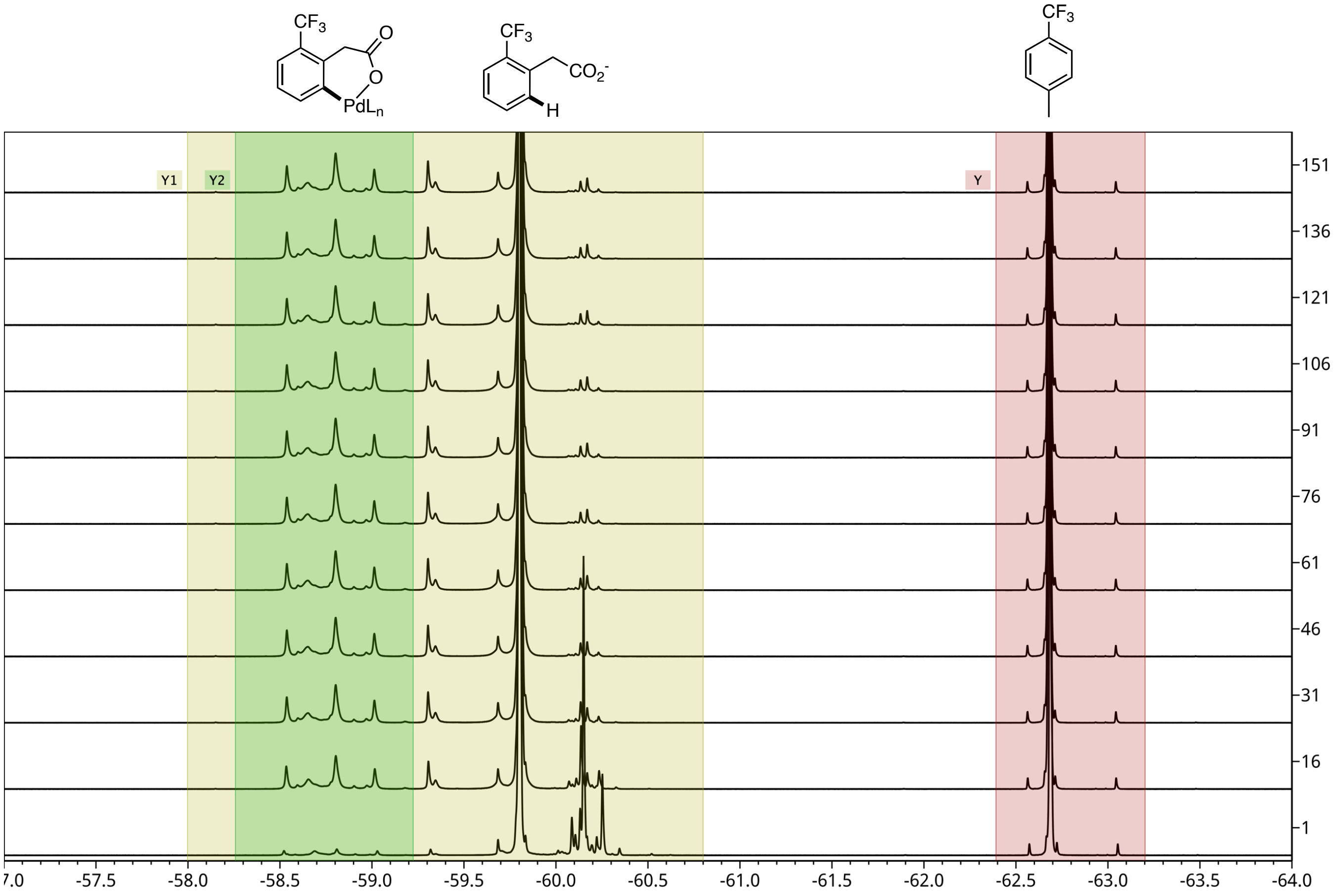

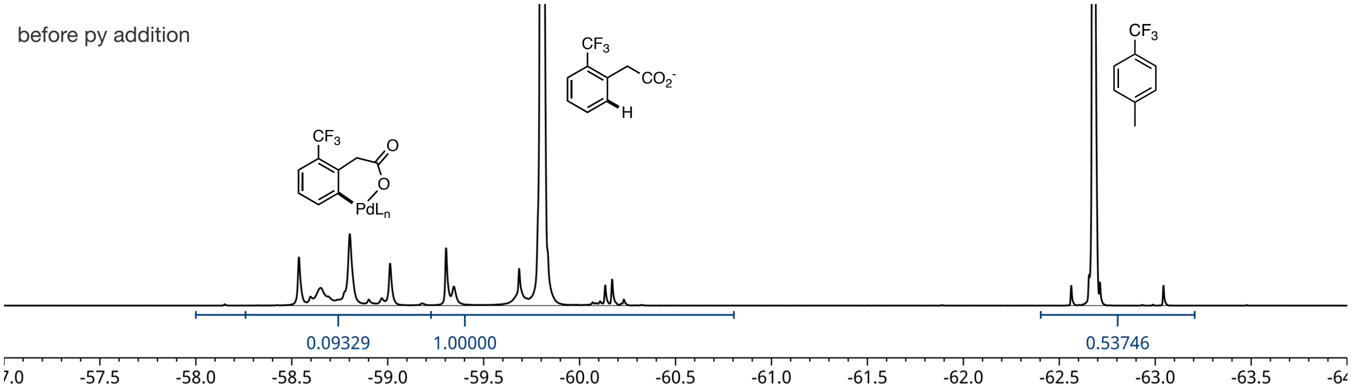

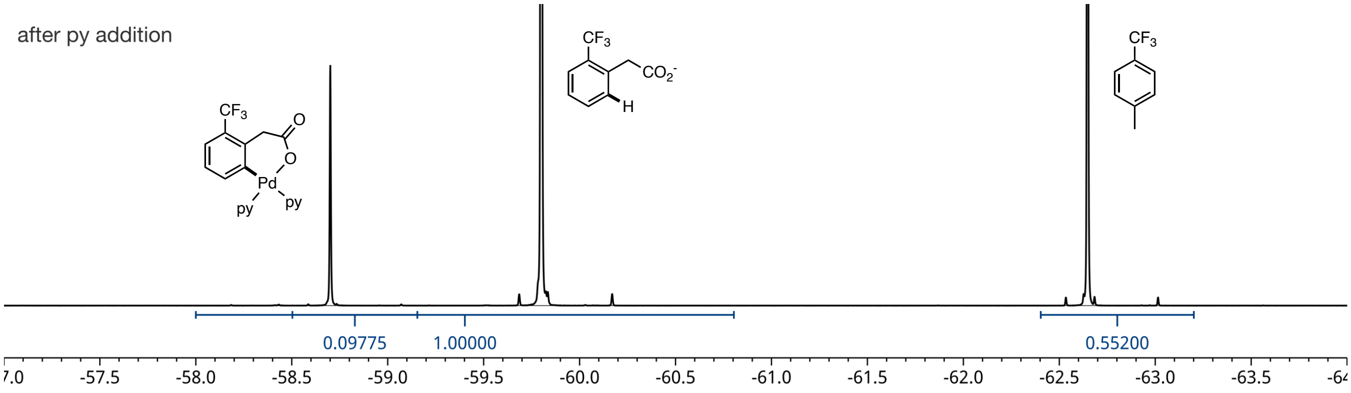

Substrate **2b** (potassium 4-fluoro-2-(trifluoromethyl)phenylacetate), Ac-α-*L*-Val-OH, 40 °C, ^{19}F NMR (^{t}AmylOH, 564.3 MHz):

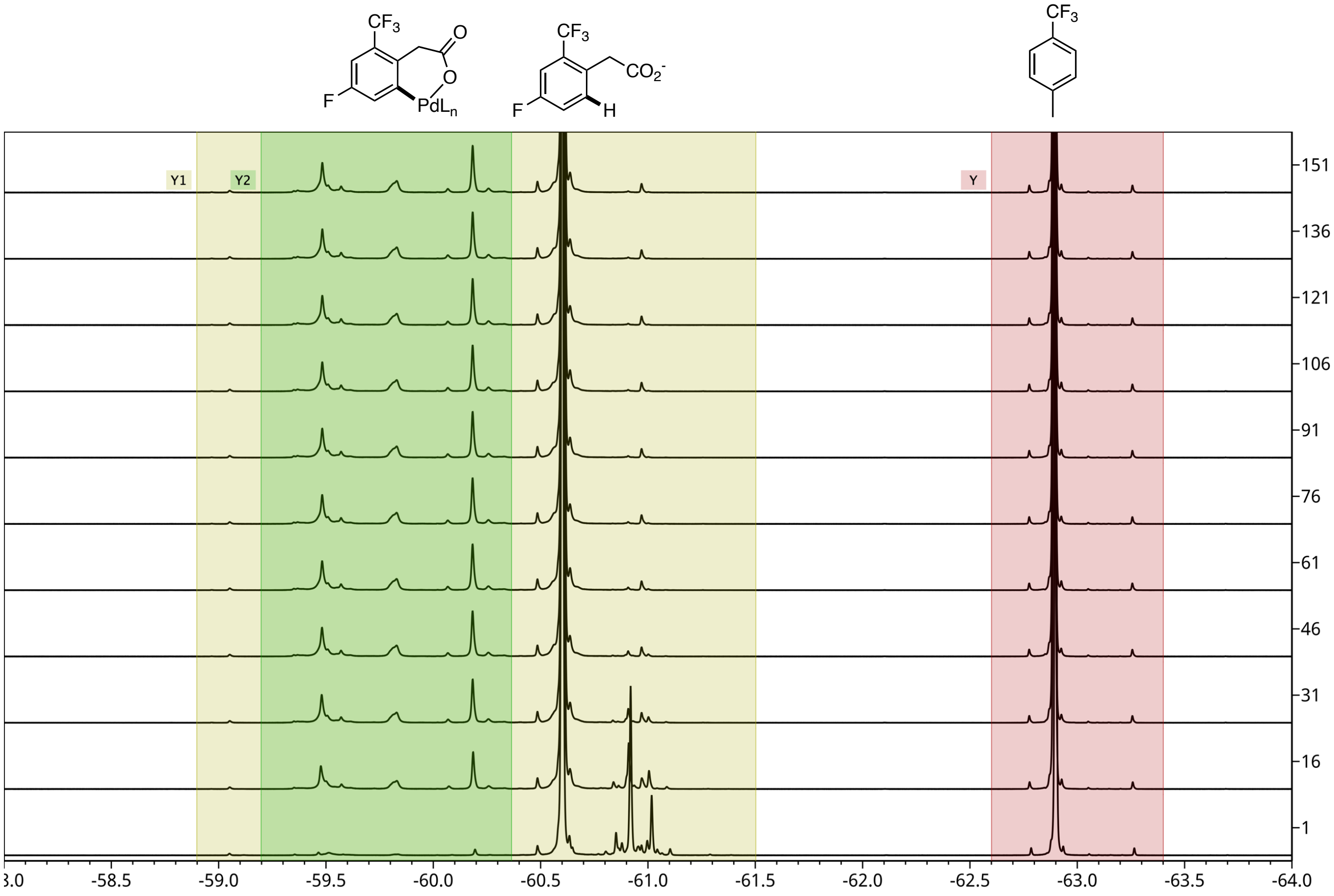

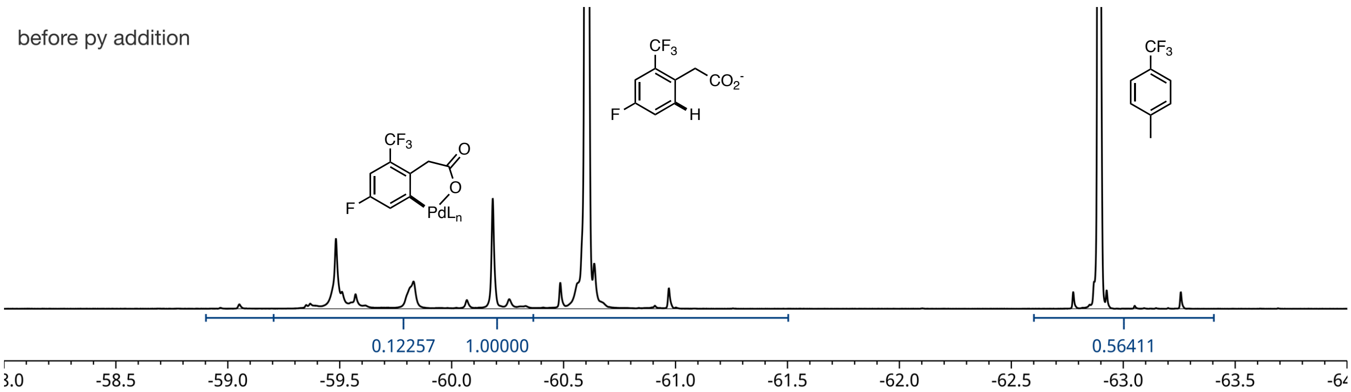

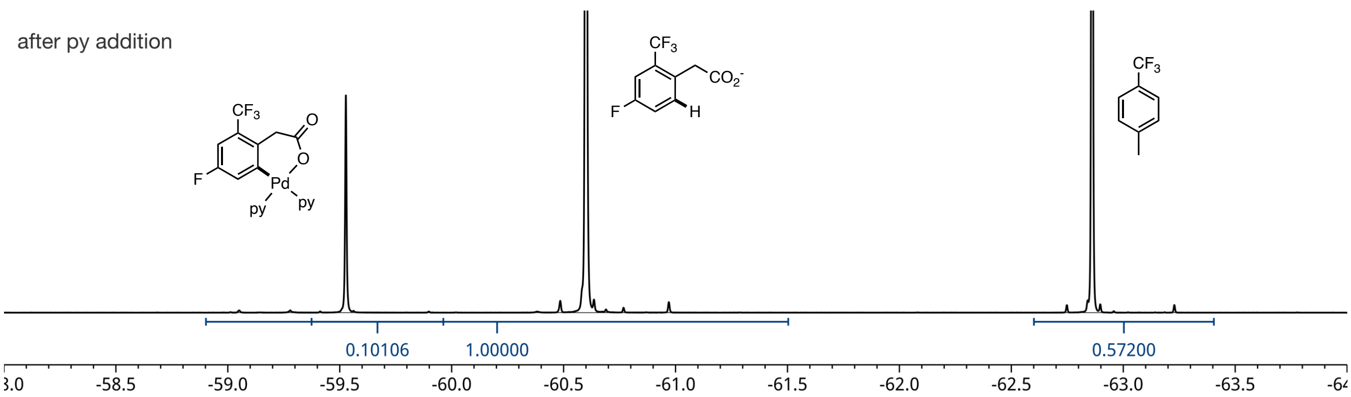

Substrate **3b** (potassium 4-fluoro-2-methylphenylacetate), Ac-*α*-*L*-Val-OH, 40 °C, ^{19}F NMR (^{t}AmylOH, 564.3 MHz):

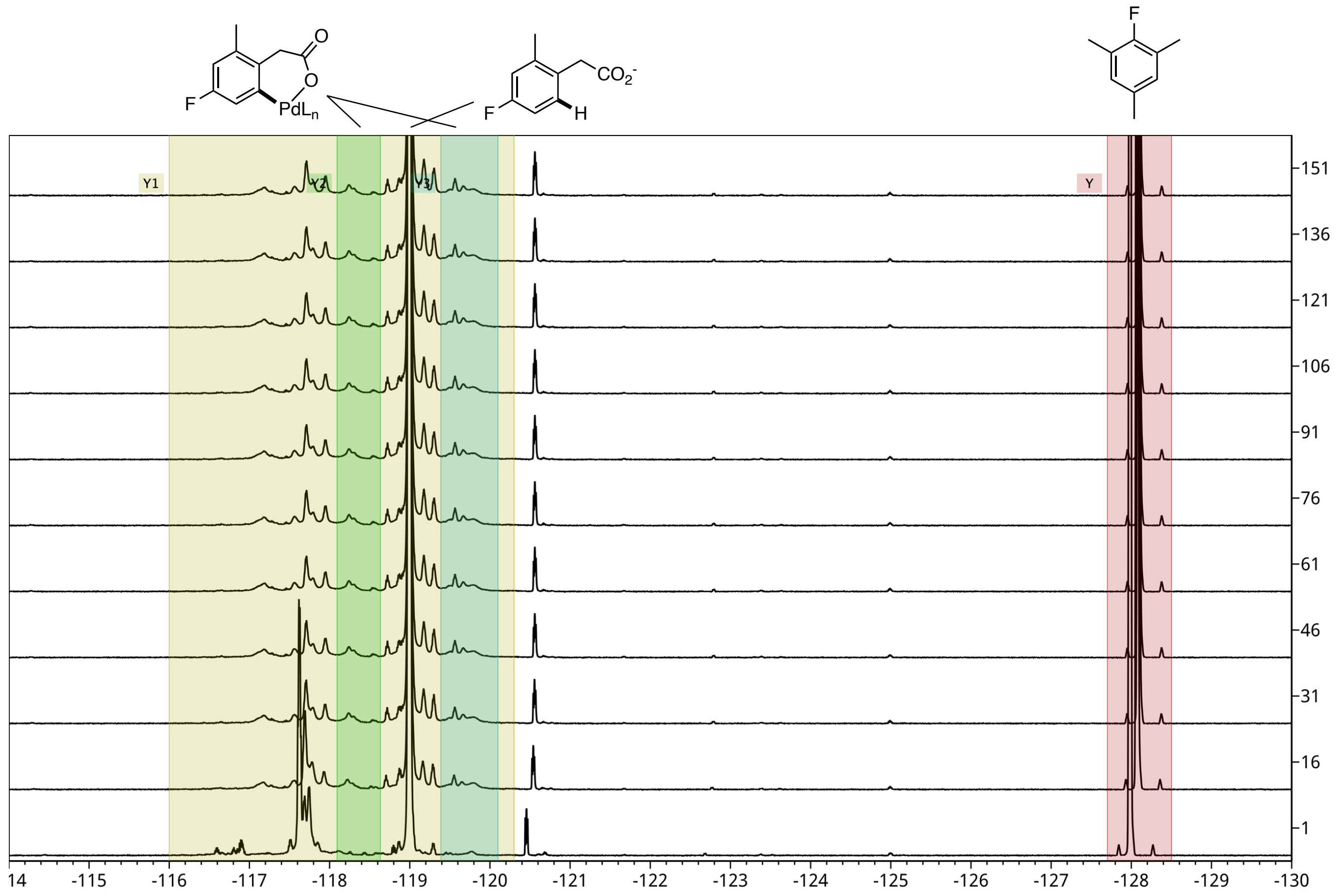

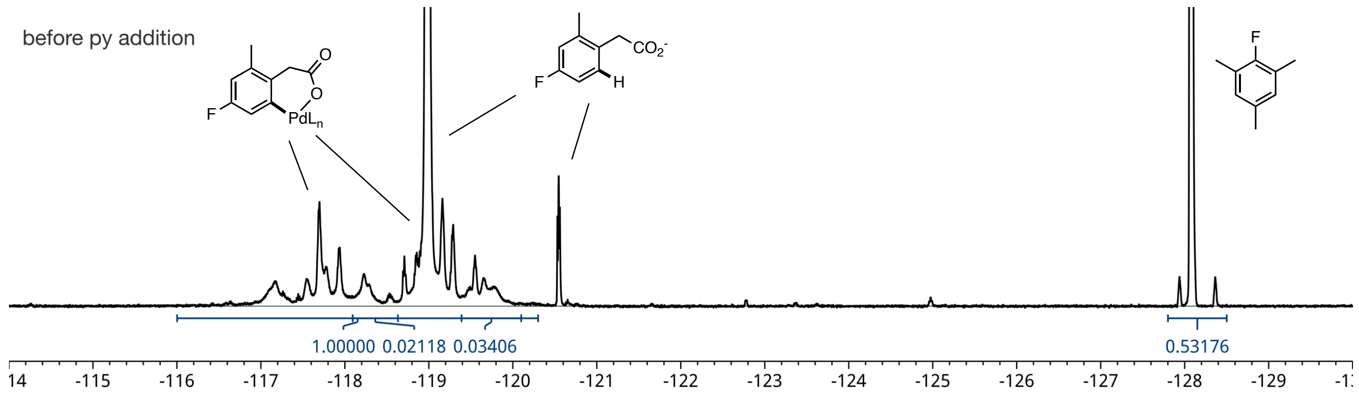

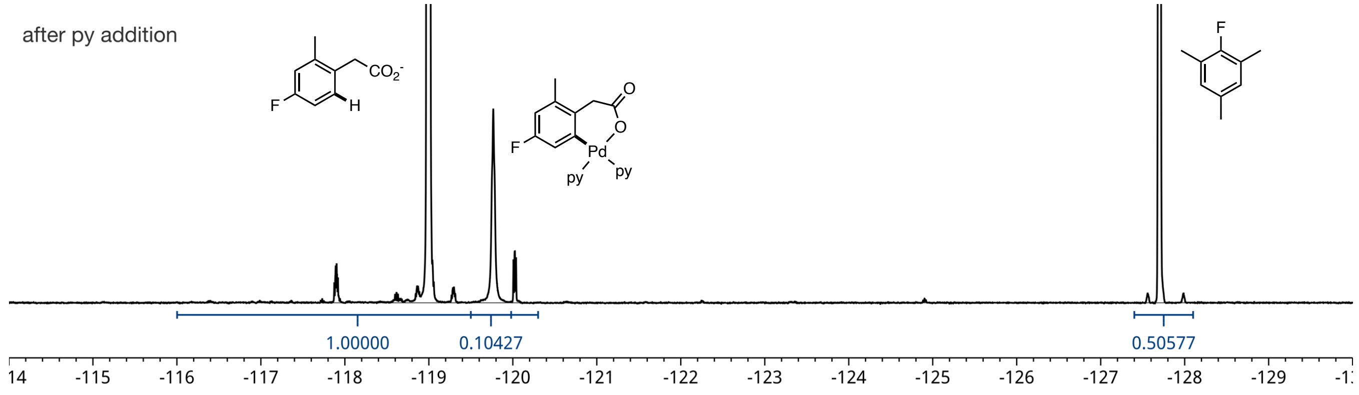

Substrate **1a** (potassium 2-trifluoromethylbenzoate), Ac-β^3-*L*-Val-OH, 40 °C, ^{19}F NMR (^{t}AmylOH, 564.3 MHz):

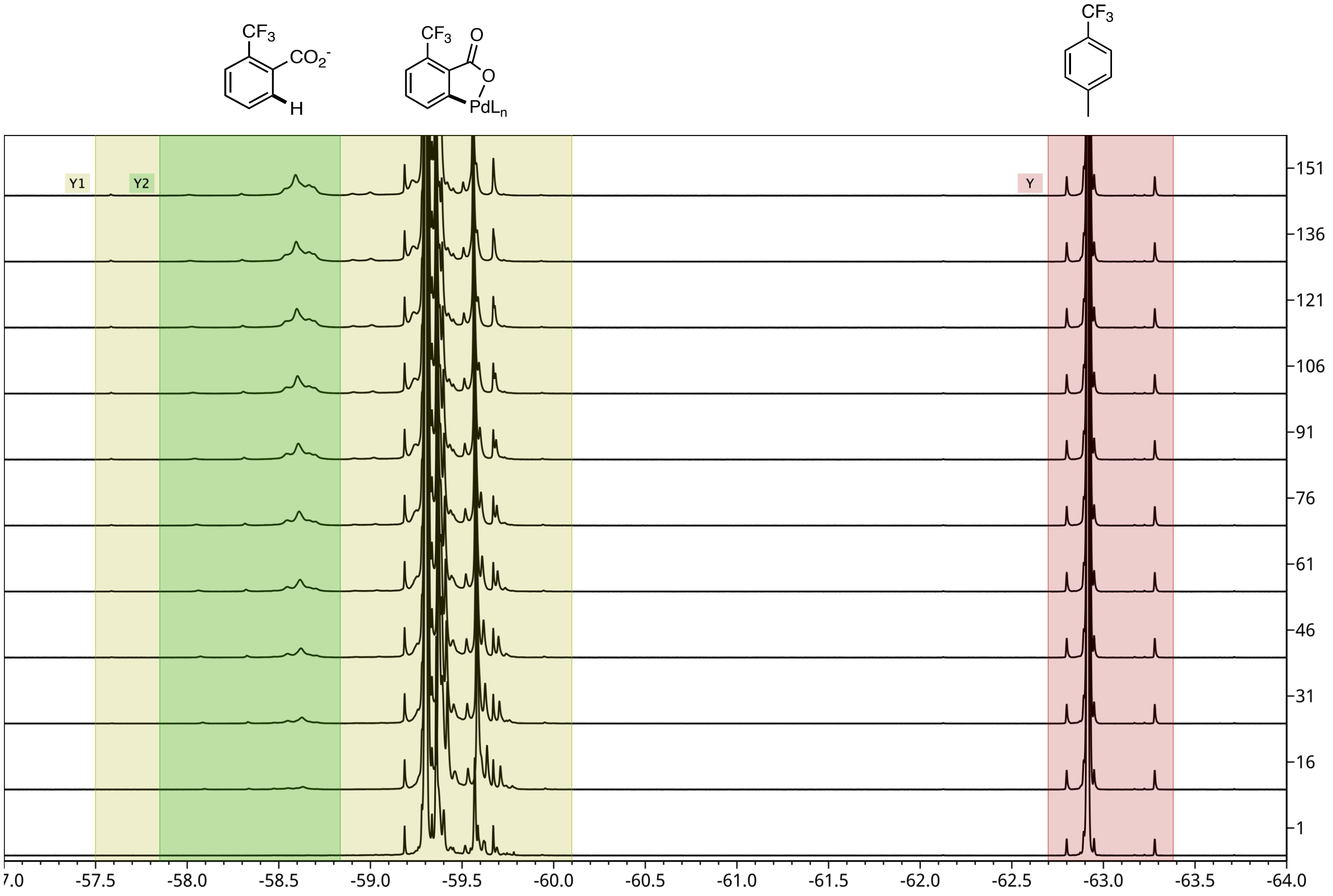

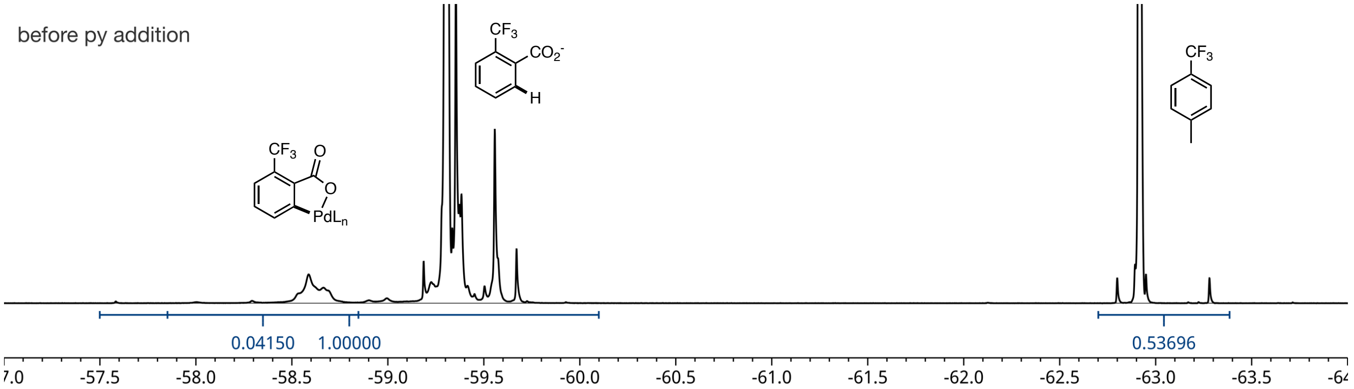

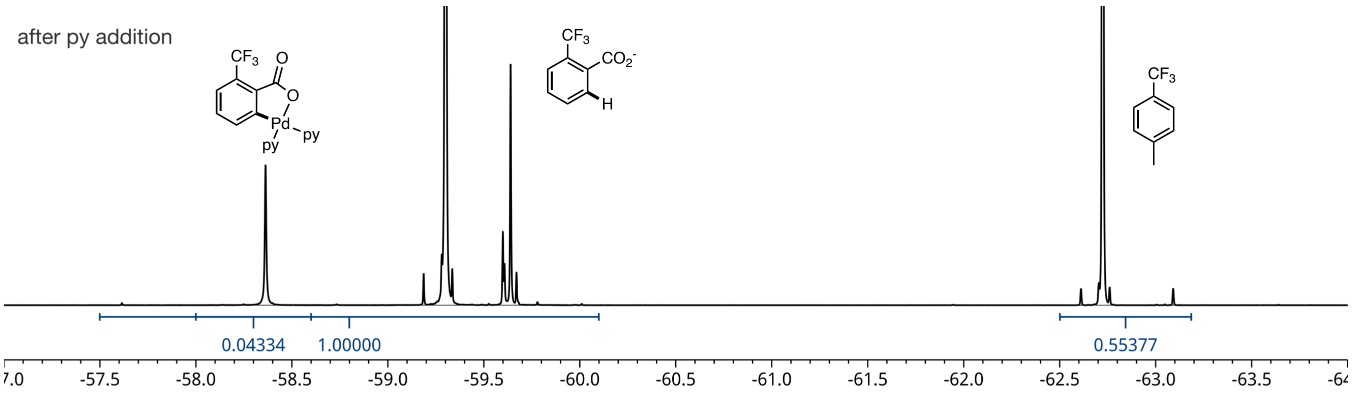

Substrate **1b** (potassium 2-(trifluoromethyl)phenylacetate), Ac-β^3-*L*-Val-OH, 40 °C, ^{19}F NMR (^{t}AmylOH, 564.3 MHz):

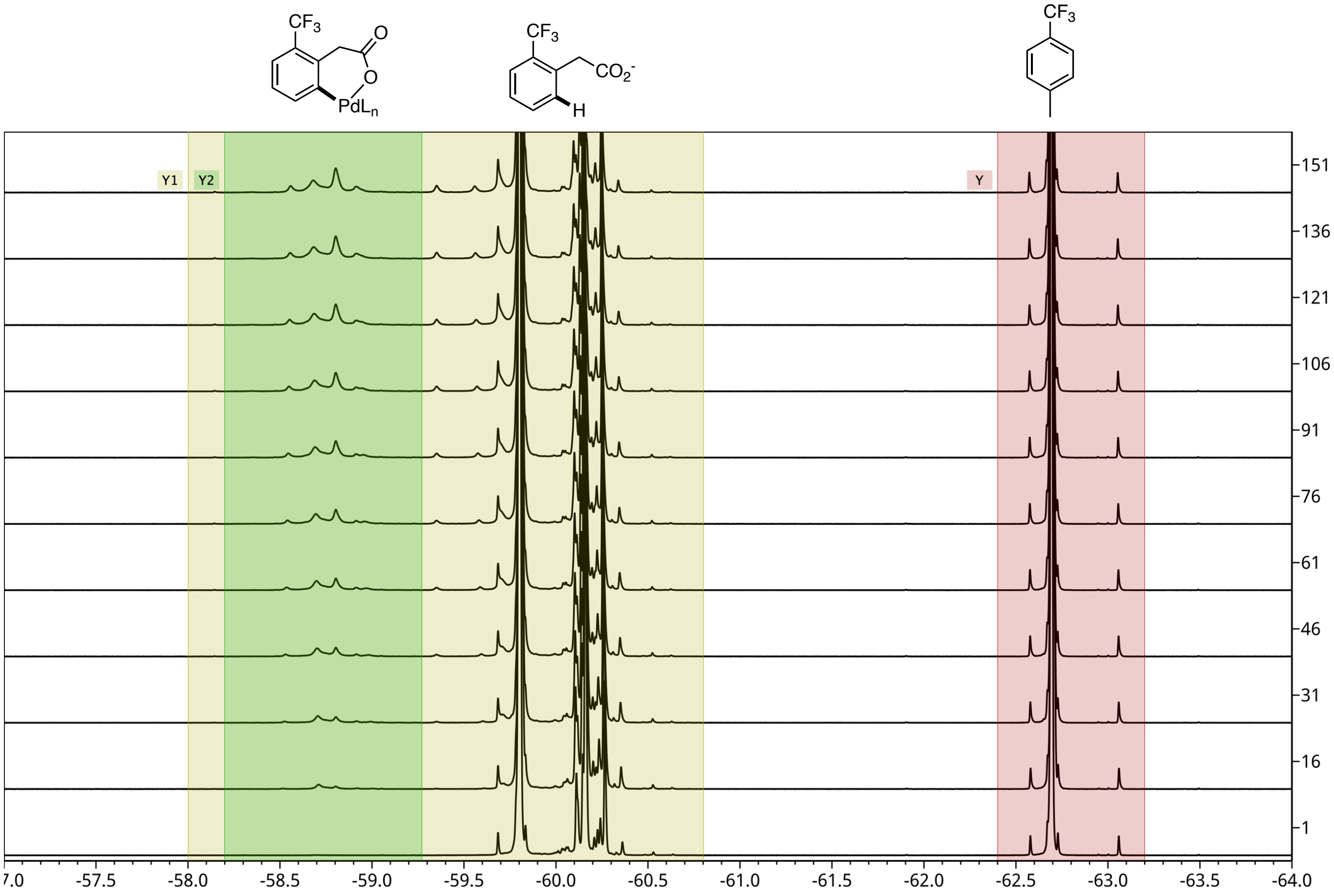

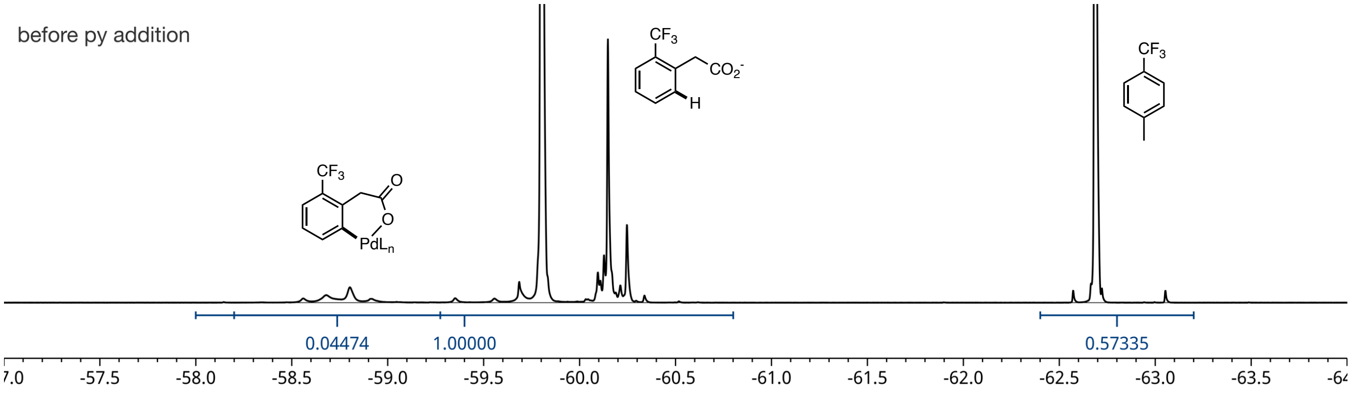

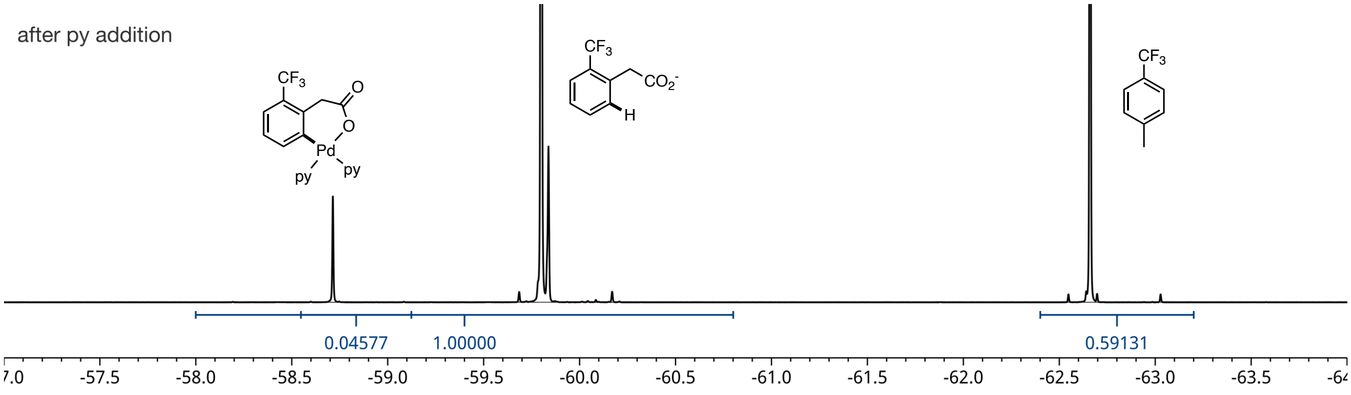

4. *In situ* NMR observation of C–H activation equilibrium

4a. General experimental procedures

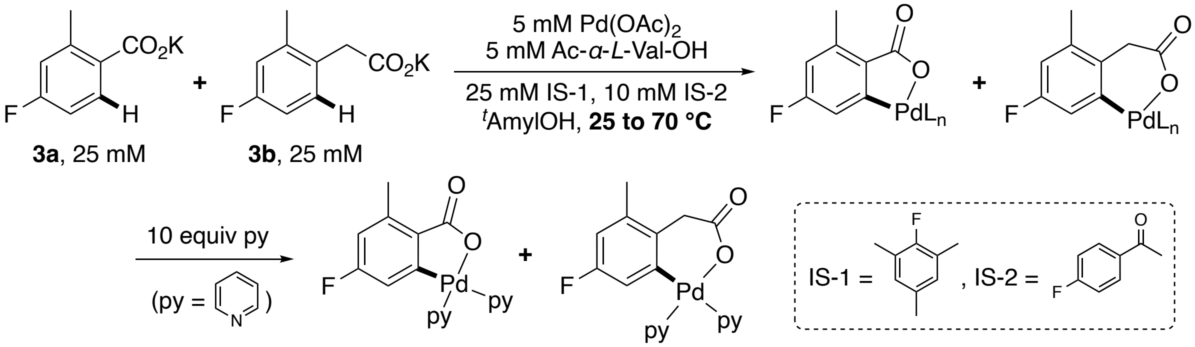

These data were recorded on a Bruker Avance III 600 MHz spectrometer with a TCI-F cryoprobe. Three stock solutions A, B, and C were prepared in volumetric flasks. For stock solutions A and B, sonication in a warm water bath was required to dissolve the solids. A screw cap NMR tube* was charged with 500 μL of stock solution A containing both potassium salt substrates **3a** and **3b** (18.8 μmol, 0.5 equiv each), *N*-acetyl-*L*-valine (3.8 μmol, 0.1 equiv), internal standard-1 2,4,6-trimethylfluorobenzene (18.8 μmol, 0.5 equiv) and internal standard-2 4-fluoroacetophenone (7.5 μmol, 0.2 equiv)†. The spectrometer was set to 25 °C, allowed to stabilize for 1 h, and calibrated to ± 0.1 °C using 4 % methanol in MeOD-*d*4.[1] The NMR tube was inserted into the spectrometer. No lock was used, and shimming was performed using the *t*AmylOH solvent peaks by tuning the probe to ^{1}H. The probe was then tuned to ^{19}F, and consecutive time points were recorded using the *multizg* command in Topshim. The time when the first spectrum was recorded was defined as the starting point. At ~ 0.5 h, the NMR tube was removed from the spectrometer, and 250 μL of stock solution B containing palladium acetate (3.8 μmol, 0.1 equiv) in *t*AmylOH was injected into the NMR tube, the time of injection was recorded. The sample was shimmed, and consecutive time points were recorded, using the same procedure as above. At ~ 1.2 h, the spectrometer was set to 70 °C, and allowed to heat up to the desired temperature over ~ 20 min. At ~ 5.3 h, an additional time point ("3a before py addition") was collected, the NMR tube was then removed from the spectrometer. 500 μL of stock solution C containing pyridine (375 μmol, 10 equiv) in acetone was added, the NMR tube was shaken vigorously to allow the liquid to mix. The reaction mixture changed from yellow to almost colorless. The NMR tube was returned to the spectrometer and a final spectrum ("3a after py addition") was collected. The data set was imported to MestReNova, the chemical shifts were referenced to the substrate **3b** peak. The concentration of the palladacycle products were determined using the same procedure as previously described in **Section 3a**‡.

* Norell® item# S-5-600-SC-7.

† Two internal standards were used for the purpose of aiding in phase correction of the ^{19}F NMR spectra. The substrate and palladacycle region appear as an ensemble of broad peaks, making phase correction difficult. Manual adjustments to each spectrum were made using the sharp peaks of both internal standards as reference, until both internal standard peaks have correct phases. Only internal standard-1 was used for quantification.

‡ The reaction mixture contains very small amounts of 6-membered palladacycles at ~ 5 h. For quantification of the 6-membered palladacycle, an additional set of "py addition" spectra need to be acquired prior to raising the temperature. Therefore, the experiment was repeated using the same procedure until ~ 1 h. An additional time point ("3b before py addition") was collected. Stock solution C containing pyridine was added, and a final spectrum ("3b after py addition") was collected, following the above procedure.

4b. NMR spectra of C–H activation equilibrium observation

Substrate **3a** (potassium 4-fluoro-2-methylbenzoate) and **3b** (potassium 4-fluoro-2-methylphenylacetate), Ac-*α*-*L*-Val-OH, 40 °C, ^{19}F NMR (^{t}AmylOH, 564.3 MHz):

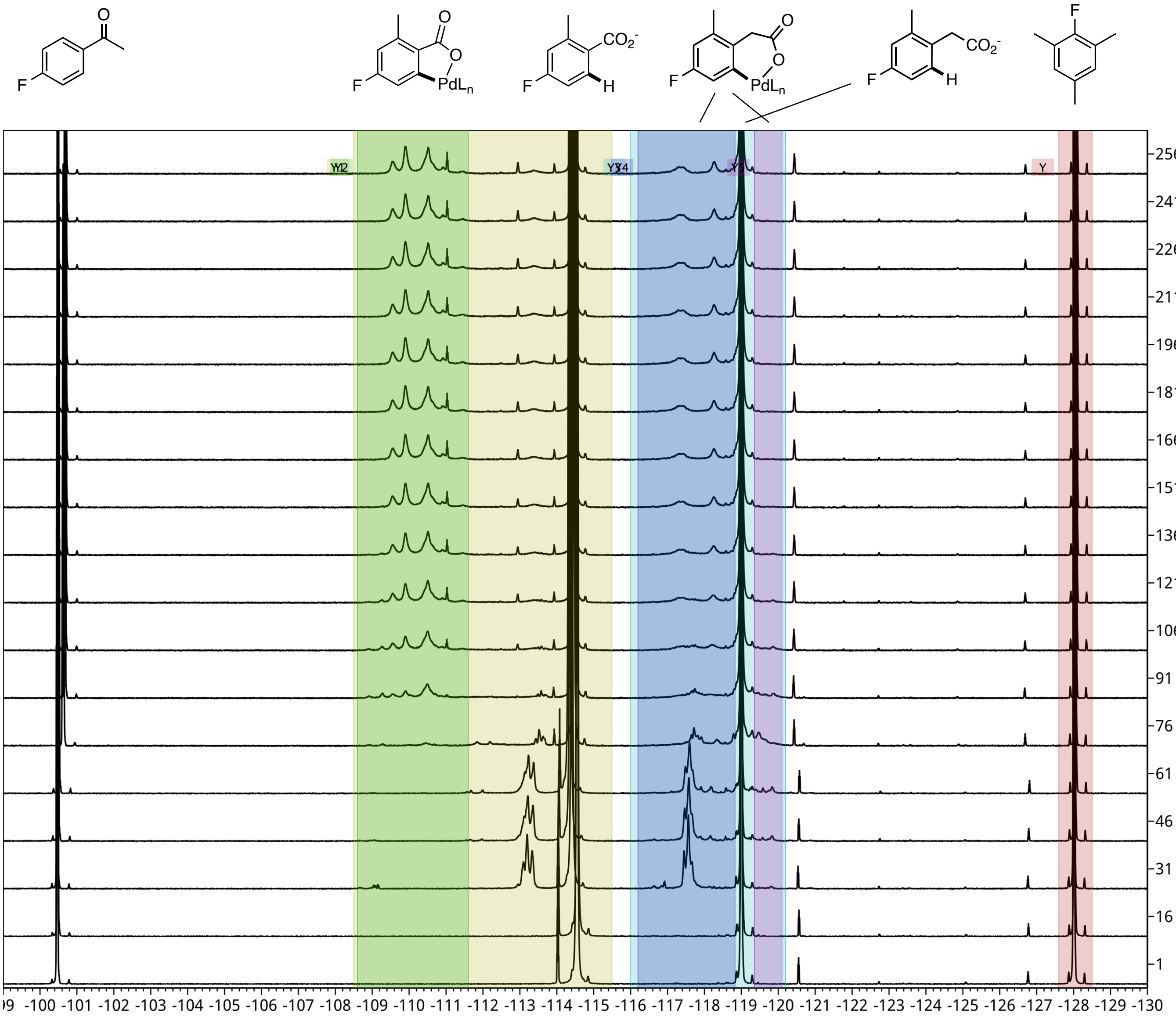

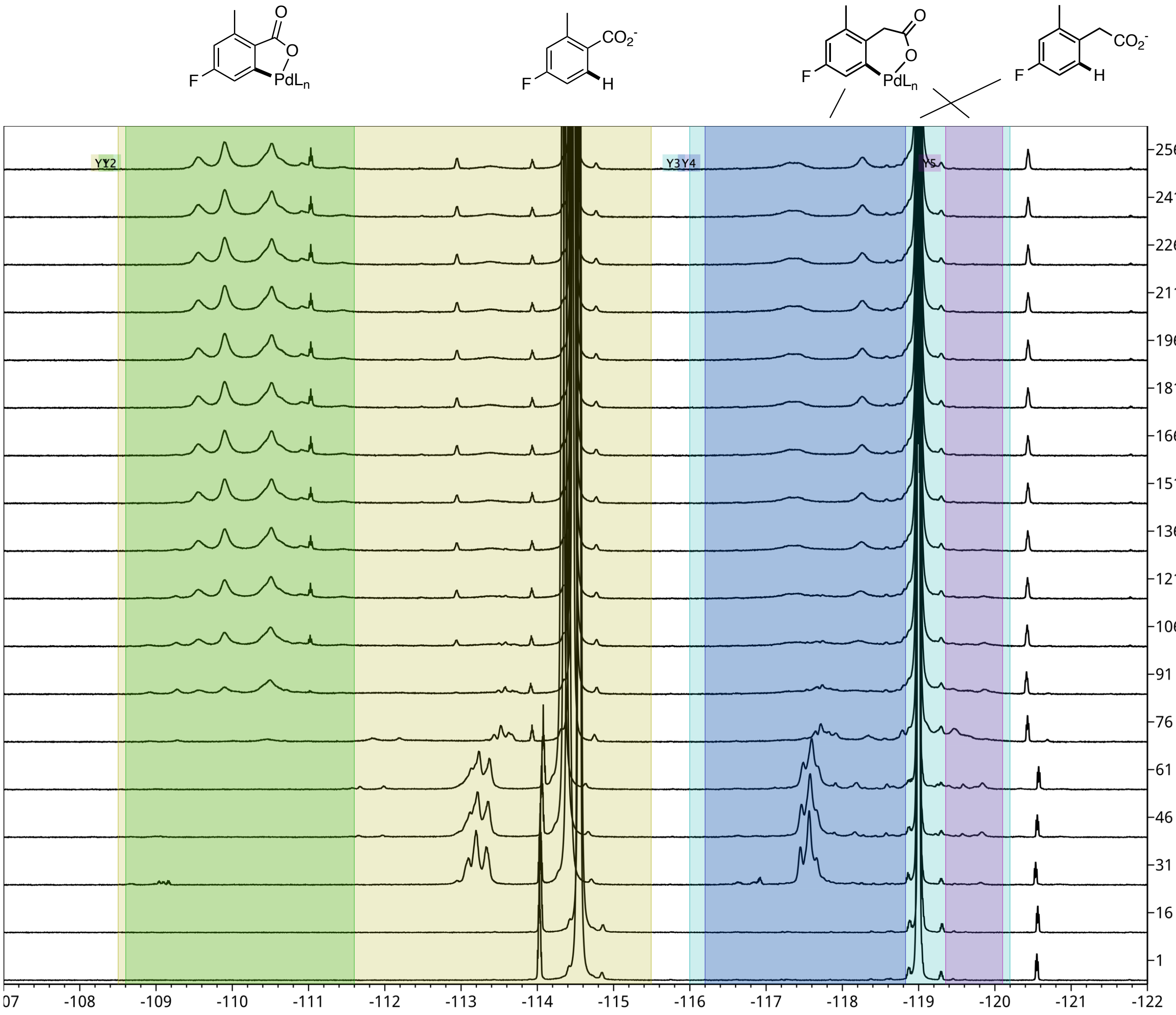

F
PdLn
CO2-
F
H
F
PdLn
CO2-
F
H
Y1
Y2
Y3
Y4
Y5
256
241
226
211
196
181
166
151
136
121
106
91
76
61
46
31
16
1
-108
-109
-110
-111
-112
-113
-114
-115
-116
-117
-118
-119
-120
-121
-122

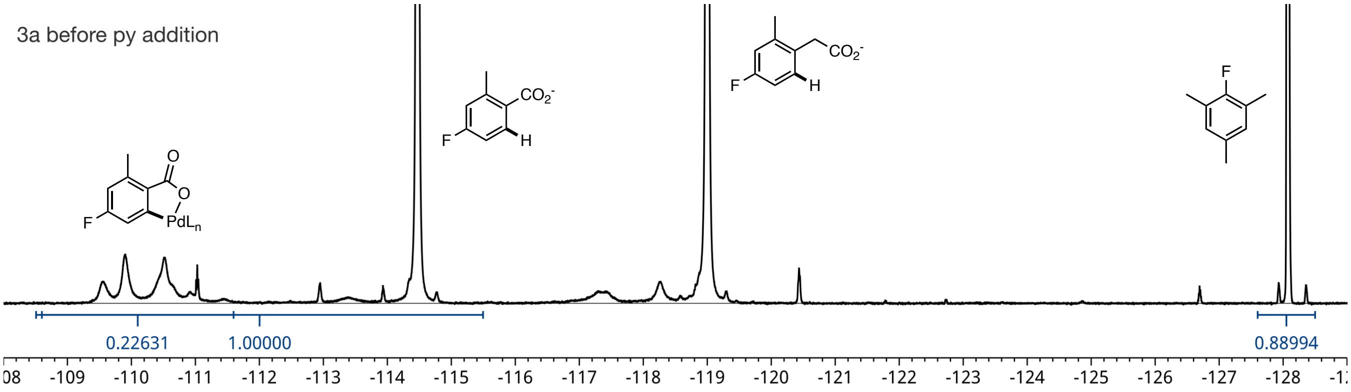
3a before py addition
CO2-
F
H
O
O
PdLn
0.22631
1.00000
0.88994
-109 -110 -111 -112 -113 -114 -115 -116 -117 -118 -119 -120 -121 -122 -123 -124 -125 -126 -127 -128

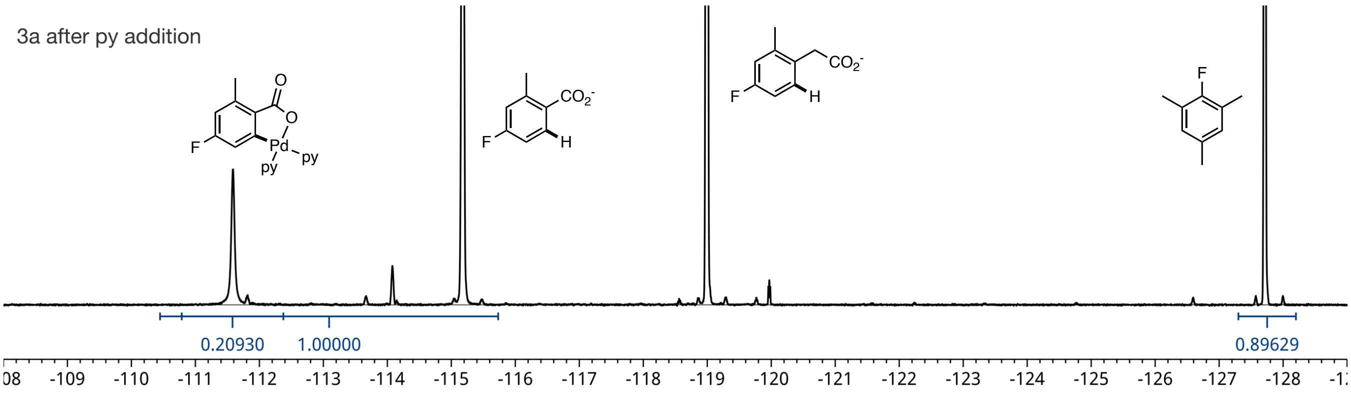
3a after py addition
O
O
F
Pd
py
py
CO2-
H
0.20930
1.00000
0.89629
-109 -110 -111 -112 -113 -114 -115 -116 -117 -118 -119 -120 -121 -122 -123 -124 -125 -126 -127 -128

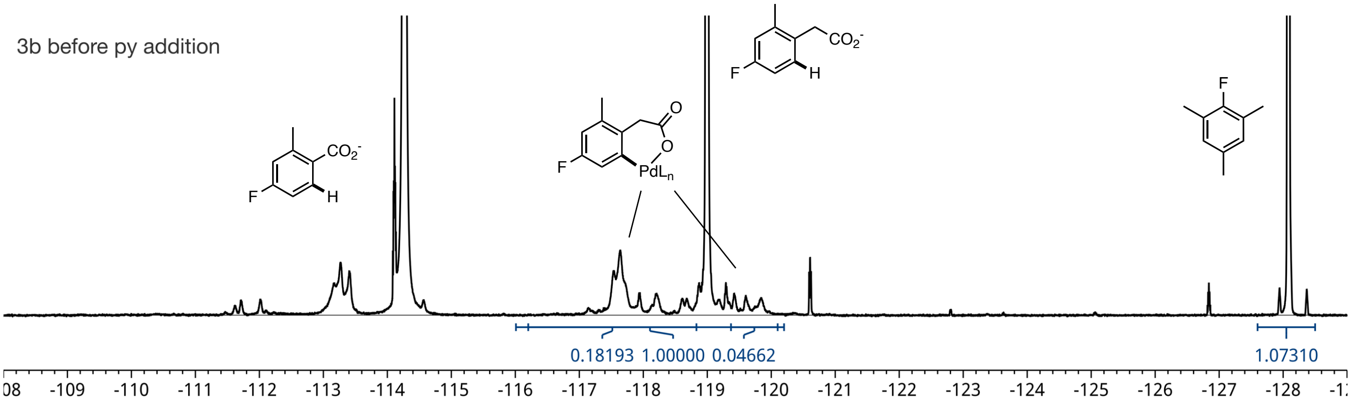
3b before py addition
CO2-
F
H
O
O
PdLn
0.18193
1.00000
0.04662
1.07310
-109 -110 -111 -112 -113 -114 -115 -116 -117 -118 -119 -120 -121 -122 -123 -124 -125 -126 -127 -128

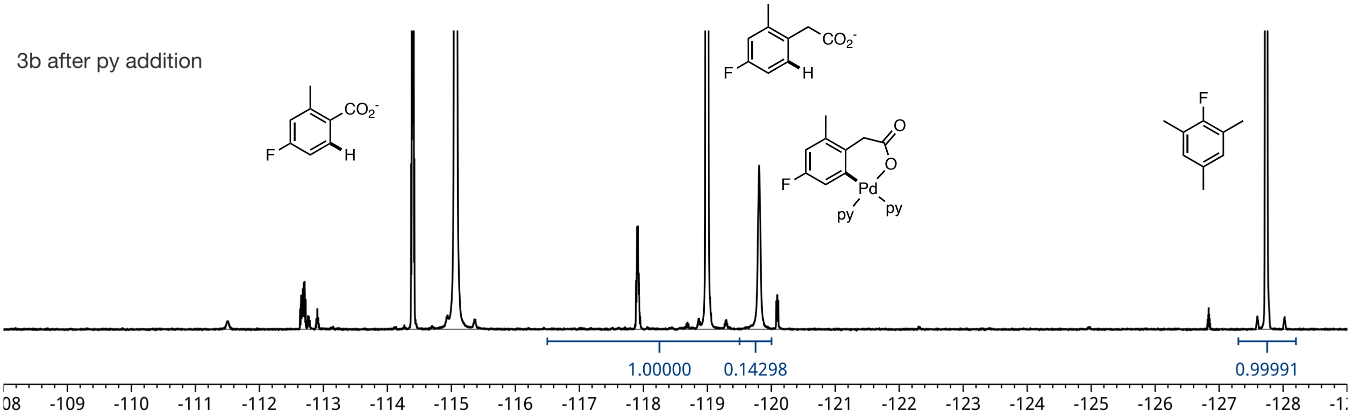
3b after py addition
CO2-
F
H
O
O
Pd
py
py
1.00000
0.14298
0.99991
-109 -110 -111 -112 -113 -114 -115 -116 -117 -118 -119 -120 -121 -122 -123 -124 -125 -126 -127 -128

5. C–H activation assay with various MPAA ligands

5a. General experimental procedures

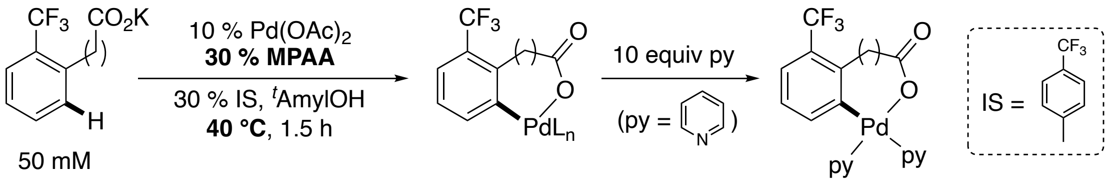

Reactions were carried out in 1 mL (8×30 mm) glass vials in an aluminum heating block, sealed by a thin sheet of Viton™ rubber clamped onto the top of the vials by an aluminum face plate bolted into the heating block. Three stock solutions A, B, and C were prepared in ^{t}AmylOH. Each tube was charged with 150 μL of stock solution A containing potassium salt substrate (**1a** or **1b**, 20.0 μmol, 1 equiv) and 4-methylbenzotrifluoride (6.0 μmol, 0.3 equiv, internal standard), 150 μL of stock solution B containing MPAA ligand (6.0 μmol, 0.3 equiv), and 100 μL of stock solution C containing palladium acetate (2.0 μmol, 0.1 equiv). Stock solutions A and C are prepared in volumetric flasks, and sonication in a warm water bath was required to dissolve the solids. A 1.9×4.8 mm stainless steel stir bar was added to each tube. The tubes were placed into the heating block, sealed with the rubber sheet, and stirred at 40 °C for 1.5 h. The tubes were cooled to room temperature, and each charged with 200 μL of stock solution D containing pyridine (200.0 μmol, 10 equiv) in acetone. 500 μL aliquots were taken from each tube and transferred to NMR tubes for ^{19}F NMR analysis. No lock was used, and shimming was performed using the ^{t}AmylOH solvent peaks by tuning the probe to ^{1}H. The product yields were determined by integrating the product –CF_3 peaks and comparing to the internal standard –CF_3 peak. The concentration of the palladacycle products were determined using the same procedure as previously described in **Section 3a**.

* Equipment used:
1 mL glass vials: Analytical Sales and Services, Inc. item# 84001.
Stir bars: V&P Scientific, Inc. item# VP 711D.

5b. C–H activation assay results

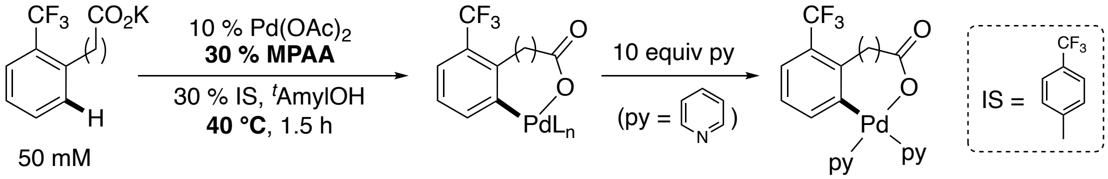

Table S3. C–H activation assay results. Yields were relative to amount of $Pd(OAc)_2$ added. The yields were obtained as outlined in **Section 5a**.

MPAA Ligands			Palladacycle yields	
			***o*-CF₃ benzoic**	***o*-CF₃ phenylacetic**
No ligand			0 %	0 %
α	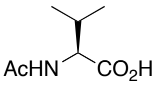	*N*-acetyl-*L*-valine<br>Ac-*α*-*L*-Val-OH	13 %	100 %
	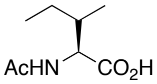	*N*-acetyl-*L*-isoleucine<br>Ac-*α*-*L*-Ile-OH	13 %	100 %
	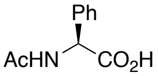	*N*-acetyl-*L*-phenylglycine<br>Ac-*α*-*L*-Ph-OH	1 %	51 %
	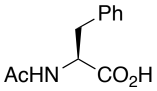	*N*-acetyl-*L*-phenylalanine<br>Ac-*α*-*L*-Phe-OH	21 %	98 %
	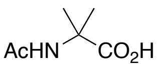	*N*-acetyl-*α*,*α*-dimethylglycine<br>Ac-*α*-Me2-OH	1 %	50 %
***β*²**	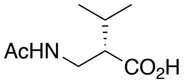	*N*-acetyl-*β*²-*L*-homovaline<br>Ac-*β*²-*L*-Val-OH	6 %	74 %
	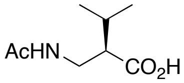	*N*-acetyl-*β*²-*D*-homovaline<br>Ac-*β*²-*D*-Val-OH	9 %	76 %
	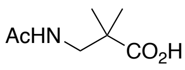	*N*-acetyl-2,2-dimethyl-*β*-alanine<br>Ac-*β*²-Me2-OH	6 %	75 %
***β*³**	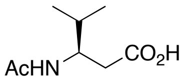	*N*-acetyl-*β*³-*L*-leucine<br>Ac-*β*³-*L*-Val-OH	26 %	34 %
	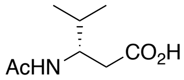	*N*-acetyl-*β*³-*D*-leucine<br>Ac-*β*³-*D*-Val-OH	21 %	60 %
	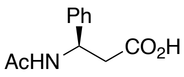	*N*-acetyl-*β*³-*L*-phenylalanine<br>Ac-*β*³-*L*-Ph-OH	32 %	76 %
	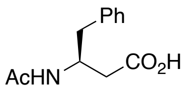	*N*-acetyl-*β*³-*L*-homophenylalanine<br>Ac-*β*³-*L*-Phe-OH	29 %	83 %
	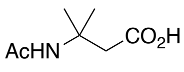	*N*-acetyl-3,3-dimethyl-*β*-alanine<br>Ac-*β*³-Me2-OH	2 %	15 %

6. C–H arylation assay

6a. General experimental procedures

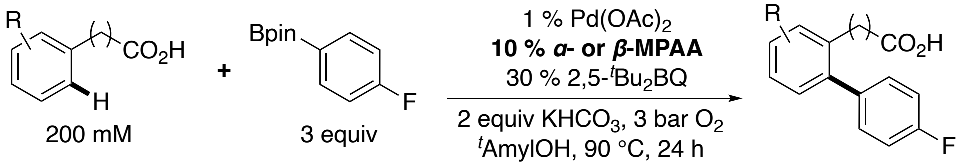

Reactions were carried out in a custom apparatus on an orbital shaker, that allowed up to 35 simultaneous reactions in thick-walled 10 mL microwave tubes under pressurized O_2 atmosphere, under controlled heating and orbital agitation. O_2 was supplied via a custom apparatus, that delivered pressurized O_2 from a gas cylinder to each reaction tube in parallel, through flexible Tygon® tubing and 22-gauge needles. To each tube were added benzoic or phenylacetic acid substrates (100.0 μmol, 1 equiv), 4-fluorophenylboronic acid pinacol ester (66.6 mg, 65 μL, 300.0 μmol, 3 equiv), and three 4 mm glass beads (to help agitating solids). Three stock solutions A, B, and C were prepared in ^{t}AmylOH. The tubes were each charged with 150 μL of stock solution A containing MPAA ligand (10.0 μmol, 0.1 equiv) and 2,5-di-*tert*-butylbenzoquinone (6.6 mg, 30.0 μmol, 0.3 equiv), 150 μL of stock solution B containing potassium bicarbonate (20.0 mg, 200.0 μmol, 2 equiv), and 200 μL of stock solution C containing palladium acetate (1.0 μmol, 0.01 equiv) Stock solutions A and C are prepared in volumetric flasks, and sonication in a warm water bath was required to dissolve the solids for stock solution C. Stock solution B was prepared as a slurry and homogenized by stirring at 23,000 rpm for 10 minutes using an immersion disperser. The tubes were sealed with crimp caps fitted with septa and placed into the reactor. The O_2 lines were attached to each tube by puncturing the septa with needles, and the headspace was evacuated and filled with 3 atm O_2 six times. The tubes were then heated to 90 °C and stirred at 900 rpm on the orbital shaker for 24 h. The tubes were cooled to room temperature, and each charged with 1.0 mL 2 M HCl, followed by 700 μL of stock solution D containing 4-chlorobenzotrifluoride (30.0 μmol, 0.3 equiv, internal standard) in EtOAc delivered via a gas-tight Hamilton® syringe. Without removing the crimp caps, the tubes were shaken vigorously until all solids fully dissolve. The crimp caps were removed, and 600 μL aliquots were taken from the organic phase of each tube and transferred to NMR tubes for $^{19}F\{^1H\}$ NMR analysis. No lock was used, and shimming was performed using the EtOAc solvent peaks on the ^1H channel. The product yields were determined by integrating the product –F peaks and comparing to the internal standard $–CF_3$ peak. Arylation products were synthesized separately or isolated from the arylation reaction mixture (see **Sections 2f** and **2g** for details) and spiked to the NMR samples to identify the product peaks.

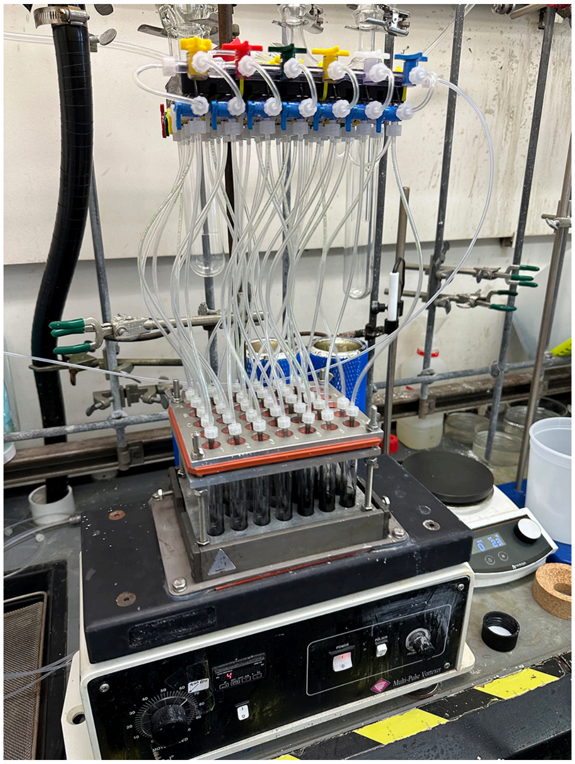

Figure S3. Reaction apparatus used in arylation yield assays.

* Equipment used:
Orbital shaker: Glas-Col® Multi-Pulse Vortexer.
10 mL microwave vials: CEM Corporation® item# 908035.
Crimp caps with septa for microwave vials: Analytical Sales and Services, Inc. item# 62700.
Immersion disperser: IKA® T 18 digital Ultra-Turrax®, fitted with an S18N-10G Dispersing Tool.

6b. NMR analyses of C–H arylation product mixtures

Shown below are typical $^{19}F\{^1H\}$ NMR spectra of C–H arylation product mixtures after the workup procedure described in **Section 6a**. Internal stand 4-chlorobenzotrifluoride (red) appear at –62.0 ppm. Fluorobenzene (cyan, –113.0 ppm), 4,4'-difluorobiphenyl (blue, –115.7 ppm), and 4-fluorophenol (pink, –126.6 ppm) are the common side products in the C–H arylation reaction.[3] Also seen in some spectra are unreacted 4-fluorophenylboronic acid pinacol ester (yellow, –108.3 ppm), which partially hydrolyzes into 4-fluorophenyl boronic acid after the workup (green, –110.9 ppm). The peaks of aryl–F motifs in desired *ortho*-arylation products (brown), di-ortho-arylation products for some phenylacetic acid substrates (green) as well as non-directed arylation products (unlabeled), appear in the region around –113 to –116.5 ppm.

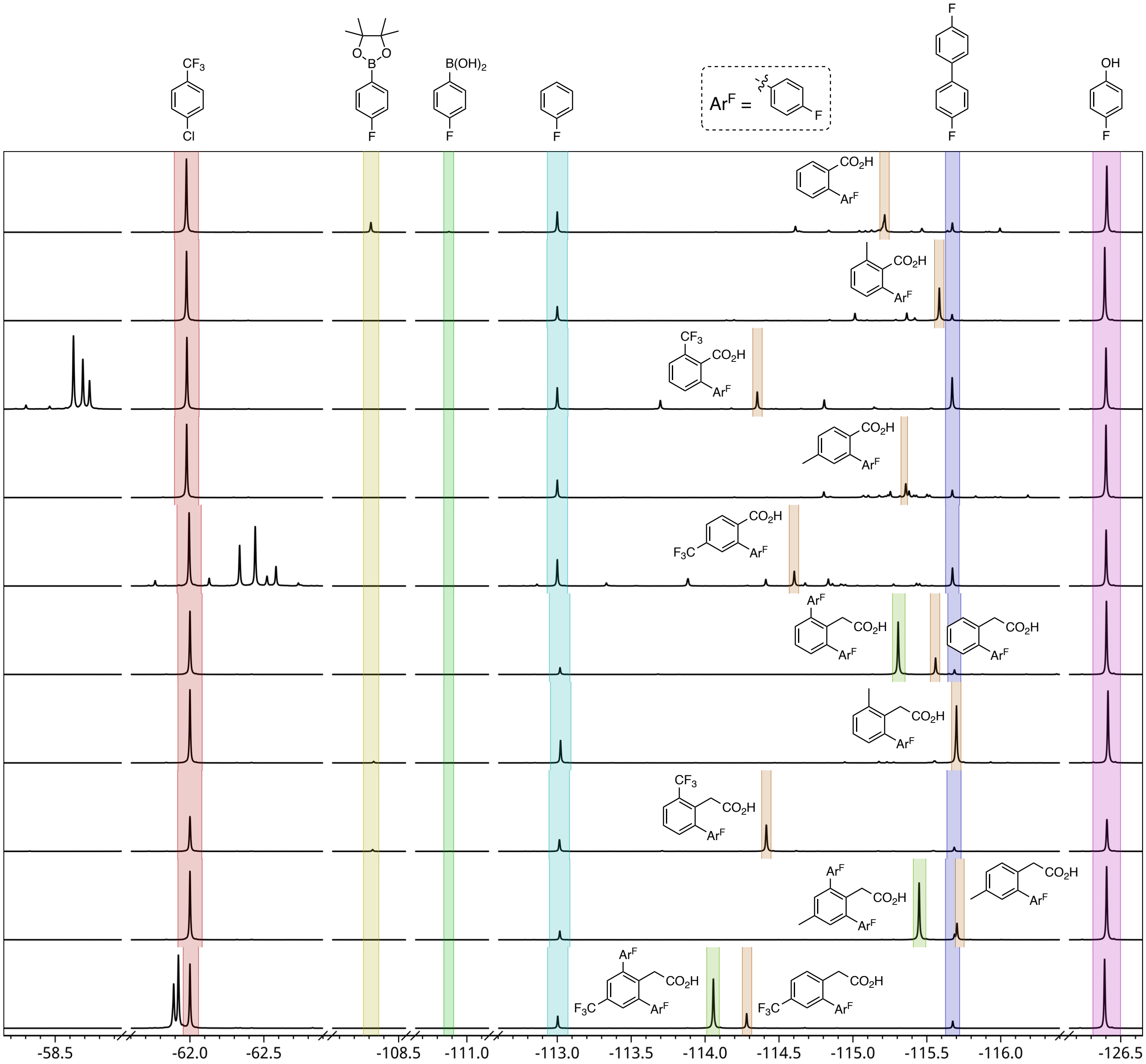

Figure S4. $^{19}F\{^1H\}$ NMR spectra of C–H arylation reaction mixtures after workup of benzoic and phenylacetic acid substrates with **L1** as ligand.

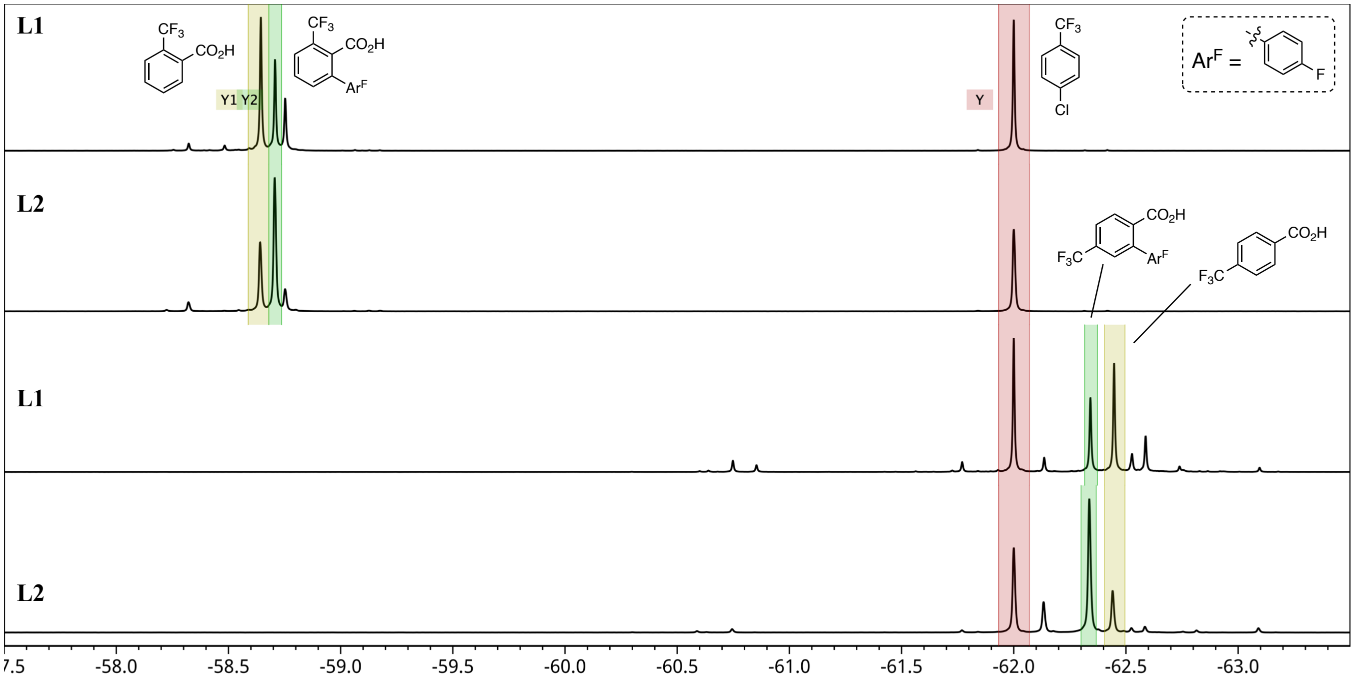

Figure S5. Comparison of the ^{19}F{^{1}H} NMR spectra –CF3 region of C–H arylation reaction mixtures after workup of *o*-CF3 and *p*-CF3 benzoic acid substrates, with **L1** and **L2** as ligand. Unlabeled peaks are unidentified side products.

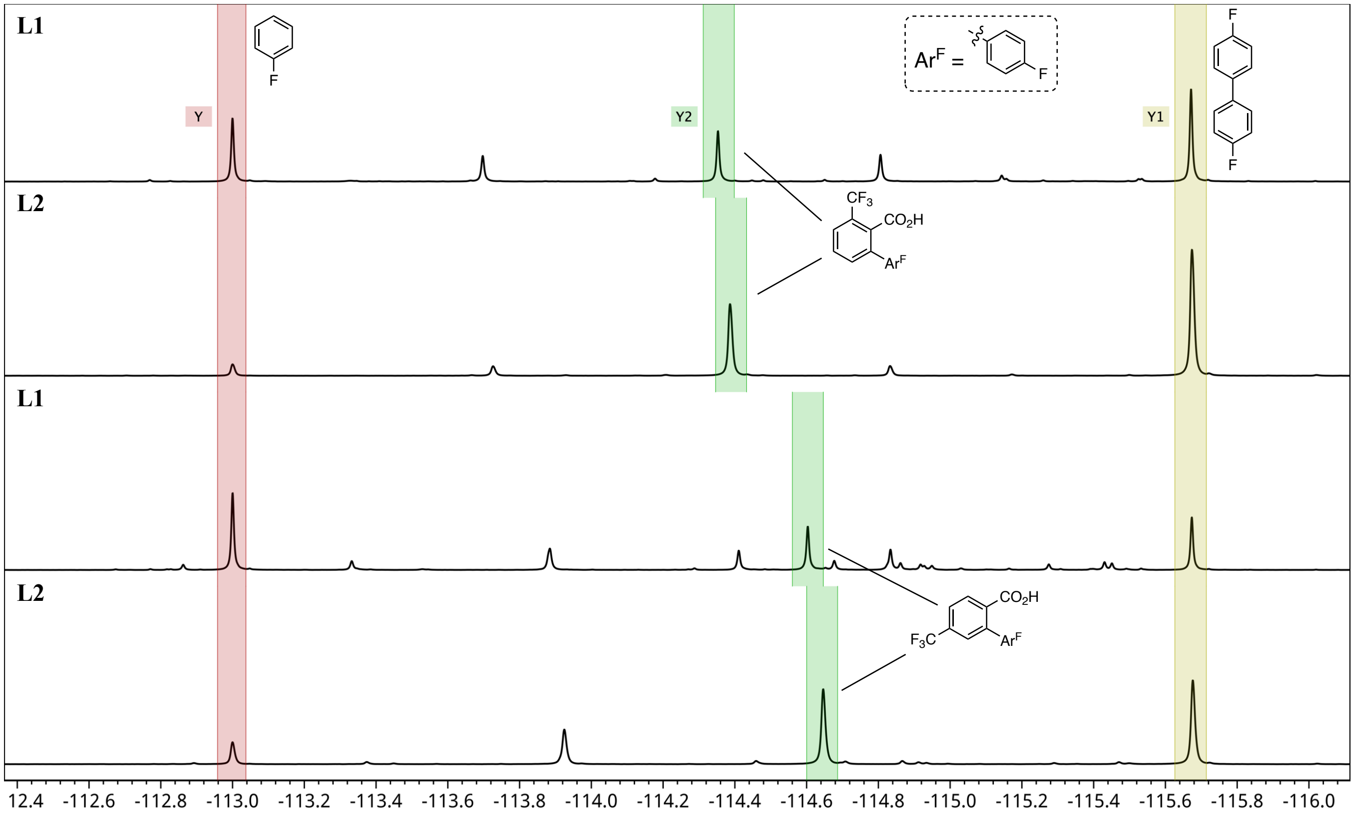

Figure S6. Comparison of the ^{19}F{^{1}H} NMR spectra –F region of C–H arylation reaction mixtures after workup of *o*-CF3 and *p*-CF3 benzoic acid substrates, with **L1** and **L2** as ligand. Unlabeled peaks are unidentified side products.

6c. C–H arylation assay results

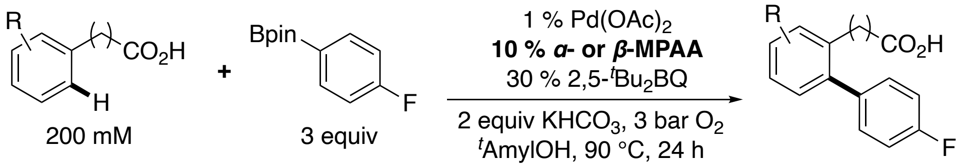

Table S4. C–H arylation yields of benzoic and phenylacetic acid substrates, using either **L1** or **L2** as ligands. Di-arylation products are observed in phenylacetic acid substrates without *ortho*-substituents. The products are identified after isolation from the reaction mixtures, see **Section 2g** for details of the product isolation and characterization. The yields were obtained as outlined in **Section 6a**.

Substrate	L1, Ac-*α*-*L*-Val-OH	L2, Ac-*β*³-*L*-Val-OH
benzoic acid	53 %	85 %
o-CH_3 benzoic acid	63 %	70 %
o-CF_3 benzoic acid	31 %	58 %
p-CH_3 benzoic acid	23 %	78 %
p-CF_3 benzoic acid	26 %	57 %
phenylacetic acid	33 % mono + 55 % di	46 % mono + 32 % di
o-CH_3 phenylacetic acid	100 %	74 %
o-CF_3 phenylacetic acid	96 %	99 %
p-CH_3 phenylacetic acid	29 % mono + 55 % di	41 % mono + 32 % di %
p-CF_3 phenylacetic acid	29 % mono + 50 % di	47 % mono + 29 % di

6d. C–H arylation of phenylacetic acids to minimize di-arylation products

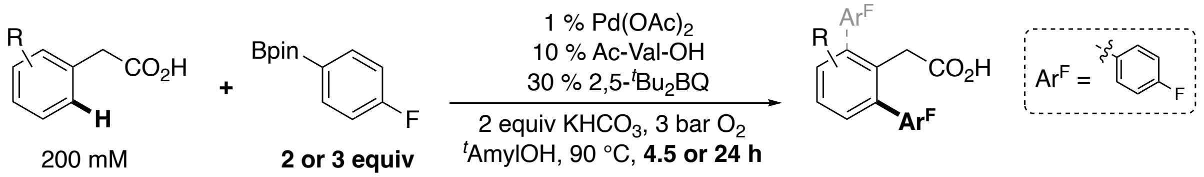

Phenylacetic acid substrates react much faster under the C–H arylation condition than benzoic acid substrates, especially in the case of substrates without *ortho*- substituents, where significant amounts of di-functionalized products are observed. The condition can be modified with lower amount of the Ar^F–Bpin coupling partner, as well as shorter reaction time, to minimize the amounts of di-arylation products and improve selectivity for the desired mono-arylation products.

Table S5. C–H arylation selectivity of phenylacetic acid substrates without *ortho*- substituents under different conditions.

Reaction condition	Substrate	Mono-arylation yield	Di-arylation yield
3 equiv Ar^F–Bpin, 24 h	phenylacetic acid	33 %	55 %
	p-CH_3 phenylacetic acid	29 %	55 %
	p-CF_3 phenylacetic acid	29 %	50 %
2 equiv Ar^F–Bpin, 4.5 h	phenylacetic acid	50 %	29 %
	p-CH_3 phenylacetic acid	46 %	24 %
	p-CF_3 phenylacetic acid	55 %	24 %

7. DFT studies

7a. Computation details

All calculations were carried out by utilizing the Gaussian-16 quantum chemistry software package.[4] Geometries, frequencies, and thermodynamic parameters of these species were calculated at the B3LYP density functional,[5–7] in conjunction with Grimme's empirical dispersion-correction (D3),[8] and Becke and Becke-Johnson (BJ) damping-corrections.[9–11] (this approach is labeled below as a B3LYP-D3(BJ) approximation). In these calculations we utilized the 6-31G (d,p) basis sets for all atoms except Pd for which we used the Lanl2dz basis sets and associated Hay-Wadt effective core potentials.[12,13] Frequency analysis was used to characterize each minimum with zero imaginary frequency and each transition state (TS) structure with only one imaginary frequency. Bulk solvent effects were incorporated for all calculations (including the geometry optimization and frequency calculations) using the SMD continuum solvation model.[14] As a solvent we chose 2-butanol (which has very close characteristics to the experimentally used ^{t}AmylOH solvent). The reported thermodynamic data were computed at a temperature of 298.15 K and at 1 atm of pressure.

7b. Comparison of *ortho*- and *meta*- substituted substrates

Table S6. Calculated energies (relative to corresponding pre-reaction complex, in kcal·mol^{-1}) of Pd(II)-catalyzed C–H activation of *ortho*- and *meta*-substituted substrates, mediated by ligands **L1**.

**Ligand and Substrate**	Ac-*α*-*L*-Val-OH (**L1**)	
	**Δ*G*‡**	**Δ*G***
o-CF_3 benzoate (**1a**)	14.93	–7.81
o-CF_3 phenylacetate (**1b**)	8.59	–6.93
o-CH_3 benzoate	12.1	–10.1
o-CH_3 phenylacetate	9.3	–5.5
o-CH_3-*p*-F benzoate (**3a**)	11.9	–11.5
o-CH_3-*p*-F phenylacetate (**3b**)	10.6	–4.9
m-CF_3 benzoate	9.5	–14.1
m-CF_3 phenylacetate	9.2	–7.8

7c. Comparison of L1 and L2

Table S7. Calculated energies (relative to corresponding pre-reaction complex, in kcal·mol^{-1}) of Pd(II)-catalyzed C–H activation of various substrates, mediated by ligands **L1** and **L2**.

**Ligand and Substrate**	Ac-*α*-*L*-Val-OH (**L1**)		Ac-*β*^{3}-*L*-Val-OH (**L2**)	
	**Δ*G*‡**	**Δ*G***	**Δ*G*‡**	**Δ*G***
o-CF_3 benzoate (**1a**)	14.93	–7.81	13.91	–8.51
o-CF_3 phenylacetate (**1b**)	8.59	–6.93	7.01	–7.44
o-CH_3 benzoate	12.1	–10.1	11.9	–10.4
o-CH_3 phenylacetate	9.3	–5.5	7.7	–5.7

7d. Distortion/interaction analyses

Table S8. Distortion/interaction analyses of the C–H activation transition states (**TS-5** and **TS-6**) with *o*-CH_3 or *o*-CF_3 substrates and ligands **L1** and **L2**. Energies are in kcal·mol^{-1}. The total energy ΔE is relative to the pre-reaction complex.

Ligand	Substrate	$\Delta E_{d\text{-}sub}$	$\Delta E_{d\text{-}cat}$	ΔE_{int}	ΔE
Ac-*α*-*L*-Val-OH (**L1**)	*o*-CF_3 benzoate (**1a**)	31.7	0.9	–14.9	17.7
	o-CF_3 phenylacetate (**1b**)	23.6	1.5	–14.6	10.5
	o-CH_3 benzoate	30.6	0.1	–16.6	14.1
	o-CH_3 phenylacetate	25.2	1.2	–15.3	11.1
Ac-*β*^{3}-*L*-Val-OH (**L2**)	*o*-CF_3 benzoate (**1a**)	29.8	0.4	–16.4	13.8
	o-CF_3 phenylacetate (**1b**)	22.6	1.4	–16.0	8.0

7e. Linear Free Energy Relationship (LFER) analyses

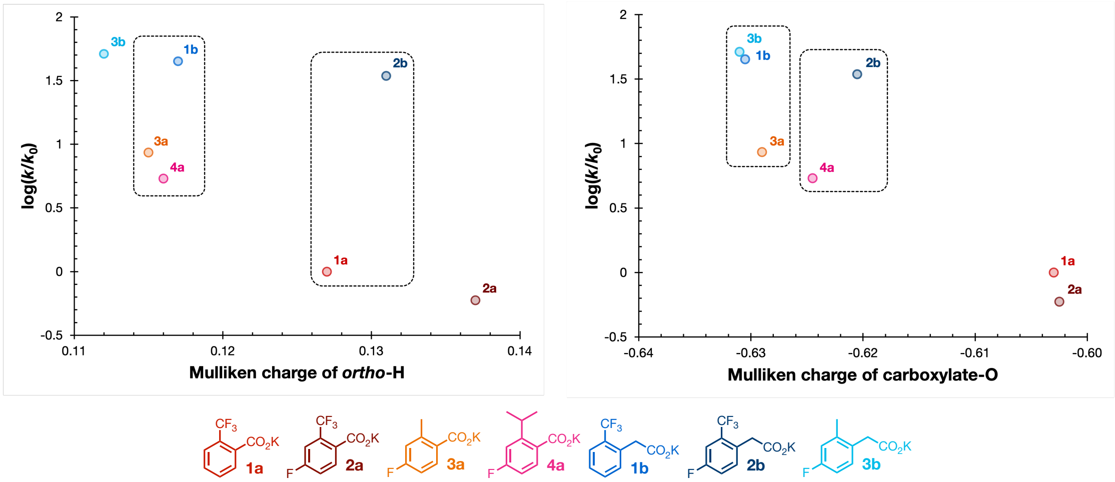

Figure S7. LFER analyses of C–H activation rates relative to computed Mulliken charges on *ortho*-H and carboxylate-O atoms. Rates are relative to those of substrate **1a**. The boxes compare benzoate and phenylacetate substrates with similar electronic features. Mulliken charges on the *ortho*-H atom should influence C–H activation, while charges on carboxylate-O atoms should influence substrate binding to Pd^{II}. In all cases of substrate with similar electronic features, the phenylacetate undergoes more rapid reaction. These observations implicate the chelate ring size of 6- vs. 5-membered transition state structures as being more important than the innate electronic effects of the substrates.

7f. Coordinates of computed structures

Included in this section are coordinates of computed structures for the following substrate / MPAA pairs:
o-CF_3 benzoate (**1a**) and phenylacetate (**1b**): Ac-*α*-*L*-Val-OH (**L1**) and Ac-*β*^{3}-*L*-Val-OH (**L2**)
o-CH_3 benzoate and phenylacetate: Ac-*α*-*L*-Val-OH (**L1**)
m-CF_3 benzoate and phenylacetate: Ac-*α*-*L*-Val-OH (**L1**)
o-CH_3-*p*-F benzoate and phenylacetate: Ac-*α*-*L*-Val-OH (**L1**) and Ac-*β*^{3}-*L*-Val-OH (**L2**)
Each pair of substrate / MPAA have three associated structures: **Sub** (Sub-Pd-MPAA complex), **TS** (C–H activation transition state), and **PdCycle** (MPAA-bound palladacycle).

Sub-5*α* (*o*-CF_3 benzoate, Ac-*α*-*L*-Val-OH, Sub-Pd-MPAA complex):

Pd 1.04116000 0.70603400 -0.52076200
O 0.98653000 0.37218600 1.51433100
C -0.24342600 0.40872000 2.00538100
C -1.35418300 0.53646000 0.95461900
N -0.81644300 1.31902600 -0.16144000
C -1.80602100 -0.86628000 0.46210100
O -0.46289500 0.30306900 3.20748700
H -2.20520100 1.03635900 1.42311400
H -1.97600400 0.73756100 -4.19992500
C 0.55340700 -2.68178900 -2.31081900
F -0.61606700 -3.33816000 -2.21963200
F 1.10062700 -2.70254600 -1.04490500
F 1.37563500 -3.41989100 -3.07799800
C -1.16474400 2.58884600 -0.44641500
O -0.44511800 3.32901700 -1.14805300
H -2.51929100 -2.76655000 1.21655400

H -0.95627700 -1.29124700 -0.08677200
C -2.15508400 -1.81308200 1.61363600
H -1.29196200 -2.02510700 2.25195200
C 2.77751700 -0.70290400 -2.03810200
C 1.42001700 -0.32321300 -2.61389700
C 1.18906700 1.02711100 -2.96960200
C 0.37940200 -1.27207700 -2.79920500
C -0.04091400 1.40144900 -3.55114200
H 2.00254600 1.74336900 -2.90624500
C -0.82211000 -0.88022100 -3.36567100
C -1.02887600 0.45480000 -3.75340600
H -0.19829000 2.43513700 -3.83443600
H -1.61816000 -1.60452900 -3.49092400
H -2.94327000 -1.39152700 2.24858900
C -2.98015400 -0.72251800 -0.50827700
H -3.87873900 -0.37703100 0.01726500
H -2.75183000 -0.00934200 -1.30403200
H -3.21456500 -1.68649200 -0.97229800
O 3.57091700 -1.42531700 -2.62558500
O 2.94369400 -0.17124600 -0.85526200
K 2.52499700 -1.79637400 1.25635200
C -2.49768600 3.10305700 0.07726500
H -2.41572700 3.35271400 1.14118900
H -2.75291200 4.01077200 -0.47218000
H -3.30186700 2.37159700 -0.03583800

Sub-6*α* (*o*-CF_3 phenylacetate, Ac-*α*-*L*-Val-OH, Sub-Pd-MPAA complex):

Pd 0.45795100 0.43484100 0.43253500
O 0.25134700 0.09730700 2.44715500
C -0.97535900 0.29736200 2.88606400
C -1.99481700 0.66181300 1.80024400
N -1.27463000 1.38284200 0.75227400
C -2.66432900 -0.61415600 1.21643800
O -1.26781500 0.17289700 4.07333100
H -2.76886000 1.28108900 2.25954300
H -1.86817200 -1.17598100 0.70752500
C -3.27536700 -1.50324800 2.30279500
H -2.52101000 -1.88675800 2.99539600
O 4.40383300 -0.93693200 0.09190300
C 3.27286000 -0.59058800 -0.25649300
O 2.25808200 -0.60907700 0.55773900
C 3.01154500 -0.08548300 -1.68162900
H 3.27530400 0.97775400 -1.69220000
H 3.69335700 -0.59851000 -2.35921600
C 1.56937800 -0.24398500 -2.08401500
C 0.68089400 0.84102500 -1.88600300
C 1.03863400 -1.45084200 -2.57099800
C -0.69105400 0.71215500 -2.18985400
H 1.08095400 1.82465200 -1.65317200
C -0.32237600 -1.56259000 -2.87398500
C -1.19050700 -0.49015000 -2.68003800
H -1.33385800 1.57097400 -2.04641700
H -0.70507600 -2.50169500 -3.25613900
H -2.24387000 -0.59481300 -2.91490000
C 1.92844100 -2.64850900 -2.76507300
F 1.24798200 -3.73591600 -3.18059000
F 2.56041800 -2.99966100 -1.61427500
F 2.90296600 -2.42416400 -3.68148100
K 2.50745900 -1.05548400 3.10228100
C -1.47185000 2.66522500 0.41380700
O -0.69912400 3.27903500 -0.35695100
H -3.77982100 -2.36197000 1.84716000
H -4.01964800 -0.95197500 2.88988700
C -3.71395500 -0.21810200 0.17526600
H -4.56084800 0.29130400 0.65096500
H -3.29354100 0.45082200 -0.57988800
H -4.10298700 -1.10564300 -0.33531300
C -2.70044600 3.37823100 0.96593900
H -2.78919200 4.34215700 0.46260600
H -3.61716200 2.80371200 0.80846900
H -2.59567600 3.55308900 2.04234700

Sub-5*β* (*o*-CF_3 benzoate, Ac-β^3-*L*-Val-OH, Sub-Pd-MPAA complex):

Pd 1.04116000 0.70603400 -0.52076200
O 0.98653000 0.37218600 1.51433100
C -0.24342600 0.40872000 2.00538100
C -1.35418300 0.53646000 0.95461900
N -0.81644300 1.31902600 -0.16144000
C -1.80602100 -0.86628000 0.46210100
O -0.46289500 0.30306900 3.20748700
H -2.20520100 1.03635900 1.42311400
H -0.95627700 -1.29124700 -0.08677200
C -2.15508400 -1.81308200 1.61363600
H -1.29196200 -2.02510700 2.25195200
C 2.77751700 -0.70290400 -2.03810200
C 1.42001700 -0.32321300 -2.61389700
C 1.18906700 1.02711100 -2.96960200
C 0.37940200 -1.27207700 -2.79920500
C -0.04091400 1.40144900 -3.55114200
H 2.00254600 1.74336900 -2.90624500
C -0.82211000 -0.88022100 -3.36567100
C -1.02887600 0.45480000 -3.75340600
H -0.19829000 2.43513700 -3.83443600
H -1.61816000 -1.60452900 -3.49092400
H -1.97600400 0.73756100 -4.19992500
C 0.55340700 -2.68178900 -2.31081900
F -0.61606700 -3.33816000 -2.21963200
F 1.10062700 -2.70254600 -1.04490500
F 1.37563500 -3.41989100 -3.07799800
C -1.16474400 2.58884600 -0.44641500
O -0.44511800 3.32901700 -1.14805300
H -2.51929100 -2.76655000 1.21655400
H -2.94327000 -1.39152700 2.24858900
C -2.98015400 -0.72251800 -0.50827700
H -3.87873900 -0.37703100 0.01726500
H -2.75183000 -0.00934200 -1.30403200
H -3.21456500 -1.68649200 -0.97229800
O 3.57091700 -1.42531700 -2.62558500
O 2.94369400 -0.17124600 -0.85526200
K 2.52499700 -1.79637400 1.25635200
C -2.49768600 3.10305700 0.07726500
H -2.41572700 3.35271400 1.14118900
H -2.75291200 4.01077200 -0.47218000
H -3.30186700 2.37159700 -0.03583800

Sub-6*β* (*o*-CF_3 phenylacetate, Ac-β^3-*L*-Val-OH, Sub-Pd-MPAA complex):

Pd 0.29821600 0.38473000 0.04741900
H -1.91279600 -0.74419000 -3.71913400

C -1.97510400 1.11538900 2.45464400
C -2.42630500 0.98791300 0.98874300
N -1.37920100 1.48814000 0.09534600
C -2.87057800 -0.43226100 0.56847700
H -3.30616500 1.63144600 0.88567700
H -1.96579800 -1.04630800 0.46286900
C -3.77256300 -1.09441400 1.61606500
H -3.24675700 -1.28084200 2.55717200
O 4.21449500 -1.07582500 0.18709000
C 3.14658100 -0.73527500 -0.33493900
O 2.01388300 -0.80517400 0.29402200
C 3.11278900 -0.19288400 -1.76960000
H 3.36169400 0.87264000 -1.71476400
H 3.89780300 -0.68339300 -2.34458000
C 1.74805400 -0.35760400 -2.38346500
C 0.82186700 0.70617200 -2.27723400
C 1.31217900 -1.55577900 -2.96987300
C -0.49360100 0.56517100 -2.75789300
H 1.15969700 1.68792200 -1.95361800
C 0.00422900 -1.68174000 -3.45303000
C -0.90199800 -0.63074200 -3.34377500
H -1.16966100 1.40741100 -2.68364500
H -0.30540800 -2.61436300 -3.91009800
C 2.24404200 -2.73203600 -3.07725300
F 1.64890700 -3.81710800 -3.61268800
F 2.72316700 -3.10984800 -1.86274700
F 3.32818200 -2.46186800 -3.84676400
K 2.23418500 -1.39886100 2.84087600
C -1.31128000 2.75960800 -0.32854400
O -0.29444500 3.22023100 -0.89961900
H -4.13645600 -2.05884700 1.24534100
H -4.64963400 -0.47250600 1.83433600
C -3.57596600 -0.37202000 -0.78985200
H -4.54647400 0.13173700 -0.69703300
H -2.98098300 0.17889500 -1.52130500
H -3.75788100 -1.37703400 -1.18558200
C -2.52720200 3.66064200 -0.14892500
H -2.31882200 4.61292900 -0.63864900
H -3.42562100 3.21949300 -0.59167400
H -2.73303300 3.84699300 0.91023200
H -2.80815000 0.94082700 3.13801500
H -1.63980200 2.14865700 2.61248000
C -0.82571400 0.22242100 2.88381600
O -0.78677500 -0.26804800 4.02144900
O 0.16427000 0.01763100 2.05912600

TS-5*α* (*o*-CF_3 benzoate, Ac-*α*-*L*-Val-OH, C–H activation transition state):

Pd 1.53368600 -0.85021400 0.37244200
O 1.50688100 -0.94171100 2.44926900
C 0.45154800 -0.36208000 2.97936400
C -0.70870800 -0.06660700 2.00681100
N -0.11867700 0.20681300 0.69937500
C -1.67600000 -1.27871600 1.92613900
O 0.36972500 -0.10322300 4.17798500
H -1.25433100 0.80215300 2.38529500
H -1.09279900 -2.11530900 1.51713400
C -2.21052800 -1.67988300 3.30431300
C 3.92204300 -1.32030200 -1.07356400
C 2.82473600 -1.38466800 -2.11614300
C 1.56353300 -0.84055600 -1.76762300
C 2.96029100 -2.10785500 -3.31154300
C 0.46332900 -1.06093900 -2.61407800
H 1.28534400 0.33660800 -1.23945300
C 1.85463800 -2.28368000 -4.15137100
C 0.60803500 -1.76875700 -3.80284700
H -0.49852000 -0.63437400 -2.34817900
H 1.96787500 -2.84354500 -5.07169200
H -0.24271700 -1.92412200 -4.45849500
C 4.26463500 -2.75854300 -3.70798700
F 4.10634200 -3.58550000 -4.76940500
F 4.78023500 -3.51601600 -2.71116700
F 5.20838800 -1.86137300 -4.06441900
K 4.09842700 -1.20697900 2.65677800
C -0.24808800 1.30727100 -0.01824800
O 0.50478600 1.51338800 -1.03611500
C -2.82709000 -0.97520800 0.96333100
H -2.92028500 -2.50759200 3.20380000
H -2.73744700 -0.84394500 3.77963300
H -1.41354800 -2.00096200 3.97992000
H -2.46113800 -0.68505000 -0.02574200
H -3.45496300 -0.16303000 1.34879000
H -3.46317800 -1.85815100 0.84065400
O 5.09632700 -1.08322000 -1.33975500
O 3.48349100 -1.56259600 0.13898200
C -1.27488700 2.35545900 0.33242600
H -1.38825700 3.04141400 -0.50739100
H -2.24058200 1.90833400 0.57537500
H -0.93400700 2.92277700 1.20570100

TS-6*α* (*o*-CF_3 phenylacetate, Ac-*α*-*L*-Val-OH, C–H activation transition state):

Pd 0.69387600 -0.28654900 0.61828000
O 0.59527100 -0.49356100 2.69630200
C -0.35779200 0.22935800 3.23426400
C -1.43061200 0.74794700 2.25572200
N -0.76660500 1.02031200 0.98140500
C -2.54780700 -0.31725100 2.07243200
O -0.42918900 0.45164800 4.44264700
H -1.86754300 1.65657000 2.67804200
H -2.06475800 -1.20047900 1.63242100
C -3.17868900 -0.71748400 3.40984800
O 4.56031400 -1.04127000 0.63508400
C 3.45154100 -0.91368700 0.09710600
O 2.38784900 -1.51657000 0.54862300
C 3.26706100 0.02486700 -1.10313400
H 3.07193300 1.01851200 -0.68060800
C -0.16833600 -1.28182200 -3.40008600
H -1.32592600 -0.29898200 -1.87630600
H 1.25683700 -2.15998700 -4.74983800
H -1.02972400 -1.62950200 -3.96133700
C 3.60777000 -1.54106200 -3.61138700
F 3.56494000 -2.33792500 -4.69952600
F 4.29219200 -2.22271500 -2.65513100
F 4.37786400 -0.47317200 -3.94265800
K 3.07191800 -1.38074700 3.10355200
C -0.81346200 2.14459700 0.29787700
O -0.09267700 2.30587300 -0.75405400
C -1.70160700 3.29019900 0.71987700
C -3.61703200 0.18252800 1.09729700
H -3.98239600 -1.44211600 3.24292700
H -3.61381700 0.15345000 3.91441300

H 4.20774800 0.08553600 -1.64626800
C 2.09251700 -0.37761500 -1.96279900
C 0.78566700 -0.07823400 -1.50685300
C 2.23989300 -1.13818400 -3.13299200
C -0.32899200 -0.54511600 -2.23096300
H 0.50074800 1.11142400 -1.02364200
C 1.11843200 -1.57743900 -3.84648200
H -2.45381800 -1.17106100 4.09075100
H -3.18560800 0.47316400 0.13507200
H -4.14981900 1.04742500 1.50994800
H -4.35532700 -0.60415800 0.90926600
H -2.72972100 2.95871500 0.87939500
H -1.68280600 4.06063600 -0.05089800
H -1.33504100 3.71797300 1.65907900

TS-5*β* (*o*-CF_3 benzoate, Ac-β^3-*L*-Val-OH, C–H activation transition state):

Pd 1.68001100 -0.82102100 0.45130700
C 0.08733300 0.15494000 3.22403800
C -0.76726800 -0.03640900 1.95695100
N 0.04009600 0.28238000 0.78232300
C -1.37992000 -1.44654900 1.81407600
H -1.59667900 0.67621300 2.02336900
H -0.54962600 -2.14890700 1.65980600
C -2.13277600 -1.86354500 3.08249700
C 3.92390300 -1.48202200 -1.17753200
C 2.75023700 -1.42530600 -2.13278600
C 1.58124000 -0.75991100 -1.68969800
C 2.72446800 -2.13675000 -3.34403400
C 0.41100000 -0.83842500 -2.46411000
H 1.48176100 0.42058700 -1.10624900
C 1.55226100 -2.17776200 -4.10654500
C 0.39716900 -1.53420100 -3.66808400
H -0.48037800 -0.31860800 -2.12961800
H 1.54039400 -2.73126400 -5.03741100
H -0.50768300 -1.58206300 -4.26544100
C 3.91925600 -2.91511700 -3.84291000
F 3.59444600 -3.70463300 -4.89565900
F 4.42422100 -3.73993800 -2.89538700
F 4.92335800 -2.11806800 -4.26570900
K 4.34226700 -2.20363500 2.49107600
C -0.01422300 1.41045100 0.10014300
O 0.83155000 1.65350700 -0.83652100
C -2.29550400 -1.51001800 0.58814000
H -2.61990600 -2.83277200 2.93208300
H -2.91377300 -1.13601400 3.33645700
H -1.46622300 -1.96081800 3.94431400
H -1.76092400 -1.23562900 -0.32576500
H -3.14752500 -0.82793300 0.70140300
H -2.69450700 -2.52122100 0.45467200
O 5.09405000 -1.39209500 -1.54016500
O 3.56113100 -1.65254000 0.06919900
C -1.07608200 2.44891600 0.37358400
H -0.96731200 3.26661700 -0.33886300
H -2.07605200 2.01641500 0.27684100
H -0.97622900 2.84390000 1.38926300
H -0.54709000 0.16262400 4.11175000
H 0.56658000 1.14031100 3.16181300
C 1.20238900 -0.85848700 3.46458400
O 1.44679800 -1.25068000 4.61252400
O 1.92559600 -1.26075100 2.45461100

TS-6*β* (*o*-CF_3 phenylacetate, Ac-β^3-*L*-Val-OH, C–H activation transition state):

Pd 0.72076900 -0.36160700 0.64265300
C -0.63145300 0.78971300 3.47786800
C -1.52215100 0.78143100 2.22353900
N -0.69438000 1.01990300 1.03690900
C -2.37032000 -0.49699500 2.04128100
H -2.22265500 1.61477700 2.33767400
H -1.68378800 -1.31765600 1.79919200
C -3.12110700 -0.86353900 3.32647400
O 4.50945200 -1.57053900 0.41567700
C 3.40443900 -1.23809500 -0.03757900
O 2.29060600 -1.73939000 0.40912400
C 3.28773000 -0.16987900 -1.13235800
H 3.18043400 0.79040700 -0.61278400
H 4.21936300 -0.13462700 -1.69282500
C 2.06728800 -0.40084900 -1.99082300
C 0.80049000 -0.03131700 -1.48018300
C 2.12716200 -1.07721500 -3.21957500
C -0.36293200 -0.34230400 -2.20994600
H 0.65403300 1.12425000 -0.90560400
C 0.95946800 -1.36914800 -3.93354700
C -0.28820400 -1.00237300 -3.43226500
H -1.32757400 -0.03518700 -1.81728400
H 1.03039600 -1.89100800 -4.88075300
H -1.18729900 -1.23378300 -3.99443800
C 3.44797600 -1.54432300 -3.76626600
F 3.31957700 -2.24261000 -4.91377800
F 4.09672100 -2.35406000 -2.88803000
F 4.29330400 -0.51527100 -4.03121800
K 3.11577100 -2.24209100 2.87804000
C -0.65002200 2.16868200 0.39101200
O 0.16737400 2.35780000 -0.58209600
C -1.56637700 3.31846100 0.74420800
C -3.34627700 -0.32386500 0.87374100
H -3.77449600 -1.72450800 3.14967500
H -3.75232000 -0.03347000 3.66756100
H -2.44149900 -1.12919000 4.14171900
H -2.82506900 -0.04634500 -0.04705300
H -4.08346900 0.45838000 1.09396800
H -3.89374100 -1.25321100 0.68387600
H -2.61461000 3.02260500 0.64233000
H -1.36283200 4.15164300 0.07175200
H -1.40535600 3.64377600 1.77619000
H -1.24361300 0.87008800 4.37824800
H -0.00431400 1.68996200 3.44313700
C 0.31279800 -0.39187500 3.66679100
O 0.64202600 -0.74622000 4.80903400
O 0.79248000 -0.99471300 2.61818000

PdCycle-5*α* (*o*-CF_3 benzoate, Ac-*α*-*L*-Val-OH, MPAA-bound palladacycle):

Pd 0.52826300 -0.06107600 0.92572500
O 0.48098800 0.39030800 3.04823600
C -0.70231500 0.74524100 3.45211400
C -1.82118100 0.71006400 2.38376400
H -1.40869700 -0.01928900 -1.62530300
H 0.89180300 -2.17543500 -4.52505500
H -1.16942400 -0.97901500 -3.88920600
C 3.05888400 -2.68958400 -3.07274100

N -1.20044700 1.03090200 1.09155500
C -2.49110400 -0.68700700 2.31824100
O -0.97437600 1.06979200 4.61152600
H -2.57199000 1.45344100 2.66107500
H -1.70767600 -1.39645400 2.01819100
C -3.04498000 -1.11537700 3.68007300
H -2.25912000 -1.20519700 4.43432200
O 3.83371800 -2.04555800 -0.33442300
C 2.72693400 -1.52630600 -0.21088100
O 2.31407600 -1.05045500 0.94809900
C 1.71672800 -1.37474000 -1.31592000
C 0.54606700 -0.66715200 -0.96446100
C 1.84221400 -1.91843000 -2.61304400
C -0.49392900 -0.53803700 -1.89139600
H -0.03080400 1.82875900 -0.73681100
C 0.80018000 -1.75970300 -3.52988900
C -0.36295300 -1.08055000 -3.16856700
F 2.92382400 -3.10903500 -4.35855400
F 3.27583600 -3.80697100 -2.34410500
F 4.18917800 -1.95038000 -3.04599800
K 2.71471300 -0.82002000 3.50500700
C -1.54704500 2.04783000 0.38254900
O -0.82577200 2.39882700 -0.68628000
H -3.78408500 -0.39485200 4.04967900
H -3.54104400 -2.08742600 3.59122700
C -3.58924500 -0.70078900 1.25102000
H -4.40131500 -0.01323500 1.51593900
H -3.20366200 -0.41343700 0.26840000
H -4.01843800 -1.70419600 1.16090200
C -2.72249800 2.94009000 0.62839800
H -2.92247300 3.53198900 -0.26539900
H -3.60873900 2.35966400 0.88890600
H -2.50158500 3.62214800 1.45654600

PdCycle-6*α* (*o*-CF_3 phenylacetate, Ac-*α*-*L*-Val-OH, MPAA-bound palladacycle):

Pd 0.14763900 -0.38524000 0.66169300
O 0.04890000 -0.21186900 2.82400700
C -0.96216500 0.50216700 3.21311200
C -2.00174800 0.84430900 2.12092600
N -1.27635900 1.07257300 0.86204800
C -3.01244600 -0.31997800 1.94386300
O -1.14967700 0.87728700 4.37508500
H -2.54047200 1.74056300 2.43578600
H -2.43343400 -1.18536000 1.59397700
C -3.68642500 -0.68527500 3.26991300
H -2.96728100 -1.03711900 4.01441500
O 3.85255100 -1.66060400 0.81769900
C 2.77132600 -1.43421700 0.25158500
O 1.63815200 -1.80432300 0.77586200
C 2.75728300 -0.66105500 -1.06839000
H 2.88108100 0.39518300 -0.79160600
H 3.66271100 -0.93931100 -1.60210500
C 1.50231500 -0.79961300 -1.90896500
C 0.23881100 -0.59161400 -1.31670900
C 1.55029300 -1.09838400 -3.28604600
C -0.92609000 -0.68628300 -2.08812200
H -0.09198500 1.55459500 -1.06558000
C 0.37992000 -1.19147700 -4.04760400
C -0.85892100 -0.98574500 -3.44994300
H -1.89365000 -0.52030000 -1.62299100
H 0.44406500 -1.42303700 -5.10383300
H -1.76676300 -1.05801800 -4.04162000
C 2.86609600 -1.32016700 -3.98026300
F 2.72460900 -1.58168900 -5.29815400
F 3.55897400 -2.36516800 -3.45487000
F 3.68506100 -0.23903000 -3.88969400
K 2.31502600 -1.44736400 3.30885100
C -1.39956600 2.15337900 0.17106100
O -0.66187500 2.34224800 -0.92402300
H -4.21477500 0.17665200 3.69412400
H -4.42038700 -1.48157300 3.10890500
C -4.05656800 0.02556800 0.87849300
H -4.67625600 0.87201000 1.19742400
H -3.59360500 0.28197800 -0.07908400
H -4.72110900 -0.82808400 0.70867300
C -2.32261700 3.29298200 0.47597700
H -2.26461400 4.03292300 -0.32229200
H -3.35212800 2.94071500 0.57008300
H -2.03724400 3.76541900 1.42107500

PdCycle-5*β* (*o*-CF_3 benzoate, Ac-β^3-*L*-Val-OH, MPAA-bound palladacycle):

Pd 0.78215600 0.69417500 -1.06570100
C -0.89716600 1.65930000 1.72275200
C -1.66845700 1.60045100 0.38252300
N -0.75978900 2.01614700 -0.70408700
C -2.27972400 0.22044800 0.06307600
H -2.49235000 2.31667600 0.45774100
H -1.44541000 -0.47540000 -0.10802700
C -3.11785100 -0.30919100 1.23216700
H -2.50505200 -0.51903500 2.11207300
C 2.23514700 -1.35131200 -2.45953400
C 1.30205300 -0.74557400 -3.47395000
C 0.48008600 0.29588300 -2.98829600
C 1.19003600 -1.15528100 -4.82114100
C -0.44733400 0.90285700 -3.84200000
C 0.26096500 -0.53153000 -5.65670800
C -0.55478800 0.48816400 -5.16811500
H -1.08938500 1.69989800 -3.48583400
H 0.17529700 -0.83977900 -6.69076400
H -1.27434700 0.96196900 -5.82968400
C 2.04386400 -2.24549500 -5.42823300
F 3.36678600 -1.99668800 -5.31543700
C -0.80335600 3.20150400 -1.20607100
O 0.11918400 3.58930100 -2.09208700
H -3.61548300 -1.24086000 0.94303900
H -3.89773700 0.40750700 1.51775500
C -3.11739200 0.28788000 -1.21689800
H -3.97149800 0.96420400 -1.08728700
H -2.53067000 0.64066000 -2.06796600
H -3.51203900 -0.70167700 -1.47046800
O 2.92758300 -2.35008100 -2.64846500
O 2.23917100 -0.72009700 -1.30220900
K 2.01315500 -1.75799100 1.14973300
C -1.82043300 4.25856800 -0.90672400
H -1.76353600 4.55158500 0.14583900
H -1.63236800 5.13344100 -1.52897600
H -2.82950300 3.88599200 -1.10156000
H -1.59951700 1.51833400 2.54513900
H -0.45833800 2.65815800 1.81583200
C 0.22625500 0.63173100 1.84192900
O 0.14256100 -0.30530400 2.66242100

F 1.80612100 -2.37700200 -6.76015100
F 1.80173100 -3.46265600 -4.89276300
O 1.25159900 0.78465600 1.06286400
H 0.77631600 2.86822100 -2.20006200

PdCycle-6*β* (*o*-CF_3 phenylacetate, Ac-β^3-*L*-Val-OH, MPAA-bound palladacycle):

Pd -0.01975200 -0.47718300 0.32142400
C -1.72248500 1.17878200 2.77004900
C -2.43871600 0.87032100 1.44280200
N -1.47373400 0.99014100 0.32824400
C -3.15838500 -0.49387700 1.39159500
H -3.20916600 1.63790700 1.32514000
H -2.39036200 -1.27617900 1.35037100
C -4.00970100 -0.72388100 2.64569400
H -3.40143200 -0.79225900 3.55212900
O 3.62697400 -2.00822600 0.66531800
C 2.59860400 -1.64779300 0.06816900
O 1.41390700 -1.96095200 0.49890500
C 2.70702400 -0.77178100 -1.18012900
H 2.77186300 0.26157700 -0.81239000
H 3.66454900 -0.99079600 -1.64462700
C 1.53465300 -0.87567800 -2.13693400
C 0.22411100 -0.65986800 -1.65721100
C 1.70824200 -1.16561700 -3.50577700
C -0.86179600 -0.73459800 -2.53786000
H 0.06132600 1.41591700 -1.35311800
C 0.61344500 -1.23917000 -4.37378800
C -0.67243500 -1.02321600 -3.89015500
H -1.86633400 -0.55718400 -2.16781100
H 0.77234700 -1.46325700 -5.42163200
H -1.52282300 -1.07890400 -4.56347600
C 3.08011700 -1.40491900 -4.07394000
F 3.05938600 -1.66275200 -5.40018300
F 3.70476700 -2.46064500 -3.48833500
F 3.90387800 -0.33638000 -3.90514400
K 2.02822500 -2.03003100 3.08097100
C -1.45949300 2.04392600 -0.41727100
O -0.51739700 2.21167300 -1.34520200
H -4.73819300 0.08446300 2.78482800
H -4.56920600 -1.66057300 2.55215100
C -4.02048200 -0.59170700 0.12952300
H -4.84188200 0.13480000 0.16243300
H -3.43728600 -0.39765300 -0.77488200
H -4.46105800 -1.58980800 0.03615500
C -2.43599900 3.18077300 -0.36367100
H -2.18040400 3.91385000 -1.12855600
H -3.45292300 2.81887700 -0.53895000
H -2.41167400 3.66433600 0.61682000
H -2.46442100 1.38642300 3.54328200
H -1.14133400 2.10035600 2.63644500
C -0.76013400 0.12290500 3.31393000
O -0.65620400 -0.02612800 4.54378400
O -0.04986800 -0.55168900 2.47150300

Sub-5*α* (*o*-CH_3 benzoate, Ac-*α*-*L*-Val-OH, Sub-Pd-MPAA complex):

Pd 0.98752300 0.67945900 -0.52475100
O 0.96847500 0.48399700 1.53142100
C -0.25665300 0.44950300 2.02376500
C -1.37854000 0.55725800 0.97941300
N -0.85673400 1.33652900 -0.14612300
C -1.83886200 -0.84614300 0.49851600
O -0.47044200 0.31958700 3.22626000
H -2.22794400 1.05452000 1.45432100
H -0.98381600 -1.29420600 -0.02700100
C -2.23329900 -1.76340500 1.65909700
H -1.39190100 -1.97242700 2.32561300
C 2.69416700 -0.84021000 -1.97942900
C 1.38092500 -0.44190600 -2.62121100
C 1.15795700 0.94083900 -2.87411000
C 0.38465800 -1.40296500 -2.93697200
C -0.04995500 1.35766100 -3.47350800
H 1.97701100 1.64678500 -2.76385500
C -0.79917000 -0.94373700 -3.50364600
C -1.02010500 0.41891200 -3.77305700
H -0.20441000 2.40886500 -3.68373400
H -1.57926500 -1.66247600 -3.73822200
H -1.95813300 0.72798000 -4.22362100
C 0.59458200 -2.86406300 -2.64001500
H -0.32212000 -3.42934200 -2.82423400
H 0.88767300 -3.01841900 -1.59535000
H 1.39136600 -3.28447100 -3.26144800
C -1.18385500 2.61949700 -0.39321700
O -0.47268200 3.36512800 -1.09820100
H -2.60223200 -2.71995400 1.27375000
H -3.03301800 -1.31542100 2.26091100
C -2.98967000 -0.69682300 -0.49972400
H -3.88914600 -0.31490700 -0.00124100
H -2.72642800 -0.00971000 -1.30737100
H -3.24121200 -1.66539000 -0.94514600
O 3.46253300 -1.66364600 -2.46456500
O 2.88045000 -0.20709300 -0.84631600
K 3.21811900 -0.82567000 1.68749900
C -2.49049600 3.15159300 0.18101000
H -2.37746300 3.36518900 1.24996000
H -2.73416400 4.08271800 -0.33331100
H -3.31700800 2.44577700 0.06460800

Sub-6*α* (*o*-CH_3 phenylacetate, Ac-*α*-*L*-Val-OH, Sub-Pd-MPAA complex):

Pd 0.47280900 0.45919400 0.43348300
O 0.26915800 0.11754700 2.45331100
C -0.95855500 0.30739400 2.88980900
C -1.97801300 0.68098000 1.80609000
N -1.25896200 1.40859000 0.76222700
C -2.65035100 -0.59125900 1.21566400
O -1.25671100 0.16705500 4.07480900
H -2.75110800 1.29713300 2.27133400
H -1.85560300 -1.15047800 0.70187300
C -3.26183100 -1.48711700 2.29631900
C -0.29748800 -1.58265900 -2.86190500
C -1.19551400 -0.52770100 -2.67806900
H -1.39631200 1.53153800 -2.05146600
H -0.66443800 -2.53311200 -3.24062900
H -2.24578400 -0.65986100 -2.91695700
C 1.99214600 -2.63910800 -2.73741500
H 1.44506400 -3.51485400 -3.09517400
H 2.48227600 -2.90889700 -1.79466300
H 2.78836900 -2.41995800 -3.45833600
K 2.43150800 -1.22181800 3.06235400

H -2.50807700 -1.87552100 2.98675100
O 4.34677900 -1.13918100 -0.00216900
C 3.25030900 -0.65379900 -0.29595000
O 2.26732800 -0.59860500 0.55598900
C 2.98365100 -0.05971900 -1.68335000
H 3.23259700 1.00619900 -1.62265100
H 3.66868000 -0.52185000 -2.39635100
C 1.54306000 -0.22824000 -2.08279100
C 0.63789200 0.84360100 -1.88171700
C 1.06547100 -1.46341300 -2.56090700
C -0.73236700 0.68813900 -2.19100300
H 1.01978300 1.83838100 -1.66355500
C -1.47886200 2.68297300 0.41081100
O -0.72071500 3.30452200 -0.36842900
H -3.76702500 -2.34239800 1.83489000
H -4.00586300 -0.93927800 2.88697100
C -3.70125600 -0.18917200 0.17802600
H -4.54738100 0.31802600 0.65748300
H -3.28150100 0.48280700 -0.57441900
H -4.09137700 -1.07401600 -0.33648100
C -2.71559400 3.38309700 0.96398600
H -2.81506700 4.34582900 0.46033400
H -3.62623500 2.79900800 0.80663000
H -2.61341600 3.55986800 2.04031900

TS-5*α* (*o*-CH_3 benzoate, Ac-*α*-*L*-Val-OH, C–H activation transition state):

Pd 1.53815200 -0.84178300 0.39252900
O 1.50513400 -0.88952300 2.48150300
C 0.44037200 -0.31565700 2.99417200
C -0.71791000 -0.04795400 2.00979400
N -0.12496800 0.20974500 0.70139800
C -1.67228700 -1.27118600 1.94373200
O 0.34404900 -0.03776900 4.18852600
H -1.27513100 0.82046300 2.37223000
H -1.07793600 -2.10823300 1.55200900
C -2.21192700 -1.65586600 3.32457200
C 3.91426700 -1.44506000 -1.06412800
C 2.82267600 -1.43162700 -2.10212500
C 1.56197400 -0.88822400 -1.73314400
C 2.98562800 -2.10352200 -3.32804800
C 0.45684800 -1.08650900 -2.57672400
H 1.28657900 0.30627800 -1.22681600
C 1.85895600 -2.24815700 -4.15444100
C 0.60668400 -1.76235800 -3.78522100
H -0.50347300 -0.66139000 -2.30335700
H 1.97261200 -2.76909900 -5.10118700
H -0.24608400 -1.90414500 -4.44274600
C 4.29710200 -2.70327800 -3.77047300
H 4.15751500 -3.27978700 -4.68876500
H 4.71764600 -3.36322800 -3.00606100
H 5.04505400 -1.92783100 -3.95988600
K 4.07867200 -1.22129400 2.68652600
C -0.25500400 1.30039400 -0.02998900
O 0.50385500 1.49522500 -1.04604400
C -2.82001600 -0.99544700 0.96858200
H -2.91113400 -2.49364200 3.23328200
H -2.75209500 -0.81853200 3.78230700
H -1.41591500 -1.95574600 4.01097800
H -2.44988800 -0.71844400 -0.02266100
H -3.45817500 -0.18289600 1.33609700
H -3.44702600 -1.88634200 0.85659600
O 5.11549100 -1.40696200 -1.33589900
O 3.47394200 -1.55686900 0.17037200
C -1.28883400 2.34874100 0.29988600
H -1.39871500 3.02461100 -0.54860200
H -2.25468900 1.89991800 0.53961000
H -0.95901600 2.92812500 1.16948100

TS-6*α* (*o*-CH_3 phenylacetate, Ac-*α*-*L*-Val-OH, C–H activation transition state):

Pd 0.68803900 -0.31499700 0.63953900
O 0.58235500 -0.48526400 2.73290100
C -0.36878200 0.25074900 3.25126000
C -1.43669300 0.75470800 2.25854000
N -0.76847300 1.00609900 0.98240100
C -2.55547500 -0.31056200 2.08767400
O -0.44703600 0.50074400 4.45498200
H -1.87444300 1.67005500 2.66532800
H -2.07287300 -1.20189600 1.66382900
C -3.19322800 -0.68767700 3.42853300
O 4.55135500 -1.06868300 0.67195300
C 3.44350500 -0.94831000 0.12582000
O 2.38032800 -1.54589200 0.58603700
C 3.26818400 -0.03157500 -1.09201100
H 3.11111700 0.97549700 -0.68288400
H 4.20920400 -0.01177900 -1.64164200
C 2.08821900 -0.41675200 -1.95037800
C 0.78231400 -0.13470500 -1.47565100
C 2.26507600 -1.12847100 -3.14992600
C -0.33697200 -0.59131500 -2.20191000
H 0.49814000 1.06806400 -1.02022300
C 1.12567600 -1.54796300 -3.85336700
C -0.16692500 -1.29020800 -3.39168800
H -1.33463500 -0.36083800 -1.83839300
H 1.26070600 -2.09714200 -4.78179100
H -1.02600100 -1.63269000 -3.96138400
C 3.63769000 -1.47200300 -3.67565600
H 3.56382700 -2.05761800 -4.59571700
H 4.21046900 -2.05896200 -2.94867400
H 4.22413400 -0.57287900 -3.89742600
K 3.05986400 -1.34738100 3.13887200
C -0.80881100 2.12329500 0.28780700
O -0.08614800 2.27004600 -0.76539700
C -1.69094600 3.27900800 0.69566500
C -3.61910500 0.17619200 1.09987000
H -3.99726400 -1.41398200 3.27039900
H -3.62936800 0.19218600 3.91655500
H -2.47199700 -1.13050400 4.12034000
H -3.18216300 0.45019600 0.13529700
H -4.15126500 1.04921500 1.49605300
H -4.35901100 -0.61115800 0.92089700
H -2.72252000 2.95622400 0.85127000
H -1.66222000 4.04311200 -0.08113100
H -1.32777700 3.71227400 1.63365600

PdCycle-5*α* (*o*-CH_3 benzoate, Ac-*α*-*L*-Val-OH, MPAA-bound palladacycle):

Pd 0.52111600 -0.07088200 0.91080300
O 0.48355600 0.38386800 3.04363600
H -1.42136000 -0.01320200 -1.65987400
H 0.92122500 -2.15573700 -4.54780800

C -0.69474100 0.74869700 3.44915500
C -1.81884700 0.71199800 2.38638800
N -1.20655700 1.02530600 1.08866500
C -2.49227400 -0.68395100 2.33118700
O -0.96183200 1.08403000 4.60750000
H -2.56687500 1.45769200 2.66515100
H -1.71186700 -1.39627400 2.03018600
C -3.03979600 -1.10460500 3.69803300
H -2.25020100 -1.19211600 4.44867100
O 3.78986300 -2.11880700 -0.35927200
C 2.69822900 -1.55619400 -0.23691500
O 2.31154300 -1.06300100 0.92676500
C 1.69935800 -1.38086400 -1.33521600
C 0.52814500 -0.67002800 -0.98357900
C 1.86184100 -1.92309100 -2.62911500
C -0.50320900 -0.53159300 -1.91548600
H -0.07535700 1.78544400 -0.77580200
C 0.81673800 -1.74600500 -3.54681600
C -0.35113300 -1.07040700 -3.19661700
H -1.15042200 -0.96310700 -3.92580900
C 3.09128700 -2.68346300 -3.05740800
H 2.99358500 -2.99991800 -4.09978800
H 3.25076600 -3.57052000 -2.43724800
H 3.99505600 -2.07494100 -2.96052900
K 2.71163700 -0.83199000 3.48019000
C -1.55825200 2.03969600 0.37874100
O -0.85283500 2.37790400 -0.70471000
H -3.77558200 -0.38082800 4.06801000
H -3.53825700 -2.07613200 3.61682700
C -3.59627400 -0.70083300 1.27007700
H -4.40498500 -0.00938800 1.53535600
H -3.21503700 -0.42048600 0.28377800
H -4.02896400 -1.70346900 1.18809300
C -2.72309100 2.94259200 0.63844300
H -2.91888700 3.54784800 -0.24718500
H -3.61508000 2.36784700 0.89282300
H -2.49470600 3.61076200 1.47572300

PdCycle-6*α* (*o*-CH_3 phenylacetate, Ac-*α*-*L*-Val-OH, MPAA-bound palladacycle):

Pd 0.12932200 -0.41668900 0.65445100
O 0.03606400 -0.22934300 2.82847300
C -0.95701000 0.51055700 3.21081300
C -2.00274900 0.84530100 2.12214900
N -1.28929900 1.05099100 0.85299500
C -3.02280900 -0.31430400 1.97091300
O -1.12702600 0.91746900 4.36570400
H -2.53332300 1.74889200 2.42985000
H -2.45271000 -1.18773400 1.62656300
C -3.68537000 -0.65755600 3.30863500
H -2.96053900 -1.00281200 4.05081300
O 3.83786700 -1.67064700 0.83346400
C 2.75924400 -1.45416900 0.25501900
O 1.62535100 -1.83361400 0.77170900
C 2.74891400 -0.68958800 -1.06896900
H 2.91308900 0.36407400 -0.79947700
H 3.64108900 -0.99705600 -1.61484700
C 1.49530000 -0.80655100 -1.91279200
C 0.22750600 -0.61092000 -1.32568900
C 1.58619000 -1.07813100 -3.29547600
C -0.93406400 -0.69436700 -2.10400100
H -0.16242800 1.47930800 -1.12208200
C 0.40838200 -1.15468400 -4.05355300
C -0.84249500 -0.96689200 -3.47081000
H -1.90735700 -0.53986700 -1.64659400
H 0.48168100 -1.36387800 -5.11767300
H -1.74264400 -1.03126900 -4.07678000
C 2.91624400 -1.28139700 -3.98343400
H 2.77104500 -1.48284200 -5.04825500
H 3.47084300 -2.12587300 -3.55820300
H 3.55984100 -0.39829800 -3.89684200
K 2.30375200 -1.45688600 3.30139100
C -1.41501000 2.12363800 0.14953100
O -0.70080000 2.28806700 -0.96472800
H -4.20486800 0.21298500 3.72622500
H -4.42513200 -1.45210000 3.16639700
C -4.07562400 0.02396500 0.91182400
H -4.68509300 0.88002700 1.22484500
H -3.62062100 0.26265300 -0.05407800
H -4.74885900 -0.82653500 0.76130900
C -2.31803900 3.27866900 0.45849400
H -2.24275700 4.02268100 -0.33450200
H -3.35462400 2.94406300 0.54279400
H -2.03183700 3.73939000 1.40881100

Sub-5*α* (*m*-CF_3 benzoate, Ac-*α*-*L*-Val-OH, Sub-Pd-MPAA complex):

Pd 1.00005900 0.67021500 -0.54774600
O 1.00143900 0.44914400 1.49844500
C -0.21873000 0.41011900 2.00714800
C -1.35441900 0.52498200 0.97934100
N -0.84433900 1.30464500 -0.15176500
C -1.82988100 -0.87447500 0.50164300
O -0.41263800 0.27036100 3.21080300
H -2.19351300 1.02756600 1.46678200
H -0.98705700 -1.32342200 -0.04270300
C -2.20468800 -1.79529100 1.66594200
H -1.35063500 -2.01323000 2.31314400
C 2.66613100 -0.88332900 -2.01000800
C 1.38079800 -0.42267600 -2.66597300
C 1.16801800 0.96139500 -2.90929500
C 0.37148700 -1.35175600 -2.98067800
C -0.04961800 1.38577100 -3.48056400
H 1.98664900 1.66623900 -2.79453000
C -0.83159900 -0.89835200 -3.49908000
C -1.18117300 2.58403600 -0.40781900
O -0.47354900 3.32611900 -1.11946700
H -2.58740200 -2.74721900 1.28278300
H -2.98890100 -1.34602800 2.28688500
C -3.00044800 -0.71829500 -0.47236000
H -3.88417100 -0.32468000 0.04479100
H -2.74984700 -0.03790800 -1.28931500
H -3.26950100 -1.68515300 -0.90723600
O 3.35463400 -1.79294900 -2.45763100
O 2.89568700 -0.21965100 -0.90364000
K 3.25712700 -0.86297700 1.62672400
C -2.49164700 3.10880000 0.16162500
H -2.37828100 3.33567800 1.22779600
H -2.74586200 4.03132800 -0.36297600
H -3.31029000 2.39252100 0.05491300
H 0.53888000 -2.40558100 -2.78881100
C -1.91522100 -1.89515600 -3.80815800
F -3.14114700 -1.32951000 -3.81221700

C -1.04789200 0.46752700 -3.75505100
H -0.20136700 2.43765300 -3.68694600
H -1.99480800 0.79545400 -4.16755200
F -1.74032100 -2.46669600 -5.02277300
F -1.94183400 -2.90303200 -2.90449700

Sub-6*α* (*m*-CF_3 phenylacetate, Ac-*α*-*L*-Val-OH, Sub-Pd-MPAA complex):

Pd 0.64125900 0.96794000 0.38793800
O 0.39464100 0.66729500 2.39961000
C -0.85851000 0.43473100 2.74255800
C -1.87488900 0.48151400 1.59376700
N -1.31707600 1.32868600 0.53501300
C -2.20088600 -0.93042000 1.03576400
O -1.17137900 0.18600500 3.90412800
H -2.79528800 0.90720300 2.00176500
H -1.31539800 -1.25636000 0.47304400
C -2.49002700 -1.96078100 2.13043900
H -1.62017700 -2.14449200 2.76718000
O 3.90230700 -1.31213700 0.71400000
C 3.16453500 -0.54287400 0.07954700
O 2.68216500 0.54362100 0.60563900
C 2.83880900 -0.82981700 -1.38896200
H 3.60323800 -0.30183400 -1.97361800
H 2.97039100 -1.89833500 -1.56690600
C 1.46750800 -0.38328500 -1.83018000
C 1.15084600 0.99668900 -1.96428500
C 0.48804200 -1.32751800 -2.14785600
C -0.11377200 1.38876500 -2.44185800
C -0.76405900 -0.91766500 -2.61267200
C -1.07604500 0.43349800 -2.76088400
H -0.33941000 2.44199800 -2.54242700
H -2.05108700 0.73654000 -3.12202300
K 2.70859100 -0.42533800 3.09595300
C -1.76480900 2.56696500 0.25166000
O -1.06515200 3.39924100 -0.36453100
H -2.78022700 -2.91412300 1.67586300
H -3.31375800 -1.63377600 2.77560200
C -3.38058100 -0.82422600 0.06549700
H -4.29252400 -0.53410000 0.60148900
H -3.18963900 -0.08166600 -0.71289100
H -3.56893100 -1.78424700 -0.42171600
C -3.18248000 2.93812300 0.66363200
H -3.47548800 3.82920900 0.10574900
H -3.90011600 2.13697400 0.47150100
H 1.93386600 1.74125500 -1.84907900
H -3.21712000 3.17329600 1.73352600
H 0.71247800 -2.38431400 -2.04583700
C -1.76634600 -1.98015200 -2.96683500
F -2.99896100 -1.48111100 -3.19019000
F -1.40275000 -2.65694700 -4.08248200
F -1.87938100 -2.90444900 -1.98015000

TS-5*α* (*m*-CF_3 benzoate, Ac-*α*-*L*-Val-OH, C–H activation transition state):

Pd 1.58046300 -0.71843500 0.42600700
O 1.56660100 -0.82249900 2.49242800
C 0.46837500 -0.34075400 3.03475600
C -0.69580100 -0.05877300 2.06340000
N -0.10992300 0.27128600 0.76905100
C -1.62548600 -1.29823400 1.94225000
O 0.35658900 -0.15775100 4.24429200
H -1.27077000 0.77818000 2.46843800
H -1.01168900 -2.10891900 1.52580400
C -2.17198300 -1.74354000 3.30200100
C 3.91395800 -1.50551500 -1.04758800
K 4.07802900 -1.44926700 2.74316600
C -0.32745900 1.31763500 -0.00468700
O 0.41747400 1.52616200 -1.02838700
C -2.76879500 -1.00646400 0.96601200
H -2.85118000 -2.59245800 3.17160400
H -2.73623800 -0.93511600 3.78201400
H -1.37753900 -2.04986000 3.98696500
C 2.80561000 -1.40458400 -2.06105100
C 1.56126100 -0.81755500 -1.72104200
C 2.96451900 -2.07049400 -3.27385700
C 0.48083700 -0.99259200 -2.60151200
H 1.26738800 0.35178300 -1.18331100
C 1.88270400 -2.19876000 -4.14644900
C 0.63382100 -1.67553200 -3.80601100
H -0.47668700 -0.54371900 -2.36116100
H -0.20995200 -1.79436800 -4.47679000
H -2.39627000 -0.71089200 -0.01883200
H -3.41072200 -0.20205600 1.34438600
H -3.39200900 -1.89724700 0.83500100
O 5.08514200 -1.70516200 -1.37177200
O 3.51402000 -1.43898800 0.19897200
C -1.46115300 2.28163700 0.22770200
H -1.96356200 2.14472100 1.18395400
H -1.07423700 3.30187700 0.16244600
H -2.19366400 2.15069400 -0.57546300
H 3.92520000 -2.51557600 -3.50778400
C 2.07559000 -2.87292700 -5.47493000
F 3.01258200 -3.84571700 -5.42164700
F 0.93533100 -3.43795800 -5.93028800
F 2.48110400 -1.99435000 -6.42460800

TS-6*α* (*m*-CF_3 phenylacetate, Ac-*α*-*L*-Val-OH, C–H activation transition state):

Pd 0.69134900 -0.29527600 0.65371800
O 0.57370400 -0.48187100 2.73112900
C -0.38627100 0.24277000 3.25543300
C -1.44702300 0.75833300 2.26237600
N -0.76797500 1.01784700 0.99405700
C -2.56761700 -0.30258500 2.07521800
O -0.47068400 0.46872400 4.46213900
H -1.88414500 1.67220800 2.67317800
H -2.08386500 -1.19316800 1.65102000
C -3.21921300 -0.68489200 3.40787300
O 4.54459300 -1.05080000 0.71819100
C -0.18021700 -1.28070400 -3.38696800
H -1.31765800 -0.30584800 -1.84885600
H -1.04885400 -1.61027000 -3.94544100
K 3.05341000 -1.33720600 3.16822600
C -0.79596100 2.13798200 0.30051100
O -0.05767500 2.28450300 -0.73903600
C -1.68509000 3.29083600 0.70127800
C -3.62014900 0.19317500 1.08007300
H -4.02355100 -1.40811500 3.23781400
H -3.65790800 0.19366000 3.89592400
H -2.50600700 -1.13324900 4.10435200

C 3.44832100 -0.95419500 0.14613800
O 2.37595700 -1.53354100 0.60750400
C 3.31141700 -0.09974400 -1.12130200
H 3.25382900 0.94211400 -0.77915800
H 4.23268700 -0.20192400 -1.69927600
C 2.09715000 -0.44287300 -1.94374000
C 0.79771300 -0.12461100 -1.47336000
C 2.23715100 -1.15495500 -3.13320500
C -0.32371800 -0.56194200 -2.20365300
H 0.53560000 1.05496200 -0.98526000
C 1.10626900 -1.56912400 -3.84540600
H -3.17343000 0.47067200 0.12097000
H -4.15300200 1.06588300 1.47589000
H -4.36084400 -0.59084000 0.89032400
H -2.71782800 2.96588700 0.84371100
H -1.64763600 4.05741800 -0.07266900
H -1.33312600 3.72090000 1.64502100
H 3.22771700 -1.40635700 -3.49965600
C 1.30224600 -2.29620400 -5.14549100
F 2.29802000 -3.20947500 -5.06790700
F 0.19064900 -2.94981400 -5.54596900
F 1.63935200 -1.44409200 -6.14525700

PdCycle-5*α* (*m*-CF_3 benzoate, Ac-*α*-*L*-Val-OH, MPAA-bound palladacycle):

Pd 0.51013200 -0.02872200 0.89673100
O 0.50115700 0.41032400 3.01796900
C -0.67610200 0.75882600 3.44575900
C -1.81516400 0.71464200 2.40017900
N -1.22417900 1.04150200 1.09534300
C -2.47319100 -0.68897900 2.34888000
O -0.92583300 1.08207600 4.61028100
H -2.56700700 1.45144600 2.69141900
H -1.68719500 -1.39269700 2.04216600
C -3.00547000 -1.11528500 3.72002600
H -2.20954800 -1.19280200 4.46498800
O 3.75260800 -2.11501700 -0.41081400
C 2.68237400 -1.52551600 -0.24785600
O 2.32195000 -1.01370600 0.91113500
C 1.66808100 -1.34030300 -1.32875900
C 0.49827500 -0.62391200 -0.99951200
C 1.85045300 -1.89195100 -2.59553000
C -0.50513000 -0.50345100 -1.96903000
H -0.12331000 1.84077100 -0.77822600
C 0.85056100 -1.74014100 -3.55562900
C -0.32892300 -1.05616200 -3.23906700
H -1.42992800 0.02104300 -1.75315300
H -1.10977900 -0.95185000 -3.98627700
K 2.72951000 -0.81129300 3.46768600
C -1.60674100 2.04654700 0.38752300
O -0.92253300 2.40172100 -0.70316300
H -3.74735100 -0.40016300 4.09454400
H -3.49265600 -2.09277400 3.64274000
C -3.58505700 -0.71892900 1.29638200
H -4.40011600 -0.03767000 1.56811700
H -3.21546700 -0.43397700 0.30695100
H -4.00568900 -1.72692100 1.21803900
C -2.78942400 2.92130500 0.66044800
H -3.01795400 3.51018200 -0.22842700
H -3.66101200 2.32801400 0.94057700
H -2.55998200 3.60603400 1.48404400
H 2.76444700 -2.43394700 -2.81473200
C 0.99782500 -2.36595700 -4.90820700
F 2.29055500 -2.59126300 -5.23521000
F 0.36516300 -3.56611300 -4.98714300
F 0.46681400 -1.59463700 -5.88765000

PdCycle-6*α* (*m*-CF_3 phenylacetate, Ac-*α*-*L*-Val-OH, MPAA-bound palladacycle):

Pd 0.13982200 -0.36780000 0.65520400
O 0.05618500 -0.19146500 2.81886300
C -0.96140100 0.50944800 3.21448400
C -2.00894300 0.84570900 2.12778800
N -1.29196400 1.08129400 0.86525800
C -3.01370500 -0.32391700 1.95333300
O -1.14907500 0.87765600 4.37870000
H -2.55167300 1.73798900 2.44688900
H -2.43173700 -1.18518800 1.59821700
C -3.67923800 -0.69625100 3.28167800
H -2.95461300 -1.04583200 4.02188600
O 3.85814400 -1.67002100 0.79615100
C 2.77650600 -1.43826800 0.23226800
O 1.64148700 -1.77782800 0.77422400
C 2.75660400 -0.69983800 -1.10582700
H 2.95162500 0.35445800 -0.86211800
H 3.61691900 -1.04203000 -1.68678300
C 0.38607900 -1.16914000 -4.04917000
C -0.86166400 -0.94576800 -3.47046900
H -1.91057300 -0.47053400 -1.65890000
H -1.76297800 -1.00000900 -4.07124100
K 2.33721700 -1.40518300 3.29603000
C -1.42383100 2.16497700 0.18028500
O -0.69217100 2.36405000 -0.91698500
H -4.21033800 0.16169300 3.71050000
H -4.40956900 -1.49615900 3.12206800
C -4.06510300 0.01890600 0.89414400
H -4.68804000 0.86072400 1.21896700
H -3.60836700 0.28120500 -0.06483400
C 1.48114100 -0.80462600 -1.91077700
C 0.21766500 -0.57669900 -1.31995400
C 1.54914700 -1.10117600 -3.27304300
C -0.93773500 -0.64972000 -2.10717900
H -0.11763900 1.58158400 -1.06482300
H -4.72555600 -0.83798300 0.72478700
C -2.35180700 3.29803100 0.49472600
H -2.30199600 4.04147000 -0.30084800
H -3.37878300 2.93947700 0.59256600
H -2.06424400 3.76833000 1.44021900
H 2.51602000 -1.27523800 -3.73709300
C 0.50861000 -1.52908200 -5.49713000
F 1.48261800 -0.81717200 -6.11813900
F 0.83343700 -2.83716800 -5.67089400
F -0.63493200 -1.31903000 -6.18707800

Sub-5*α* (*o*-CH_3-*p*-F benzoate, Ac-*α*-*L*-Val-OH, Sub-Pd-MPAA complex):

Pd 1.00726700 0.64904800 -0.52947100
O 0.96657300 0.46703600 1.52672700
C -0.26488300 0.42190100 2.00395000
F -2.22909400 0.79685300 -4.17153100
C 0.59983300 -2.83081600 -2.56922000
H -0.31860700 -3.40365900 -2.71455900

C -1.37788300 0.53483800 0.94938500
N -0.84158000 1.30510500 -0.17568300
C -1.85357300 -0.86198600 0.46633900
O -0.49136200 0.28185600 3.20264900
H -2.22421800 1.04344700 1.41794700
H -1.00740500 -1.31283400 -0.07075700
C -2.24380600 -1.78484300 1.62391900
H -1.39667800 -2.00823900 2.27837100
C 2.71712800 -0.81023000 -2.01022200
C 1.38435700 -0.40714500 -2.61276300
C 1.15771800 0.96692700 -2.89840200
C 0.37609700 -1.37670500 -2.88313700
C -0.06336500 1.38299100 -3.46540600
H 1.97409200 1.67778800 -2.81140200
C -0.82654000 -0.93485200 -3.41258400
C -1.02505200 0.42479200 -3.68414100
H -0.24900300 2.42382800 -3.69536500
H -1.63467200 -1.63148200 -3.60928800
H 0.92800500 -2.96327900 -1.53221000
H 1.37983400 -3.25420500 -3.20956900
C -1.14515400 2.59674800 -0.41479100
O -0.40753600 3.33882300 -1.09503400
H -2.62698000 -2.73418000 1.23465200
H -3.03162000 -1.33418000 2.23914200
C -3.01529300 -0.69694100 -0.51739700
H -3.90510300 -0.31209100 -0.00405900
H -2.76184200 -0.00675100 -1.32559600
H -3.28083000 -1.66096700 -0.96445700
O 3.48639600 -1.60350600 -2.53975400
O 2.90870400 -0.21934100 -0.85585600
K 3.21705300 -0.84995000 1.68224100
C -2.45786700 3.13923400 0.13387600
H -2.36259100 3.35333900 1.20447000
H -2.68462400 4.07181500 -0.38556700
H -3.28717500 2.43952800 0.00259100

Sub-6*α* (*o*-CH_3-*p*-F phenylacetate, Ac-*α*-*L*-Val-OH, Sub-Pd-MPAA complex):

Pd 0.72648000 0.44465700 0.41606400
O 0.37875600 0.24533400 2.43522800
C -0.90426400 0.25420200 2.74319300
C -1.85653200 0.42867500 1.55156900
N -1.13718700 1.18785300 0.51921500
C -2.34091800 -0.93277500 0.98806400
O -1.29297000 0.11878800 3.90081000
H -2.73026000 0.97434500 1.91612900
H -1.48392400 -1.37581300 0.46347400
C -2.79761600 -1.90434300 2.08011800
H -1.97857300 -2.19578100 2.74397300
O 4.42193300 -1.26027400 -0.29414400
C 3.34900600 -0.66074500 -0.37292300
O 2.59506200 -0.42338900 0.66722900
C 2.84567200 -0.08790500 -1.69332500
H 3.29729700 0.90679200 -1.78318700
H 3.24650500 -0.69365500 -2.51125700
C 1.33784800 0.04410500 -1.82916400
C 0.77926200 1.30490300 -2.15269100
C 0.51503800 -1.12808400 -1.96324000
C -0.53292600 1.43125000 -2.61422800
H 1.40865700 2.18513000 -2.08928100
C -0.79182600 -0.98697800 -2.41428900
C -1.28144600 0.27894200 -2.73753900
H -0.96003700 2.39802800 -2.84941500
H -1.43849100 -1.85044400 -2.52524900
F -2.56011700 0.37255900 -3.16749400
C 1.07388700 -2.49289900 -1.66768900
H 0.31928200 -3.26584800 -1.82965700
H 1.42086300 -2.56526600 -0.63207400
H 1.93388100 -2.71279300 -2.31114000
K 2.50042400 -1.08401700 3.16595000
C -1.30721800 2.52010500 0.36718800
O -0.42341300 3.25681000 -0.11488300
H -3.19935400 -2.81598700 1.62480700
H -3.58814900 -1.46345000 2.69840200
C -3.46395000 -0.69035600 -0.02400500
H -4.36809300 -0.33447900 0.48535500
H -3.18165000 0.05256600 -0.77189300
H -3.72160300 -1.61677900 -0.54867800
C -2.65102400 3.12511100 0.75327800
H -2.74872900 4.08617300 0.24504200
H -3.49541500 2.48512100 0.48583000
H -2.68945900 3.30313600 1.83409900

TS-5*α* (*o*-CH_3-*p*-F benzoate, Ac-*α*-*L*-Val-OH, C–H activation transition state):

Pd 1.54554600 -0.81800000 0.39270700
O 1.51299100 -0.87723400 2.47434600
C 0.44285100 -0.31565500 2.99282600
C -0.71677700 -0.04753000 2.01072800
N -0.12429200 0.21909200 0.70415300
C -1.66573600 -1.27487600 1.93841100
O 0.34674700 -0.05137300 4.18959000
H -1.27743600 0.81698300 2.37723500
H -1.06625800 -2.10858800 1.54732900
C -2.20903000 -1.66437400 3.31643800
C 3.91655900 -1.43892900 -1.06908100
C 2.82278000 -1.42504400 -2.10312300
C 1.56666500 -0.87047600 -1.73903800
C 2.97798900 -2.11172500 -3.32453600
C 0.45493200 -1.05882700 -2.57345000
H 1.29467400 0.31588400 -1.21832800
C 1.85663500 -2.25653400 -4.15356200
C 0.62743900 -1.74744300 -3.76279600
H -0.50964600 -0.63039200 -2.32630000
C 4.28276200 -2.72554200 -3.76571500
H 4.13738200 -3.30650300 -4.67983300
H 4.69673300 -3.38264900 -2.99563500
H 5.03546700 -1.95603500 -3.95886900
K 4.08549300 -1.22207300 2.68951300
C -0.26022200 1.30958000 -0.02739400
O 0.50015000 1.50901200 -1.04067700
C -2.81082800 -1.00235500 0.95925800
H -2.90375400 -2.50536400 3.22082800
H -2.75525800 -0.83038000 3.77307100
H -1.41443900 -1.96141600 4.00566600
H -2.43830500 -0.72378500 -0.03064400
H -3.45313000 -0.19225700 1.32488100
H -3.43418900 -1.89545700 0.84473900
O 5.11681200 -1.42301000 -1.34676100
O 3.48160900 -1.52568400 0.16932700
C -1.30272100 2.34948000 0.30112700
H -1.41595200 3.02511400 -0.54709900
H -2.26553500 1.89312500 0.53874600

H 1.93631100 -2.78052100 -5.10043400
F -0.43642100 -1.92396000 -4.57905000
H -0.97868100 2.93044700 1.17185400

TS-6*α* (*o*-CH_3-*p*-F phenylacetate, Ac-*α*-*L*-Val-OH, C–H activation transition state):

Pd 0.69407000 -0.29907300 0.64031400
O 0.58988100 -0.48001600 2.72518600
C -0.36958100 0.24286500 3.24998800
C -1.43726300 0.75124000 2.25982000
N -0.76612800 1.01362400 0.98767100
C -2.55240500 -0.31627900 2.07830200
O -0.45159300 0.47512100 4.45631500
H -1.87840100 1.66257100 2.67195500
H -2.06497800 -1.20553500 1.65555600
C -3.19845400 -0.69795100 3.41383700
O 4.55972100 -1.05399200 0.65750200
C 3.45008100 -0.93287600 0.11595900
O 2.38748100 -1.52685400 0.58307900
C 3.26997400 -0.01958900 -1.10378400
H 3.11648300 0.98901700 -0.69740100
H 4.20815100 -0.00203700 -1.65837200
C 2.08596800 -0.40620600 -1.95566400
C 0.78404200 -0.11305300 -1.48214600
C 2.25661300 -1.13839900 -3.14598300
C -0.34609700 -0.56695000 -2.19232100
H 0.50866000 1.08018900 -1.00977200
C 1.11949500 -1.56212200 -3.84629100
C -0.15144000 -1.27674200 -3.36164400
H -1.35004200 -0.33984400 -1.84861400
H 1.21869300 -2.12461100 -4.76944900
F -1.22608600 -1.71404100 -4.06449300
C 3.62489200 -1.49887300 -3.66927400
H 3.54705200 -2.09674500 -4.58059900
H 4.19251200 -2.07786000 -2.93222900
H 4.21496900 -0.60523600 -3.90211700
K 3.07365200 -1.33420400 3.13337200
C -0.80540600 2.13439800 0.29789100
O -0.07783100 2.28722400 -0.75035500
C -1.69393400 3.28437400 0.70764000
C -3.61000400 0.17053100 1.08409400
H -3.99843800 -1.42709800 3.24853300
H -3.64171900 0.17952500 3.89974000
H -2.48061300 -1.13891900 4.11029100
H -3.16712300 0.44560700 0.12252200
H -4.14559100 1.04267600 1.47750300
H -4.34779000 -0.61752900 0.89976300
H -2.72578700 2.95700900 0.85157100
H -1.66068300 4.05482100 -0.06264800
H -1.34010600 3.71055700 1.65239100

PdCycle-5*α* (*o*-CH_3-*p*-F benzoate, Ac-*α*-*L*-Val-OH, MPAA-bound palladacycle):

Pd 0.52292700 -0.05990400 0.90923700
O 0.48908000 0.38970200 3.03534500
C -0.69040000 0.74822100 3.44588200
C -1.81634600 0.71180300 2.38523000
N -1.20561900 1.03229400 1.08833800
C -2.48476500 -0.68642000 2.32631100
O -0.95457100 1.07641400 4.60640300
H -2.56625100 1.45430700 2.66727100
H -1.70080200 -1.39618100 2.02846000
C -3.03621700 -1.10881100 3.69103200
H -2.24952500 -1.19185400 4.44518500
O 3.78011300 -2.12852800 -0.35579200
C 2.69259600 -1.55761100 -0.23594800
O 2.31038800 -1.05506200 0.92594200
C 1.69590700 -1.38042200 -1.33297600
C 0.52877900 -0.66061400 -0.98312600
C 1.85392300 -1.92852100 -2.62580400
C -0.50375800 -0.51058800 -1.91025300
H -0.06784600 1.81801600 -0.76503100
C 0.81638500 -1.75074900 -3.54938900
C -0.32794100 -1.06204100 -3.17404100
H -1.42635100 0.01091700 -1.68355100
H 0.89017000 -2.15352700 -4.55410100
F -1.32154100 -0.92125400 -4.08532700
C 3.07677800 -2.69907200 -3.05325500
H 2.97515600 -3.02315800 -4.09245900
H 3.23147300 -3.58035400 -2.42429200
H 3.98247000 -2.09291300 -2.96171200
K 2.72022100 -0.82382200 3.47865500
C -1.56303300 2.04631900 0.38065800
O -0.85643300 2.39543600 -0.69846400
H -3.77715200 -0.38829000 4.05706300
H -3.52959100 -2.08277600 3.60831500
C -3.58421300 -0.70704300 1.26060800
H -4.39597300 -0.01768700 1.52180900
H -3.19978400 -0.42658600 0.27557000
H -4.01362300 -1.71100800 1.17783400
C -2.73651900 2.93793800 0.63914500
H -2.94063200 3.53663300 -0.24910400
H -3.62187000 2.35570100 0.89911000
H -2.51197800 3.61300900 1.47198500

PdCycle-6*α* (*o*-CH_3-*p*-F phenylacetate, Ac-*α*-*L*-Val-OH, MPAA-bound palladacycle):

Pd 0.14227600 -0.39617600 0.66555300
O 0.04591900 -0.20992900 2.83171800
C -0.96523400 0.50547200 3.21648100
C -2.00523100 0.84209100 2.12253900
N -1.28144100 1.06351100 0.86185600
C -3.01673300 -0.32236800 1.95185600
O -1.15383400 0.88732000 4.37650000
H -2.54414100 1.73975700 2.43312300
H -2.43755300 -1.19186200 1.61262600
C -3.69727200 -0.67372800 3.27831800
H -2.98170200 -1.01626500 4.03054300
O 3.85433600 -1.64679000 0.82877000
C 0.38909200 -1.16647000 -4.04577100
C -0.83338400 -0.96236900 -3.42691500
H -1.92115400 -0.52256600 -1.63186100
H 0.42189700 -1.38103000 -5.10901200
F -1.96598600 -1.03958500 -4.17510400
C 2.88945700 -1.30940800 -3.99149100
H 2.73394700 -1.52016600 -5.05268200
H 3.44109600 -2.15271800 -3.56079100
H 3.53736700 -0.42864600 -3.91693900
K 2.33617300 -1.40556200 3.30759200
C -1.40346400 2.14205800 0.16706200
O -0.67191700 2.32278600 -0.93349600

C 2.77234300 -1.43617600 0.25509800
O 1.64160200 -1.80676500 0.78585300
C 2.75406800 -0.68974200 -1.07897300
H 2.93109500 0.36584900 -0.82572000
H 3.63832100 -1.01263100 -1.62893800
C 1.49228200 -0.80883000 -1.90985200
C 0.23125000 -0.60180700 -1.31221600
C 1.56860200 -1.09141400 -3.29159800
C -0.94223400 -0.68055300 -2.07257000
H -0.11647300 1.52560100 -1.08278500
H -4.22872000 0.19240400 3.69002000
H -4.42970500 -1.47246700 3.12244400
C -4.05535100 0.01458900 0.87830600
H -4.67280000 0.86740300 1.18444500
H -3.58741400 0.25734900 -0.08037200
H -4.72274800 -0.83831000 0.71605100
C -2.32039700 3.28675200 0.47205800
H -2.24309100 4.03593500 -0.31585300
H -3.35449400 2.94182700 0.54328300
H -2.04844000 3.74458800 1.42799400

8. X-ray crystallography

8a. 4-Methylpyridine supported 5-membered palladacycle (1a-PdCycle-MePy)

Data Collection:

A colorless crystal with approximate dimensions 0.03 × 0.03 × 0.02 mm^3 was selected under oil under ambient conditions and attached to the tip of a MiTeGen MicroMount©. The crystal was mounted in a stream of cold nitrogen at 100(1) K and centered in the X-ray beam by using a video camera. The crystal evaluation and data collection were performed on a Bruker D8 VENTURE PhotonIII four-circle diffractometer with Cu Kα (λ = 1.54178 Å) radiation and the detector to crystal distance of 4.7 cm.[15] The initial cell constants were obtained from a 180° φ scan conducted at a 2θ = 50° angle with an exposure time of 1 second per frame. The reflections were successfully indexed by an automated indexing routine built into the APEX3 program. The final cell constants were calculated from a set of 9789 strong reflections from the actual data collection. The data were collected by using a full sphere data collection routine to survey reciprocal space to the extent of a full sphere to a resolution of 0.80 Å. A total of 33146 data were harvested by collecting 38 sets of frames with 1.0º scans in ω and φ with exposure times of 10–20 sec per frame. These highly redundant datasets were corrected for Lorentz and polarization effects. The absorption correction was based on fitting a function to the empirical transmission surface as sampled by multiple equivalent measurements.[16]

Structure Solution and Refinement:

The diffraction data were consistent with the space groups $P\bar{1}$ and $P1$. The E-statistics strongly suggested the non-centrosymmetric space group $P1$. However, the inversion symmetry present in the structure is unambiguous and the space group $P\bar{1}$ yielded chemically reasonable and computationally stable results of refinement.[17–22] A successful solution by intrinsic phasing provided most non-hydrogen atoms from the *E*-map. The remaining non-hydrogen atoms were located with an alternating series of least-squares cycles and difference Fourier maps. All non-hydrogen atoms were refined with anisotropic displacement coefficients. All hydrogen atoms were included in the structure factor calculation at idealized positions and were allowed to ride on the neighboring atoms with relative isotropic displacement coefficients. There is one partially occupied molecule of water per Pd complex. This solvent molecule has an occupancy of 59.0(6) % and was refined with geometric constraints. The final least-squares refinement of 268 parameters against 3891 data resulted in residuals R (based on F^2 for $I \geq 2\sigma$) and wR (based on F^2 for all data) of 0.0227 and 0.0575, respectively. The final difference Fourier map was featureless.

Summary:

Crystal Data for $C_{20}H_{18.18}F_3N_2O_{2.59}Pd$ (M = 491.38 g/mol): triclinic, space group $P\bar{1}$ (no. 2), a = 8.0327(10) Å, b = 11.3267(12) Å, c = 11.5088(11) Å, α = 101.596(6)°, β = 109.284(6)°, γ = 95.257(6)°, V = 953.96(18) Å^3, Z = 2, T = 101.00 K, μ(Cu Kα) = 8.310 mm^{-1}, $Dcalc$ = 1.711 g/cm^3, 33146 reflections measured (8.088° ≤ 2Θ ≤ 149.238°), 3891 unique (R_{int} = 0.0355, R_{sigma} = 0.0188) which were used in all calculations. The final R_1 was 0.0227 (I > 2σ(I)) and wR_2 was 0.0575 (all data).

Acknowledgement:

The purchase of the Bruker D8 VENTURE Photon III X-ray diffractometer was partially funded by NSF Award #CHE-1919350 to the UW–Madison Department of Chemistry.

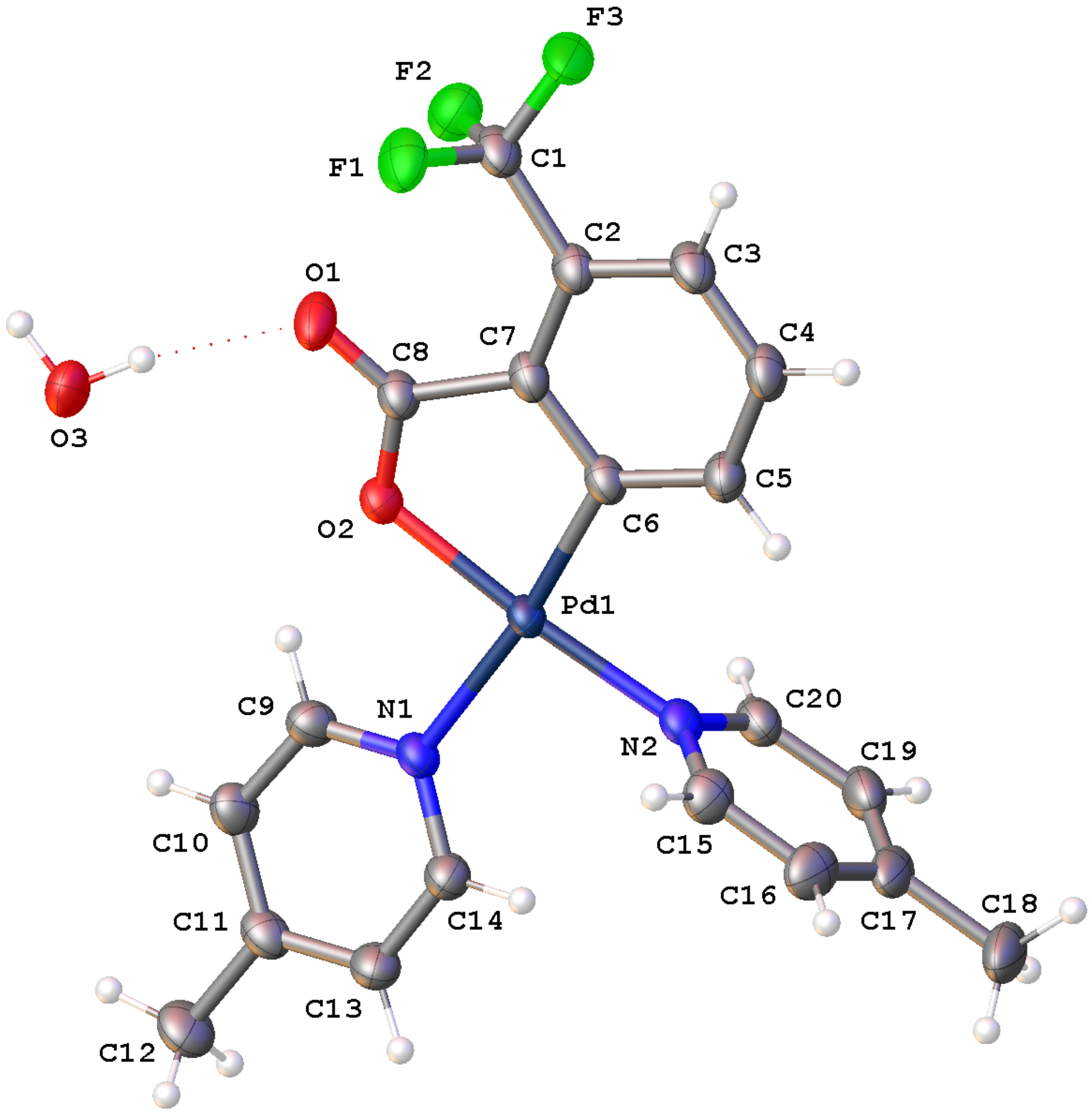

Figure S8. A molecular drawing of the asymmetric unit in **1a-PdCycle-MePy** shown with 50% probability ellipsoids. The O3 water molecule is present 59.0(6) % of the time.

Table S9. Crystal data and structure refinement for **1a-PdCycle-MePy**.

Identification code	**1a-PdCycle-MePy**
Empirical formula	$C_{20}H_{17}F_3N_2O_2Pd \cdot 0.59(H_2O)$
Formula weight	491.38
Temperature/K	101.00
Crystal system	triclinic
Space group	$P\bar{1}$
a/Å	8.0327(10)
b/Å	11.3267(12)
c/Å	11.5088(11)
α/°	101.596(6)
β/°	109.284(6)
γ/°	95.257(6)
Volume/Å^3	953.96(18)
Z	2
ρ_{calc}g/cm^3	1.711
μ/mm^{-1}	8.310
F(000)	492.0
Crystal size/mm^3	0.03 × 0.03 × 0.02
Radiation	Cu Kα (λ = 1.54178)
2Θ range for data collection/°	8.088 to 149.238
Index ranges	$-10 \leq h \leq 10$, $-14 \leq k \leq 14$, $-14 \leq l \leq 14$
Reflections collected	33146
Independent reflections	3891 [R_{int} = 0.0355, R_{sigma} = 0.0188]
Data/restraints/parameters	3891/0/268
Goodness-of-fit on F^2	1.104
Final R indexes [I>=2σ (I)]	R_1 = 0.0227, wR_2 = 0.0568
Final R indexes [all data]	R_1 = 0.0242, wR_2 = 0.0575
Largest diff. peak/hole / e Å^{-3}	0.95/-0.64

Table S10. Fractional Atomic Coordinates ($\times10^4$) and Equivalent Isotropic Displacement Parameters ($Å^2\times10^3$) for **1a-PdCycle-MePy**. U_{eq} is defined as 1/3 of the trace of the orthogonalized U_{IJ} tensor.

Atom	*x*	*y*	*z*	U(eq)
Pd1	2804.1(2)	4978.9(2)	5449.5(2)	24.30(6)
F1	5244(2)	10011.4(13)	8370.8(14)	42.9(3)
F2	7571(2)	9278.6(14)	8235.2(15)	42.7(3)
F3	7217(3)	9753.9(14)	10026.2(14)	53.8(4)
O1	4710(3)	8526.7(15)	6019.9(15)	42.4(4)
O2	3563(2)	6582.3(14)	5118.7(14)	29.4(3)
N1	1690(2)	4134.9(17)	3446.5(16)	27.1(4)
N2	2165(3)	3413.4(17)	5920.9(16)	29.5(4)
C1	6346(4)	9240(2)	8776(2)	34.7(5)
C2	5386(3)	7962(2)	8562(2)	28.7(4)
C3	5381(3)	7568(2)	9637(2)	32.7(5)
C4	4547(3)	6392(2)	9501(2)	34.1(5)
C5	3777(3)	5587(2)	8317(2)	31.0(5)
C6	3781(3)	5961(2)	7227(2)	28.0(4)
C7	4540(3)	7159(2)	7355(2)	27.4(4)
C8	4305(3)	7496(2)	6108(2)	29.0(5)
C9	2165(3)	4673(2)	2637(2)	32.2(5)
C10	1437(3)	4211(2)	1336(2)	33.9(5)
C11	167(3)	3150(2)	812(2)	32.7(5)
C12	-617(4)	2602(3)	-597(2)	45.1(6)
C13	-332(4)	2604(2)	1656(2)	35.9(5)
C14	445(3)	3114(2)	2939(2)	32.9(5)
C15	598(4)	3155(2)	6084(2)	36.0(5)
C16	107(4)	2089(2)	6381(2)	43.0(6)
C17	1240(4)	1237(2)	6535(2)	43.9(7)
C18	743(5)	60(3)	6847(3)	59.1(9)
C19	2868(4)	1513(2)	6375(2)	43.4(6)
C20	3280(3)	2589(2)	6063(2)	34.6(5)
O3	3479(4)	9136(3)	3663(3)	42.3(10)

Table S11. Anisotropic Displacement Parameters ($Å^2\times10^3$) for **1a-PdCycle-MePy**. The Anisotropic displacement factor exponent takes the form: $-2\pi^2[h^2a^{*2}U_{11}+2hka^*b^*U_{12}+\ldots]$.

Atom	U_{11}	U_{22}	U_{33}	U_{23}	U_{13}	U_{12}
Pd1	30.19(9)	24.20(9)	16.02(8)	4.41(5)	5.72(6)	3.25(6)
F1	67.8(10)	26.9(7)	36.5(7)	8.8(6)	18.6(7)	17.9(7)
F2	47.6(8)	37.4(8)	45.1(8)	13.6(6)	17.4(7)	5.7(6)

Atom	U_{11}	U_{22}	U_{33}	U_{23}	U_{13}	U_{12}
F3	84.3(12)	35.5(8)	22.1(7)	3.2(6)	1.9(7)	-11.3(8)
O1	73.4(13)	26.6(8)	24.3(8)	8.4(6)	13.6(8)	4.7(8)
O2	36.6(8)	29.6(8)	19.3(7)	5.9(6)	7.7(6)	2.1(6)
N1	31.5(9)	27.8(9)	19.0(8)	4.4(7)	6.0(7)	4.9(7)
N2	38.3(10)	28.7(9)	17.3(8)	3.6(7)	6.5(7)	3.2(8)
C1	48.8(14)	30.2(11)	19.6(10)	3.9(8)	6.7(9)	7.0(10)
C2	37.7(12)	25.6(10)	22.8(10)	6.3(8)	9.1(9)	10.8(9)
C3	45.6(13)	29.5(11)	19.2(10)	3.3(8)	7.7(9)	9.0(10)
C4	48.1(14)	35.1(12)	22.1(10)	11.3(9)	13.2(10)	10.5(10)
C5	41.1(13)	27.7(11)	23.9(10)	8.7(8)	9.9(9)	6.1(9)
C6	34.8(11)	29.0(11)	19.4(10)	5.8(8)	7.8(8)	8.6(9)
C7	34.5(11)	28.2(11)	20.1(10)	7.3(8)	8.3(8)	11.1(9)
C8	38.6(12)	27.4(11)	20.2(10)	5.6(8)	9.1(9)	8.1(9)
C9	32.9(12)	33.8(12)	26.6(11)	4.5(9)	9.8(9)	0.0(9)
C10	37.9(12)	40.8(13)	24.6(11)	8.4(9)	13.5(9)	4.4(10)
C11	38.4(12)	33.7(12)	21.8(10)	2.0(9)	7.5(9)	8.5(10)
C12	58.6(17)	45.6(15)	23.2(12)	0.7(10)	10.3(11)	4.1(13)
C13	44.4(14)	29.3(11)	26.9(11)	3.2(9)	7.3(10)	0.1(10)
C14	41.5(13)	28.1(11)	25.0(11)	5.6(8)	8.3(9)	1.4(9)
C15	46.9(14)	36.3(13)	27.6(11)	6.5(9)	17.9(10)	5.7(10)
C16	60.7(17)	42.4(14)	28.1(12)	6.9(10)	22.5(12)	-1.7(12)
C17	71.4(19)	33.7(13)	17.2(10)	6.5(9)	7.5(11)	-6.6(12)
C18	97(3)	41.3(15)	27.7(13)	15.5(11)	9.5(14)	-9.9(15)
C19	56.5(16)	33.0(13)	29.7(12)	11.1(10)	-0.7(11)	7.0(11)
C20	39.9(13)	31.3(12)	25.3(11)	6.9(9)	2.4(9)	5.8(10)
O3	48(2)	43.5(19)	37.0(17)	21.0(14)	12.6(14)	-1.0(14)

Table S12. Bond Lengths for **1a-PdCycle-MePy**.

Atom	Atom	Length/Å	Atom	Atom	Length/Å	Atom	Atom	Length/Å
Pd1	O2	2.0107(16)	N2	C15	1.348(3)	C10	C11	1.387(3)
Pd1	N1	2.1502(17)	N2	C20	1.348(3)	C11	C12	1.504(3)
Pd1	N2	2.0264(19)	C1	C2	1.509(3)	C11	C13	1.393(3)
Pd1	C6	1.976(2)	C2	C3	1.398(3)	C13	C14	1.373(3)
F1	C1	1.341(3)	C2	C7	1.405(3)	C15	C16	1.377(4)
F2	C1	1.329(3)	C3	C4	1.389(3)	C16	C17	1.385(4)

Atom	Atom	Length/Å	Atom	Atom	Length/Å	Atom	Atom	Length/Å
F3	C1	1.350(3)	C4	C5	1.383(3)	C17	C18	1.501(4)
O1	C8	1.217(3)	C5	C6	1.403(3)	C17	C19	1.396(4)
O2	C8	1.299(3)	C6	C7	1.394(3)	C19	C20	1.381(4)
N1	C9	1.343(3)	C7	C8	1.515(3)			
N1	C14	1.345(3)	C9	C10	1.381(3)			

Table S13. Bond Angles for **1a-PdCycle-MePy**.

Atom	Atom	Atom	Angle/°	Atom	Atom	Atom	Angle/°
O2	Pd1	N1	91.08(7)	C4	C5	C6	120.2(2)
O2	Pd1	N2	175.68(6)	C5	C6	Pd1	127.68(17)
N2	Pd1	N1	93.08(7)	C7	C6	Pd1	113.28(15)
C6	Pd1	O2	82.02(8)	C7	C6	C5	119.0(2)
C6	Pd1	N1	172.45(8)	C2	C7	C8	124.9(2)
C6	Pd1	N2	93.88(8)	C6	C7	C2	120.7(2)
C8	O2	Pd1	116.37(13)	C6	C7	C8	114.36(19)
C9	N1	Pd1	119.59(15)	O1	C8	O2	122.2(2)
C9	N1	C14	117.14(19)	O1	C8	C7	124.0(2)
C14	N1	Pd1	123.16(15)	O2	C8	C7	113.70(19)
C15	N2	Pd1	120.96(17)	N1	C9	C10	122.6(2)
C15	N2	C20	117.9(2)	C9	C10	C11	120.3(2)
C20	N2	Pd1	121.13(17)	C10	C11	C12	121.8(2)
F1	C1	F3	104.71(19)	C10	C11	C13	116.8(2)
F1	C1	C2	113.5(2)	C13	C11	C12	121.3(2)
F2	C1	F1	107.48(19)	C14	C13	C11	119.7(2)
F2	C1	F3	106.2(2)	N1	C14	C13	123.4(2)
F2	C1	C2	113.30(19)	N2	C15	C16	122.5(2)
F3	C1	C2	111.03(19)	C15	C16	C17	120.4(3)
C3	C2	C1	117.2(2)	C16	C17	C18	121.8(3)
C3	C2	C7	119.3(2)	C16	C17	C19	117.0(2)
C7	C2	C1	123.5(2)	C19	C17	C18	121.2(3)
C4	C3	C2	119.8(2)	C20	C19	C17	120.1(3)
C5	C4	C3	120.8(2)	N2	C20	C19	122.2(3)

Table S14. Hydrogen Bonds for **1a-PdCycle-MePy**.

D	H	A	d(D-H)/Å	d(H-A)/Å	d(D-A)/Å	D-H-A/°
O3	H3A	O1*	0.87	1.93	2.795(3)	175.0
O3	H3B	O1	0.87	1.95	2.814(3)	170.8

* 1-X,2-Y,1-Z

Table S15. Torsion Angles for **1a-PdCycle-MePy**.

A	B	C	D	Angle/°	A	B	C	D	Angle/°
Pd1	O2	C8	O1	-175.03(19)	C3	C2	C7	C8	174.6(2)
Pd1	O2	C8	C7	2.7(2)	C3	C4	C5	C6	-1.9(4)
Pd1	N1	C9	C10	-176.90(19)	C4	C5	C6	Pd1	179.07(18)
Pd1	N1	C14	C13	176.85(19)	C4	C5	C6	C7	-1.2(4)
Pd1	N2	C15	C16	178.73(18)	C5	C6	C7	C2	3.9(3)
Pd1	N2	C20	C19	-179.54(17)	C5	C6	C7	C8	-174.2(2)
Pd1	C6	C7	C2	-176.40(17)	C6	C7	C8	O1	172.2(2)
Pd1	C6	C7	C8	5.5(3)	C6	C7	C8	O2	-5.4(3)
F1	C1	C2	C3	-111.3(2)	C7	C2	C3	C4	0.2(4)
F1	C1	C2	C7	69.8(3)	C9	N1	C14	C13	0.7(4)
F2	C1	C2	C3	125.8(2)	C9	C10	C11	C12	-178.3(2)
F2	C1	C2	C7	-53.2(3)	C9	C10	C11	C13	0.8(4)
F3	C1	C2	C3	6.4(3)	C10	C11	C13	C14	-0.7(4)
F3	C1	C2	C7	-172.6(2)	C11	C13	C14	N1	0.0(4)
N1	C9	C10	C11	-0.1(4)	C12	C11	C13	C14	178.4(2)
N2	C15	C16	C17	0.6(4)	C14	N1	C9	C10	-0.6(4)
C1	C2	C3	C4	-178.8(2)	C15	N2	C20	C19	-0.5(3)
C1	C2	C7	C6	175.6(2)	C15	C16	C17	C18	-179.4(2)
C1	C2	C7	C8	-6.5(4)	C15	C16	C17	C19	-0.1(4)
C2	C3	C4	C5	2.5(4)	C16	C17	C19	C20	-0.6(4)
C2	C7	C8	O1	-5.8(4)	C17	C19	C20	N2	1.0(4)
C2	C7	C8	O2	176.6(2)	C18	C17	C19	C20	178.7(2)
C3	C2	C7	C6	-3.3(3)	C20	N2	C15	C16	-0.3(3)

Table S16. Hydrogen Atom Coordinates (Å×10^4) and Isotropic Displacement Parameters (Å^2×10^3) for **1a-PdCycle-MePy**.

Atom	*x*	*y*	*z*	U(eq)
H3	5946.7	8102.52	10458.6	39
H4	4505.54	6138.29	10230.66	41
H5	3243.85	4777.29	8241.14	37
H9	3033.83	5398.88	2971.7	39
H10	1808.92	4620.84	796.78	41
H12A	-1926.13	2478.81	-879.22	68
H12B	-231.05	1813.23	-792.89	68
H12C	-204.25	3155.73	-1036.8	68
H13	-1206	1880.81	1345.28	43
H14	83.35	2726.55	3496.94	39
H15	-198.01	3729.61	5991.15	43
H16	-1015.7	1938.58	6479.62	52
H18A	1078.83	194.37	7768.95	89
H18B	1377.18	-558.78	6540.28	89
H18C	-550.85	-222.59	6435.16	89
H19	3693.78	958.45	6480.14	52
H20	4385.38	2754.93	5943.68	42
H3A	4014.44	9857.2	3712.39	63
H3B	3949.97	9019.99	4419.67	63

Table S17. Atomic Occupancy for **1a-PdCycle-MePy**.

Atom	*Occupancy*
O3	0.590(6)
H3A	0.590(6)
H3B	0.590(6)

8b. 4-*tert*-butylpyridine supported 5-membered palladacycle (1a-PdCycle-^{t}BuPy)

Data Collection:

A colorless crystal with approximate dimensions 0.14 × 0.13 × 0.04 mm^3 was selected under oil under ambient conditions and attached to the tip of a MiTeGen MicroMount©. The crystal was mounted in a stream of cold nitrogen at 100(1) K and centered in the X-ray beam by using a video camera. The crystal evaluation and data collection were performed on a Bruker D8 VENTURE PhotonIII four-circle diffractometer with Cu Kα (λ = 1.54178 Å) radiation and the detector to crystal distance of 4.5 cm.[15] The initial cell constants were obtained from a 180° φ scan conducted at a 2θ = 50° angle with an exposure time of 1 second per frame. The reflections were successfully indexed by an automated indexing routine built into the APEX3 program. The final cell constants were calculated from a set of 9997 strong reflections from the actual data collection. The data were collected by using a full sphere data collection routine to survey reciprocal space to the extent of a full sphere to a resolution of 0.79 Å. A total of 83081 data were harvested by collecting 38 sets of frames with 0.6–1.0º scans in ω and φ with exposure times of 1–5 sec per frame. These highly redundant datasets were corrected for Lorentz and polarization effects. The absorption correction was based on fitting a function to the empirical transmission surface as sampled by multiple equivalent measurements.[16]

Structure Solution and Refinement:

The systematic absences in the diffraction data were consistent for the space groups *Cc* and *C*2/*c*. The *E*-statistics strongly suggested the centrosymmetric space group *C*2/*c* that yielded chemically reasonable and computationally stable results of refinement.[17–22] A successful solution by intrinsic phasing provided most non-hydrogen atoms from the *E*-map. The remaining non-hydrogen atoms were located in an alternating series of least-squares cycles and difference Fourier maps. All non-hydrogen atoms were refined with anisotropic displacement coefficients. All hydrogen atoms were included in the structure factor calculation at idealized positions and were allowed to ride on the neighboring atoms with relative isotropic displacement coefficients. There are 0.308(3) molecules of solvent water per Pd complex in the lattice. The oxygen atom of the water molecule resides on a 2-fold crystallographic axis. The water molecule was refined with an idealized geometry.[23] The final least-squares refinement of 322 parameters against 5408 data resulted in residuals *R* (based on F^2 for $I \geq 2\sigma$) and *wR* (based on F^2 for all data) of 0.0204 and 0.0517. The final difference Fourier map was featureless.

Summary:

Crystal Data for $C_{26}H_{29.62125}F_3N_2O_{2.31}Pd$ (*M* =570.50 g/mol): monoclinic, space group C2/c (no. 15), *a* = 27.924(2) Å, *b* = 8.7423(9) Å, *c* = 20.8214(17) Å, *β* = 92.062(5)°, *V* = 5079.6(8) Å^3, *Z* = 8, *T* = 100.15 K, μ(Cu Kα) = 6.315 mm^{-1}, *Dcalc* = 1.492 g/cm^3, 83081 reflections measured (6.334° ≤ 2Θ ≤ 154.896°), 5408 unique (R_{int} = 0.0315, R_{sigma} = 0.0146) which were used in all calculations. The final R_1 was 0.0204 (I > 2σ(I)) and wR_2 was 0.0517 (all data).

Acknowledgement:

The purchase of the Bruker D8 VENTURE Photon III X-ray diffractometer was partially funded by NSF Award #CHE-1919350 to the UW–Madison Department of Chemistry.

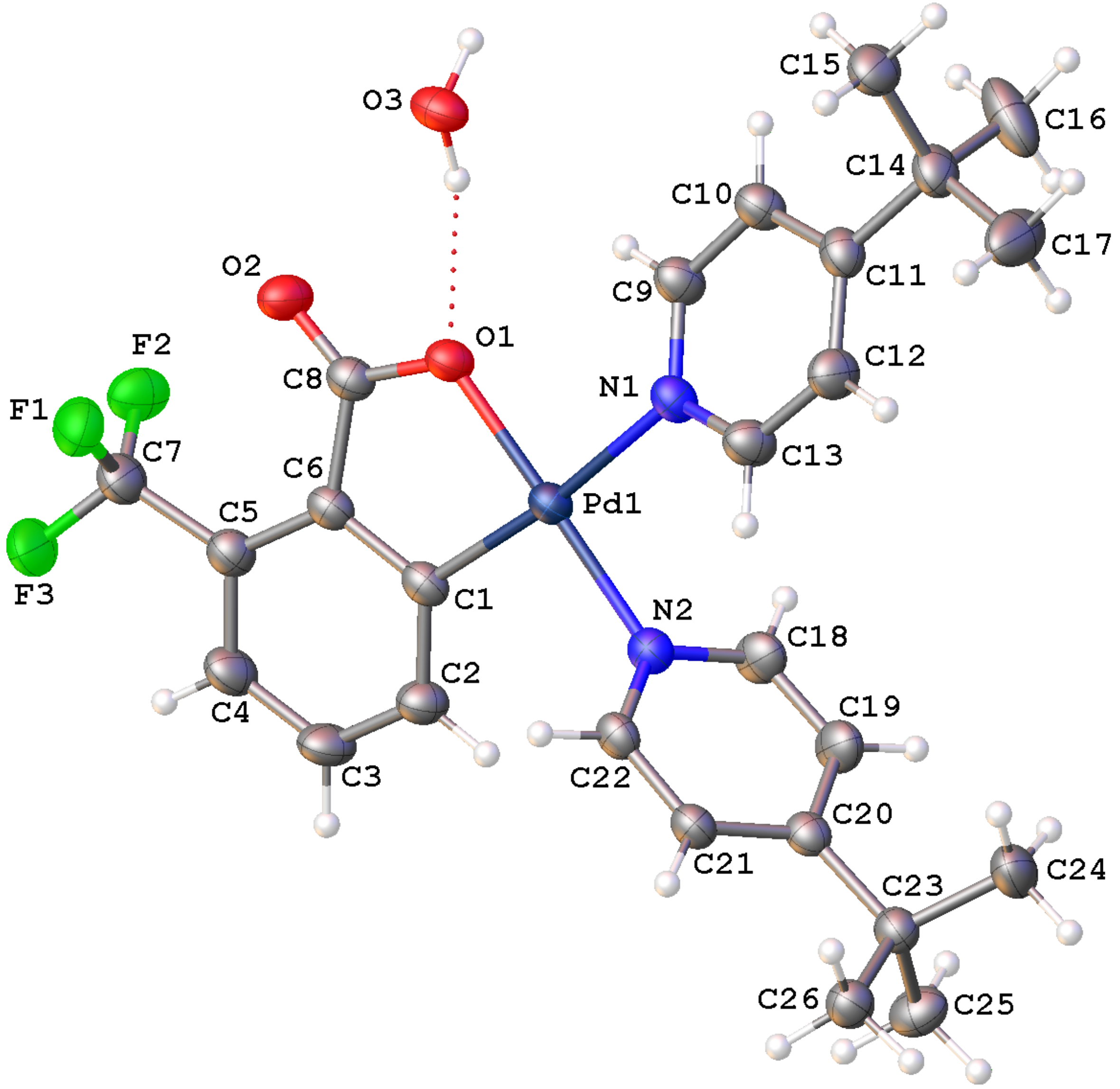

Figure S9. A molecular drawing of **1a-PdCycle-^{t}BuPy** shown with 50% probability ellipsoids. The solvent water molecule is present 30.8(3) % of the time.

Table S18. Crystal data and structure refinement for **1a-PdCycle-^{t}BuPy**.

Identification code	**1a-PdCycle-^{t}BuPy**
Empirical formula	$C_{26}H_{29}F_3N_2O_2Pd \cdot 0.31(H_2O)$
Formula weight	570.50
Temperature/K	100.15
Crystal system	monoclinic
Space group	C2/c
a/Å	27.924(2)
b/Å	8.7423(9)
c/Å	20.8214(17)
α/°	90
β/°	92.062(5)
γ/°	90
Volume/Å^3	5079.6(8)
Z	8
ρ_{calc}g/cm^3	1.492
μ/mm^{-1}	6.315
F(000)	2329.0
Crystal size/mm^3	0.14 × 0.13 × 0.04
Radiation	Cu Kα (λ = 1.54178)
2Θ range for data collection/°	6.334 to 154.896
Index ranges	-35 ≤ h ≤ 35, -10 ≤ k ≤ 11, -26 ≤ l ≤ 26
Reflections collected	83081
Independent reflections	5408 [Rint = 0.0315, Rsigma = 0.0146]
Data/restraints/parameters	5408/3/322
Goodness-of-fit on F^2	1.130
Final R indexes [I>=2σ (I)]	R1 = 0.0204, wR2 = 0.0516
Final R indexes [all data]	R1 = 0.0205, wR2 = 0.0517
Largest diff. peak/hole / e Å^{-3}	0.42/-0.25

Table S19. Fractional Atomic Coordinates (×10^4) and Equivalent Isotropic Displacement Parameters (Å^2×10^3) for **1a-PdCycle-^{t}BuPy**. U_{eq} is defined as 1/3 of the trace of the orthogonalized U_{IJ} tensor.

Atom	*x*	*y*	*z*	U(eq)
Pd1	4944.7(2)	2743.3(2)	4662.4(2)	25.60(5)
F1	3192.5(4)	4490.2(14)	3584.0(5)	47.1(3)
F2	3084.2(4)	2099.7(14)	3348.5(5)	46.0(3)
F3	2649.9(4)	3191.9(17)	4040.2(6)	55.1(3)
O1	4674.8(4)	3040.3(13)	3768.1(5)	30.1(2)
O2	4004.6(4)	3098.1(16)	3164.2(6)	38.0(3)
N1	5661.9(5)	3120.9(16)	4355.6(7)	29.6(3)
N2	5195.4(5)	2383.6(15)	5574.6(7)	28.0(3)
C1	4260.4(6)	2474.3(18)	4847.5(8)	27.9(3)
C2	4064.9(6)	2049(2)	5430.1(8)	33.9(4)
C3	3573.5(7)	1922(2)	5478.1(8)	39.9(4)
C4	3270.3(6)	2264(2)	4957.1(9)	38.8(4)
C5	3454.5(6)	2692.4(19)	4371.7(8)	32.4(3)
C6	3953.7(6)	2751.2(17)	4311.9(8)	27.9(3)
C7	3103.6(6)	3113(2)	3830.4(9)	39.3(4)
C8	4207.6(6)	3003.5(18)	3691.9(8)	28.9(3)
C9	5754.5(6)	2875(2)	3733.9(8)	33.3(4)
C10	6207.2(6)	2998(2)	3492.7(8)	33.8(4)
C11	6597.9(6)	3383(2)	3899.7(8)	32.4(3)
C12	6496.6(6)	3683(2)	4536.2(8)	37.0(4)
C13	6032.7(6)	3550(2)	4743.2(8)	35.4(4)
C14	7101.9(6)	3434(2)	3635.9(9)	37.6(4)
C15	7116.4(6)	4587(2)	3086.3(9)	41.4(4)
C16	7216.5(8)	1841(3)	3371.0(14)	63.6(7)
C17	7478.0(7)	3908(3)	4150.6(10)	59.7(6)
C18	5475.0(6)	1163.6(19)	5711.0(8)	35.7(4)
C19	5714.9(7)	982(2)	6294.3(8)	36.8(4)
C20	5683.6(6)	2094.1(18)	6774.3(8)	28.3(3)
C21	5387.5(5)	3336.6(19)	6628.0(8)	28.9(3)
C22	5150.0(5)	3440.8(18)	6036.4(8)	28.1(3)
C23	5961.8(6)	1923.0(19)	7413.4(8)	30.9(3)
C24	6491.8(6)	1616(3)	7285.2(9)	45.2(4)
C25	5751.6(7)	563(2)	7777.9(9)	40.2(4)
C26	5931.4(6)	3374(2)	7821.3(8)	33.2(3)
O3	5000	3842(3)	2500	37.9(9)

Table S20. Anisotropic Displacement Parameters ($Å^2 \times 10^3$) for **1a-PdCycle-^{t}BuPy**. The Anisotropic displacement factor exponent takes the form: $-2\pi^2[h^2a^{*2}U_{11}+2hka^*b^*U_{12}+\ldots]$.

Atom	U_{11}	U_{22}	U_{33}	U_{23}	U_{13}	U_{12}
Pd1	24.63(7)	25.76(7)	26.67(7)	-1.67(4)	4.52(4)	-1.05(4)
F1	42.1(6)	49.6(6)	49.6(6)	11.4(5)	1.7(5)	7.5(5)
F2	39.5(6)	61.1(7)	37.0(6)	-1.9(5)	-2.9(4)	-11.6(5)
F3	26.7(5)	88.7(9)	50.2(7)	11.6(6)	4.8(5)	6.5(6)
O1	27.7(5)	36.1(6)	26.9(5)	-1.2(5)	5.2(4)	-2.4(5)
O2	34.1(6)	51.2(7)	28.6(6)	1.1(5)	2.1(5)	-5.9(5)
N1	26.1(6)	29.8(7)	33.2(7)	-2.6(5)	4.7(5)	-1.7(5)
N2	28.9(7)	24.2(6)	31.0(7)	0.0(5)	3.6(5)	-1.3(5)
C1	27.5(8)	25.2(7)	31.4(8)	-3.4(6)	6.7(6)	-1.2(6)
C2	32.9(8)	40.0(9)	29.0(8)	0.5(7)	4.9(6)	-3.8(7)
C3	36.5(9)	54.1(11)	29.7(8)	1.3(8)	9.9(7)	-6.8(8)
C4	27.1(8)	51.8(11)	37.9(9)	-2.0(8)	7.8(7)	-4.8(7)
C5	29.7(8)	35.1(9)	32.7(8)	-2.5(7)	3.2(6)	-1.0(6)
C6	28.3(8)	26.2(8)	29.5(8)	-2.8(6)	5.9(6)	-0.9(6)
C7	27.4(8)	50.8(10)	39.8(9)	3.2(8)	5.6(7)	-0.3(7)
C8	29.5(8)	27.9(7)	29.6(8)	-1.3(6)	5.6(6)	-2.3(6)
C9	29.2(8)	37.0(9)	33.9(8)	-5.4(7)	3.6(7)	-4.1(6)
C10	31.7(8)	37.1(9)	33.0(8)	-4.2(7)	6.0(7)	-1.6(7)
C11	27.2(8)	31.6(8)	38.7(9)	3.7(7)	4.4(6)	0.4(6)
C12	29.0(8)	46.3(10)	35.7(9)	0.2(7)	-0.3(7)	-5.7(7)
C13	32.5(8)	42.0(9)	31.9(8)	-3.9(7)	3.1(6)	-5.4(7)
C14	25.3(8)	44.3(10)	43.6(9)	6.5(8)	5.9(7)	0.8(7)
C15	29.8(8)	53.5(11)	41.2(9)	5.7(8)	6.4(7)	-2.9(8)
C16	40.3(11)	47.4(12)	105(2)	3.7(13)	29.1(12)	9.6(9)
C17	27.5(9)	104(2)	47.4(11)	20.4(12)	0.4(8)	-9.8(11)
C18	45.5(10)	25.0(8)	36.6(9)	-5.0(7)	1.7(7)	5.3(7)
C19	44.2(10)	28.1(8)	38.1(9)	-1.3(7)	1.5(7)	9.5(7)
C20	27.1(7)	27.4(8)	30.8(8)	1.7(6)	5.8(6)	-1.3(6)
C21	27.8(7)	28.9(8)	30.3(8)	-2.6(6)	5.2(6)	1.4(6)
C22	27.8(7)	23.5(7)	33.4(8)	-2.1(6)	5.2(6)	2.6(6)
C23	29.5(8)	31.8(8)	31.5(8)	1.8(6)	4.0(6)	3.1(6)
C24	31.7(9)	62.8(13)	41.2(10)	-3.3(9)	1.7(7)	11.2(9)
C25	51.8(11)	34.6(9)	34.3(9)	6.0(7)	0.8(8)	-1.6(8)
C26	31.5(8)	35.7(9)	32.5(8)	-1.5(7)	0.3(6)	0.1(7)
O3	43.4(17)	36.8(16)	34.4(15)	0	15.5(12)	0

Atom	U_{11}	U_{22}	U_{33}	U_{23}	U_{13}	U_{12}
C19	56.5(16)	33.0(13)	29.7(12)	11.1(10)	-0.7(11)	7.0(11)
C20	39.9(13)	31.3(12)	25.3(11)	6.9(9)	2.4(9)	5.8(10)
O3	48(2)	43.5(19)	37.0(17)	21.0(14)	12.6(14)	-1.0(14)

Table S21. Bond Lengths for **1a-PdCycle-^{t}BuPy**.

Atom	Atom	Length/Å	Atom	Atom	Length/Å	Atom	Atom	Length/Å
Pd1	O1	2.0003(11)	C1	C2	1.398(2)	C14	C15	1.526(3)
Pd1	N1	2.1493(13)	C1	C6	1.403(2)	C14	C16	1.536(3)
Pd1	N2	2.0251(14)	C2	C3	1.384(2)	C14	C17	1.530(3)
Pd1	C1	1.9768(16)	C3	C4	1.385(3)	C18	C19	1.375(2)
F1	C7	1.336(2)	C4	C5	1.391(2)	C19	C20	1.399(2)
F2	C7	1.338(2)	C5	C6	1.405(2)	C20	C21	1.392(2)
F3	C7	1.357(2)	C5	C7	1.512(2)	C20	C23	1.524(2)
O1	C8	1.309(2)	C6	C8	1.511(2)	C21	C22	1.381(2)
O2	C8	1.221(2)	C9	C10	1.381(2)	C23	C24	1.537(2)
N1	C9	1.346(2)	C10	C11	1.398(2)	C23	C25	1.538(2)
N1	C13	1.343(2)	C11	C12	1.390(2)	C23	C26	1.530(2)
N2	C18	1.346(2)	C11	C14	1.530(2)			
N2	C22	1.343(2)	C12	C13	1.385(2)			

Table S22. Bond Angles for **1a-PdCycle-^{t}BuPy**.

Atom	Atom	Atom	Angle/°	Atom	Atom	Atom	Angle/°
O1	Pd1	N1	91.56(5)	F3	C7	C5	111.11(15)
O1	Pd1	N2	177.65(5)	O1	C8	C6	113.33(14)
N2	Pd1	N1	90.47(5)	O2	C8	O1	122.40(14)
C1	Pd1	O1	82.10(6)	O2	C8	C6	124.19(15)
C1	Pd1	N1	173.57(6)	N1	C9	C10	123.18(16)
C1	Pd1	N2	95.89(6)	C9	C10	C11	120.19(16)
C8	O1	Pd1	116.76(10)	C10	C11	C14	119.84(15)
C9	N1	Pd1	118.33(11)	C12	C11	C10	116.23(15)
C13	N1	Pd1	124.80(11)	C12	C11	C14	123.93(15)
C13	N1	C9	116.85(14)	C13	C12	C11	120.36(16)
C18	N2	Pd1	119.95(11)	N1	C13	C12	123.11(16)
C22	N2	Pd1	121.75(11)	C11	C14	C16	107.96(15)

Atom	Atom	Atom	Angle/°	Atom	Atom	Atom	Angle/°
C22	N2	C18	117.60(14)	C11	C14	C17	111.94(15)
C2	C1	Pd1	127.67(13)	C15	C14	C11	109.76(14)
C2	C1	C6	119.30(15)	C15	C14	C16	108.54(17)
C6	C1	Pd1	113.03(11)	C15	C14	C17	108.07(16)
C3	C2	C1	120.21(16)	C17	C14	C16	110.51(19)
C2	C3	C4	120.39(16)	N2	C18	C19	122.55(15)
C3	C4	C5	120.63(16)	C18	C19	C20	120.67(16)
C4	C5	C6	119.14(16)	C19	C20	C23	120.84(15)
C4	C5	C7	117.92(16)	C21	C20	C19	115.94(15)
C6	C5	C7	122.92(15)	C21	C20	C23	123.21(14)
C1	C6	C5	120.17(15)	C22	C21	C20	120.61(15)
C1	C6	C8	114.38(14)	N2	C22	C21	122.58(15)
C5	C6	C8	125.35(15)	C20	C23	C24	109.22(14)
F1	C7	F2	108.17(15)	C20	C23	C25	108.38(14)
F1	C7	F3	105.44(15)	C20	C23	C26	111.51(14)
F1	C7	C5	112.40(15)	C24	C23	C25	109.74(15)
F2	C7	F3	105.19(15)	C26	C23	C24	108.30(15)
F2	C7	C5	113.94(16)	C26	C23	C25	109.68(14)

Table S23. Hydrogen Bonds for **1a-PdCycle-^{t}BuPy**.

D	H	A	d(D-H)/Å	d(H-A)/Å	d(D-A)/Å	D-H-A/°
O3	H3A	O1	0.958(3)	2.062(9)	2.9087(13)	146.5(14)

Table S24. Torsion Angles for **1a-PdCycle-^{t}BuPy**.

A	B	C	D	Angle/°	A	B	C	D	Angle/°
Pd1	O1	C8	O2	-176.43(13)	C6	C5	C7	F3	-170.36(16)
Pd1	O1	C8	C6	0.54(17)	C7	C5	C6	C1	174.50(16)
Pd1	N1	C9	C10	-176.23(14)	C7	C5	C6	C8	-9.5(3)
Pd1	N1	C13	C12	175.56(14)	C9	N1	C13	C12	-2.6(3)
Pd1	N2	C18	C19	169.81(14)	C9	C10	C11	C12	-2.5(3)
Pd1	N2	C22	C21	-168.58(12)	C9	C10	C11	C14	176.79(16)
Pd1	C1	C2	C3	179.62(14)	C10	C11	C12	C13	2.0(3)
Pd1	C1	C6	C5	-176.45(12)	C10	C11	C14	C15	58.8(2)
Pd1	C1	C6	C8	7.09(17)	C10	C11	C14	C16	-59.3(2)

A	B	C	D	Angle/°	A	B	C	D	Angle/°
N1	C9	C10	C11	0.5(3)	C10	C11	C14	C17	178.82(18)
N2	C18	C19	C20	-1.1(3)	C11	C12	C13	N1	0.6(3)
C1	C2	C3	C4	-2.2(3)	C12	C11	C14	C15	-121.93(19)
C1	C6	C8	O1	-5.1(2)	C12	C11	C14	C16	119.9(2)
C1	C6	C8	O2	171.84(16)	C12	C11	C14	C17	-1.9(3)
C2	C1	C6	C5	3.9(2)	C13	N1	C9	C10	2.1(3)
C2	C1	C6	C8	-172.60(14)	C14	C11	C12	C13	-177.28(17)
C2	C3	C4	C5	2.1(3)	C18	N2	C22	C21	1.9(2)
C3	C4	C5	C6	1.0(3)	C18	C19	C20	C21	1.9(3)
C3	C4	C5	C7	-177.56(18)	C18	C19	C20	C23	-177.99(16)
C4	C5	C6	C1	-4.0(2)	C19	C20	C21	C22	-0.9(2)
C4	C5	C6	C8	172.06(16)	C19	C20	C23	C24	52.0(2)
C4	C5	C7	F1	126.03(17)	C19	C20	C23	C25	-67.5(2)
C4	C5	C7	F2	-110.47(18)	C19	C20	C23	C26	171.63(15)
C4	C5	C7	F3	8.1(2)	C20	C21	C22	N2	-1.0(2)
C5	C6	C8	O1	178.69(15)	C21	C20	C23	C24	-127.96(18)
C5	C6	C8	O2	-4.4(3)	C21	C20	C23	C25	112.52(17)
C6	C1	C2	C3	-0.7(3)	C21	C20	C23	C26	-8.3(2)
C6	C5	C7	F1	-52.5(2)	C22	N2	C18	C19	-0.8(3)
C6	C5	C7	F2	71.0(2)	C23	C20	C21	C22	178.99(15)

Table S25. Hydrogen Atom Coordinates (Å×10^4) and Isotropic Displacement Parameters (Å^2×10^3) for **1a-PdCycle-^{t}BuPy**.

Atom	*x*	*y*	*z*	U(eq)
H2	4270.02	1847.83	5794.15	41
H3	3443.41	1597.07	5870.86	48
H4	2933.16	2205.39	4999.73	47
H9	5495.95	2603.92	3447.34	40
H10	6253.17	2821.1	3049.03	41
H12	6746.97	3982.03	4830.87	44
H13	5973.91	3771.61	5179.98	42
H15A	6879.04	4301.41	2749.27	62
H15B	7436.67	4589.32	2908.82	62
H15C	7042.63	5609.74	3247.98	62
H16A	7226.96	1097.55	3723.55	95
H16B	7527.99	1866.05	3168.98	95

Atom	*x*	*y*	*z*	U(eq)
H16C	6967.58	1542.47	3051.49	95
H17A	7793.55	3958.27	3960.32	90
H17B	7485.78	3155.07	4499.43	90
H17C	7395.52	4915	4321.66	90
H18	5507.91	397.98	5391.84	43
H19	5903.78	93.39	6372.25	44
H21	5348.5	4119.86	6938	35
H22	4946.41	4293.91	5952.71	34
H24A	6616.59	2441.59	7020.32	68
H24B	6521.08	639.51	7058.75	68
H24C	6675.56	1570.97	7694.64	68
H25A	5937.77	401.39	8180.17	60
H25B	5765.61	-359.17	7511.92	60
H25C	5417.45	781.61	7874.05	60
H26A	6131.4	3252.99	8214.57	50
H26B	5598.13	3546.71	7934.42	50
H26C	6045.39	4249.98	7576.21	50
H3A	4868(12)	3207(8)	2824(9)	57

Table S26. Atomic Occupancy for **1a-PdCycle-^{t}BuPy**.

Atom	*Occupancy*
O3	0.616(6)
H3A	0.616(6)

8c. 4-methylpyridine supported 6-membered palladacycle (1b-PdCycle-MePy)

Data Collection:

A colorless crystal with approximate dimensions 0.14 × 0.12 × 0.12 mm^3 was selected under oil under ambient conditions and attached to the tip of a MiTeGen MicroMount©. The crystal was mounted in a stream of cold nitrogen at 100(1) K and centered in the X-ray beam by using a video camera. The crystal evaluation and data collection were performed on a Bruker D8 VENTURE PhotonIII four-circle diffractometer with Cu Kα (λ = 1.54178 Å) radiation and the detector to crystal distance of 5.0 cm.[15] The initial cell constants were obtained from a 180° φ scan conducted at a 2θ = 50° angle with an exposure time of 1 second per frame. The reflections were successfully indexed by an automated indexing routine built into the APEX3 program. The final cell constants were calculated from a set of 9708 strong reflections from the actual data collection. The data were collected by using a full sphere data collection routine to survey reciprocal space to the extent of a full sphere to a resolution of 0.78 Å. A total of 41946 data were harvested by collecting 10 sets of frames with 0.9º scans in ω and φ with exposure times of 1–5 sec per frame. These highly redundant datasets were corrected for Lorentz and polarization effects. The absorption correction was based on fitting a function to the empirical transmission surface as sampled by multiple equivalent measurements.[16]

Structure Solution and Refinement:

The systematic absences in the diffraction data were uniquely consistent for the space group *Pbca* that yielded chemically reasonable and computationally stable results of refinement.[17–22] A successful solution by intrinsic phasing provided most non-hydrogen atoms from the *E*-map. The remaining non-hydrogen atoms were located in an alternating series of least-squares cycles and difference Fourier maps. All non-hydrogen atoms were refined with anisotropic displacement coefficients. All hydrogen atoms were included in the structure factor calculation at idealized positions and were allowed to ride on the neighboring atoms with relative isotropic displacement coefficients. There is positional disorder in the complex – atoms O1, O2, C1, and C2 are disordered over two positions with the minor component contribution of 0.288(12) %. The disorder was refined with interatomic distance restraints and atomic displacement parameter constraints. In the crystal lattice there is a void occupied by solvent molecules. The solvent molecules share the same site which leads to compositional disorder. At least three different partially occupied solvent molecules could be identified: *t*-amyl alcohol, dichloromethane, and either water, or diethyl ether, or possibly a *t*-amyl alcohol in a second orientation. A significant amount of time was invested in identifying and refining the disordered molecules. Bond length restraints were applied to model the molecules, but the resulting isotropic displacement coefficients suggested the molecules were mobile. In addition, the refinement was computationally unstable. Option SQUEEZE of program PLATON was used to correct the diffraction data for diffuse scattering effects and to identify the solvate molecules.[24] PLATON calculated the upper limit of volume that can be occupied by the solvent to be 1420 Å^3, or 27.5 % of the unit cell volume. The program calculated 396 electrons in the unit cell for the diffuse species. This approximately corresponds to one molecule of *t*-amyl alcohol in the asymmetric unit (400 electrons); however, it was not possible to build a disorder model with *t*-amyl alcohol molecules only. The refinement without the solvent molecules yielded a better R factor and slightly lower s.u.'s on derived parameters, thus the model without solvents is reported. Please note that all derived results in the following tables are based on the known contents. No data are given for the diffusely scattering species. The final least-squares refinement of 277 parameters against 5523 data resulted in residuals R (based on F^2 for $I \geq 2\sigma$) and wR (based on F^2 for all data) of 0.0295 and 0.0677, respectively. The final difference Fourier map was featureless.

Summary:

Crystal Data for $C_{21}H_{19}F_3N_2O_2Pd$ (M =494.78 g/mol): orthorhombic, space group Pbca (no. 61), a = 9.4047(7) Å, b = 19.2839(11) Å, c = 28.4499(18) Å, V = 5159.7(6) Å^3, Z = 8, T = 100.00 K, μ(Cu Kα) = 6.132 mm^{-1}, $Dcalc$ = 1.274 g/cm^3, 41946 reflections measured (6.214° ≤ 2Θ ≤ 158.604°), 5523 unique (R_{int} = 0.0309, R_{sigma} = 0.0190) which were used in all calculations. The final R_1 was 0.0295 (I > 2σ(I)) and wR_2 was 0.0677 (all data).

Acknowledgement:

The purchase of the Bruker D8 VENTURE Photon III X-ray diffractometer was partially funded by NSF Award #CHE-1919350 to the UW–Madison Department of Chemistry.

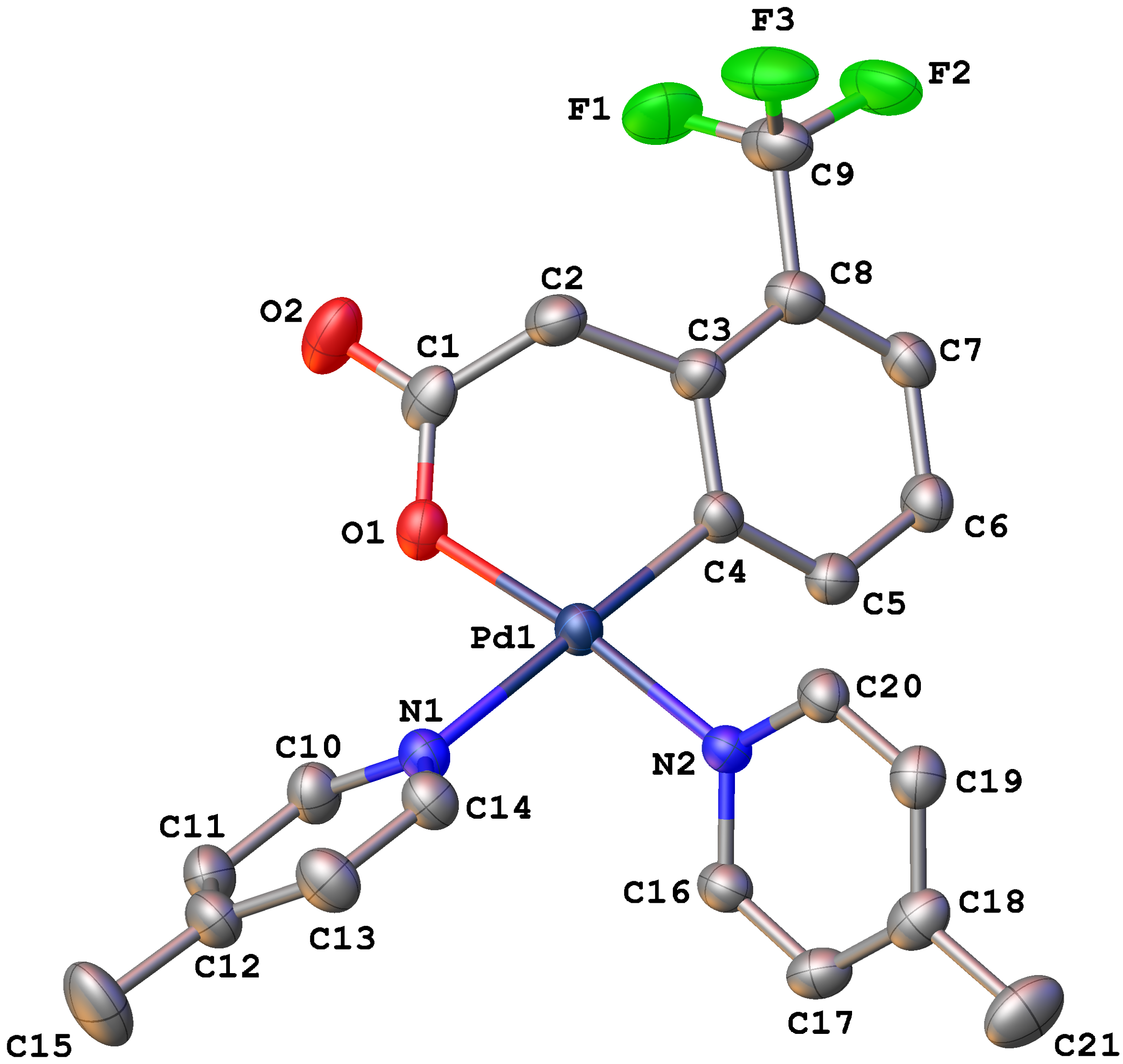

Figure S10. A molecular drawing of **1b-PdCycle-MePy** shown with 50% probability ellipsoids. All H atoms, minor disorder components, and solvent molecules are omitted.

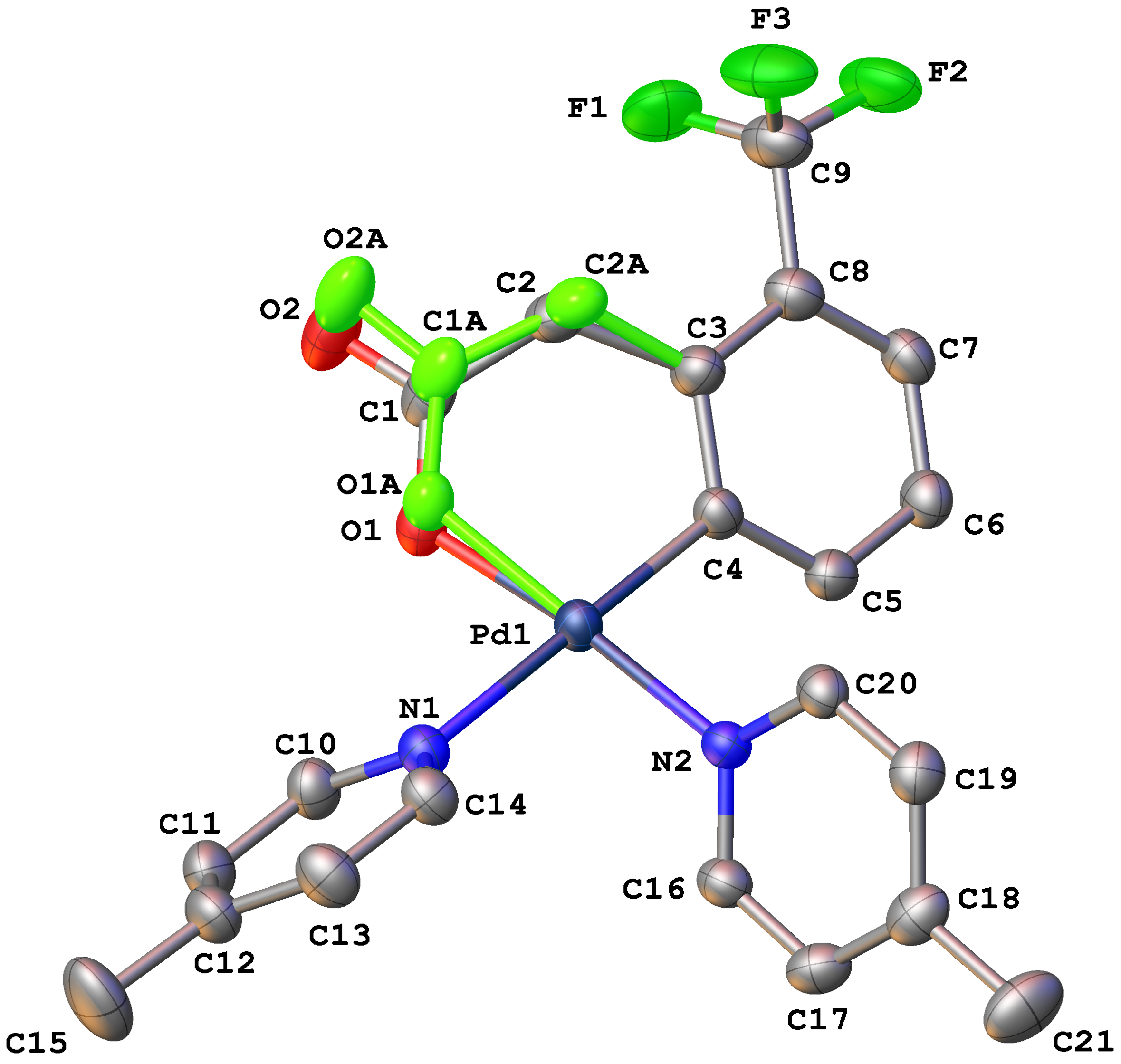

Figure S11. A molecular drawing of **1b-PdCycle-MePy** shown with 50% probability ellipsoids. The minor disorder components are shown in yellow-green. All H atoms and solvent molecules are omitted.

Table S27. Crystal data and structure refinement for **1b-PdCycle-MePy**.

Identification code	**1b-PdCycle-MePy**
Empirical formula	$C_{21}H_{19}F_3N_2O_2Pd$ · x(*t*-amyl alcohol and other solvents)
Formula weight	494.78
Temperature/K	100.00
Crystal system	orthorhombic
Space group	Pbca
a/Å	9.4047(7)
b/Å	19.2839(11)
c/Å	28.4499(18)
α/°	90
β/°	90
γ/°	90
Volume/Å^3	5159.7(6)
Z	8
ρ_{calc}g/cm^3	1.274
μ/mm^{-1}	6.132
F(000)	1984.0
Crystal size/mm^3	0.14 × 0.12 × 0.12
Radiation	Cu Kα (λ = 1.54178)
2Θ range for data collection/°	6.214 to 158.604
Index ranges	$-11 \leq h \leq 11$, $-24 \leq k \leq 24$, $-31 \leq l \leq 36$
Reflections collected	41946
Independent reflections	5523 [R_{int} = 0.0309, R_{sigma} = 0.0190]
Data/restraints/parameters	5523/10/277
Goodness-of-fit on F^2	1.154
Final R indexes [I>=2σ (I)]	R_1 = 0.0295, wR_2 = 0.0673
Final R indexes [all data]	R_1 = 0.0304, wR_2 = 0.0677
Largest diff. peak/hole / e Å^{-3}	0.51/-0.61

Table S28. Fractional Atomic Coordinates (×10^4) and Equivalent Isotropic Displacement Parameters (Å^2×10^3) for **1b-PdCycle-MePy**. U_{eq} is defined as 1/3 of the trace of the orthogonalized U_{IJ} tensor.

Atom	*x*	*y*	*z*	U(eq)
Pd1	6170.8(2)	3850.3(2)	4733.6(2)	27.10(6)
O1	6370(20)	4878(7)	4604(6)	32.2(10)
O2	6841(15)	5721(6)	4107(5)	51.5(13)
C1	6652(16)	5101(6)	4184(5)	34.1(12)
C2	6897(19)	4567(9)	3799(8)	35.5(12)
C3	5722(3)	4026.9(12)	3717.1(8)	31.9(5)
O1A	6630(8)	4828(3)	4536(2)	32.2(10)
O2A	7394(7)	5587(2)	4010.0(18)	51.5(13)
C1A	7041(6)	4996(3)	4120(2)	34.1(12)
C2A	7113(6)	4430(3)	3745(3)	35.5(12)
F1	5469(2)	4975.2(10)	2876.2(6)	63.7(5)
F2	4700.5(19)	4122.1(11)	2473.0(5)	58.6(5)
F3	6829.8(18)	4110.7(12)	2731.3(6)	62.6(5)
N1	7098(2)	4057.0(10)	5405.8(6)	28.6(4)
N2	5828.6(19)	2858.6(9)	4929.0(6)	26.1(4)
C4	5204(2)	3697.7(11)	4120.7(7)	27.8(4)
C5	3963(2)	3309.0(11)	4091.3(8)	29.7(4)
C6	3237(2)	3226.3(12)	3670.3(8)	33.1(5)
C7	3759(3)	3540.1(12)	3266.0(8)	33.5(5)
C8	4987(3)	3939.6(12)	3289.3(8)	33.5(5)
C9	5492(3)	4282.7(16)	2846.6(9)	44.2(6)
C10	6735(3)	4643.7(12)	5628.4(8)	34.3(5)
C11	7271(3)	4818.3(12)	6065.7(8)	36.7(5)
C12	8254(3)	4391.3(13)	6285.0(8)	38.2(5)
C13	8617(3)	3782.8(14)	6053.3(9)	39.3(5)
C14	8017(2)	3634.4(12)	5623.0(8)	32.4(5)
C15	8900(4)	4582.9(18)	6752.5(10)	63.1(9)
C16	5119(2)	2717.2(12)	5327.8(7)	30.1(4)
C17	4942(2)	2048.7(13)	5487.9(8)	34.1(5)
C18	5533(3)	1493.8(13)	5242.3(9)	36.2(5)
C19	6278(2)	1648.7(12)	4835.1(9)	33.5(5)
C20	6391(2)	2326.8(12)	4685.2(8)	29.6(4)
C21	5319(3)	759.9(15)	5406.7(12)	52.8(7)

Table S29. Anisotropic Displacement Parameters (Å^2×10^3) for **1b-PdCycle-MePy**. The Anisotropic displacement factor exponent takes the form: $-2\pi^2[h^2a^{*2}U_{11}+2hka^*b^*U_{12}+\ldots]$.

Atom	U_{11}	U_{22}	U_{33}	U_{23}	U_{13}	U_{12}
Pd1	29.71(9)	26.26(9)	25.32(9)	-0.42(5)	-3.19(6)	-2.09(6)
O1	35(3)	27.2(11)	34(2)	0.0(12)	-5.8(14)	-1.9(13)
O2	61(3)	36.3(17)	57(2)	13.1(15)	-19(2)	-15.2(19)
C1	26(3)	33.7(19)	43(2)	6.6(16)	-11.9(19)	-4.7(18)
C2	34(2)	41(3)	31(2)	5.7(18)	2.0(17)	-6(2)
C3	32.4(11)	35.6(11)	27.8(11)	2.8(9)	-2.0(9)	-5.7(9)
O1A	35(3)	27.2(11)	34(2)	0.0(12)	-5.8(14)	-1.9(13)
O2A	61(3)	36.3(17)	57(2)	13.1(15)	-19(2)	-15.2(19)
C1A	26(3)	33.7(19)	43(2)	6.6(16)	-11.9(19)	-4.7(18)
C2A	34(2)	41(3)	31(2)	5.7(18)	2.0(17)	-6(2)
F1	89.0(14)	57.5(10)	44.5(9)	20.5(8)	-0.2(9)	-14.4(10)
F2	57.3(10)	92.5(13)	26.2(7)	9.4(8)	-4.8(7)	-16.4(10)
F3	42.2(9)	104.0(15)	41.5(9)	16.1(9)	11.5(7)	-4.0(9)
N1	29.1(9)	29.7(9)	26.9(9)	-1.7(7)	-1.6(7)	-0.6(7)
N2	28.0(9)	27.8(9)	22.5(8)	0.0(7)	-0.1(7)	-1.7(7)
C4	31.3(11)	26.8(10)	25.2(10)	-0.3(8)	-2.7(8)	-0.8(8)
C5	31.7(11)	29.5(11)	27.9(10)	-0.1(8)	1.9(8)	-1.3(9)
C6	29.9(11)	36.0(12)	33.5(11)	-3.6(9)	-2.6(9)	-3.9(9)
C7	35.8(12)	37.8(12)	27.0(11)	-4.3(9)	-4.0(9)	-0.8(10)
C8	33.5(11)	38.2(12)	28.9(11)	1.5(9)	0.4(9)	1.2(10)
C9	40.2(14)	61.6(17)	30.8(12)	5.7(11)	-1.2(10)	-6.7(12)
C10	38.8(12)	29.2(11)	34.8(12)	-1.9(9)	-4.0(10)	1.4(9)
C11	43.6(13)	31.5(11)	35.1(12)	-6.8(9)	0.9(10)	-2.2(10)
C12	43.5(13)	42.8(13)	28.1(11)	-4.1(10)	-2.9(10)	-5.2(11)
C13	38.9(13)	46.0(14)	33.1(12)	-1.7(10)	-7.5(10)	5.7(11)
C14	33.2(11)	33.8(11)	30.1(11)	-2.8(9)	-1.8(9)	4.1(9)
C15	85(2)	65(2)	39.1(15)	-14.3(14)	-22.1(15)	5.8(18)
C16	29.6(11)	35.5(11)	25.1(10)	-1.1(8)	1.1(8)	-1.2(9)
C17	29.8(11)	40.6(12)	31.7(11)	8.8(9)	1.1(9)	-0.3(10)
C18	27.6(11)	37.7(12)	43.3(13)	8.6(10)	-4.8(9)	-2.0(9)
C19	28.5(11)	32.0(11)	40.1(12)	-2.6(9)	-1.8(9)	2.2(9)
C20	26.8(10)	33.2(11)	28.9(10)	-3.5(8)	0.4(8)	-2.3(9)
C21	47.8(16)	38.5(14)	72(2)	17.9(13)	6.4(14)	3.9(12)

Table S30. Bond Lengths for **1b-PdCycle-MePy**.

Atom	Atom	Length/Å	Atom	Atom	Length/Å	Atom	Atom	Length/Å
Pd1	O1	2.025(12)	O2A	C1A	1.229(5)	C8	C9	1.500(3)
Pd1	O1A	2.015(5)	C1A	C2A	1.529(6)	C10	C11	1.384(3)
Pd1	N1	2.1394(18)	F1	C9	1.338(3)	C11	C12	1.386(4)
Pd1	N2	2.0173(18)	F2	C9	1.334(3)	C12	C13	1.389(4)
Pd1	C4	1.988(2)	F3	C9	1.342(3)	C12	C15	1.508(3)
O1	C1	1.298(13)	N1	C10	1.341(3)	C13	C14	1.378(3)
O2	C1	1.228(12)	N1	C14	1.339(3)	C16	C17	1.377(3)
C1	C2	1.519(13)	N2	C16	1.344(3)	C17	C18	1.394(4)
C2	C3	1.537(13)	N2	C20	1.346(3)	C18	C19	1.387(3)
C3	C2A	1.523(6)	C4	C5	1.390(3)	C18	C21	1.504(4)
C3	C4	1.399(3)	C5	C6	1.388(3)	C19	C20	1.379(3)
C3	C8	1.410(3)	C6	C7	1.390(3)			
O1A	C1A	1.284(5)	C7	C8	1.389(3)			

Table S31. Bond Angles for **1b-PdCycle-MePy**.

Atom	Atom	Atom	Angle/°	Atom	Atom	Atom	Angle/°
O1	Pd1	N1	86.8(6)	C3	C4	Pd1	119.57(16)
O1A	Pd1	N1	89.3(2)	C5	C4	Pd1	121.11(16)
O1A	Pd1	N2	176.8(2)	C5	C4	C3	119.2(2)
N2	Pd1	O1	173.1(4)	C6	C5	C4	121.8(2)
N2	Pd1	N1	89.73(7)	C5	C6	C7	119.4(2)
C4	Pd1	O1	91.5(6)	C8	C7	C6	119.7(2)
C4	Pd1	O1A	89.5(2)	C3	C8	C9	121.1(2)
C4	Pd1	N1	176.28(8)	C7	C8	C3	121.0(2)
C4	Pd1	N2	91.63(8)	C7	C8	C9	117.9(2)
C1	O1	Pd1	120.7(11)	F1	C9	F3	106.1(2)
O1	C1	C2	118.1(13)	F1	C9	C8	112.5(2)
O2	C1	O1	121.0(13)	F2	C9	F1	105.9(2)
O2	C1	C2	120.6(13)	F2	C9	F3	105.7(2)
C1	C2	C3	117.4(12)	F2	C9	C8	113.0(2)
C4	C3	C2	115.6(9)	F3	C9	C8	113.1(2)
C4	C3	C2A	119.2(4)	N1	C10	C11	122.5(2)
C4	C3	C8	118.9(2)	C10	C11	C12	120.2(2)
C8	C3	C2	124.4(9)	C11	C12	C13	116.9(2)

Atom	Atom	Atom	Angle/°	Atom	Atom	Atom	Angle/°
C8	C3	C2A	121.8(4)	C11	C12	C15	121.3(2)
C1A	O1A	Pd1	123.9(4)	C13	C12	C15	121.8(2)
O1A	C1A	C2A	118.4(5)	C14	C13	C12	119.8(2)
O2A	C1A	O1A	123.4(5)	N1	C14	C13	123.2(2)
O2A	C1A	C2A	118.2(5)	N2	C16	C17	122.0(2)
C3	C2A	C1A	111.3(4)	C16	C17	C18	120.3(2)
C10	N1	Pd1	118.40(15)	C17	C18	C21	120.9(2)
C14	N1	Pd1	124.21(15)	C19	C18	C17	117.1(2)
C14	N1	C10	117.40(19)	C19	C18	C21	122.0(2)
C16	N2	Pd1	120.27(15)	C20	C19	C18	120.1(2)
C16	N2	C20	118.37(19)	N2	C20	C19	122.2(2)
C20	N2	Pd1	121.17(15)				

Table S32. Torsion Angles for **1b-PdCycle-MePy**.

A	B	C	D	Angle/°	A	B	C	D	Angle/°
Pd1	O1	C1	O2	-176.4(12)	N2	C16	C17	C18	1.6(4)
Pd1	O1	C1	C2	-3(2)	C4	C3	C2A	C1A	-57.2(6)
Pd1	O1A	C1A	O2A	-175.8(4)	C4	C3	C8	C7	0.6(4)
Pd1	O1A	C1A	C2A	3.4(8)	C4	C3	C8	C9	179.8(2)
Pd1	N1	C10	C11	179.49(19)	C4	C5	C6	C7	0.0(4)
Pd1	N1	C14	C13	179.03(19)	C5	C6	C7	C8	-0.9(4)
Pd1	N2	C16	C17	-175.88(17)	C6	C7	C8	C3	0.6(4)
Pd1	N2	C20	C19	174.09(17)	C6	C7	C8	C9	-178.5(2)
Pd1	C4	C5	C6	176.73(17)	C7	C8	C9	F1	116.5(3)
O1	C1	C2	C3	55(2)	C7	C8	C9	F2	-3.3(4)
O2	C1	C2	C3	-131.1(17)	C7	C8	C9	F3	-123.3(3)
C1	C2	C3	C4	-48.1(19)	C8	C3	C2A	C1A	126.9(5)
C1	C2	C3	C8	120.1(14)	C8	C3	C4	Pd1	-177.07(17)
C2	C3	C4	Pd1	-8.2(9)	C8	C3	C4	C5	-1.6(3)
C2	C3	C4	C5	167.3(8)	C10	N1	C14	C13	-1.5(4)
C2	C3	C8	C7	-167.2(8)	C10	C11	C12	C13	-2.2(4)
C2	C3	C8	C9	11.9(9)	C10	C11	C12	C15	177.5(3)
C3	C4	C5	C6	1.3(3)	C11	C12	C13	C14	0.8(4)
C3	C8	C9	F1	-62.6(3)	C12	C13	C14	N1	1.1(4)
C3	C8	C9	F2	177.6(2)	C14	N1	C10	C11	0.0(3)

A	B	C	D	Angle/°	A	B	C	D	Angle/°
C3	C8	C9	F3	57.5(3)	C15	C12	C13	C14	-179.0(3)
O1A	C1A	C2A	C3	51.4(8)	C16	N2	C20	C19	-1.0(3)
O2A	C1A	C2A	C3	-129.3(5)	C16	C17	C18	C19	-0.6(3)
C2A	C3	C4	Pd1	6.9(4)	C16	C17	C18	C21	-178.6(2)
C2A	C3	C4	C5	-177.6(3)	C17	C18	C19	C20	-1.0(3)
C2A	C3	C8	C7	176.6(4)	C18	C19	C20	N2	1.9(3)
C2A	C3	C8	C9	-4.3(5)	C20	N2	C16	C17	-0.8(3)
N1	C10	C11	C12	1.9(4)	C21	C18	C19	C20	176.9(2)

Table S33. Hydrogen Atom Coordinates (Å×10^4) and Isotropic Displacement Parameters (Å^2×10^3) for **1b-PdCycle-MePy**.

Atom	*x*	*y*	*z*	U(eq)
H2A	7787.41	4315.82	3872.85	43
H2B	7052.84	4819.13	3500.64	43
H2AA	7316.04	4643.15	3435.84	43
H2AB	7898.7	4106.56	3820.08	43
H5	3602.07	3093.84	4366.92	36
H6	2390.6	2957.76	3658.67	40
H7	3279.37	3481.74	2974.81	40
H10	6084.36	4951.1	5480.49	41
H11	6965.2	5232.05	6216.04	44
H13	9277.24	3469.88	6190.86	47
H14	8267.13	3211.46	5473.07	39
H15A	8616.74	5056.07	6836.26	95
H15B	9938.33	4557.03	6730.74	95
H15C	8563.74	4259.98	6994.17	95
H16	4729.11	3089.35	5504.5	36
H17	4414.28	1965.56	5767	41
H19	6712.48	1287.49	4659.15	40
H20	6880.64	2421.7	4400.37	36
H21A	5405.57	741.2	5749.73	79
H21B	6041.92	460.29	5264.02	79
H21C	4371.26	600.06	5313.43	79

Table S34. Atomic Occupancy for **1b-PdCycle-MePy**.

Atom	*Occupancy*	Atom	*Occupancy*	Atom	*Occupancy*	Atom	*Occupancy*
O1	0.288(12)	C2A	0.712(12)	O2A	0.712(12)	H2B	0.288(12)
C2	0.288(12)	O2	0.288(12)	H2AA	0.712(12)	C1A	0.712(12)
O1A	0.712(12)	H2A	0.288(12)	C1	0.288(12)	H2AB	0.712(12)

9. References

1. Bruker VT-NMR calibration manual:
For temperatures from ~ 300 K to 380 K:
100 % ethylene glycol: T (K) = (4.218 − Δδ) / 0.009132
80% ethylene glycol in DMSO-d_6: T (K) = (4.637 − Δδ) / 0.009967
Δδ is the difference in chemical shift (ppm) between the –CH_2 and –OH peaks.
For temperatures from 270 K to 300 K:
4 % methanol in MeOD-d_4: T (K) = (4.109 − Δδ) / 0.008708
Δδ is the difference in chemical shift (ppm) between the –CH_3 and –OH peaks.

2. Salazar, C. A.; Gair, J. J.; Flesch, K. N.; Guzei, I. A.; Lewis, J. C.; Stahl, S. S. Catalytic Behavior of Mono-*N*-Protected Amino-Acid Ligands in Ligand-Accelerated C−H Activation by Palladium(II). *Angew. Chem. Int. Ed.* **2020**, *59*, 10873–10877.

3. Salazar, C. A.; Flesch, K. N.; Haines, B. E.; Zhou, P. S.; Musaev, D. G.; Stahl, S. S. Tailored quinones support high-turnover Pd catalysts for oxidative C–H arylation with O_2. *Science* **2020**, *370*, 1454–1460.

4. Frisch, M. J.; Trucks, G. W.; Schlegel, H. B.; Scuseria, G. E.; Robb, M. A.; Cheeseman, J. R.; Scalmani, G.; Barone, V.; Petersson, G. A.; Nakatsuji, H.; Li, X.; Caricato, M.; Marenich, A. V.; Bloino, J.; Janesko, B. G.; Gomperts, R.; Mennucci, B.; Hratchian, H. P.; Ortiz, J. V.; Izmaylov, A. F.; Sonnenberg, J. L.; Williams-Young, D.; Ding, F.; Lipparini, F.; Egidi, F.; Goings, J.; Peng, B.; Petrone, A.; Henderson, T.; Ranasinghe, D.; Zakrzewski, V. G.; Gao, J.; Rega, N.; Zheng, G.; Liang, W.; Hada, M.; Ehara, M.; Toyota, K.; Fukuda, R.; Hasegawa, J.; Ishida, M.; Nakajima, T.; Honda, Y.; Kitao, O.; Nakai, H.; Vreven, T.; Throssell, K.; Montgomery, J. A., Jr.; Peralta, J. E.; Ogliaro, F.; Bearpark, M. J.; Heyd, J. J.; Brothers, E. N.; Kudin, K. N.; Staroverov, V. N.; Keith, T. A.; Kobayashi, R.; Normand, J.; Raghavachari, K.; Rendell, A. P.; Burant, J. C.; Iyengar, S. S.; Tomasi, J.; Cossi, M.; Millam, J. M.; Klene, M.; Adamo, C.; Cammi, R.; Ochterski, J. W.; Martin, R. L.; Morokuma, K.; Farkas, O.; Foresman, J. B.; Fox, D. J., *Gaussian 16, Revision C.01*, **Gaussian**, Inc., Wallingford CT, 2019.

5. Becke, A. D. Density-Functional Exchange-Energy Approximation with Correct Asymptotic Behavior. *Phys. Rev. A* **1988**, *38*, 3098–3100.

6. Lee, C.; Yang, W.; Parr, R. G. Development of The Colle-Salvetti Correlation-Energy Formula into a Functional of the Electron Density. *Phys. Rev. B* **1988**, *37*, 785–789.

7. Becke, A. D. A New Mixing of Hartree–Fock and Local Density-Functional Theories. *J. Chem. Phys.* **1993**, *98*, 1372–1377.

8. Grimme, S.; Antony, J.; Ehrlich, S.; Krieg, H. A Consistent and Accurate Ab Initio Parametrization of Density Functional Dispersion Correction (DFT-D) for the 94 Elements H-Pu. *J. Chem. Phys.* **2010**, *132*, 154104–154122.

9. Becke, A. D.; Johnson, E. R. A Density-Functional Model of the Dispersion Interaction. *J. Chem. Phys.* **2005**, *123*, 154101–154106.

10. Becke, A. D.; Johnson, E. R. Exchange-Hole Dipole Moment and the Dispersion Interaction. *J. Chem. Phys.* **2005**, *122*, 154104–154109.

11. Johnson, E. R.; Becke, A. D. A Post-Hartree-Fock Model of Intermolecular Interactions: Inclusion of Higher-Order Corrections. *J. Chem. Phys.* **2006**, *124*, 174104–174112.

12. Hay, P. J.; Wadt, W. R. Ab initio effective core potentials for molecular calculations. Potentials for the transition metal atoms Sc to Hg. *J. Chem. Phys.* **1985**, *82*, 270–283.

13. Wadt, W. R.; Hay, P. J. Ab initio effective core potentials for molecular calculations. Potentials for main group elements Na to Bi. *J. Chem. Phys.* **1985**, *82*, 284–298.

14. Marenich, A. V.; Cramer, C. J.; Truhlar, D. G. Universal Solvation Model Based on Solute Electron Density and on a Continuum Model of the Solvent Defined by the Bulk Dielectric Constant and Atomic Surface Tensions. *J. Phys. Chem. B* **2009**, *113*, 6378–6396.

15. Bruker-AXS (2019). APEX3. Version 2019.11-0. Madison, Wisconsin, USA.

16. Krause, L.; Herbst-Irmer, R.; Sheldrick, G. M.; Stalke, D. Comparison of silver and molybdenum microfocus X-ray sources for single-crystal structure determination. *J. Appl. Cryst.* **2015**, *48*, 3–10.

17. Sheldrick, G. M. (2013b). XPREP. Version 2013/1. Georg-August-Universität Göttingen, Göttingen, Germany.

18. Sheldrick, G. M. (2013a). The SHELX homepage, http://shelx.uni-ac.gwdg.de/SHELX/.

19. Sheldrick, G. M. *SHELXT* – Integrated space-group and crystal-structure determination. *Acta Cryst.* **2015**, *A71*, 3–8.

20. Sheldrick, G. M. Crystal structure refinement with *SHELXL*. *Acta Cryst.* **2015**, *C71*, 3–8.

21. Dolomanov, O. V.; Bourhis, L. J.; Gildea, R. J.; Howard, J. A. K.; Puschmann, H. *OLEX2*: a complete structure solution, refinement and analysis program. *J. Appl. Cryst.* **2009**, *42*, 339–341.

22. Guzei, I. A. (2007–2013). Programs Gn. University of Wisconsin–Madison, Madison, Wisconsin, USA.

23. Guzei, I. A. An idealized molecular geometry library for refinement of poorly behaved molecular fragments with constraints. *J. Appl. Cryst.* **2014**, *47*, 806–809.

24. Marenich, A. V.; Cramer, C. J.; Truhlar, D. G. Universal Solvation Model Based on Solute Electron Density and on a Continuum Model of the Solvent Defined by the Bulk Dielectric Constant and Atomic Surface Tensions. *J. Phys. Chem. B* **2009**, *113*, 6378–6396.

10. NMR spectra of compounds

10a. Benzoate and phenylacetate substrates (^{1}H, ^{13}Cs{^{1}H}, and ^{19}F NMR)

(*Pages S75 to S98*)

10b. MPAA ligands (^{1}H and ^{13}C{^{1}H} NMR)

(*Pages S99 to S118*)

10c. Pyridine-supported palladacycles (^{1}H, ^{13}C{^{1}H} and ^{19}F NMR)

(*Pages S119 to S131*)

10d. Benzoic and phenylacetic acid arylation products (^{1}H, ^{13}C{^{1}H} and ^{19}F NMR)

(*Pages S132 to S161*)

Potassium 2-(trifluoromethyl)benzoate (**1a**)

^{1}H NMR (600 MHz, D_2O) δ 7.63 (d, J = 7.9 Hz, 1H), 7.54 (d, J = 7.6 Hz, 1H), 7.43 (t, J = 7.8 Hz, 1H), 7.35 (d, J = 7.7 Hz, 1H).

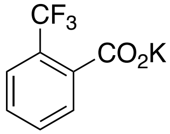

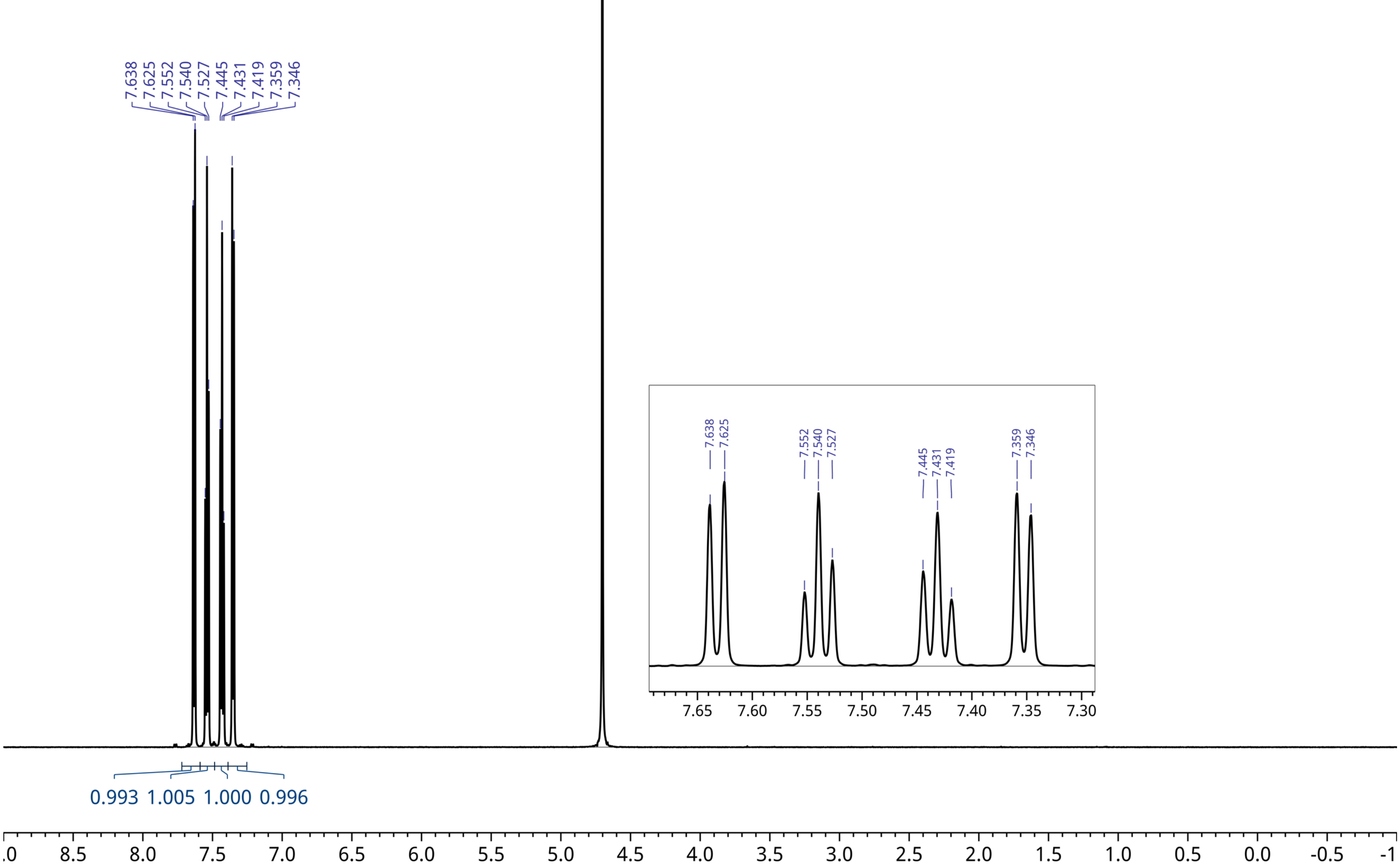

Potassium 2-(trifluoromethyl)benzoate (**1a**)

$^{13}C\{^{1}H\}$ NMR (151 MHz, D_2O) δ 176.86, 138.57 (q, J = 2.6 Hz), 132.23, 128.21, 126.57, 125.89 (q, J = 4.8 Hz), 124.52 (q, J = 31.5 Hz), 124.02 (q, J = 272.6 Hz).

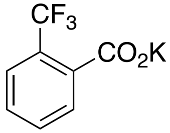

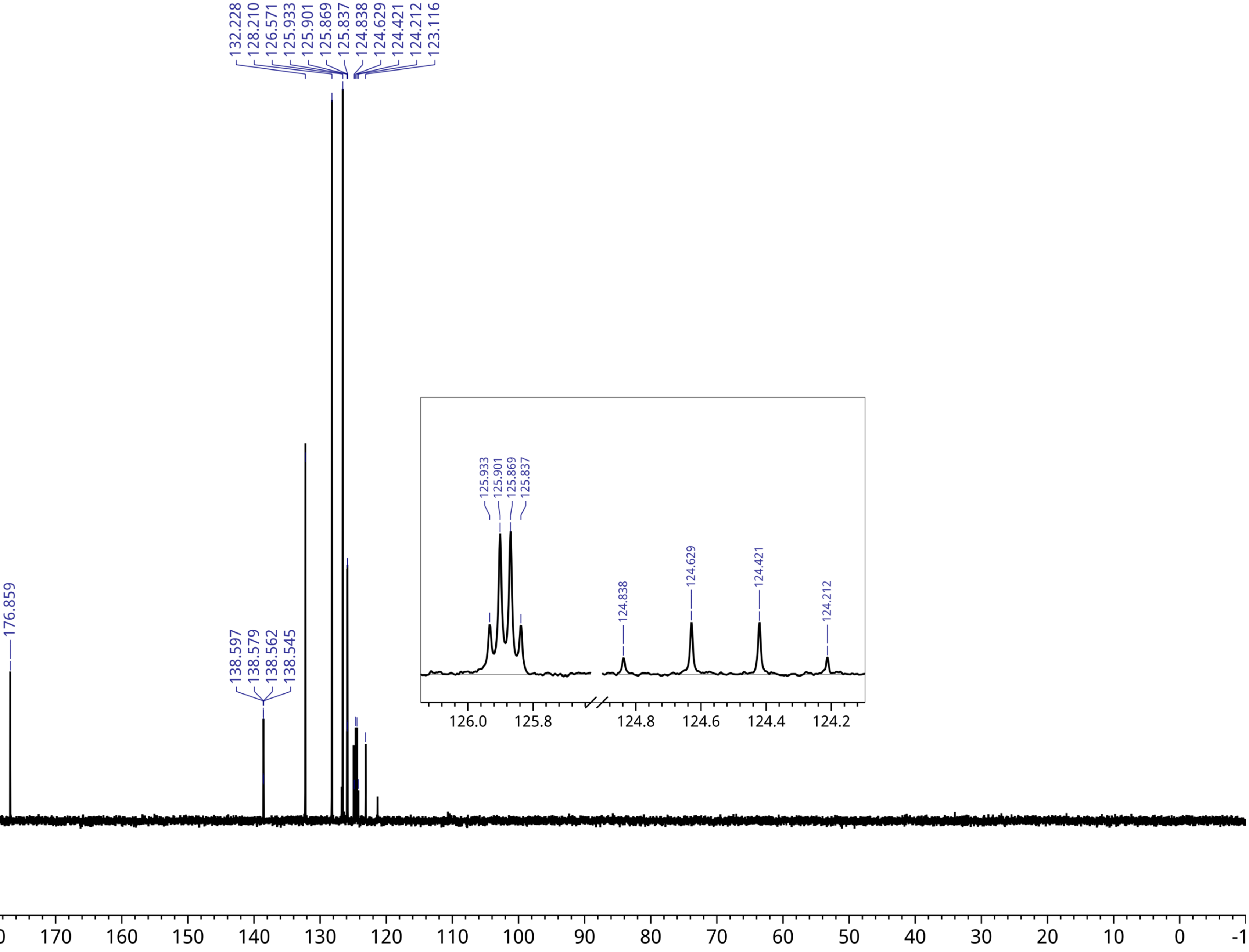

Potassium 2-(trifluoromethyl)benzoate (**1a**)

^{19}F NMR (564 MHz, D_2O) δ –59.68.

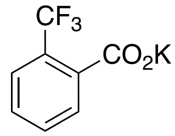

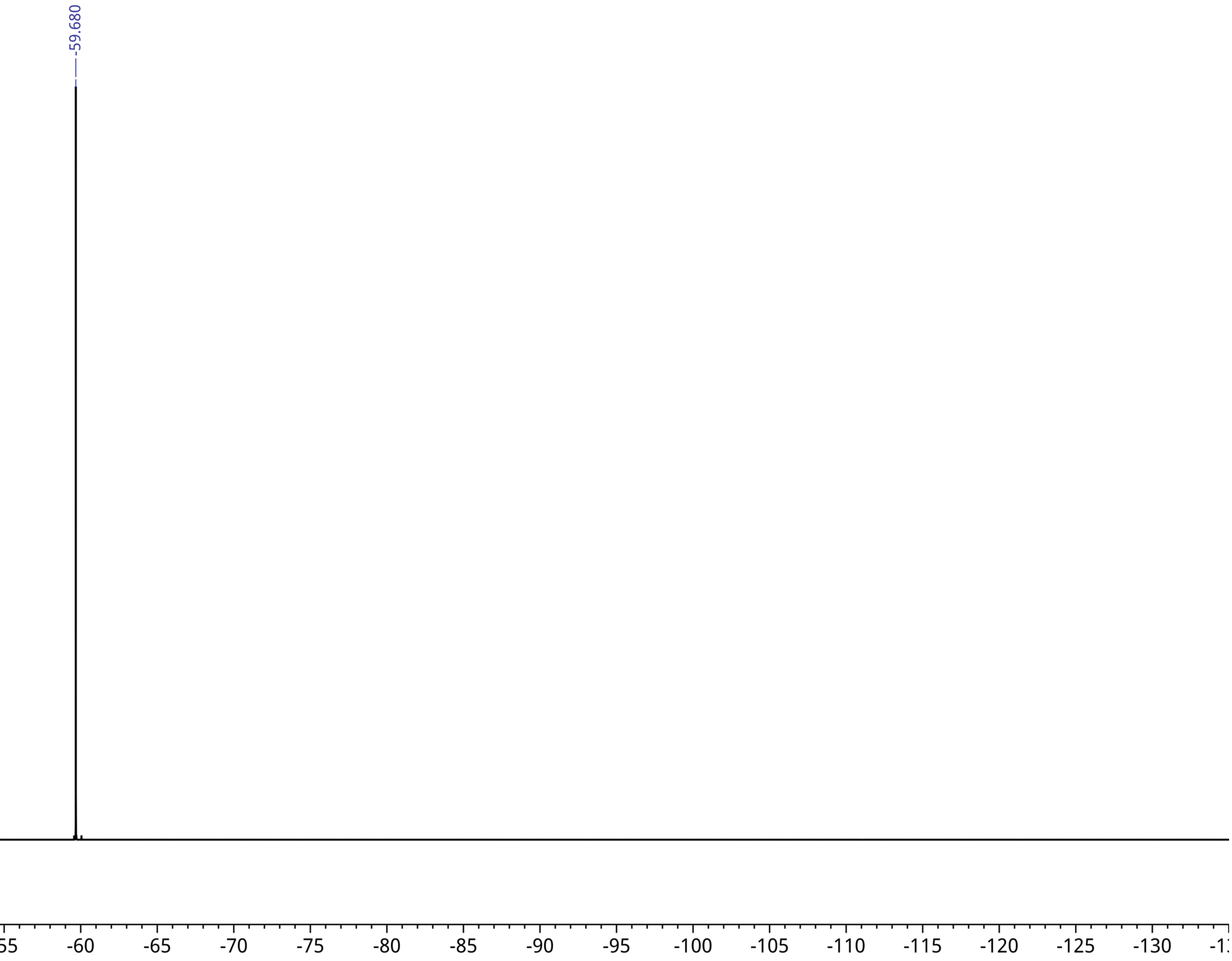

Potassium 4-fluoro-2-(trifluoromethyl)benzoate (**2a**)

^{1}H NMR (600 MHz, D_2O) δ 7.43 – 7.37 (m, 2H), 7.28 (td, J = 8.5, 2.6 Hz, 1H).

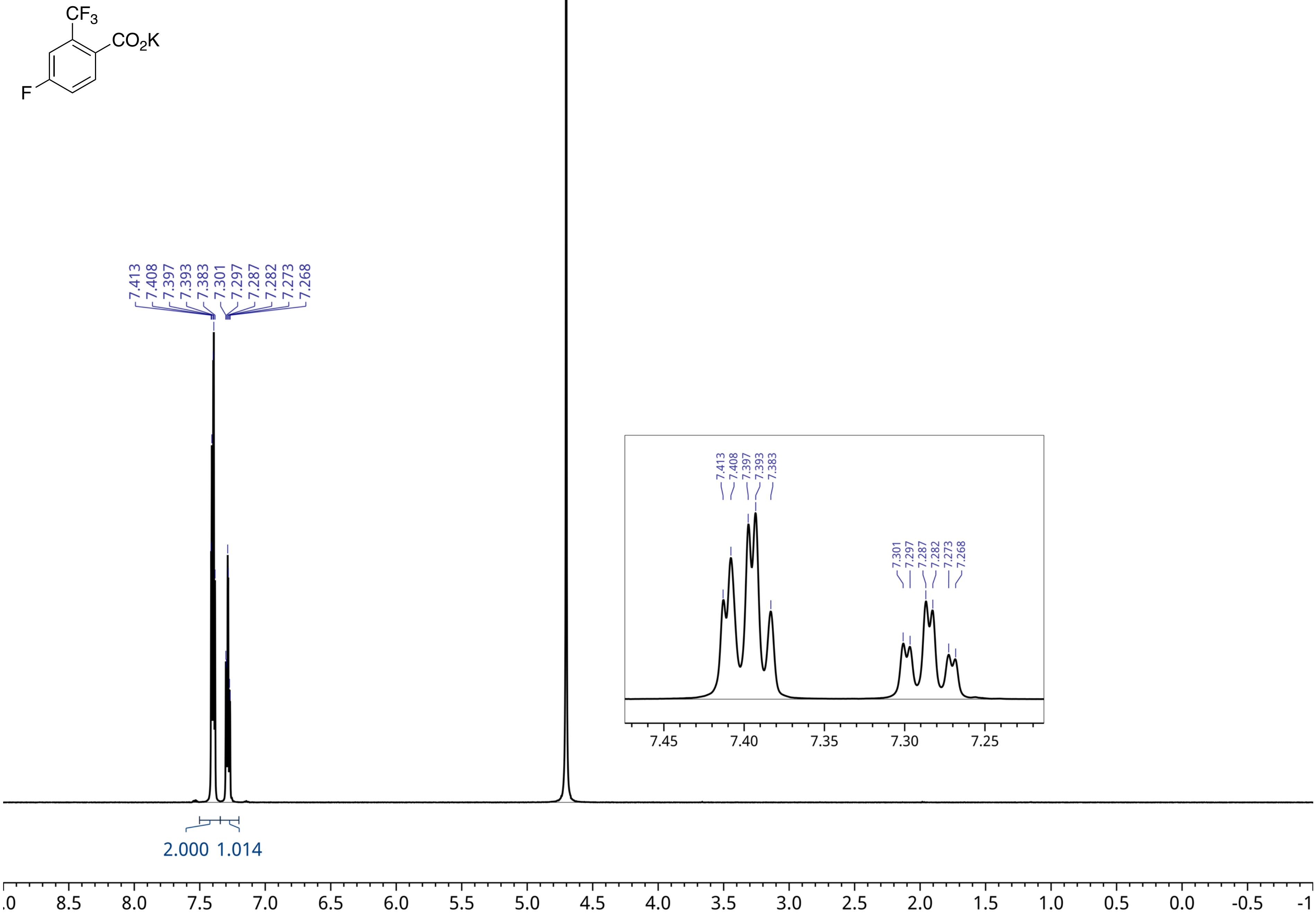

Potassium 4-fluoro-2-(trifluoromethyl)benzoate (**2a**)

$^{13}C\{^1H\}$ NMR (151 MHz, D_2O) δ 175.82, 161.36 (d, J = 246.1 Hz), 135.07, 129.18 (d, J = 8.3 Hz), 126.74 (qd, J = 32.7, 8.2 Hz), 123.11 (qd, J = 272.7, 3.0 Hz), 119.10 (d, J = 21.1 Hz), 113.36 (dq, J = 25.3, 5.0 Hz).

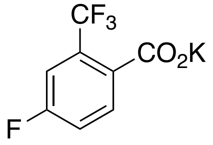

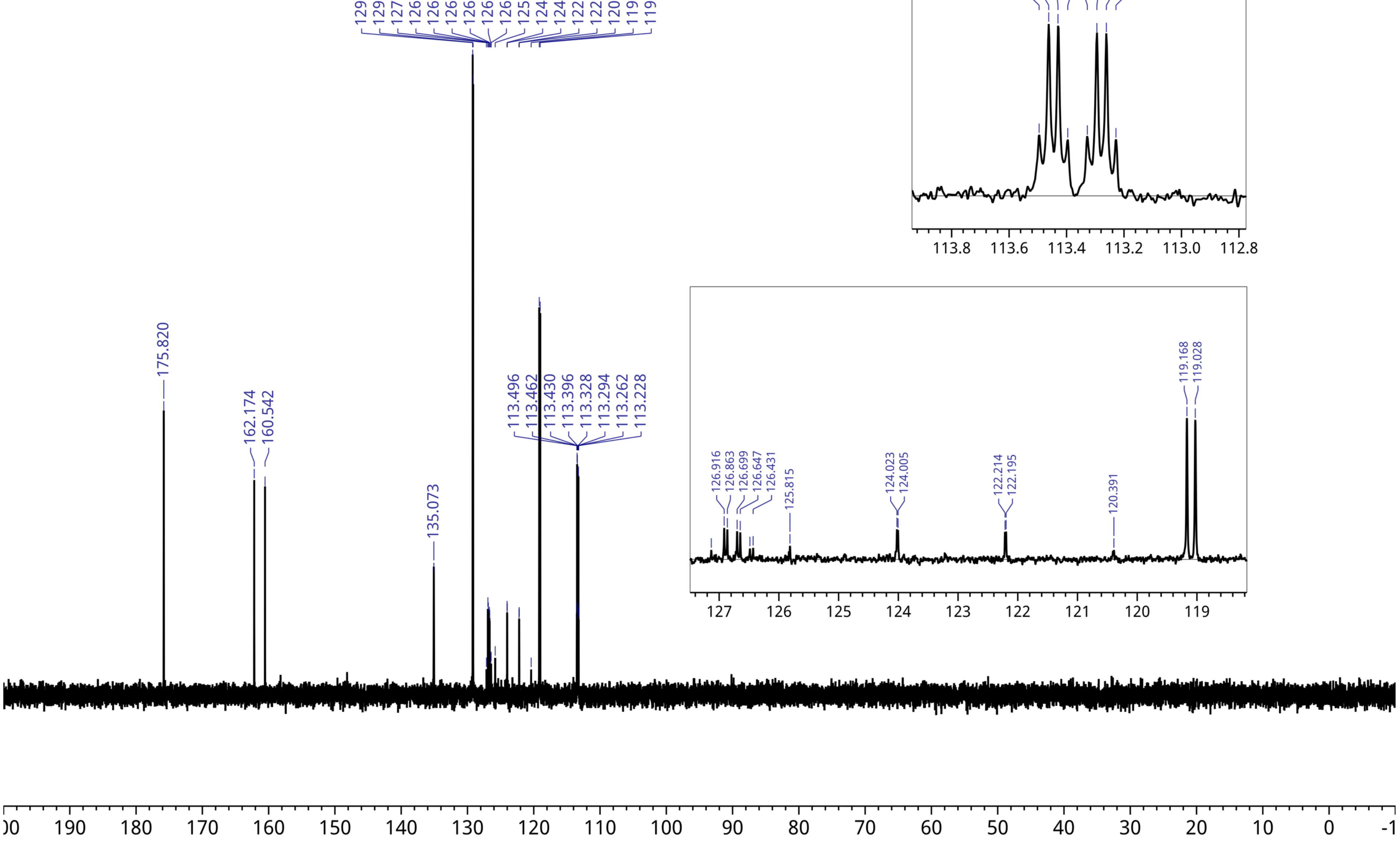

Potassium 4-fluoro-2-(trifluoromethyl)benzoate (**2a**)

^{19}F NMR (564 MHz, D_2O) δ –60.13, –112.48 (td, J = 8.9, 5.5 Hz).

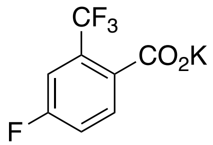

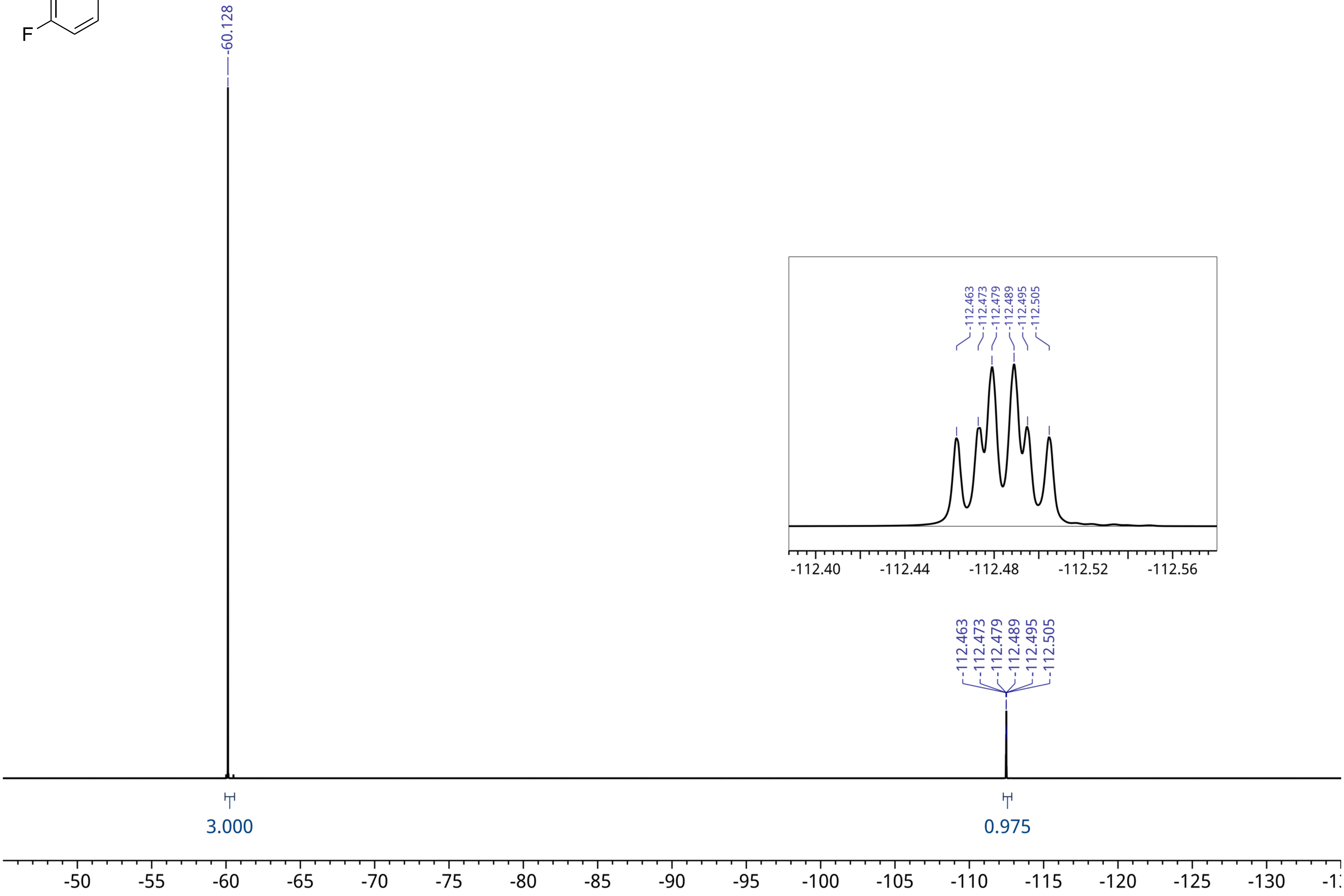

Potassium 4-fluoro-2-methylbenzoate (**3a**)

^{1}H NMR (600 MHz, D_2O) δ 7.27 (dd, J = 8.5, 6.1 Hz, 1H), 6.90 (dd, J = 10.3, 2.7 Hz, 1H), 6.85 (td, J = 8.6, 2.7 Hz, 1H), 2.28 (s, 3H).

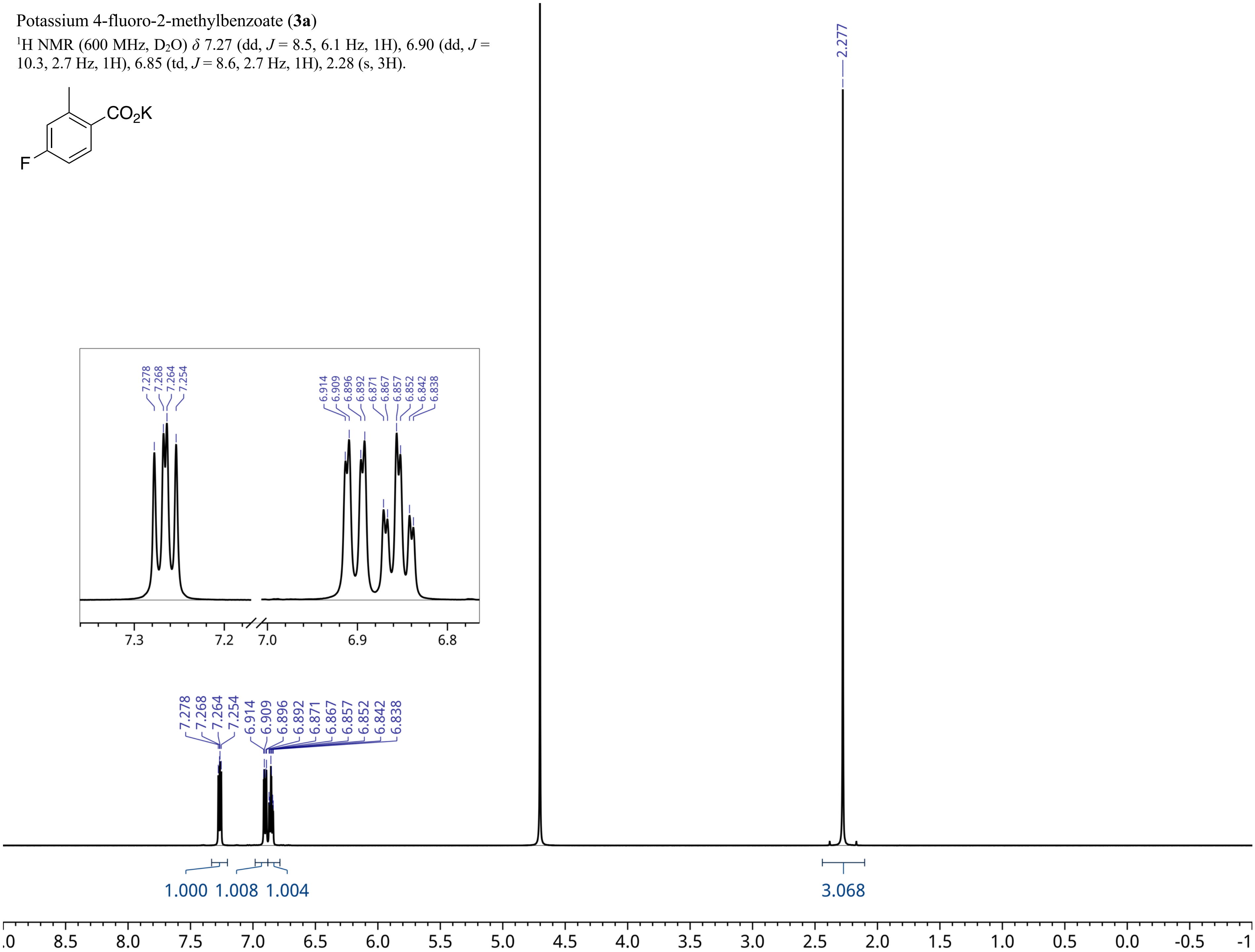

Potassium 4-fluoro-2-methylbenzoate (**3a**)

$^{13}C\{^1H\}$ NMR (151 MHz, D_2O) δ 178.21, 162.14 (d, J = 244.0 Hz), 137.25 (d, J = 8.3 Hz), 135.77 (d, J = 3.0 Hz), 128.20 (d, J = 8.9 Hz), 116.63 (d, J = 21.3 Hz), 112.05 (d, J = 21.4 Hz), 19.23 (d, J = 1.6 Hz).

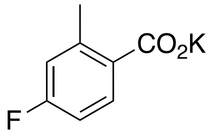

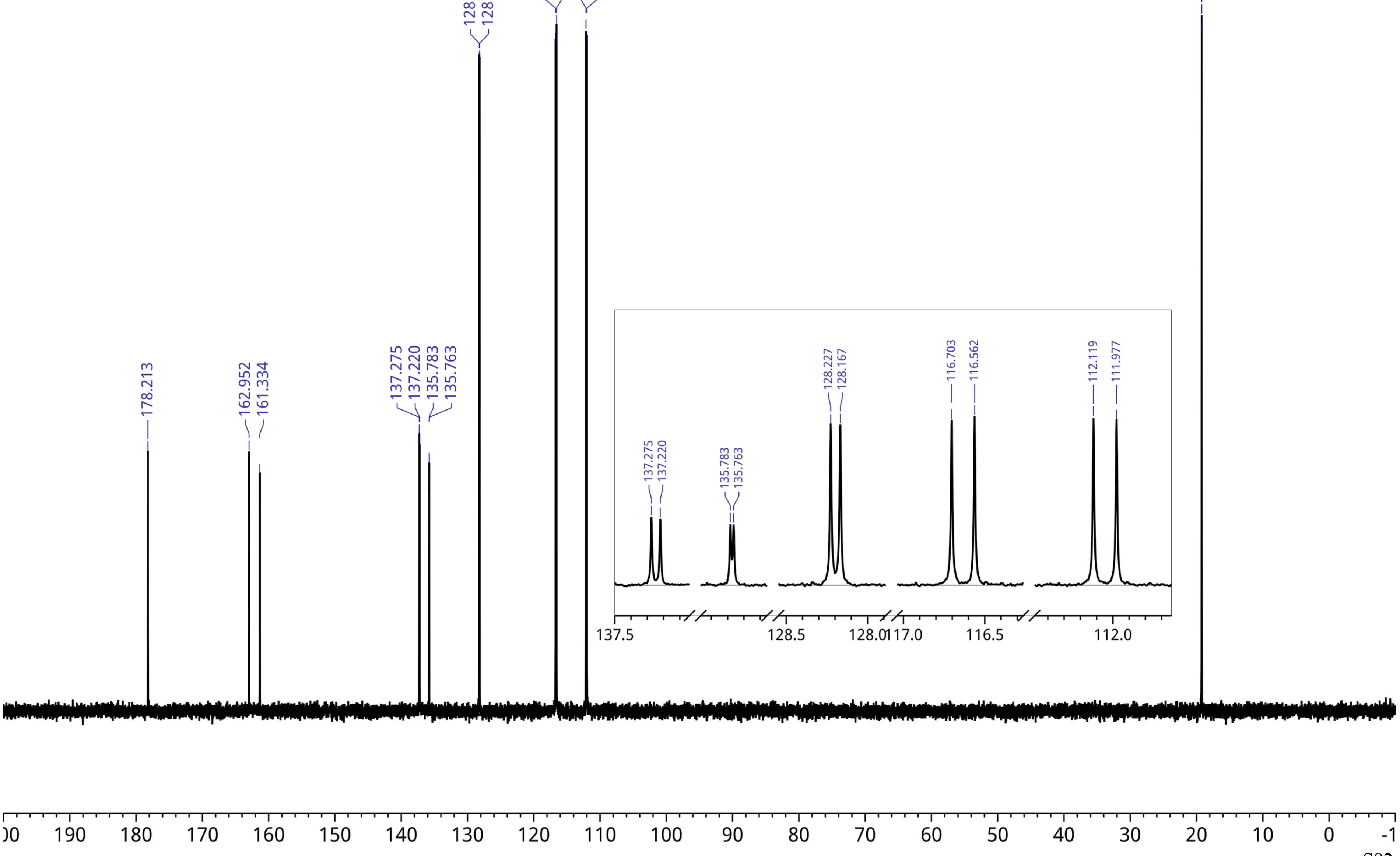

Potassium 4-fluoro-2-methylbenzoate (**3a**)

^{19}F NMR (564 MHz, D_2O) δ –114.32 (ddd, J = 10.3, 8.8, 6.1 Hz).

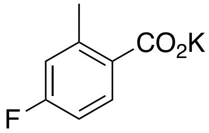

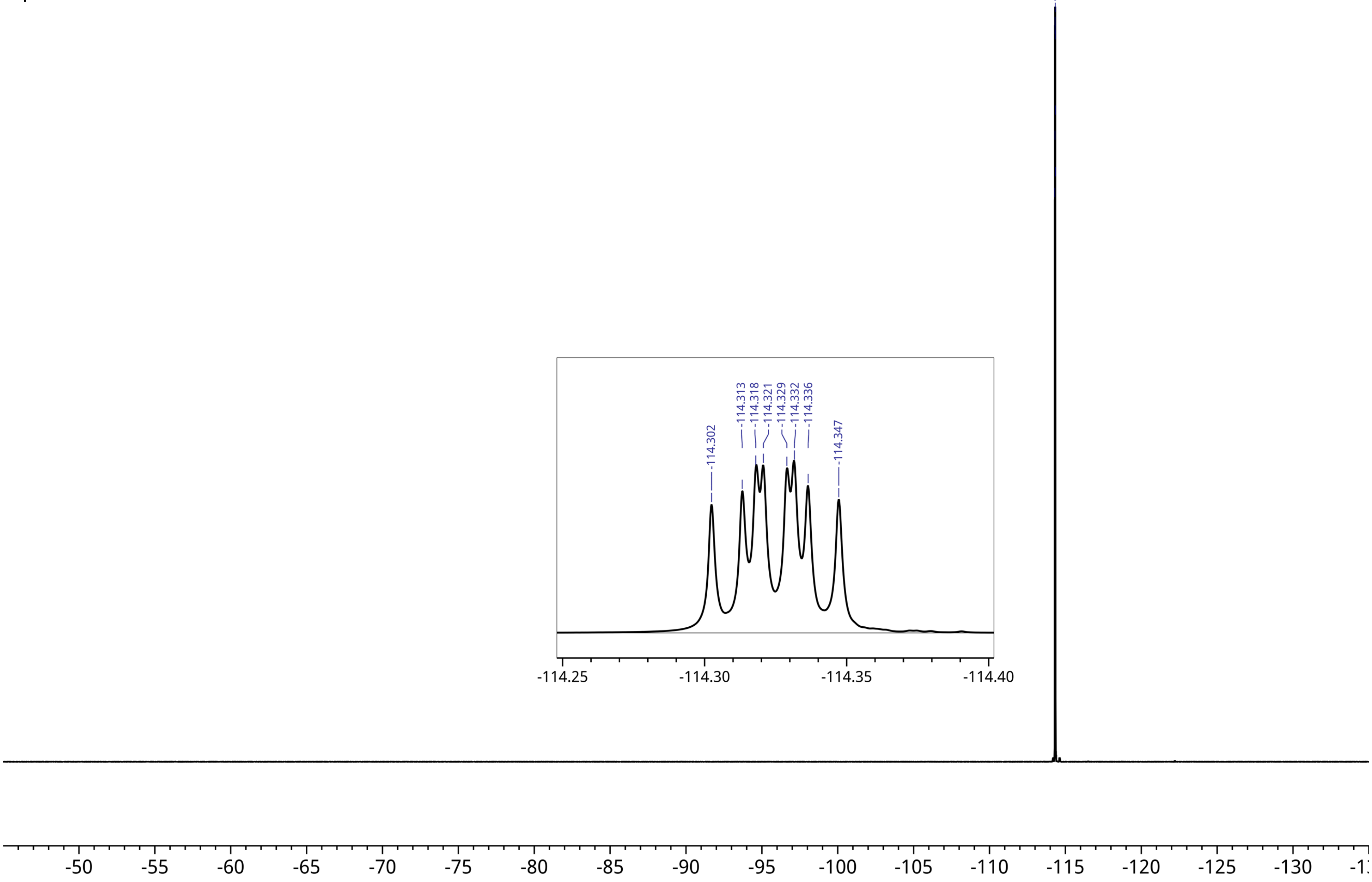

4-Fluoro-2-isopropylbenzoic acid (**4a-H**)

^{1}H NMR (600 MHz, $CDCl_3$) δ 8.01 (dd, J = 8.7, 6.1 Hz, 1H), 7.13 (dd, J = 10.7, 2.6 Hz, 1H), 6.95 (ddd, J = 8.7, 7.7, 2.6 Hz, 1H), 4.02 (heptd, J = 6.8, 1.7 Hz, 1H), 1.27 (d, J = 6.8 Hz, 6H).

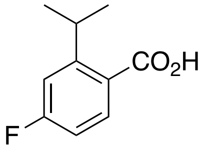

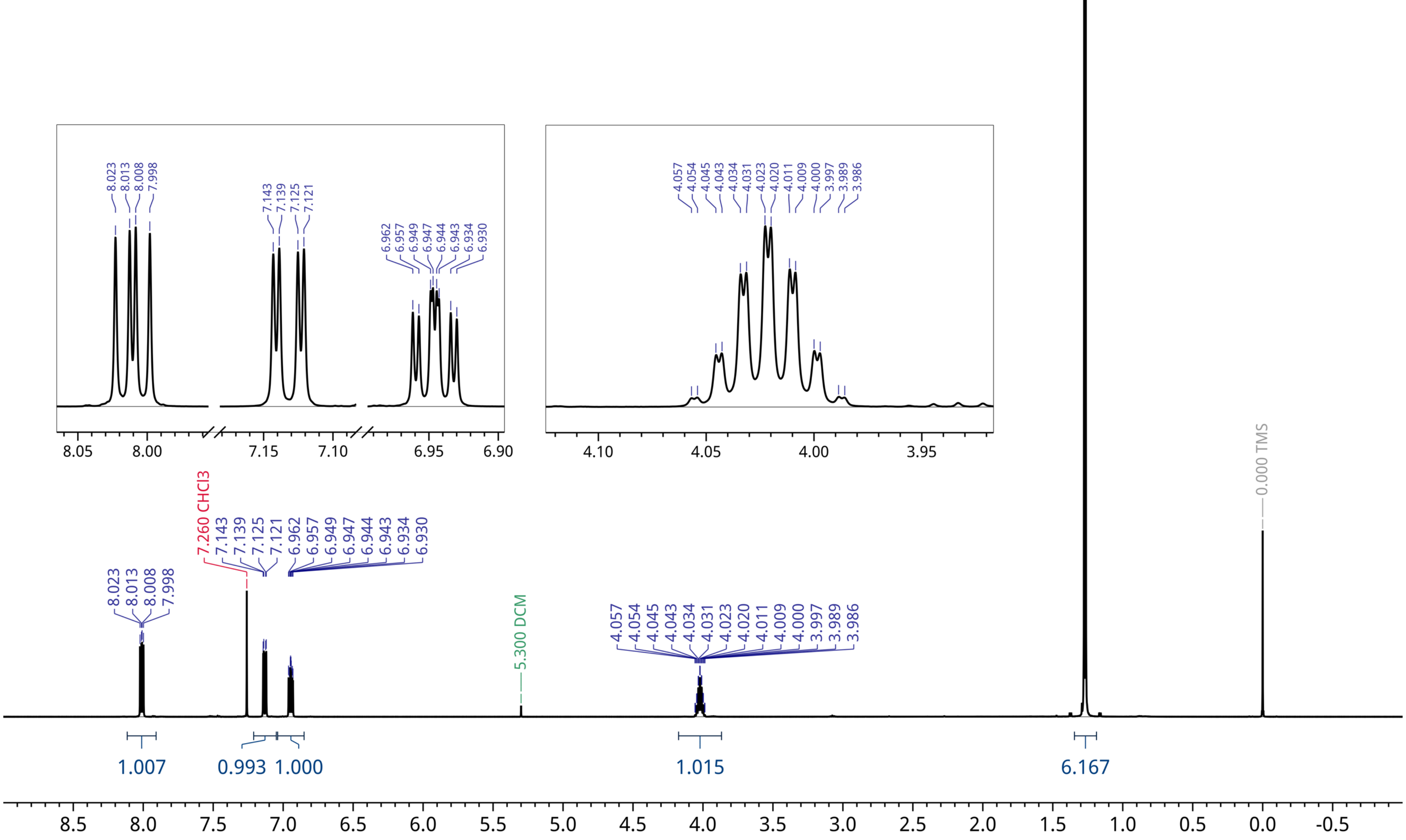

4-Fluoro-2-isopropylbenzoic acid (**4a-H**)

$^{13}C\{^{1}H\}$ NMR (151 MHz, $CDCl_3$) δ 172.34, 165.72 (d, J = 253.7 Hz), 155.30 (d, J = 7.9 Hz), 133.98 (d, J = 9.5 Hz), 123.93 (d, J = 2.9 Hz), 113.64 (d, J = 21.7 Hz), 112.80 (d, J = 21.8 Hz), 29.45 (d, J = 1.4 Hz), 23.81.

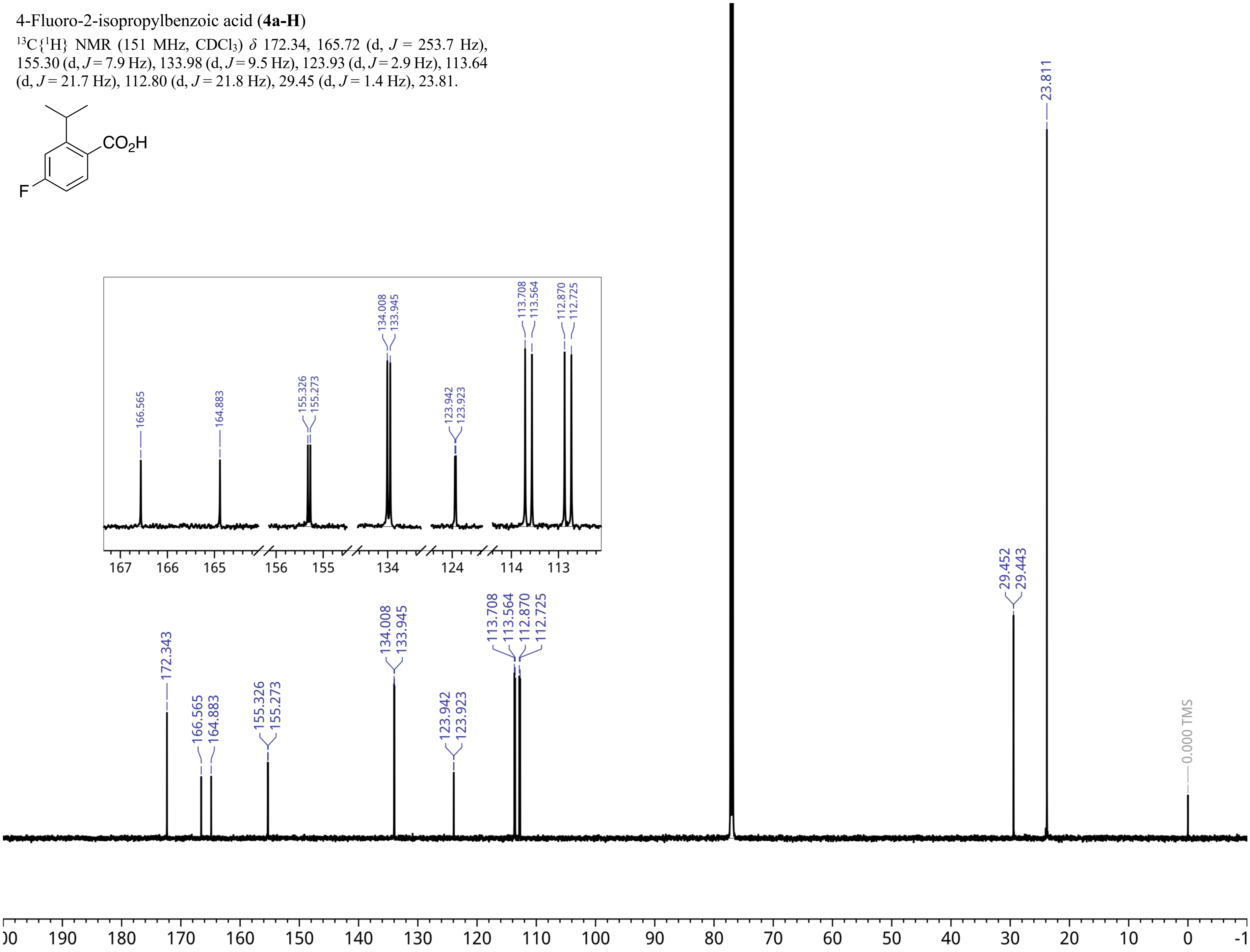

4-Fluoro-2-isopropylbenzoic acid (**4a-H**)

^{19}F NMR (564 MHz, $CDCl_3$) δ –105.51 (dddd, J = 10.7, 7.8, 6.1, 1.7 Hz).

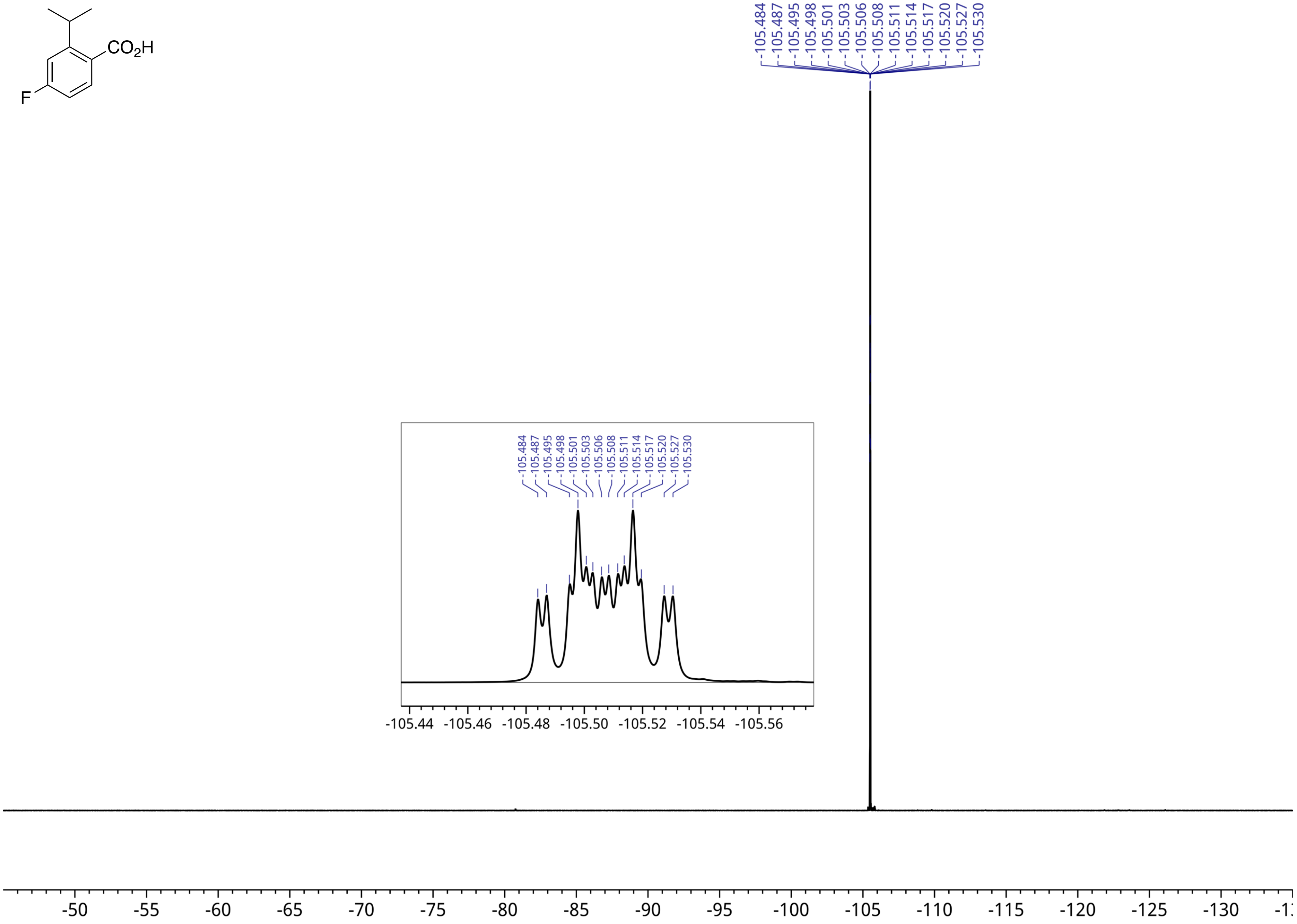

Potassium 4-fluoro-2-isopropylbenzoate (**4a**)

^{1}H NMR (600 MHz, D_2O) δ 7.16 (dd, J = 8.4, 6.1 Hz, 1H), 7.04 (dd, J = 11.2, 2.6 Hz, 1H), 6.84 (td, J = 8.7, 2.7 Hz, 1H), 3.15 (heptd, J = 6.4, 1.4 Hz, 1H), 1.11 (d, J = 6.9 Hz, 6H).

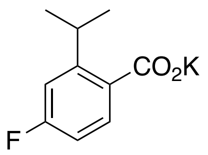

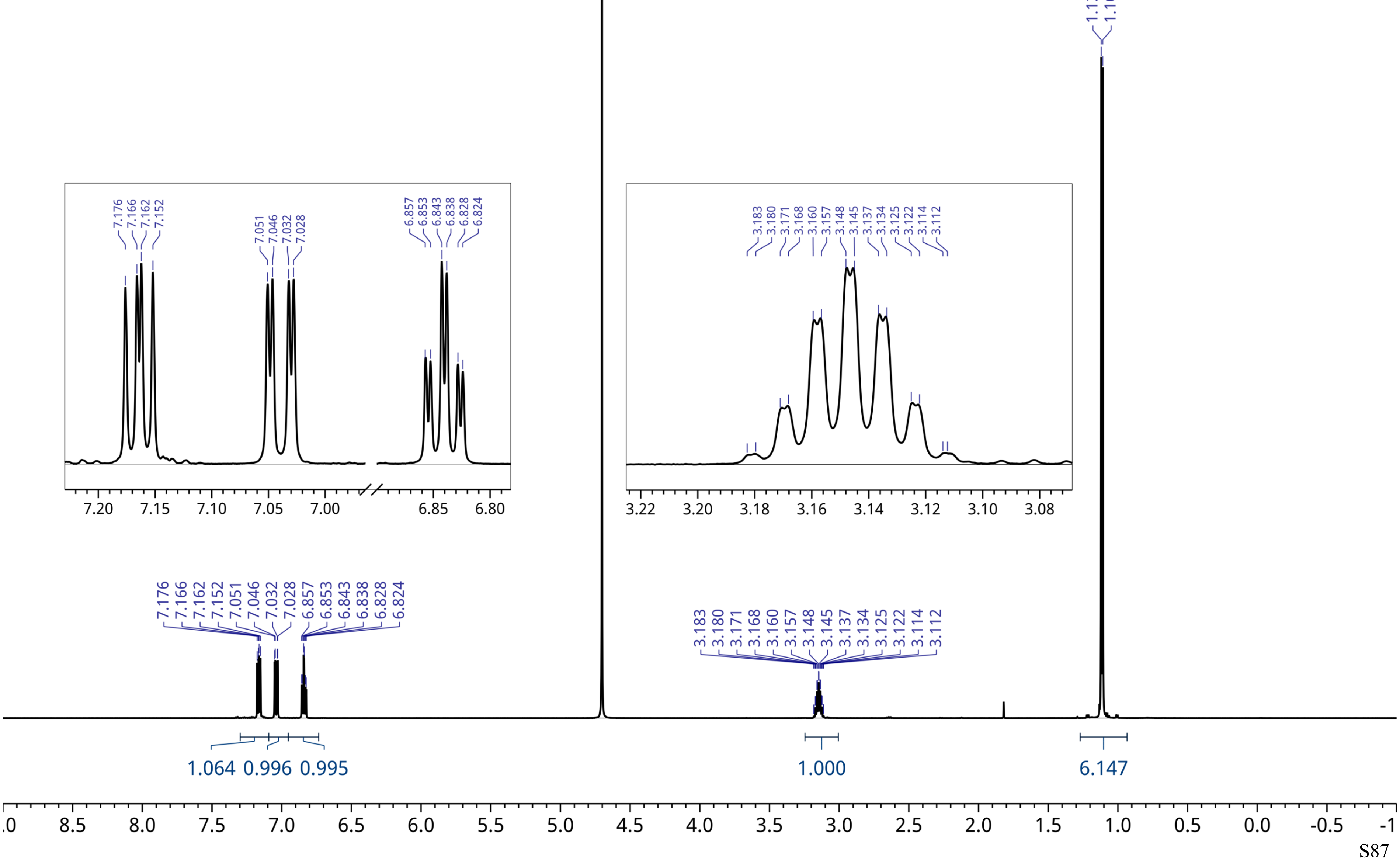

Potassium 4-fluoro-2-isopropylbenzoate (**4a**)

$^{13}C\{^{1}H\}$ NMR (151 MHz, D_2O) δ 178.89, 162.46 (d, J = 243.1 Hz), 147.43 (d, J = 7.0 Hz), 135.68 (d, J = 3.0 Hz), 127.41, 112.19 (d, J = 10.4 Hz), 112.04 (d, J = 10.3 Hz), 30.26 (d, J = 1.6 Hz), 23.02.

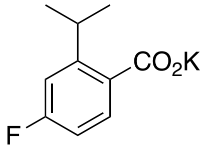

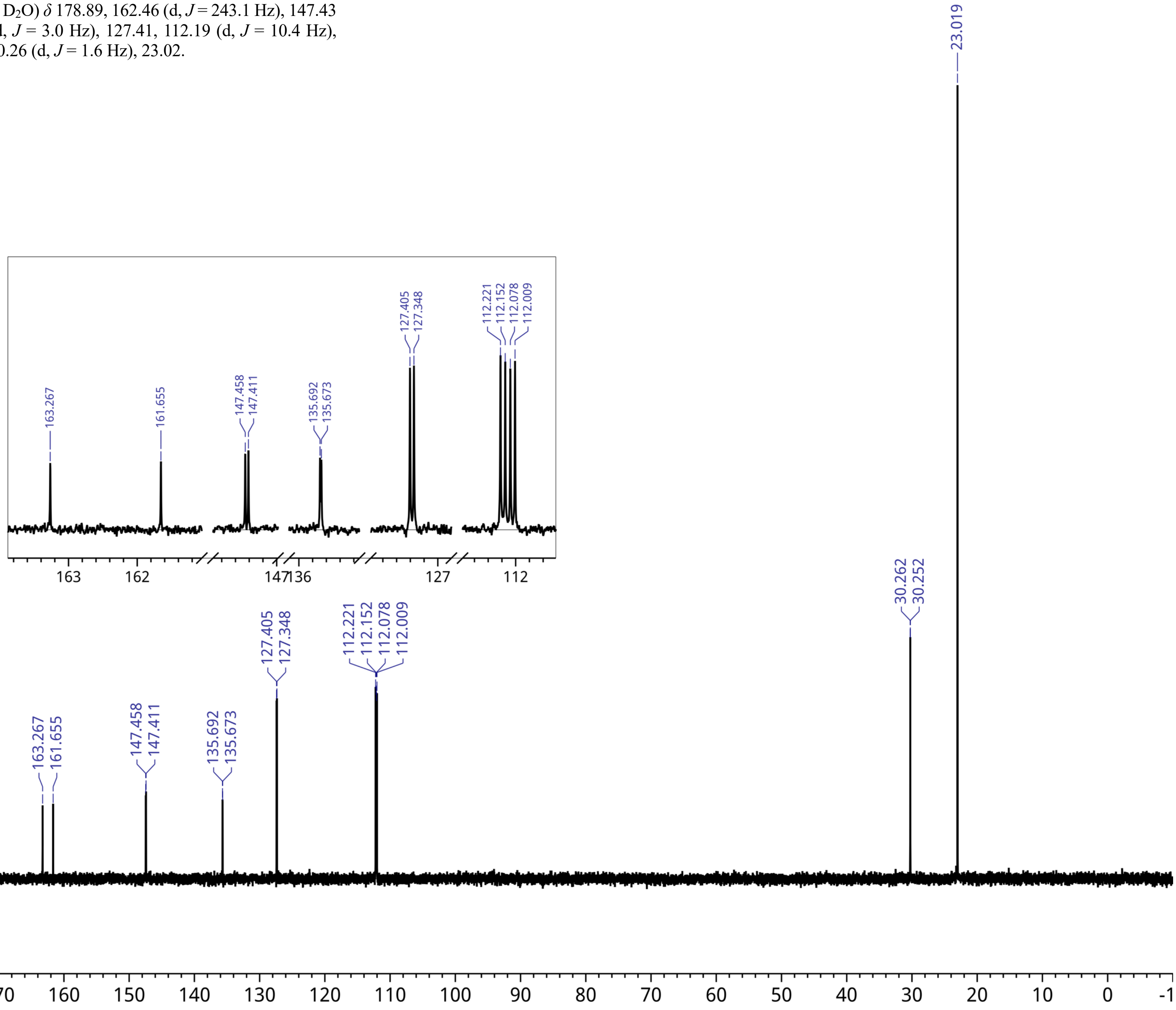

Potassium 4-fluoro-2-isopropylbenzoate (**4a**)

^{19}F NMR (564 MHz, D_2O) δ –114.07 (dddd, J = 11.2, 8.7, 6.1, 1.8 Hz).

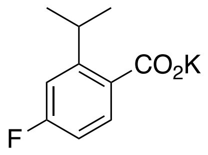

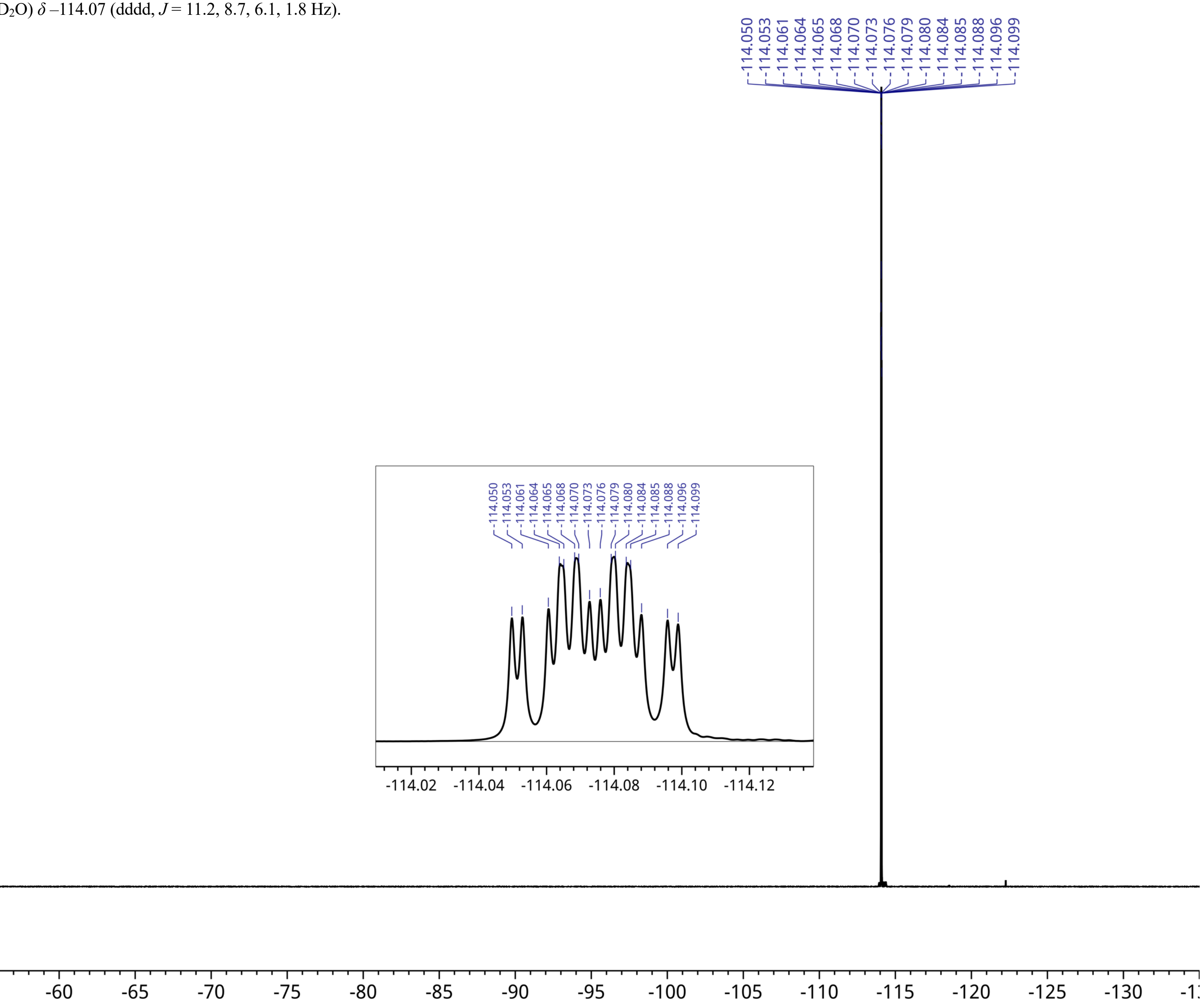

Potassium 2-(2-(trifluoromethyl)phenyl)acetate (**1b**)

^{1}H NMR (600 MHz, D_2O) δ 7.63 (d, J = 7.9 Hz, 1H), 7.50 (t, J = 7.6 Hz, 1H), 7.35 (t, J = 7.7 Hz, 1H), 7.31 (d, J = 7.7 Hz, 1H), 3.64 (s, 2H).

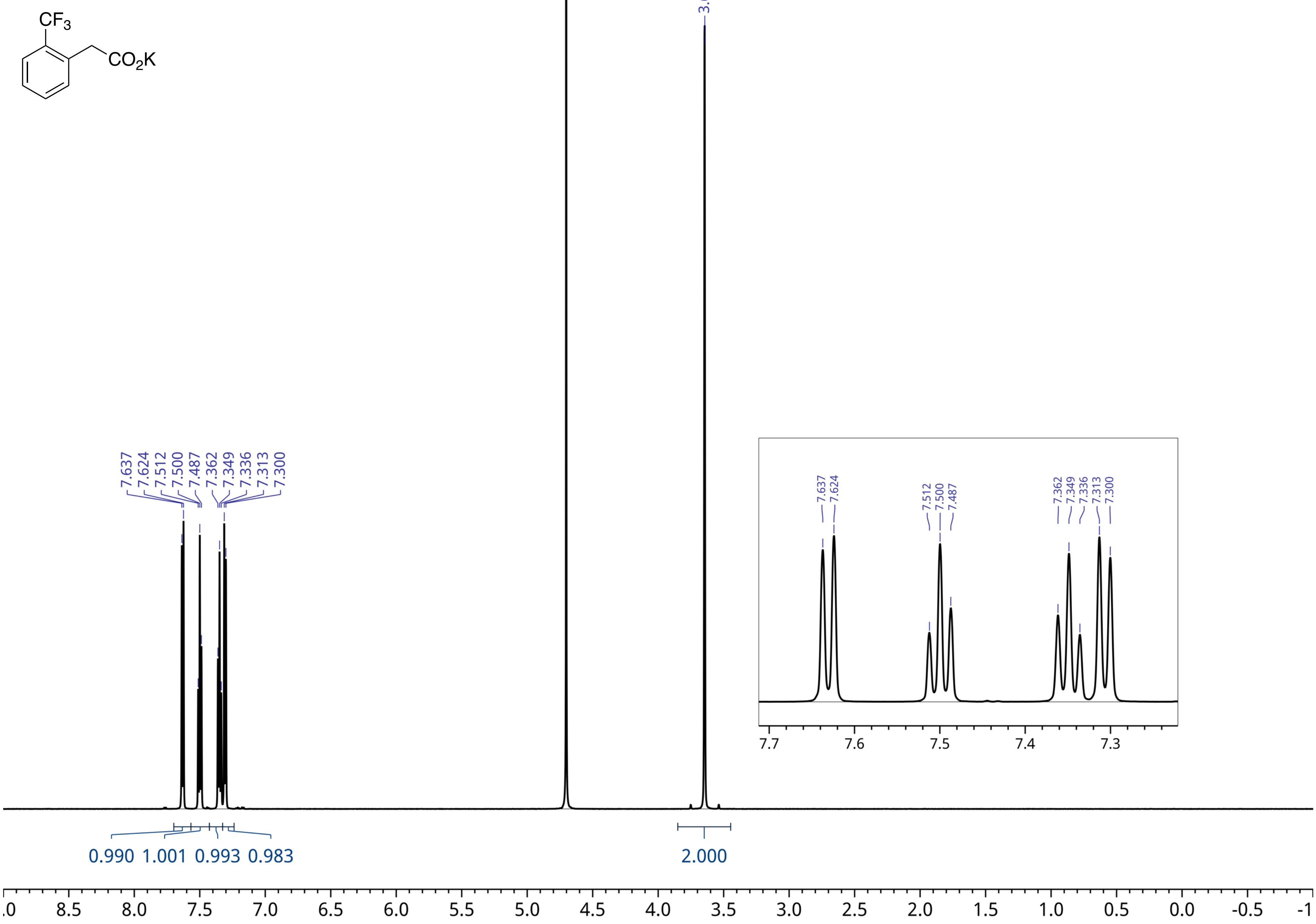

Potassium 2-(2-(trifluoromethyl)phenyl)acetate (**1b**)

$^{13}C\{^{1}H\}$ NMR (151 MHz, D_2O) δ 179.78, 135.25 (q, J = 1.7 Hz), 132.86, 132.22, 127.94 (q, J = 29.7 Hz), 126.87, 125.92 (q, J = 5.6 Hz), 124.59 (q, J = 273.2 Hz), 41.47 (q, J = 2.0 Hz).

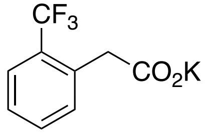

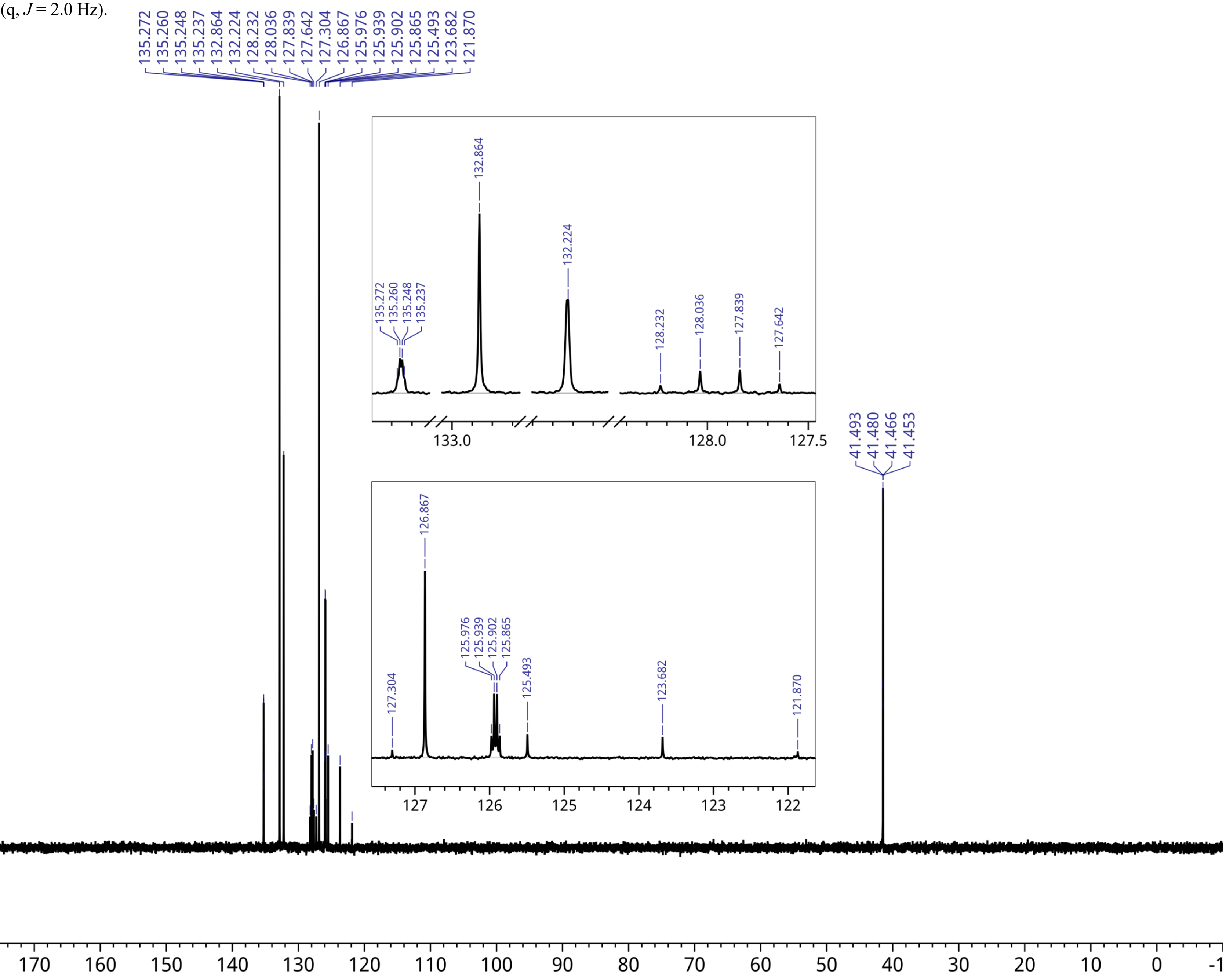

Potassium 2-(2-(trifluoromethyl)phenyl)acetate (**1b**)

^{19}F NMR (564 MHz, D_2O) δ –60.12.

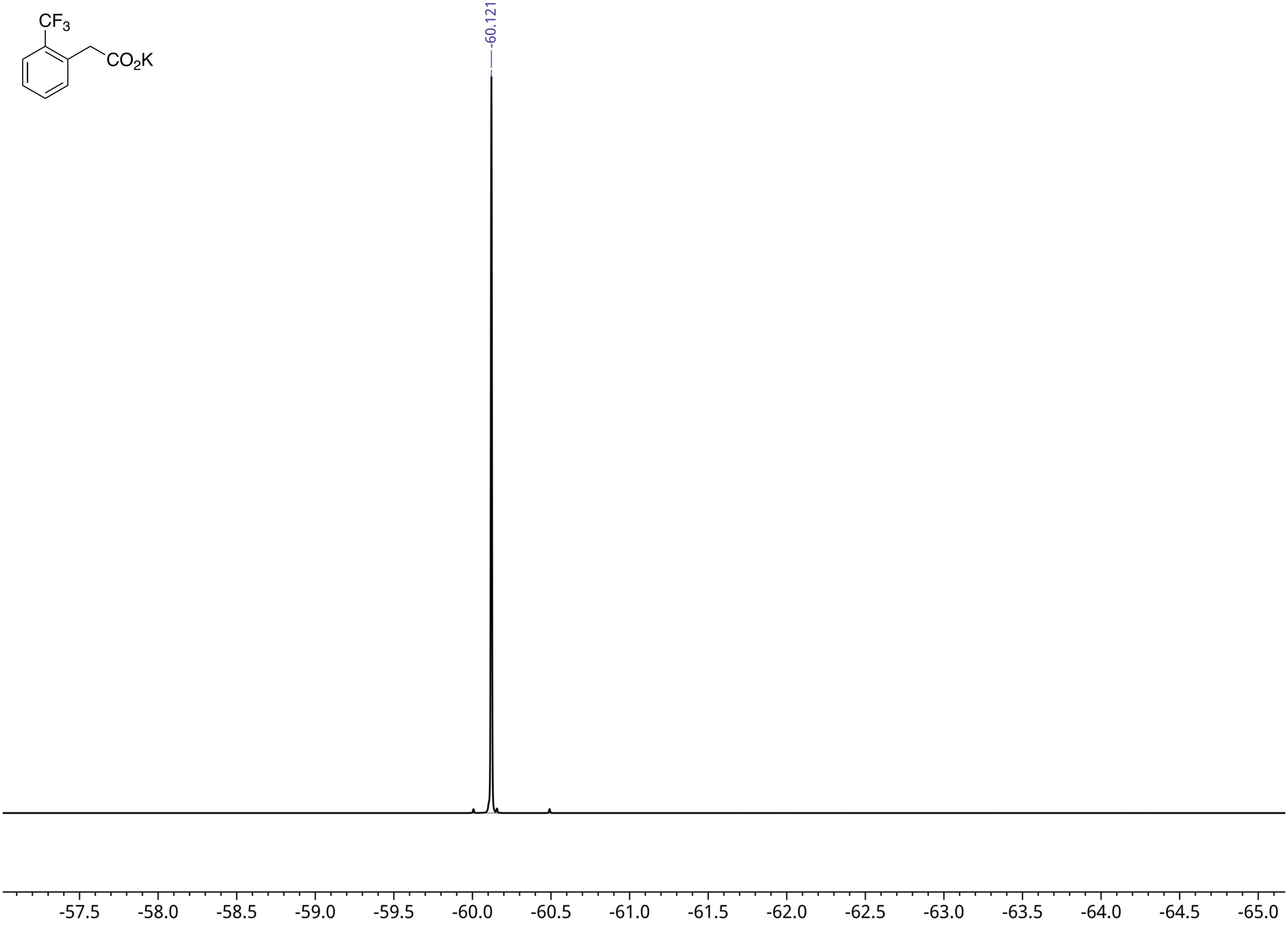

Potassium 2-(4-fluoro-2-(trifluoromethyl)phenyl)acetate (**2b**)

^{1}H NMR (600 MHz, D_2O) δ 7.39 (dd, *J* = 9.5, 2.8 Hz, 1H), 7.31 (dd, *J* = 8.6, 5.6 Hz, 1H), 7.24 (td, *J* = 8.5, 2.8 Hz, 1H), 3.61 (s, 2H).

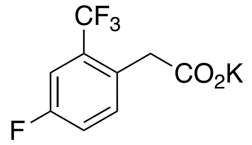

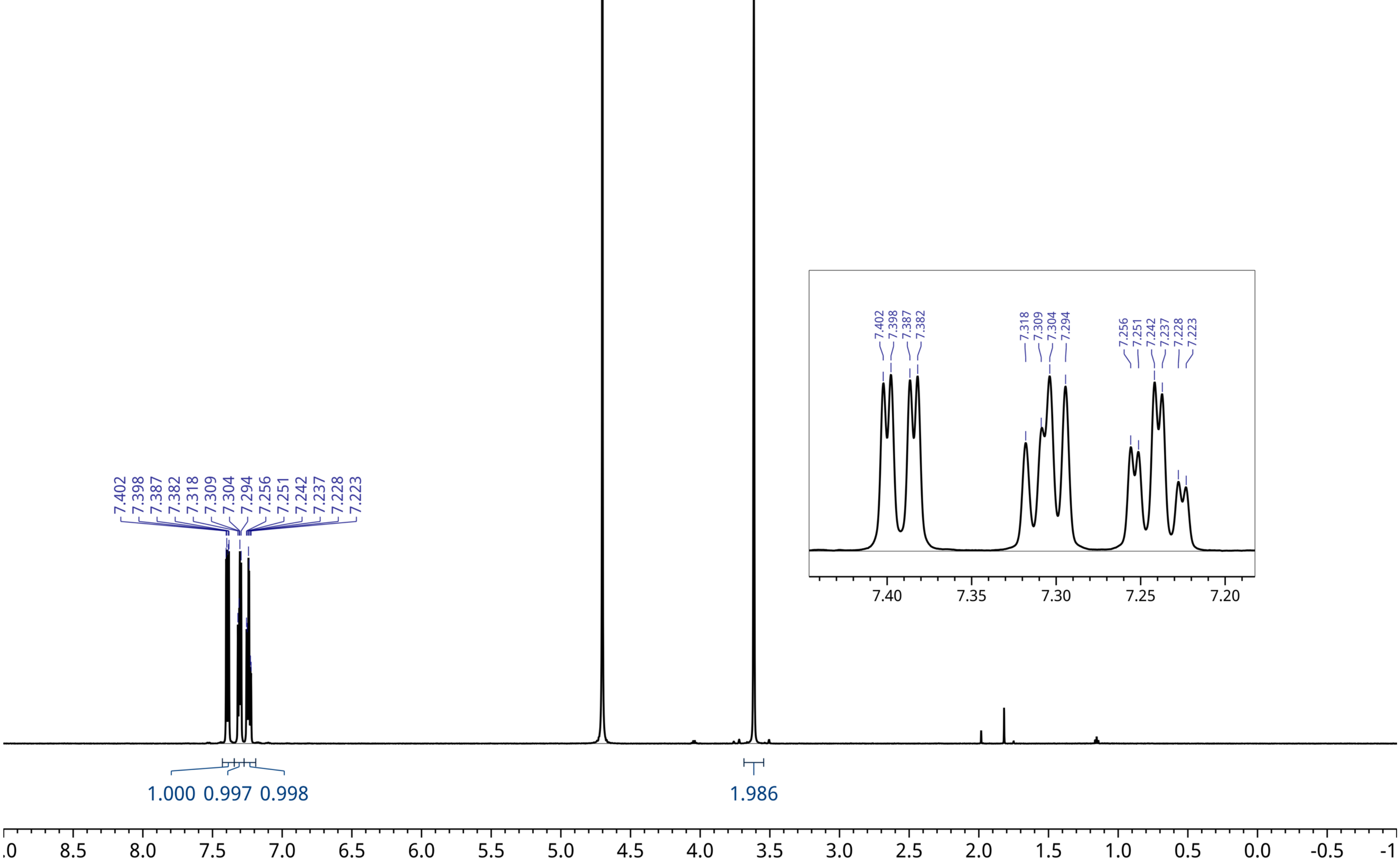

Potassium 2-(4-fluoro-2-(trifluoromethyl)phenyl)acetate (**2b**)

$^{13}C\{^{1}H\}$ NMR (151 MHz, D_2O) δ 179.58, 160.74 (d, J = 243.9 Hz), 134.87 (d, J = 8.0 Hz), 131.38 (dq, J = 3.6, 1.7 Hz), 129.54 (qd, J = 31.1, 7.7 Hz), 124.05 (qd, J = 273.2, 3.0 Hz), 118.89 (d, J = 20.9 Hz), 113.34 (dq, J = 25.3, 5.8 Hz), 40.68 (q, J = 2.0 Hz).

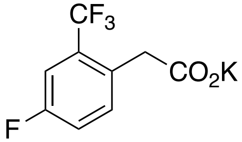

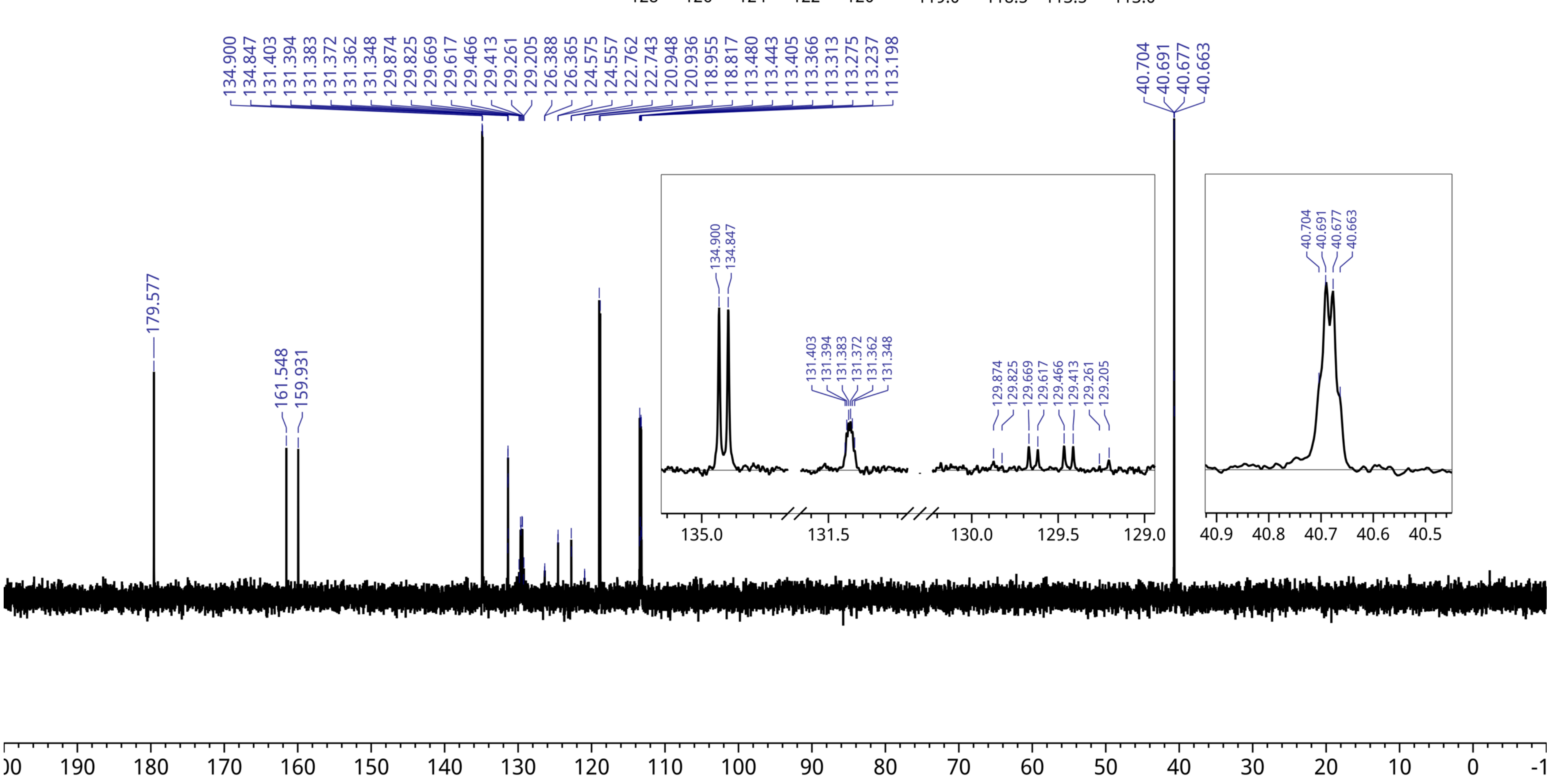

Potassium 2-(4-fluoro-2-(trifluoromethyl)phenyl)acetate (**2b**)

^{19}F NMR (564 MHz, D_2O) δ –60.77, –115.55 (td, J = 8.9, 5.6 Hz).

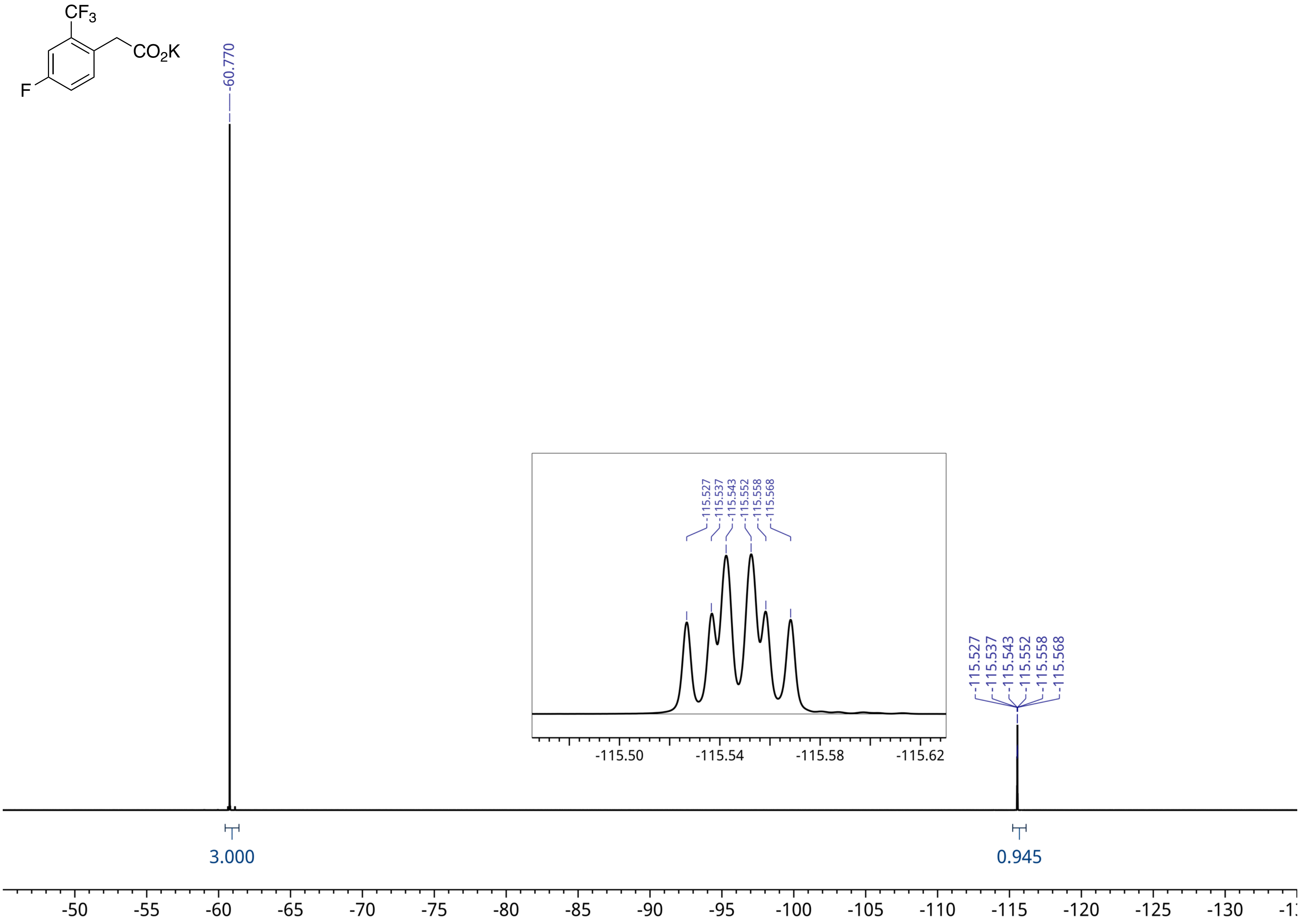

Potassium 2-(4-fluoro-2-methylphenyl)acetate (**3b**)

^{1}H NMR (600 MHz, D_2O) δ 7.06 (dd, J = 8.4, 6.1 Hz, 1H), 6.89 (dd, J = 10.2, 2.8 Hz, 1H), 6.83 (td, J = 8.7, 2.9 Hz, 1H), 3.42 (s, 2H), 2.14 (s, 3H).

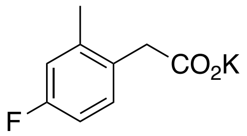

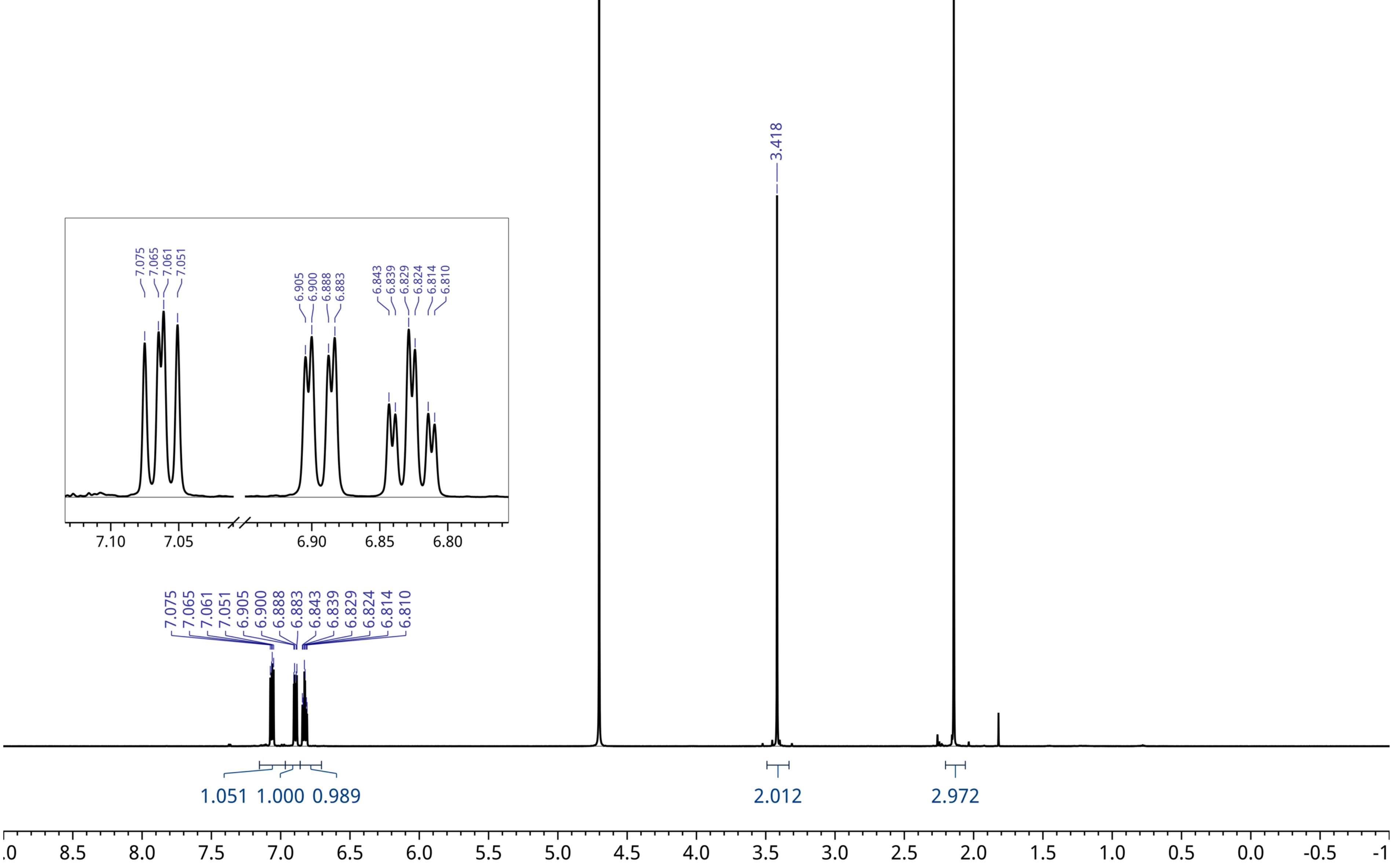

Potassium 2-(4-fluoro-2-methylphenyl)acetate (**3b**)

$^{13}C\{^{1}H\}$ NMR (151 MHz, D_2O) δ 180.69 (d, J = 1.2 Hz), 161.33 (d, J = 241.0 Hz), 139.62, 131.81 (d, J = 3.0 Hz), 131.59 (d, J = 8.5 Hz), 116.37 (d, J = 21.1 Hz), 112.23 (d, J = 20.8 Hz), 41.49, 18.70 (d, J = 1.6 Hz).

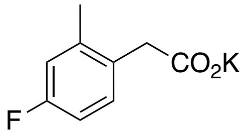

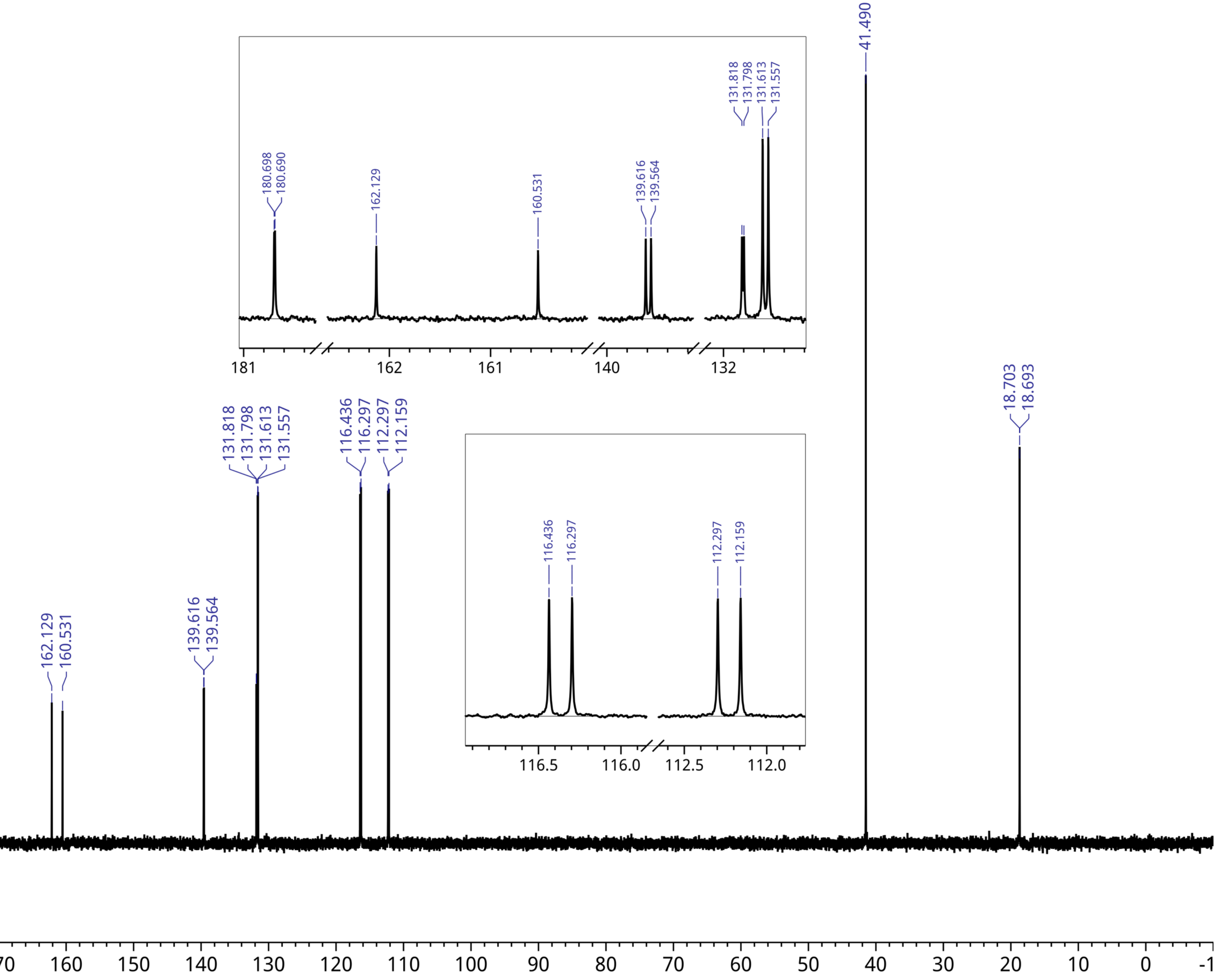

Potassium 2-(4-fluoro-2-methylphenyl)acetate (**3b**)

^{19}F NMR (564 MHz, D_2O) δ –117.85 (ddd, J = 10.2, 8.9, 6.1 Hz).

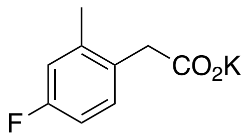

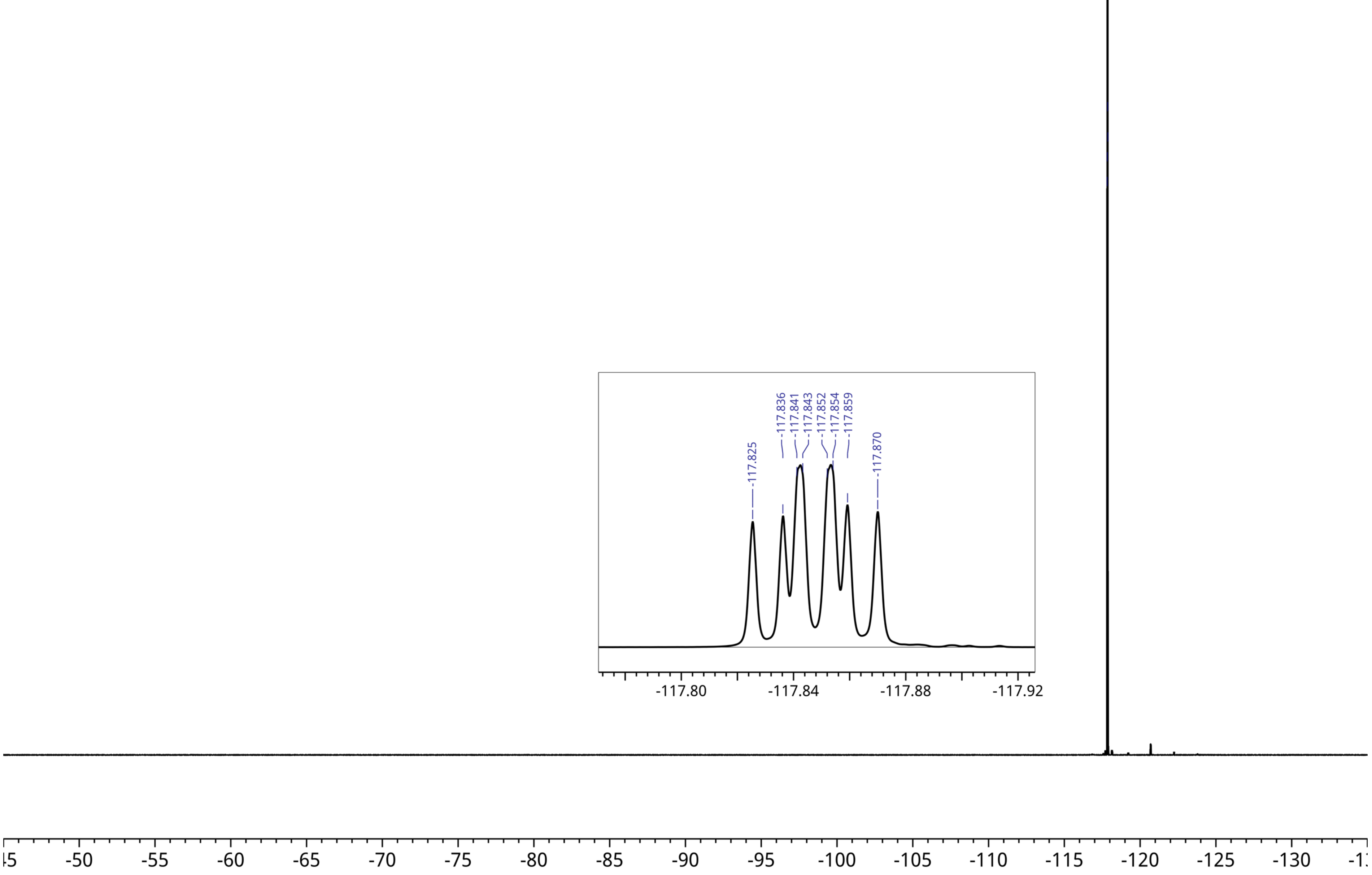

N-acetyl-*L*-phenylglycine (**Ac-*α*-*L*-Ph-OH**)

^{1}H NMR (600 MHz, MeOD-d_4) δ 7.44 – 7.39 (m, 2H), 7.37 (m, 2H), 7.35 – 7.31 (m, 1H), 5.43 (s, 1H), 2.01 (s, 3H).

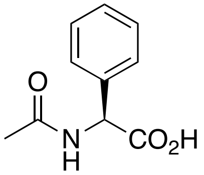

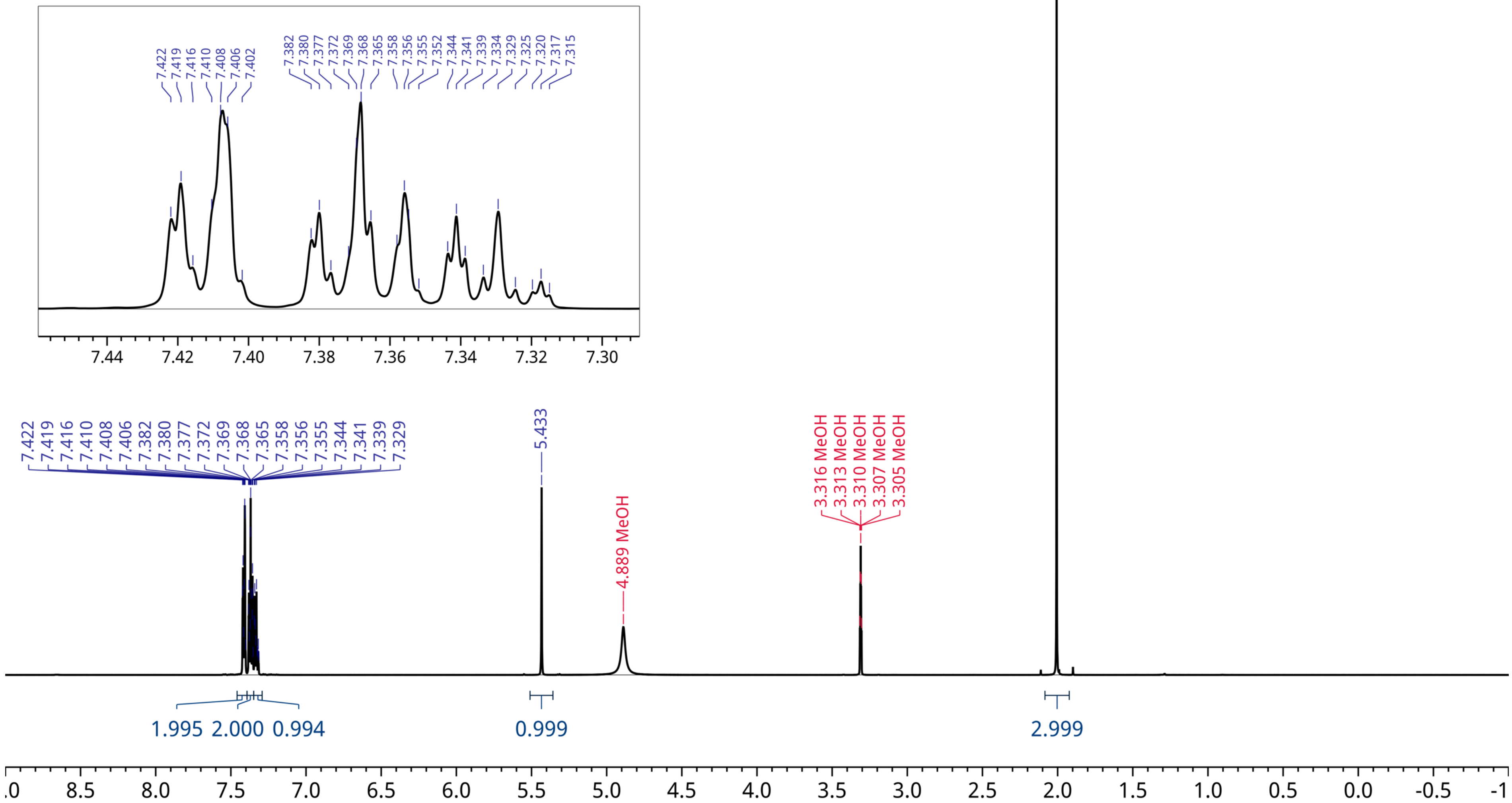

N-acetyl-*L*-phenylglycine (**Ac-*α*-*L*-Ph-OH**)

$^{13}C\{^{1}H\}$ NMR (151 MHz, MeOD-d_4) δ 173.73, 172.89, 138.04, 129.80, 129.38, 128.80, 58.24, 22.25.

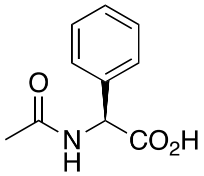

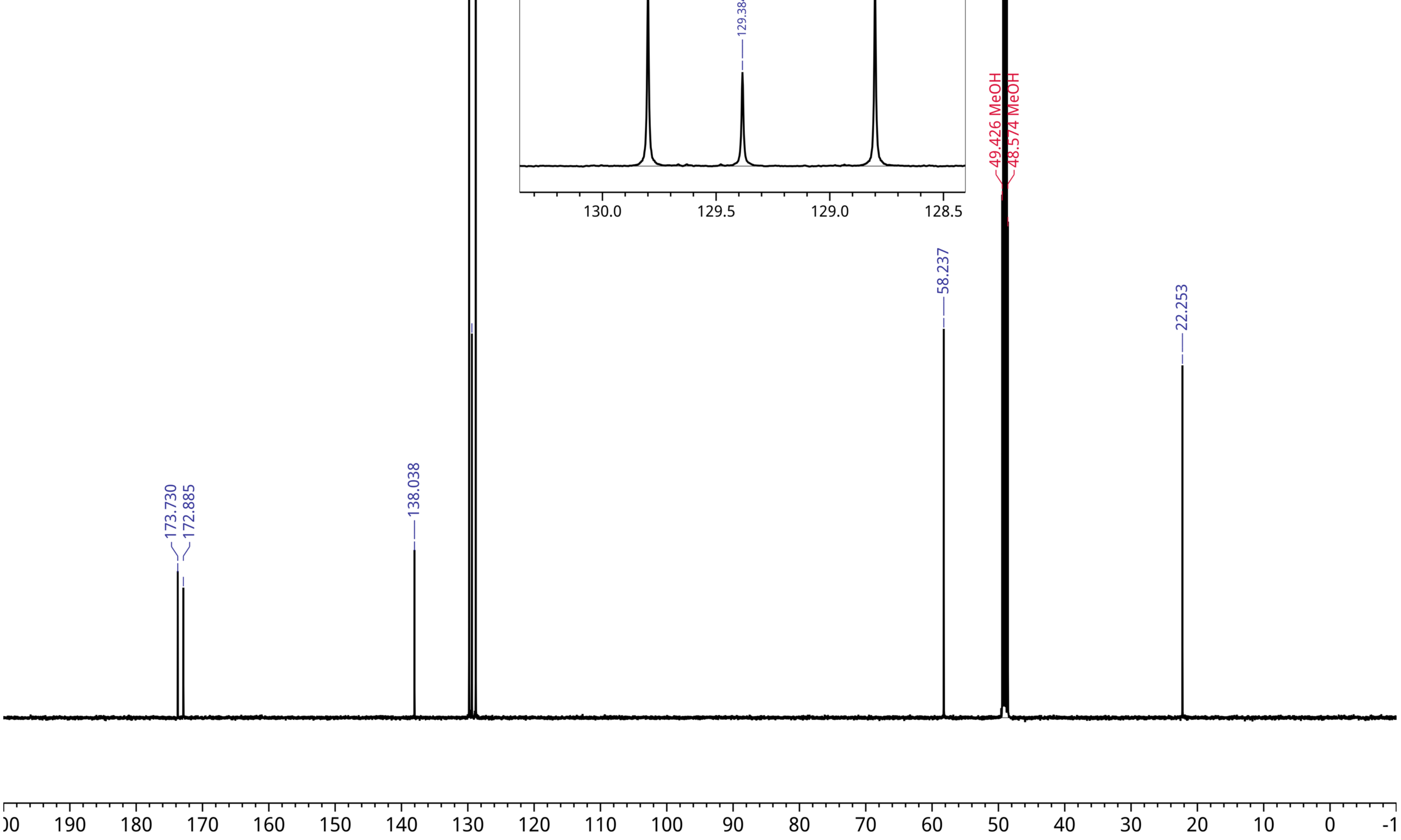

N-acetyl-*α*,*α*-dimethylglycine (**Ac-*α*-Me₂-OH**)

^{1}H NMR (600 MHz, MeOD-*d*$_4$) δ 1.91 (s, 3H), 1.45 (s, 6H).

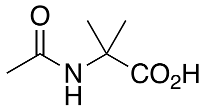

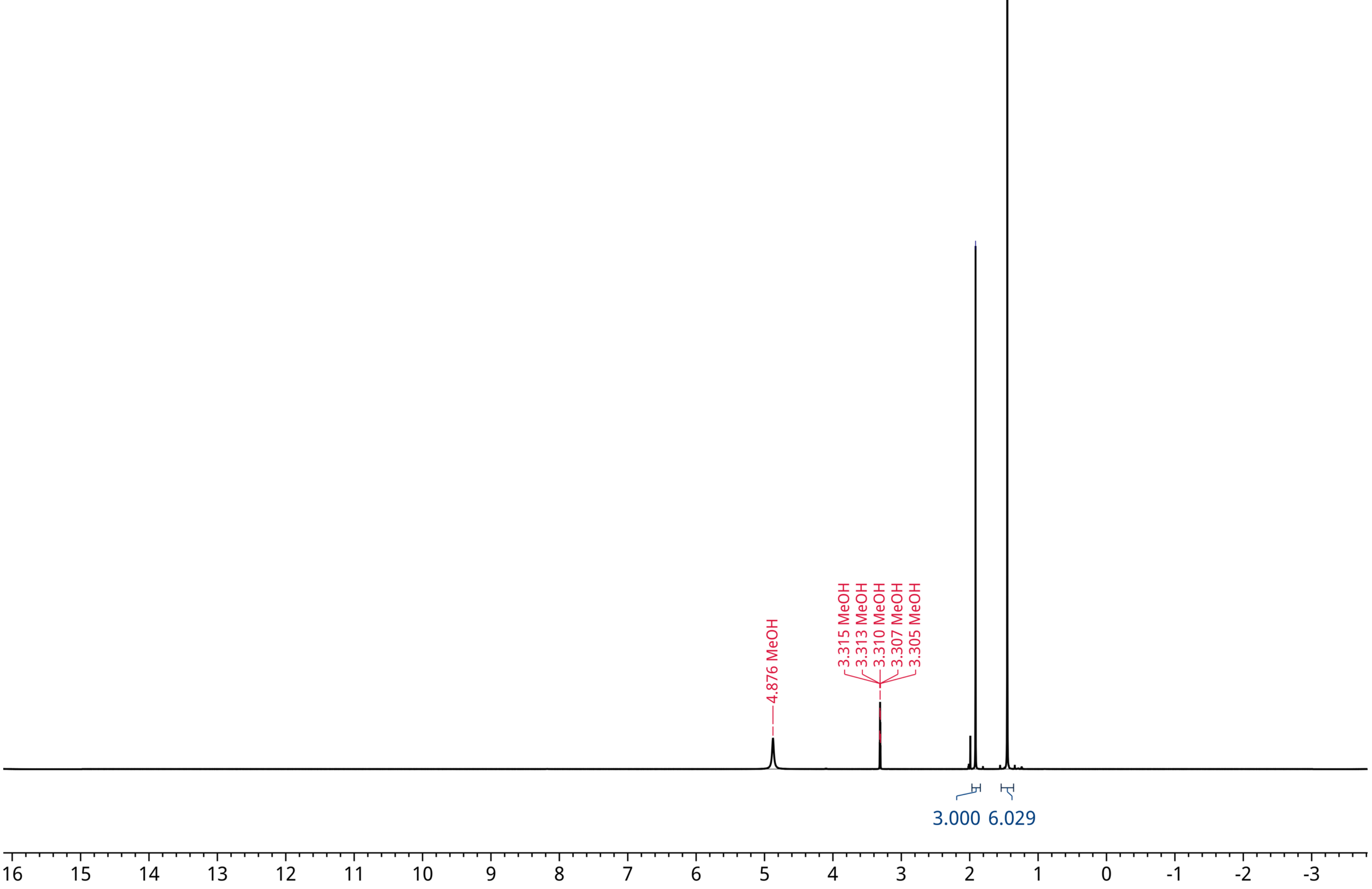

N-acetyl-α,α-dimethylglycine (Ac-α-Me_2-OH)

$^{13}C\{^1H\}$ NMR (151 MHz, MeOD-d_4) δ 178.00, 172.61, 56.82, 25.35, 22.53.

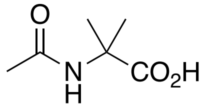

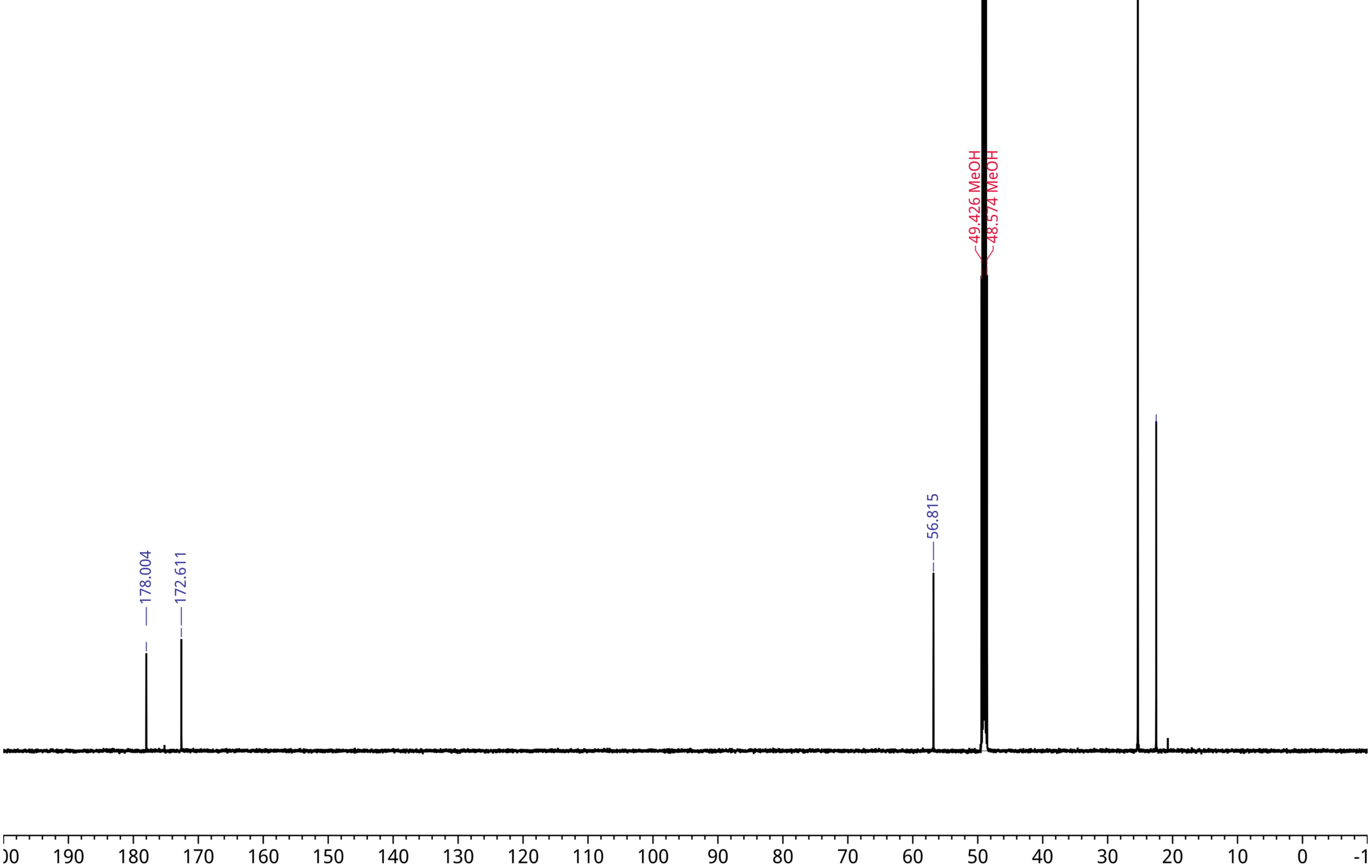

N-acetyl-β^2-*L*-homovaline (**Ac-β^2-*L*-Val-OH**)

^{1}H NMR (600 MHz, MeOD-d_4) δ 3.46 (dd, J = 13.4, 4.7 Hz, 1H), 3.27 (dd, J = 13.4, 9.5 Hz, 1H), 2.41 (ddd, J = 9.5, 6.7, 4.7 Hz, 1H), 1.91 (heptd, J = 6.8, 6.8 Hz, 1H), 1.91 (s, 3H), 0.99 (dd, J = 19.4, 6.8 Hz, 6H).

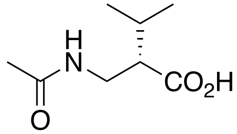

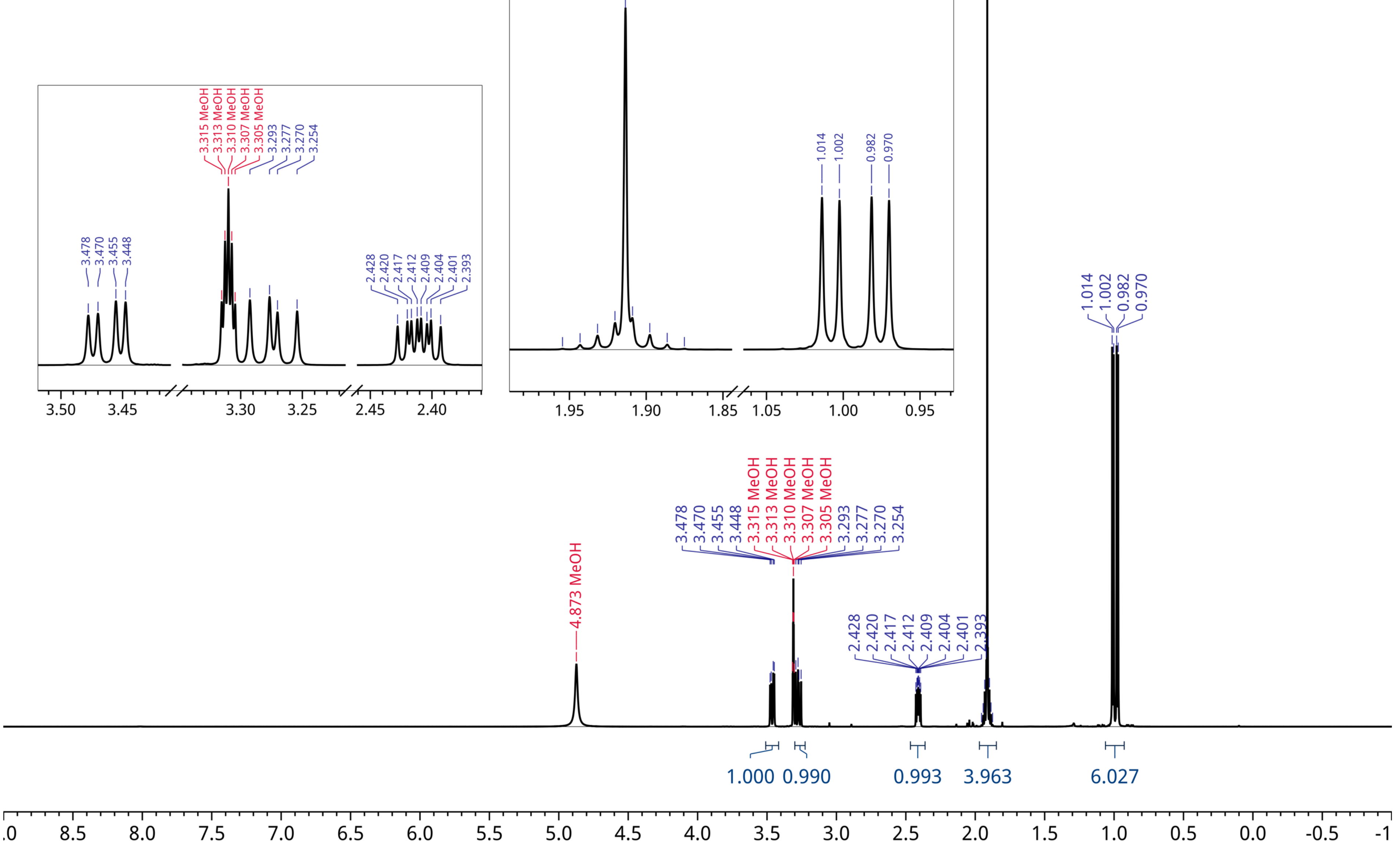

N-acetyl-β^2-*L*-homovaline (**Ac-β^2-*L*-Val-OH**)

$^{13}C\{^1H\}$ NMR (151 MHz, MeOD-d_4) δ 177.50, 173.39, 53.07, 40.36, 30.01, 22.46, 20.56, 20.35.

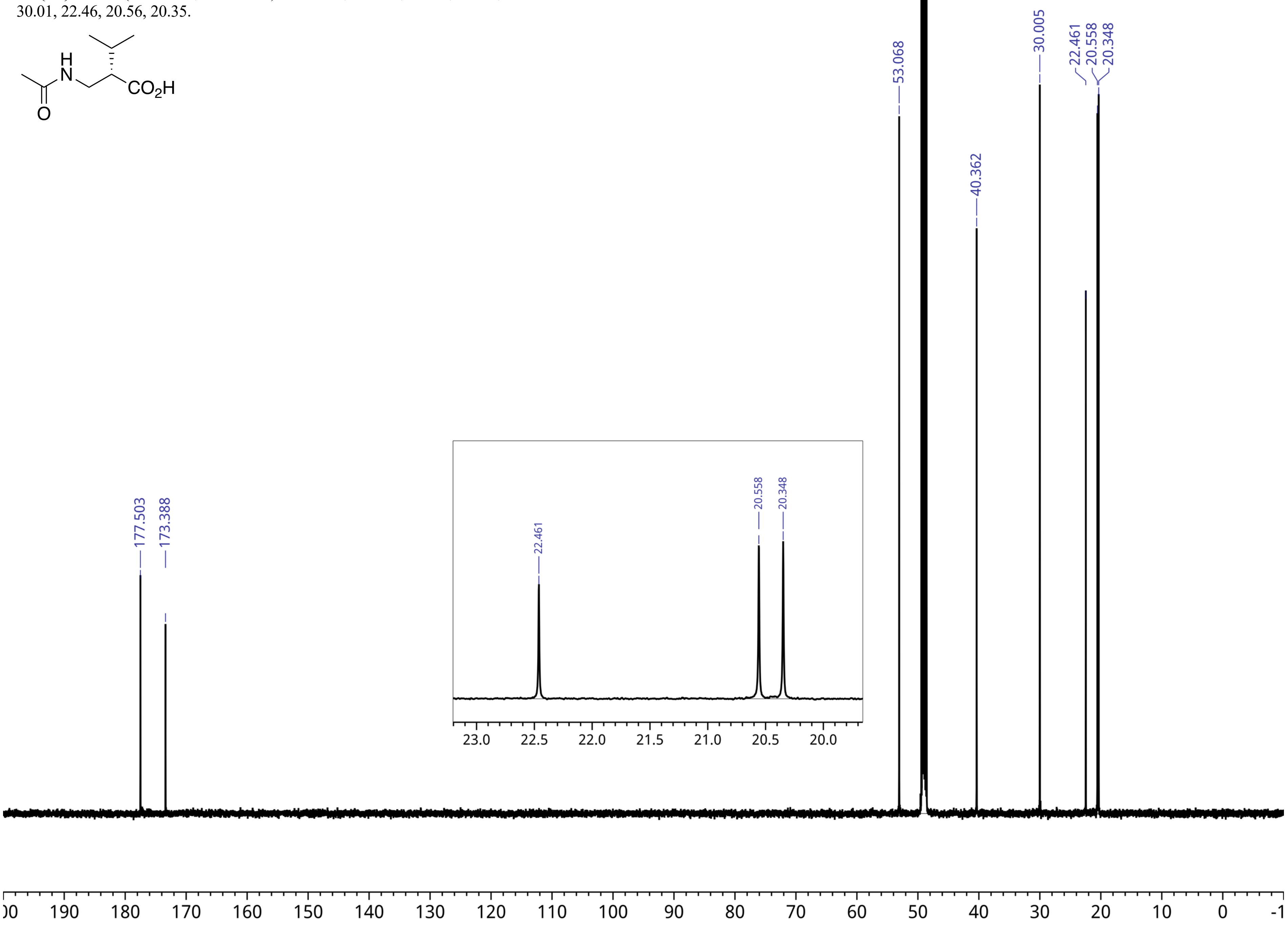

N-acetyl-β^2-*D*-homovaline (**Ac-β^2-*D*-Val-OH**)

^{1}H NMR (600 MHz, MeOD-d_4) δ 3.46 (dd, J = 13.4, 4.7 Hz, 1H), 3.27 (dd, J = 13.4, 9.5 Hz, 1H), 2.41 (ddd, J = 9.5, 6.7, 4.7 Hz, 1H), 1.91 (heptd, J = 6.8, 6.8 Hz, 1H), 1.91 (s, 3H), 0.99 (dd, J = 19.4, 6.8 Hz, 6H).

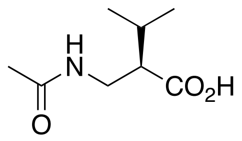

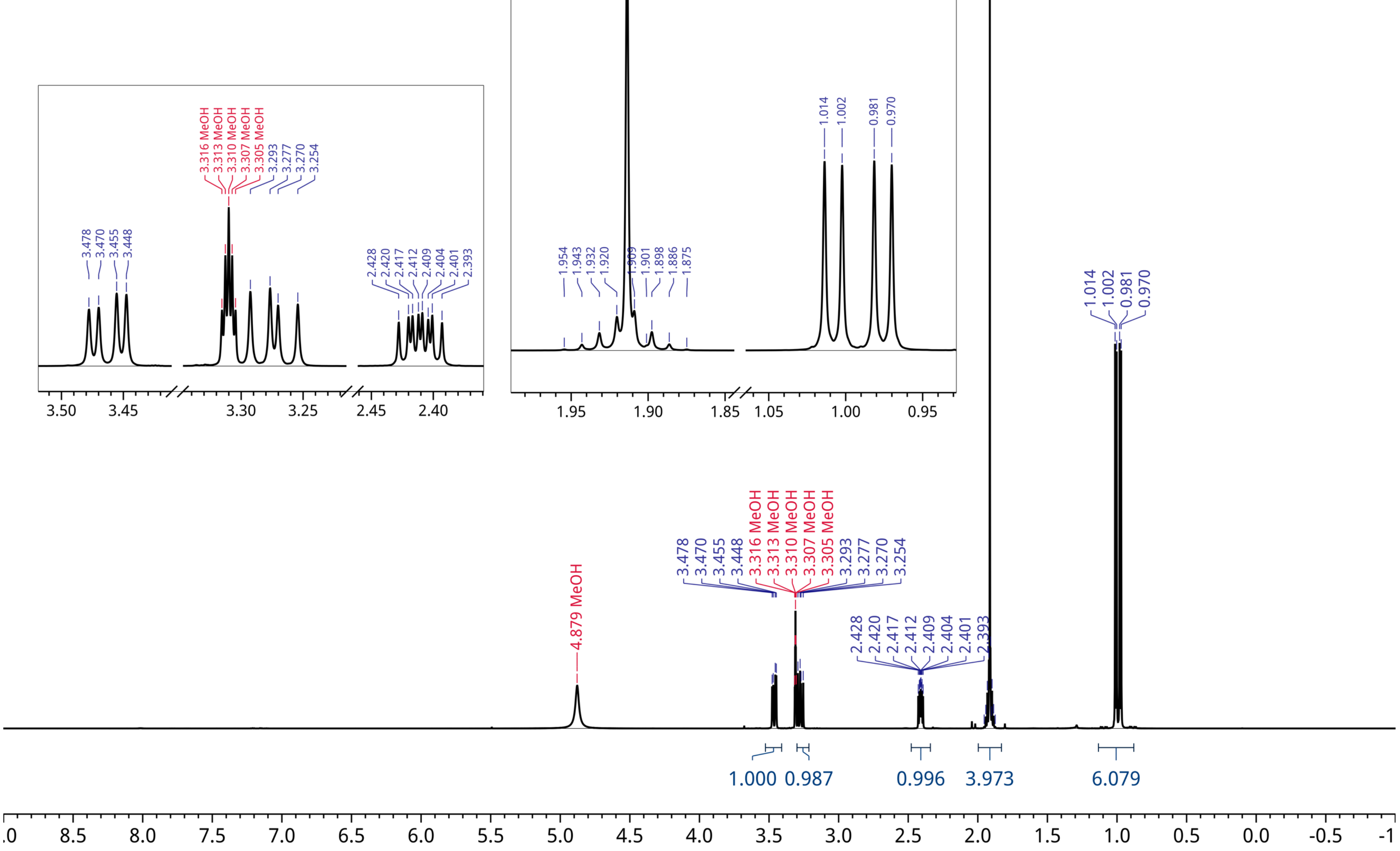

N-acetyl-β^2-*D*-homovaline (Ac-β^2-*D*-Val-OH)

$^{13}C\{^1H\}$ NMR (151 MHz, MeOD-d_4) δ 177.50, 173.39, 53.07, 40.36, 30.00, 22.46, 20.56, 20.35.

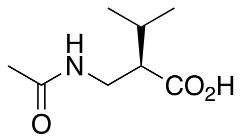

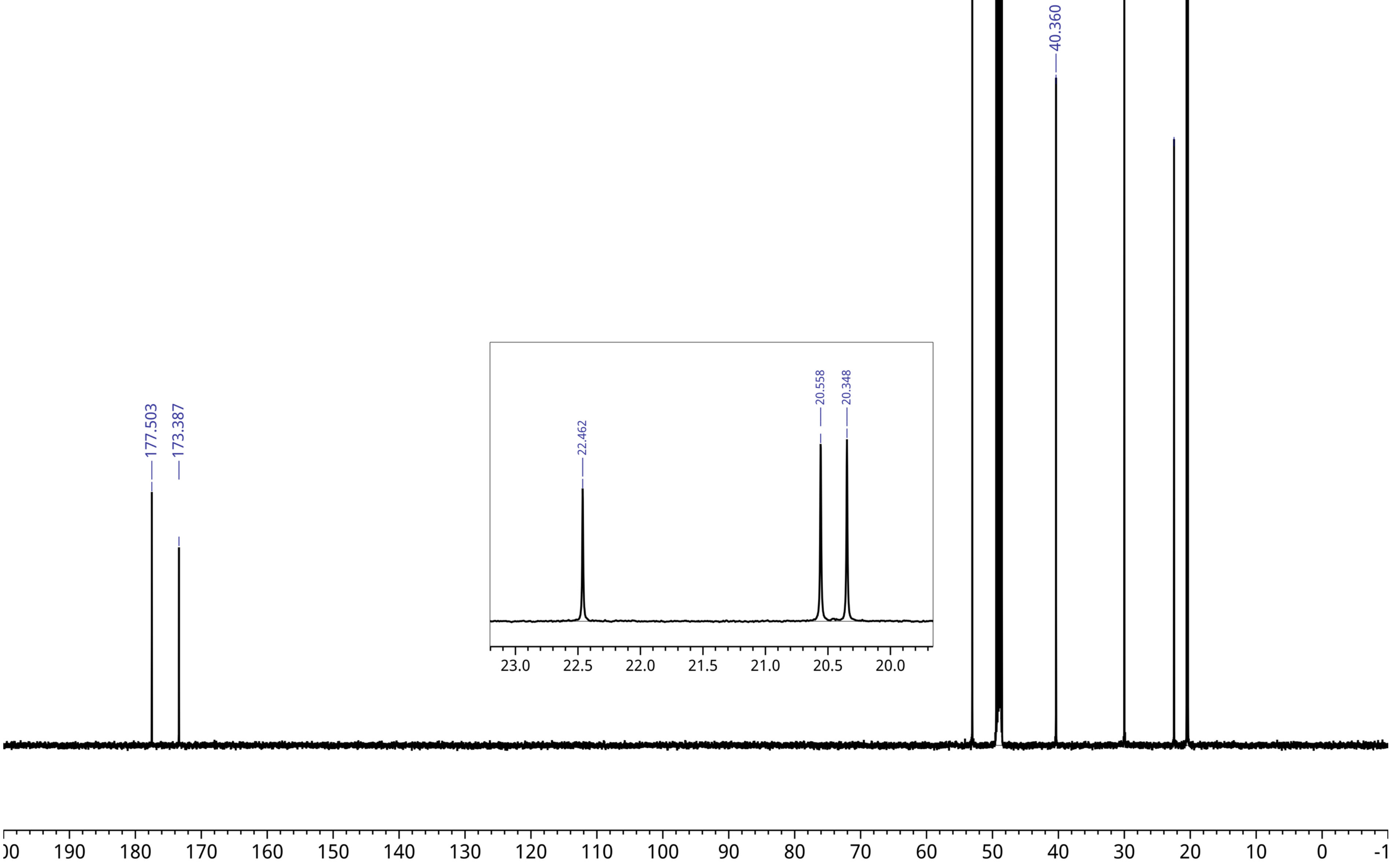

N-acetyl-2,2-dimethyl-*β*-alanine (**Ac-*β*²-Me₂-OH**)

^{1}H NMR (600 MHz, MeOD-d_4) δ 3.34 (s, 2H), 1.95 (s, 3H), 1.16 (s, 3H).

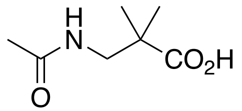

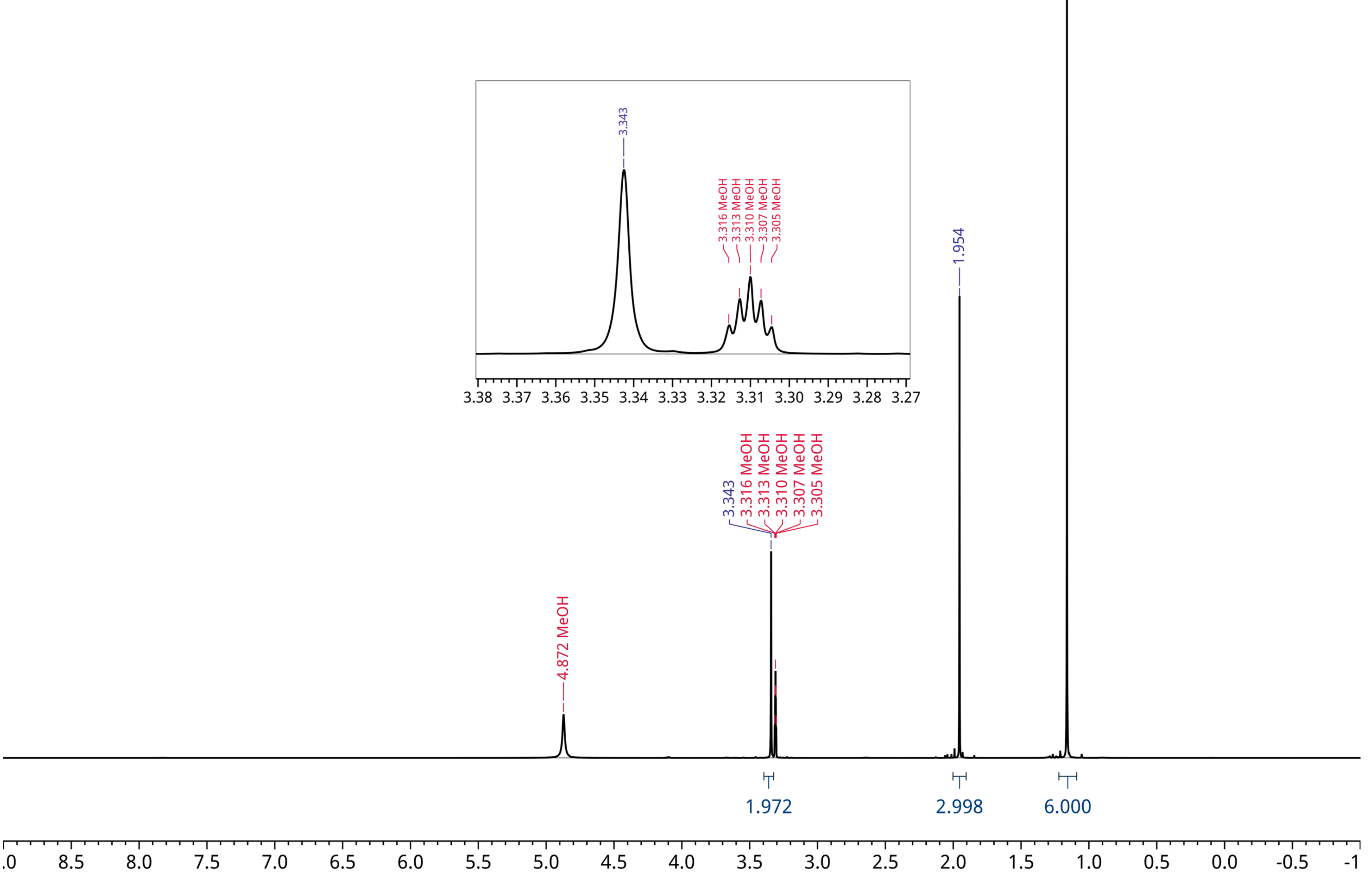

N-acetyl-2,2-dimethyl-*β*-alanine (**Ac-*β*[2]-Me$_2$-OH**)

$^{13}C\{^{1}H\}$ NMR (151 MHz, MeOD-d_4) δ 180.23, 173.59, 47.97, 44.22, 23.53, 22.52.

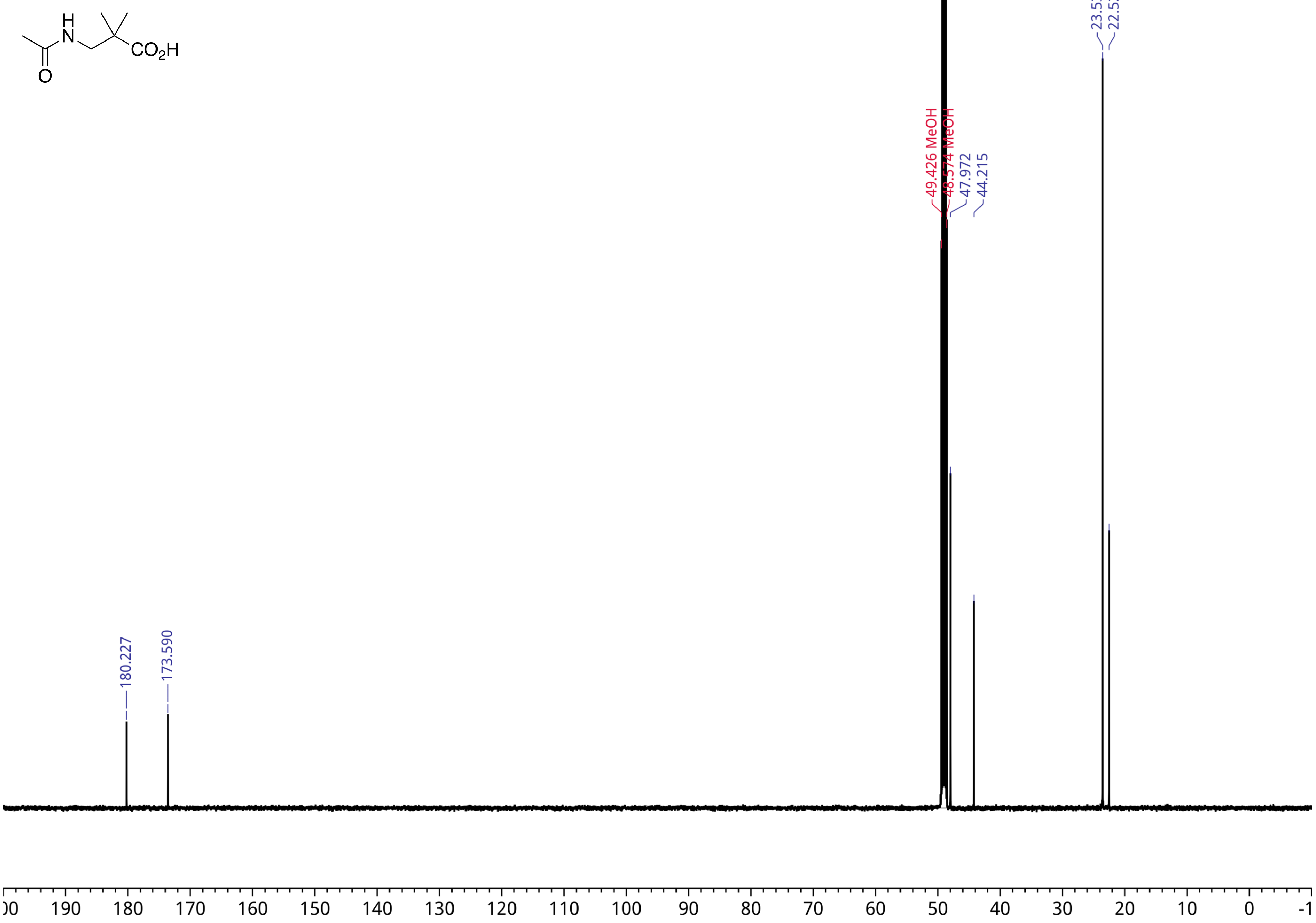

N-acetyl-β^3-*L*-leucine (**Ac-β^3-*L*-Val-OH**)

^{1}H NMR (600 MHz, MeOD-d_4) δ 4.11 (ddd, J = 8.8, 6.0, 4.8 Hz, 1H), 2.51 (dd, J = 15.3, 4.8 Hz, 1H), 2.37 (dd, J = 15.3, 8.9 Hz, 1H), 1.94 (s, 3H), 1.81 (dh, J = 13.7, 6.8 Hz, 1H), 0.92 (dd, J = 6.8, 3.9 Hz, 6H).

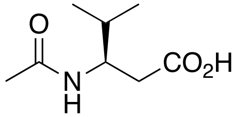

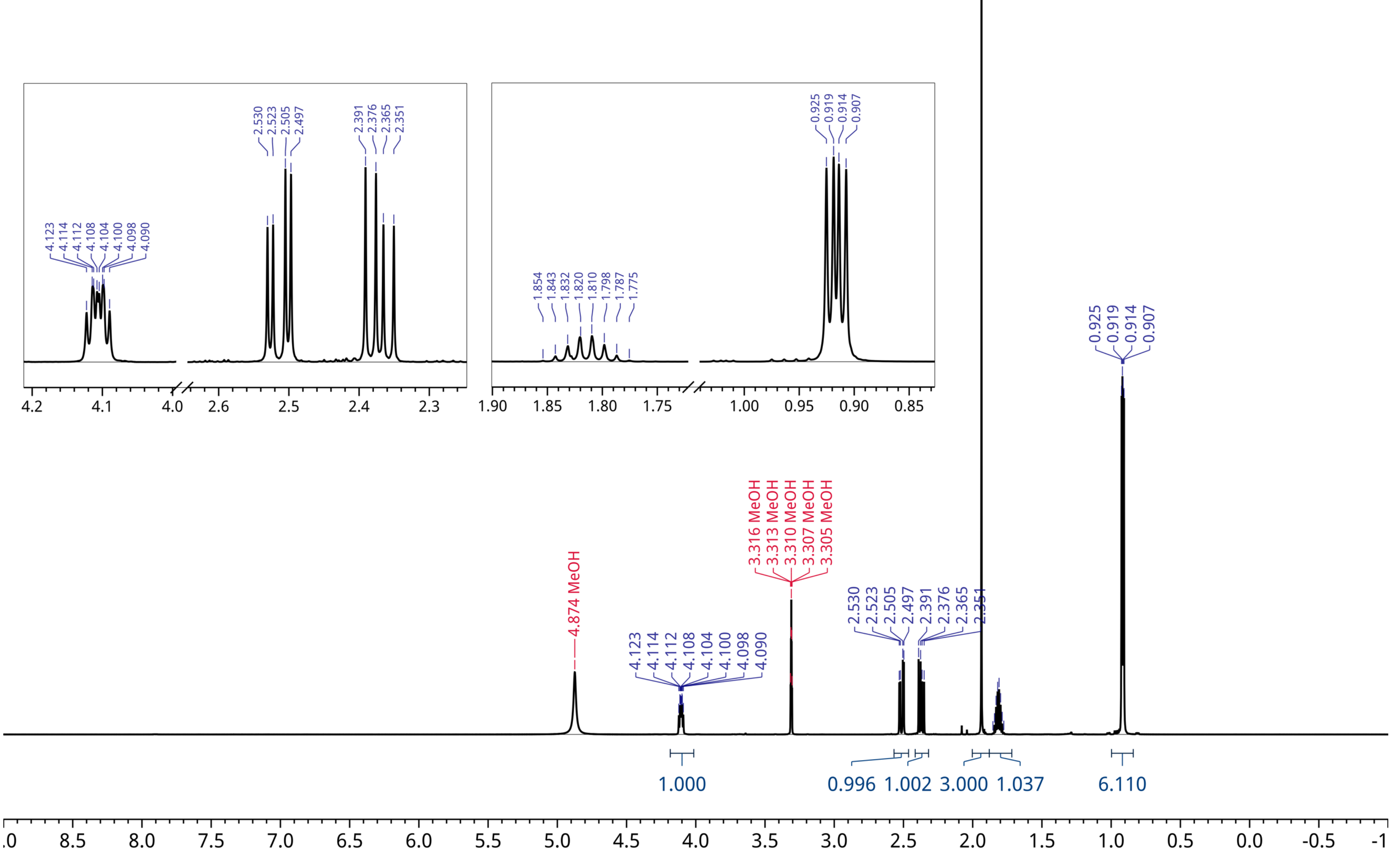

N-acetyl-*β*[3]-*L*-leucine (**Ac-*β*[3]-*L*-Val-OH**)

$^{13}C\{^1H\}$ NMR (151 MHz, MeOD-*d*$_4$) δ 175.21, 172.84, 53.00, 37.60, 33.09, 22.58, 19.42, 18.49.

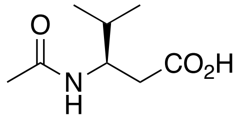

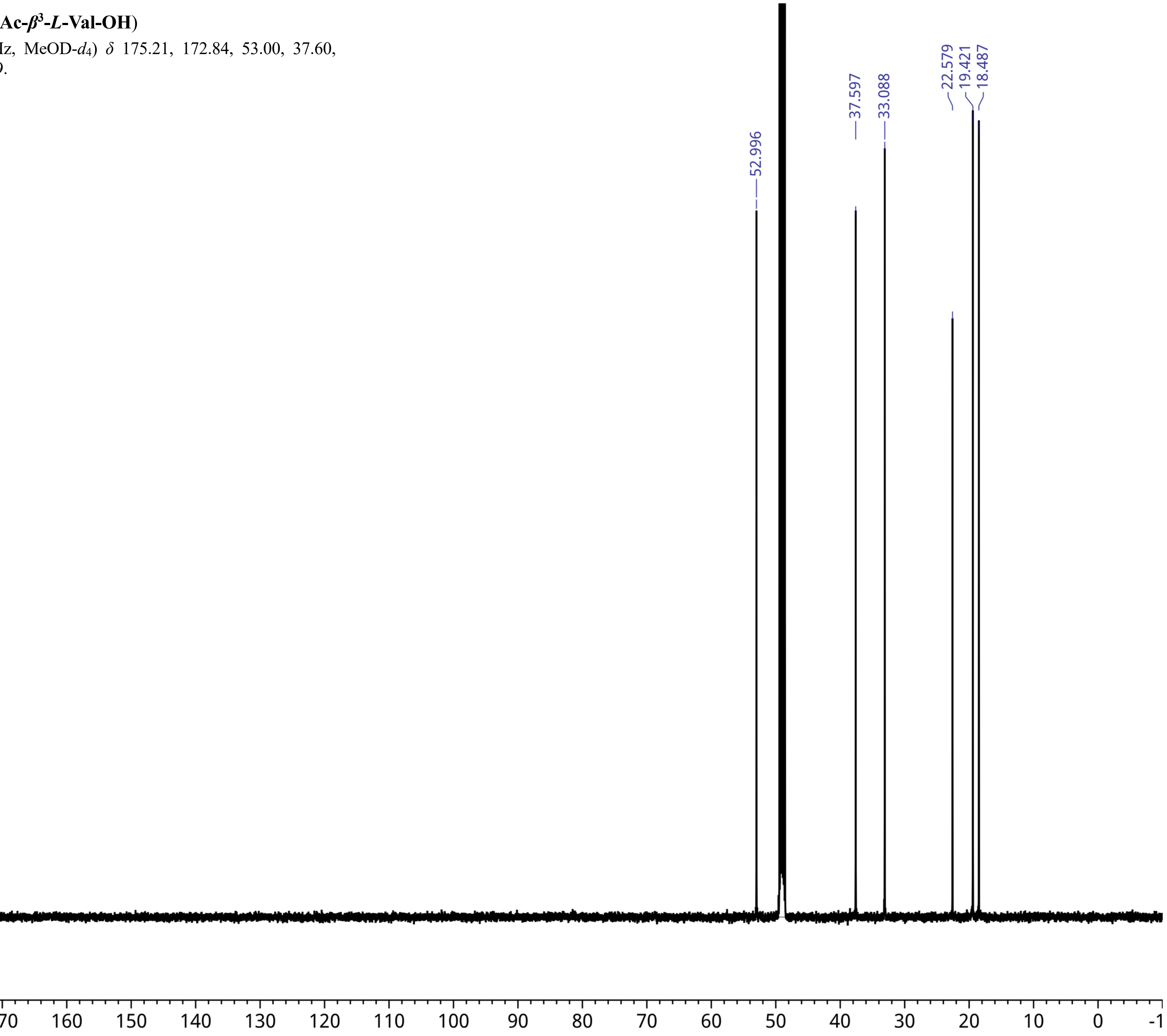

N-acetyl-β^3-*D*-leucine (**Ac-*β*³-*D*-Val-OH**)

^{1}H NMR (600 MHz, MeOD-d_4) δ 4.11 (ddd, J = 8.8, 6.1, 4.8 Hz, 1H), 2.51 (dd, J = 15.3, 4.8 Hz, 1H), 2.37 (dd, J = 15.3, 8.9 Hz, 1H), 1.94 (s, 3H), 1.81 (dh, J = 13.6, 6.8 Hz, 1H), 0.92 (dd, J = 6.8, 3.9 Hz, 6H).

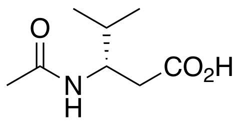

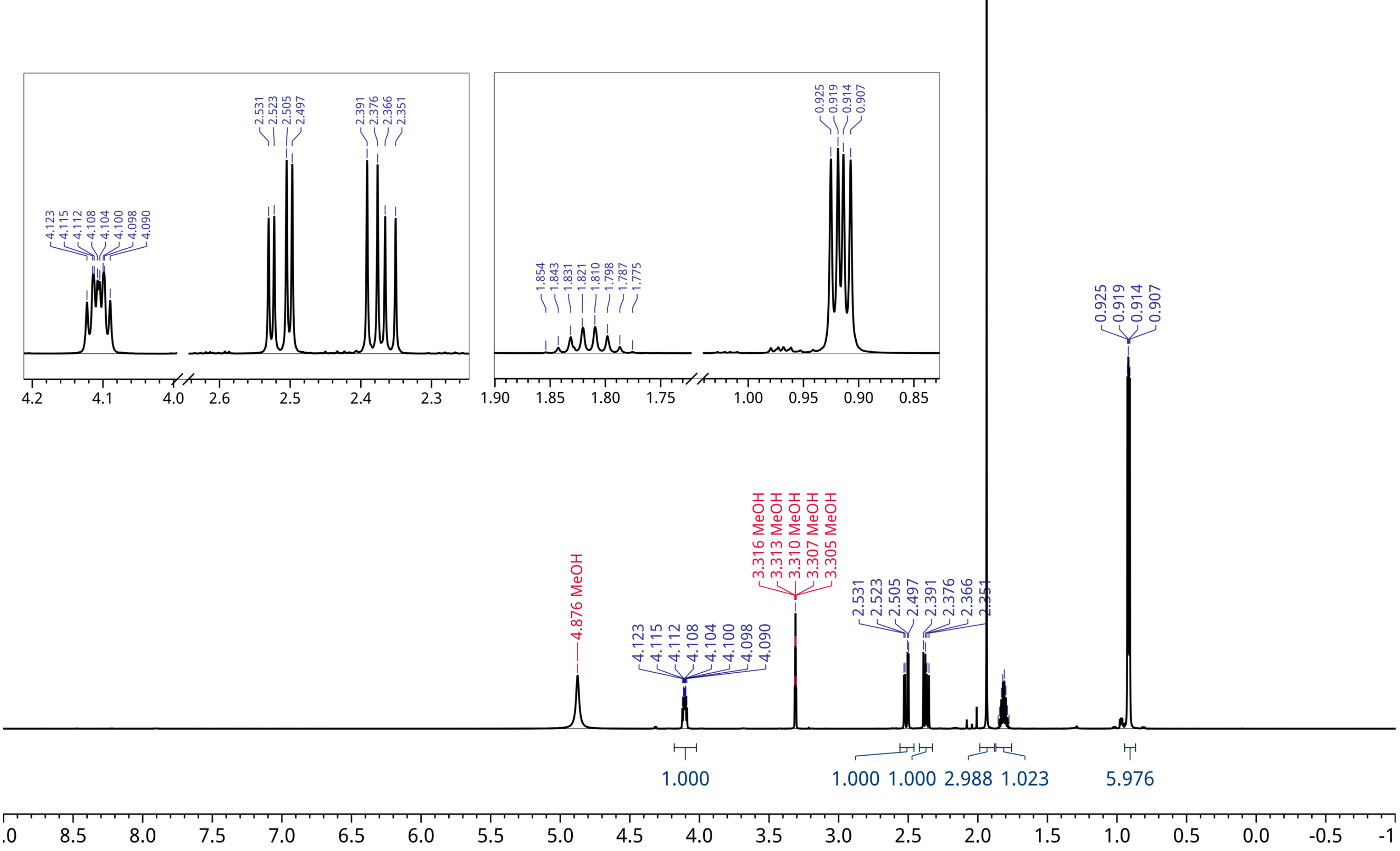

N-acetyl-β^3-*D*-leucine (**Ac-β^3-*D*-Val-OH**)

$^{13}C\{^1H\}$ NMR (151 MHz, MeOD-d_4) δ 175.20, 172.84, 52.99, 37.59, 33.09, 22.58, 19.42, 18.49.

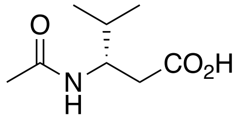

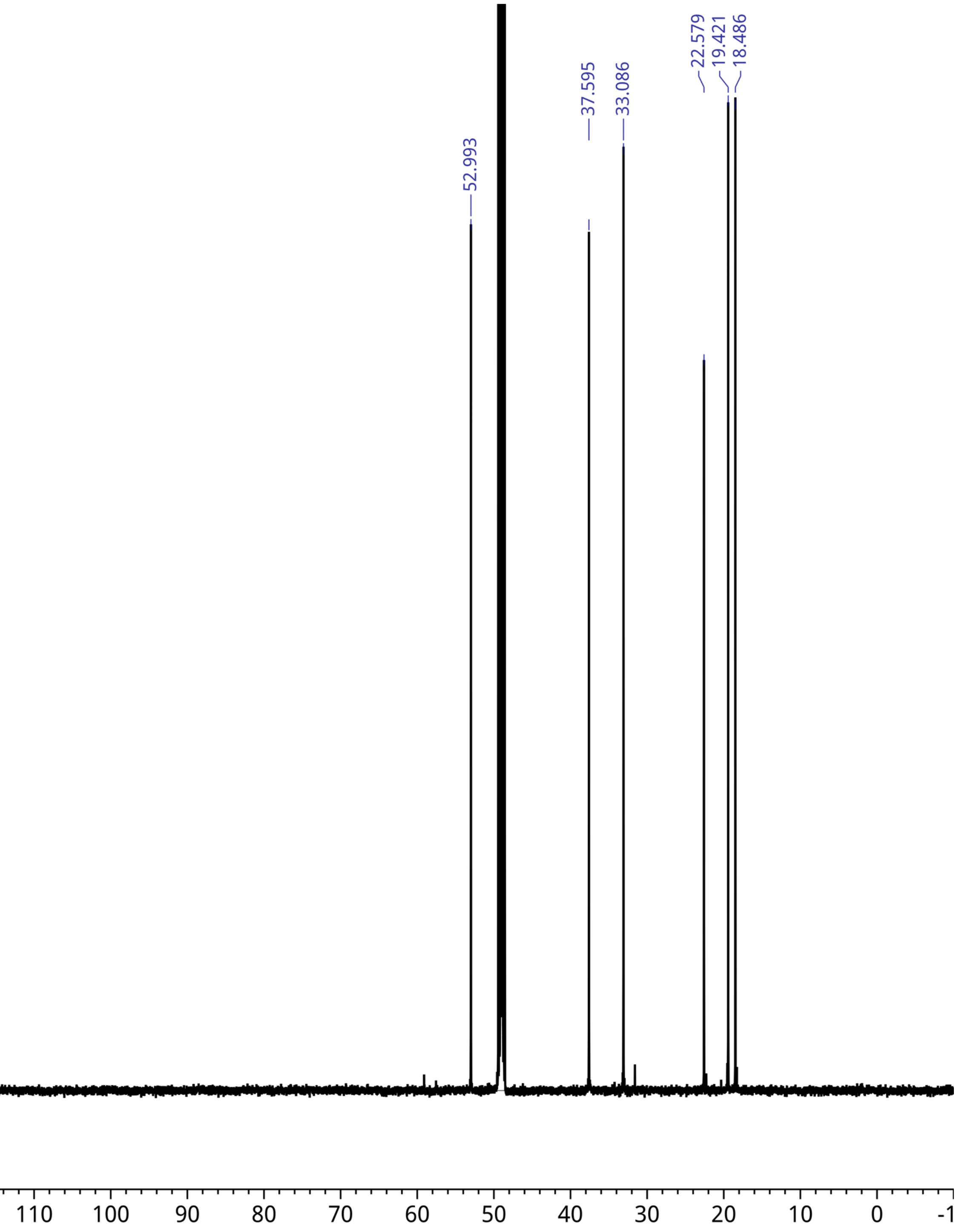

N-acetyl-*β*[3]-*L*-phenylalanine (**Ac-*β*[3]-*L*-Ph-OH**)

^{1}H NMR (600 MHz, MeOD-d_4) δ 7.39 – 7.28 (m, 4H), 7.27 – 7.21 (m, 1H), 5.34 (dd, *J* = 8.4, 6.5 Hz, 1H), 2.84 – 2.71 (m, 2H), 1.95 (s, 3H).

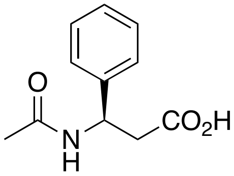

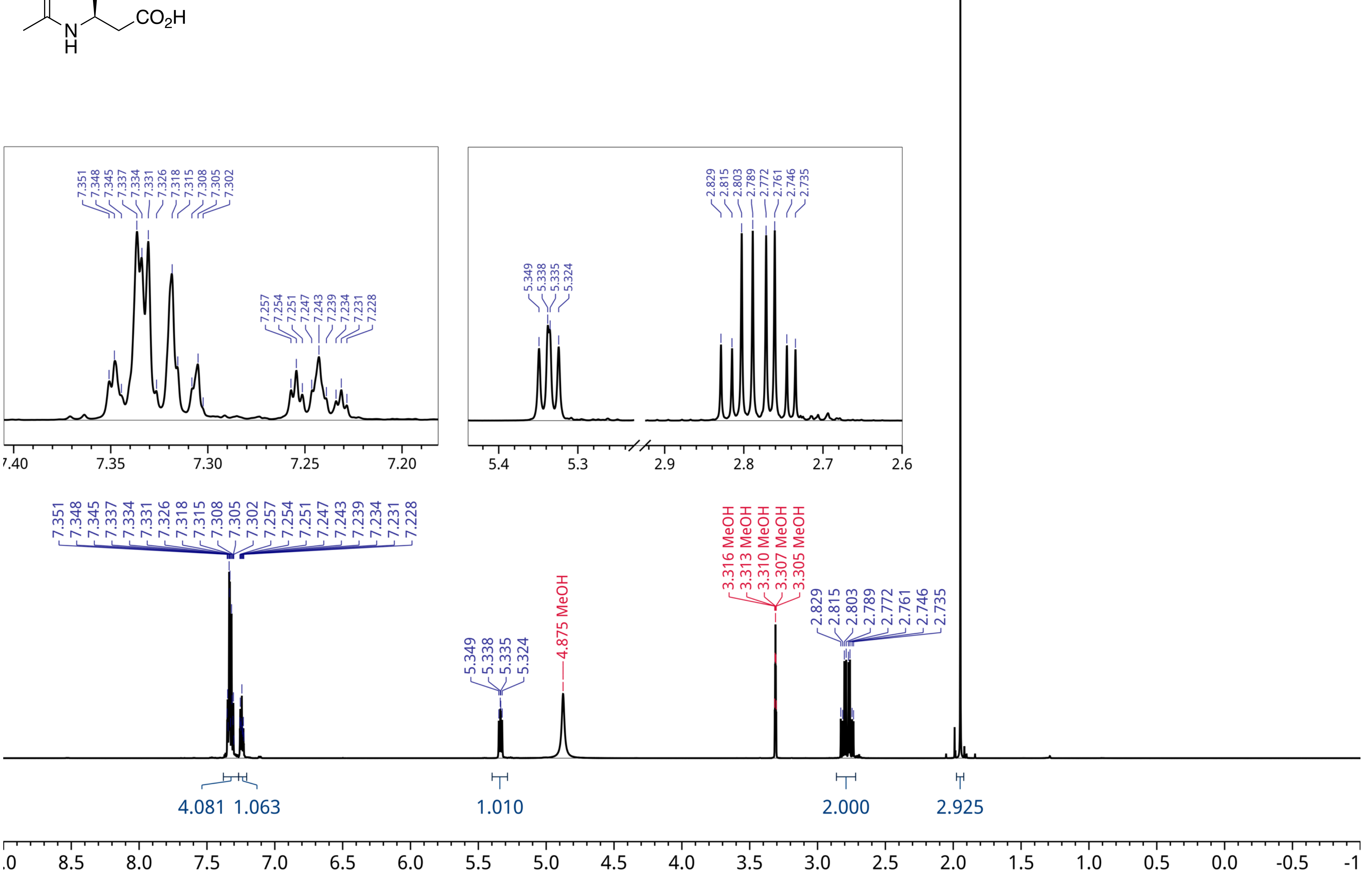

N-acetyl-*β*[3]-*L*-phenylalanine (**Ac-*β*[3]-*L*-Ph-OH**)

$^{13}C\{^1H\}$ NMR (151 MHz, MeOD-d_4) δ 174.05, 172.37, 142.91, 129.59, 128.46, 127.62, 51.58, 41.62, 22.59.

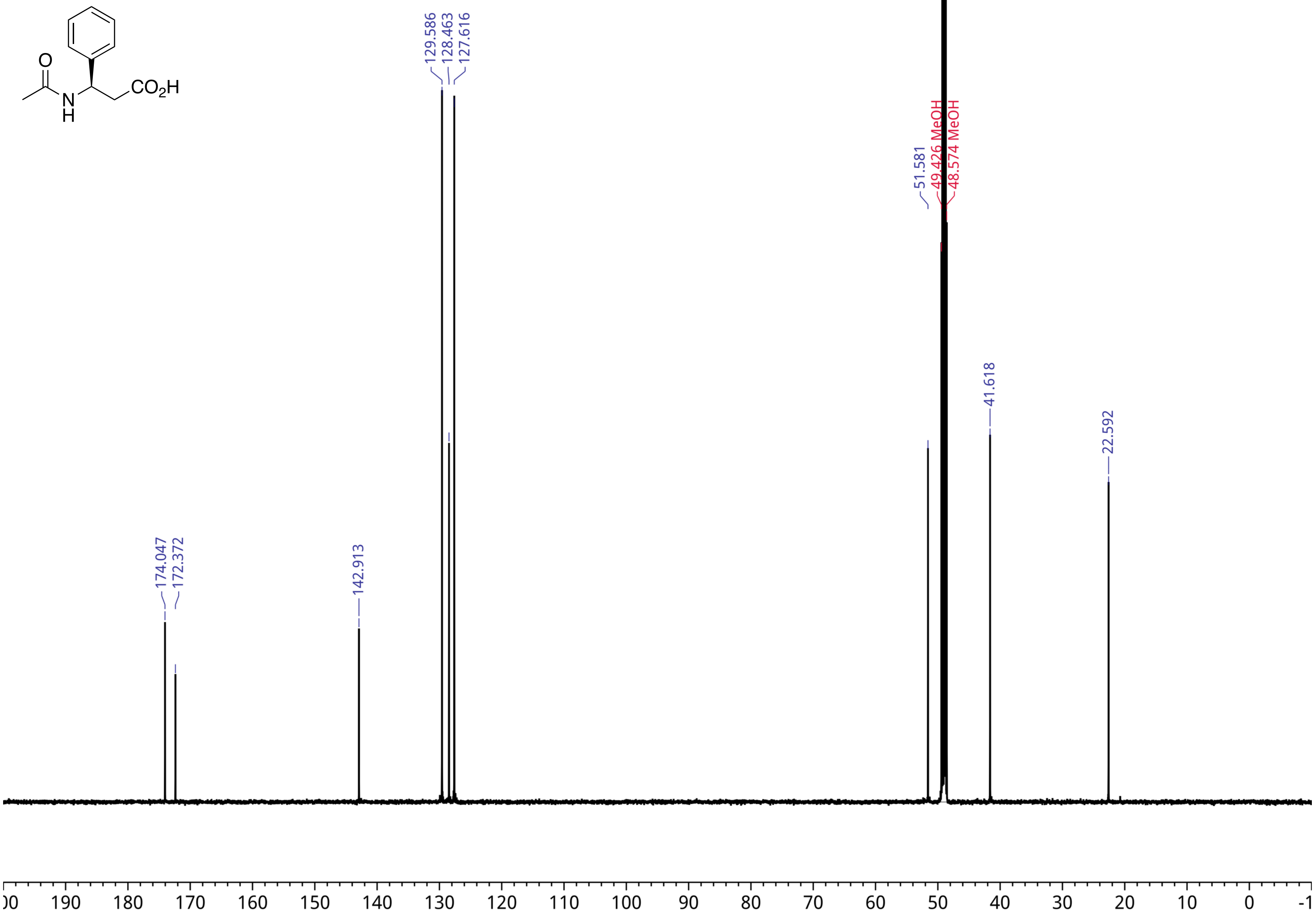

N-acetyl-*β*[3]-*L*-homophenylalanine (**Ac-*β*[3]-*L*-Phe-OH**)

^{1}H NMR (600 MHz, MeOD-d_4) δ 7.28 (m, 2H), 7.24 – 7.17 (m, 3H), 4.42 (ddd, *J* = 13.8, 7.5, 6.2 Hz, 1H), 2.89 – 2.77 (m, 2H), 2.51 – 2.39 (m, 2H), 1.85 (s, 3H).

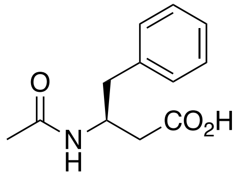

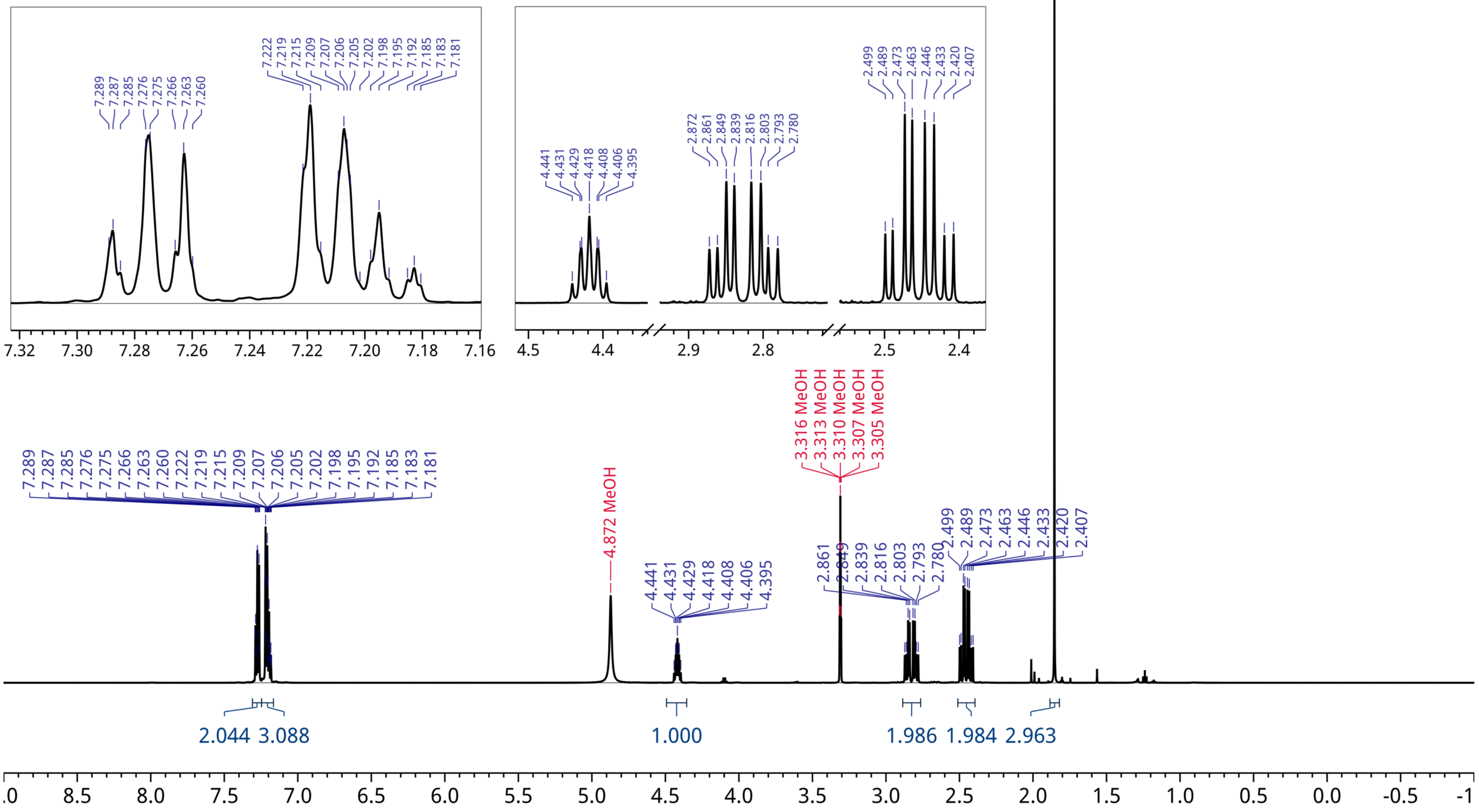

N-acetyl-β^3-*L*-homophenylalanine (**Ac-β^3-*L*-Phe-OH**)

$^{13}C\{^1H\}$ NMR (151 MHz, MeOD-d_4) δ 174.72, 172.61, 139.46, 130.39, 129.39, 127.53, 49.40, 41.08, 39.19, 22.57.

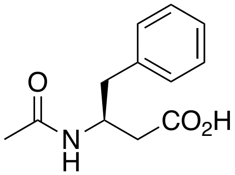

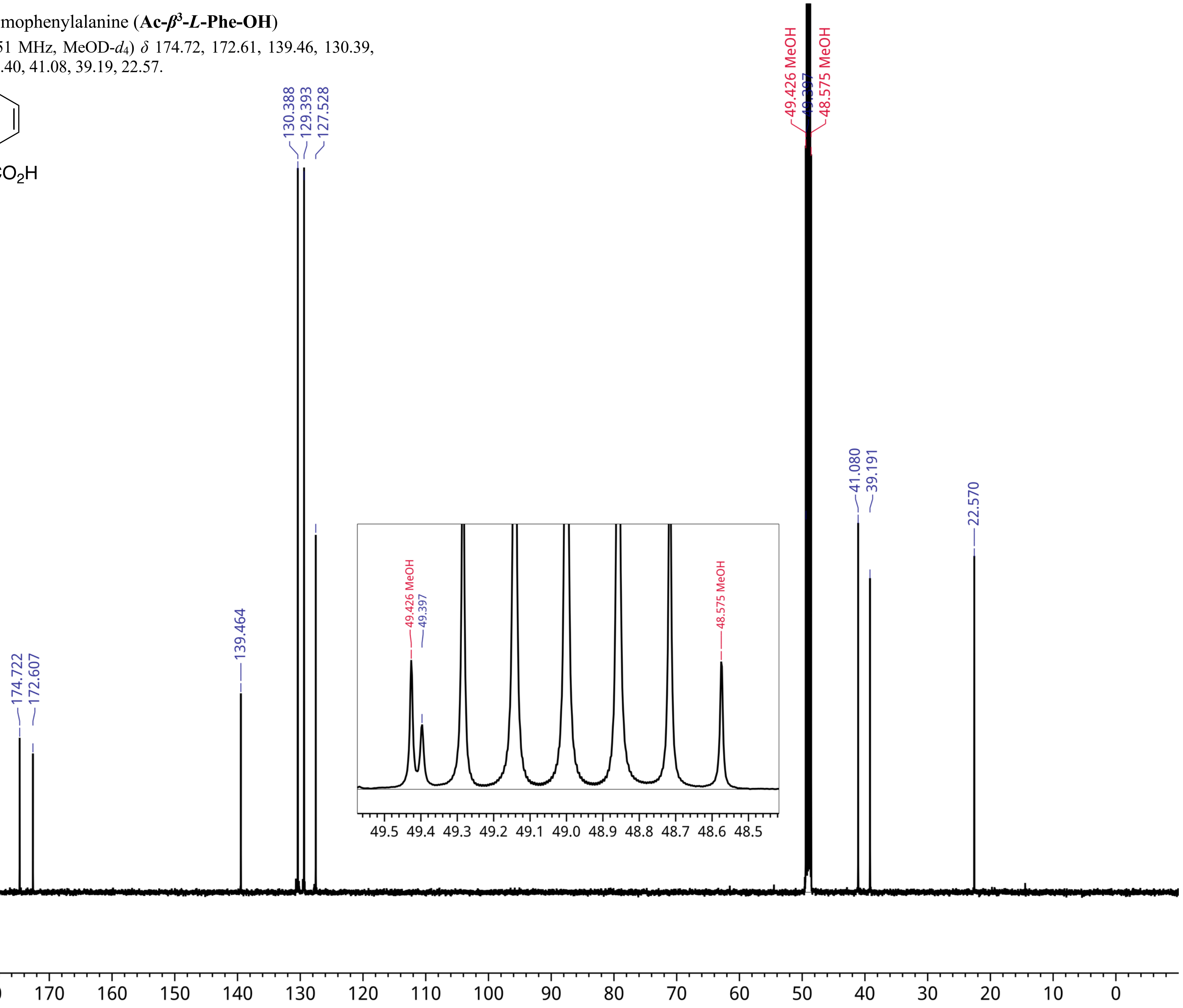

N-acetyl-3,3-dimethyl-*β*-alanine (**Ac-*β*³-Me₂-OH**)

^{1}H NMR (600 MHz, MeOD-d_4) δ 2.79 (s, 2H), 1.88 (s, 3H), 1.39 (s, 6H).

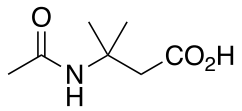

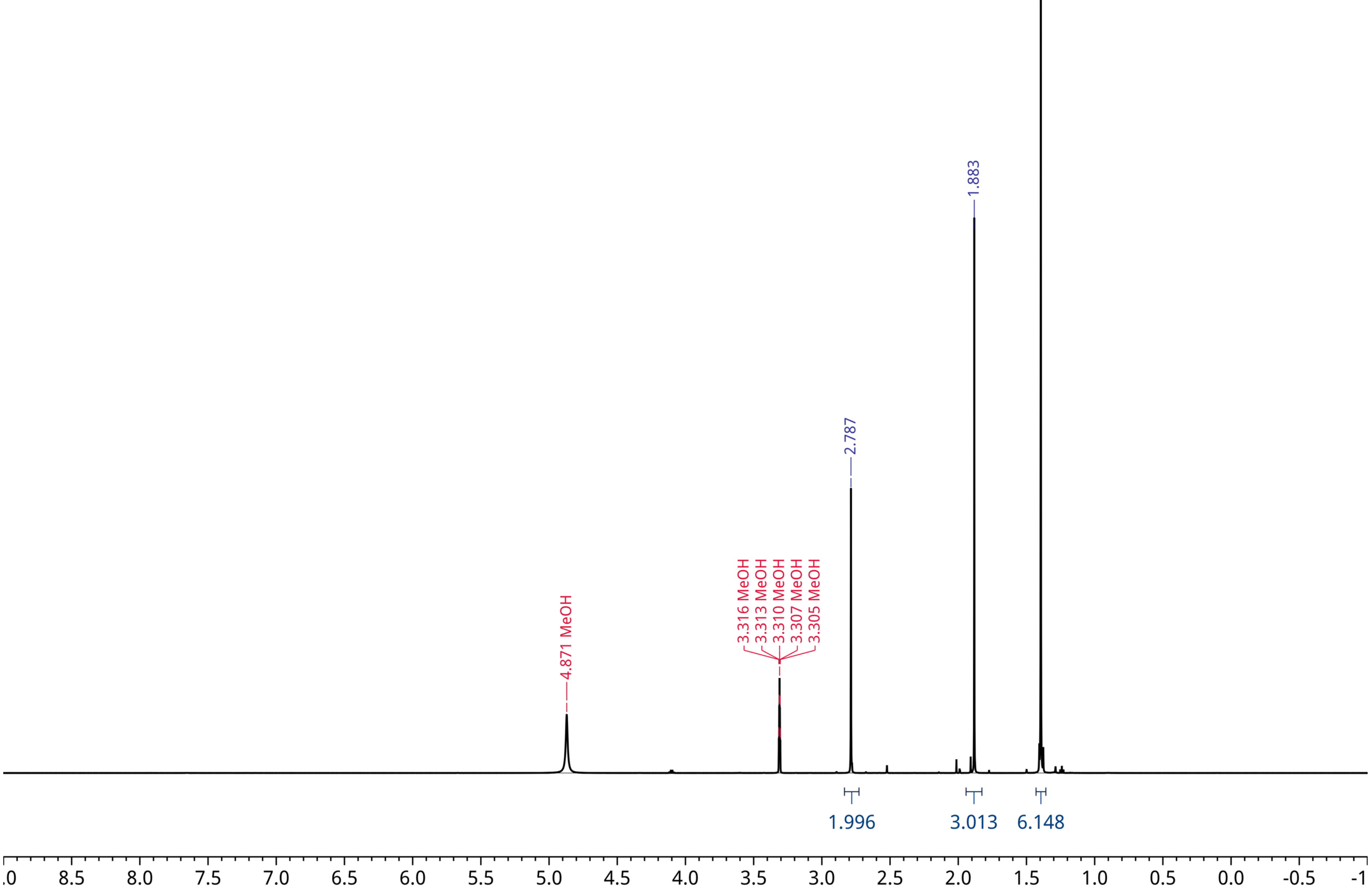

N-acetyl-3,3-dimethyl-*β*-alanine (**Ac-*β*³-Me₂-OH**)

$^{13}C\{^{1}H\}$ NMR (151 MHz, MeOD-d_4) δ 174.78, 172.98, 52.77, 43.93, 27.44, 23.53.

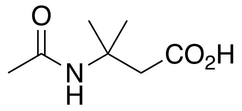

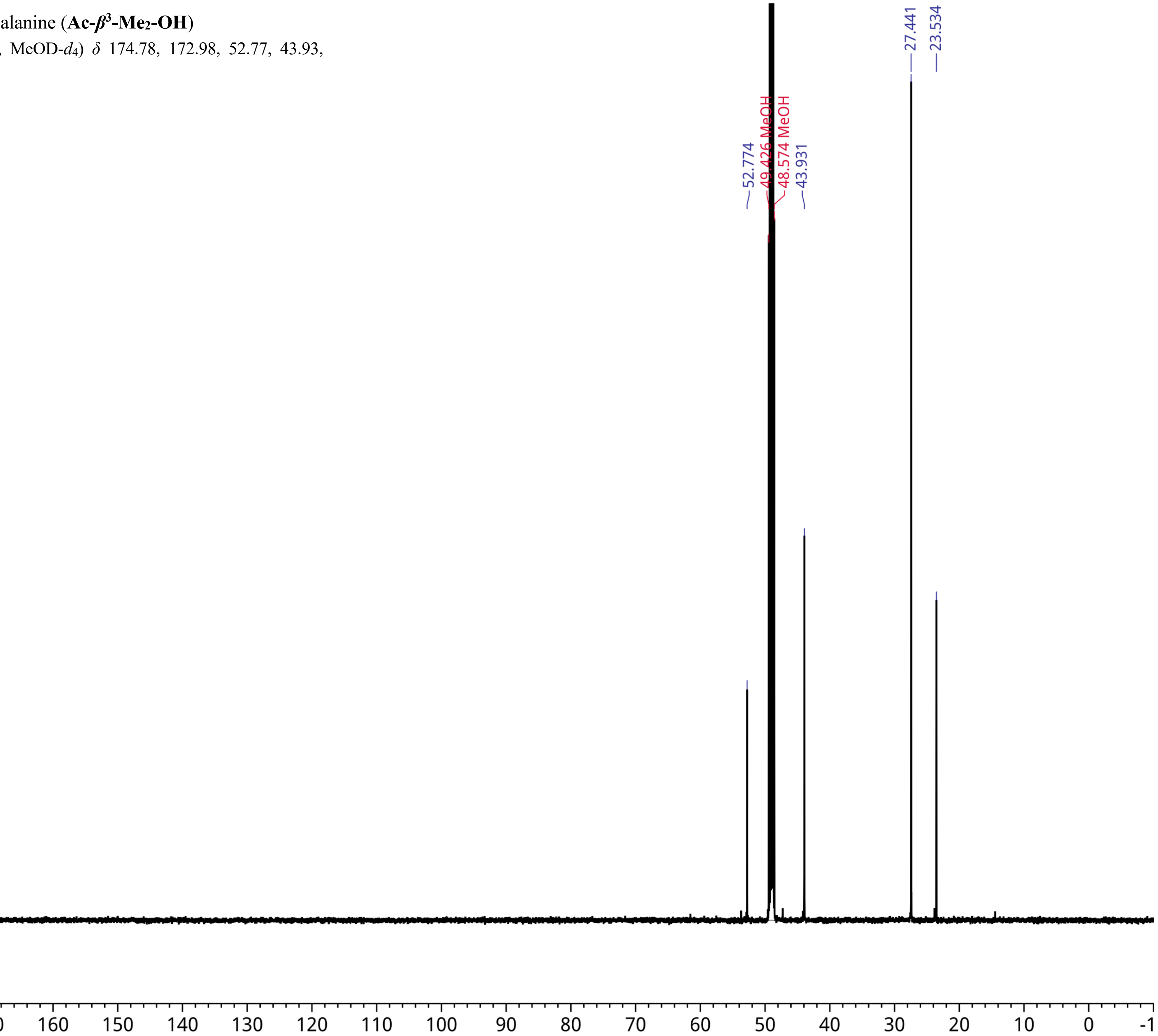

4-methylpyridine supported 5-membered palladacycle (**1a-PdCycle-MePy**)

^{1}H NMR (600 MHz, $CDCl_3$) δ 8.83 – 8.79 (m, 2H), 8.10 – 8.06 (m, 2H), 7.36 – 7.32 (m, 3H), 6.96 (d, J = 5.7 Hz, 2H), 6.87 (t, J = 7.7 Hz, 1H), 6.15 (d, J = 7.6 Hz, 1H), 2.49 (s, 3H), 2.26 (s, 3H).

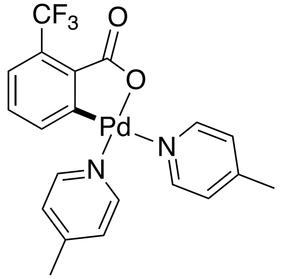

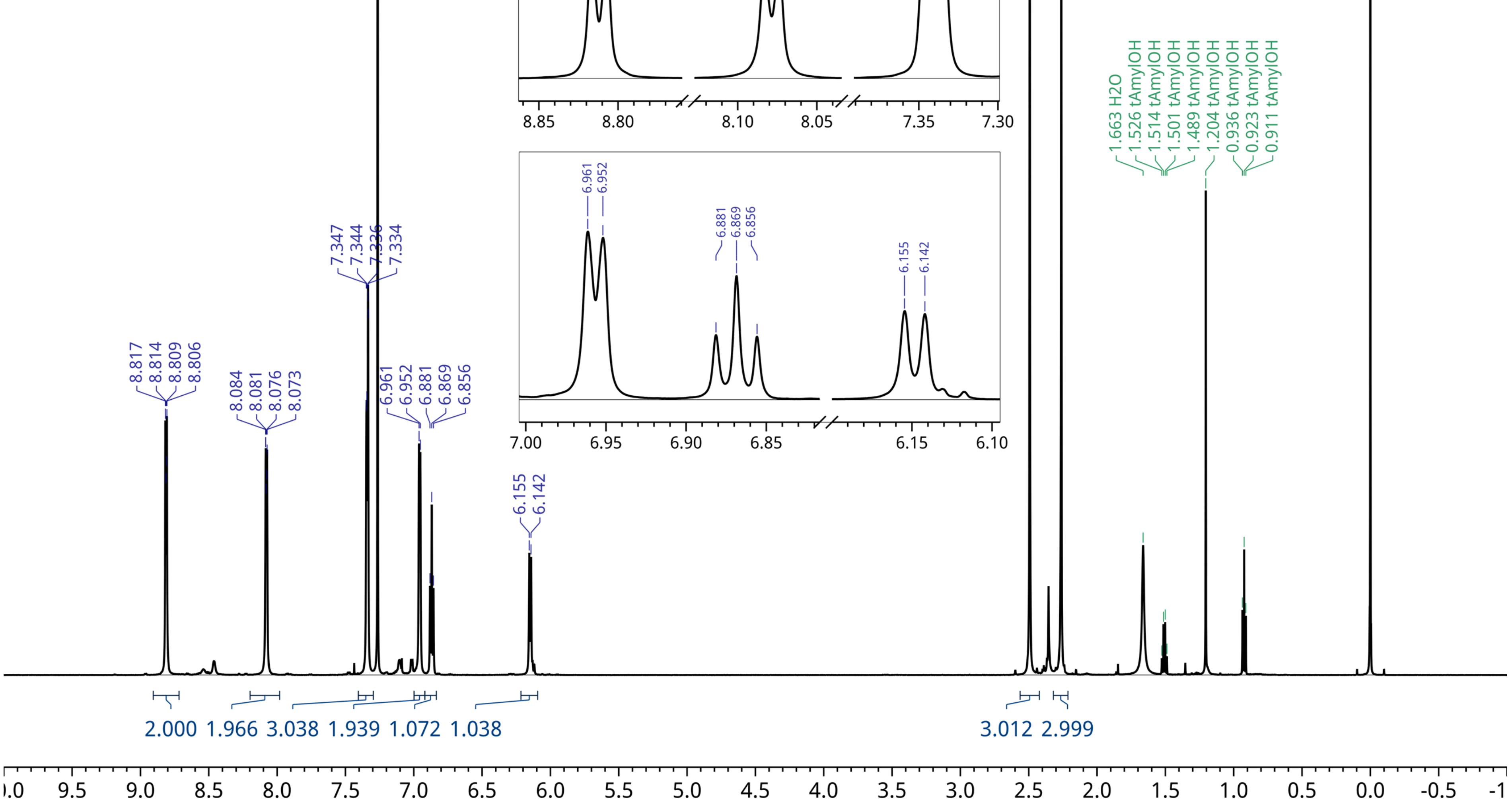

4-methylpyridine supported 5-membered palladacycle (**1a-PdCycle-MePy**)

^{13}C NMR (151 MHz, $CDCl_3$) δ 176.58, 152.43, 151.00, 149.89, 149.14, 147.35, 139.35, 134.84, 128.80 (q, J = 31.4 Hz), 128.19, 127.49, 125.77, 123.32 (q, J = 274.7 Hz), 122.76 (q, J = 6.6 Hz), 21.35, 21.09.

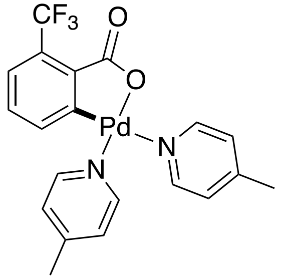

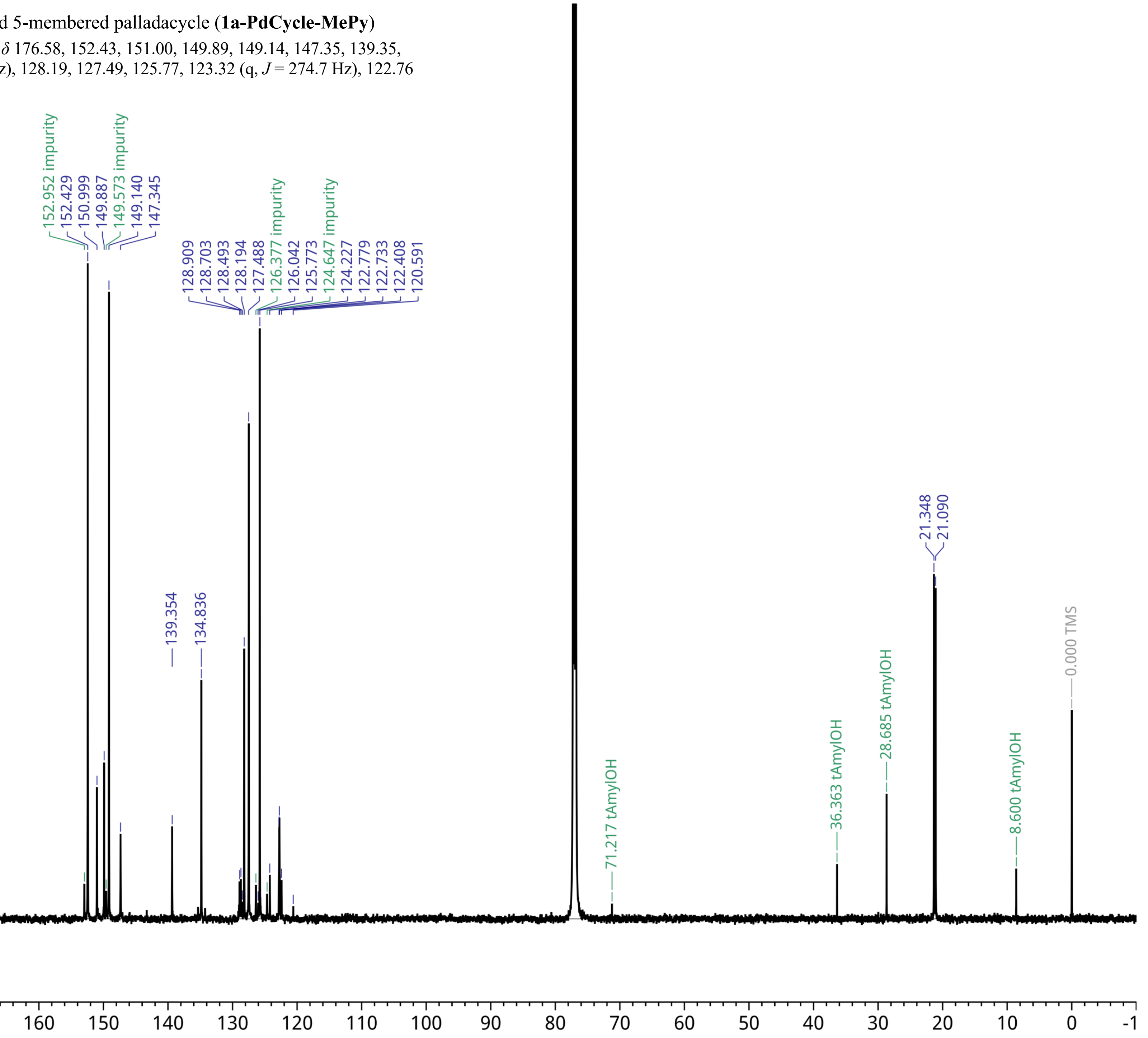

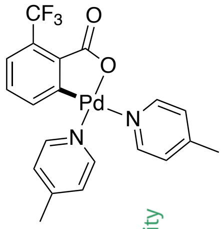

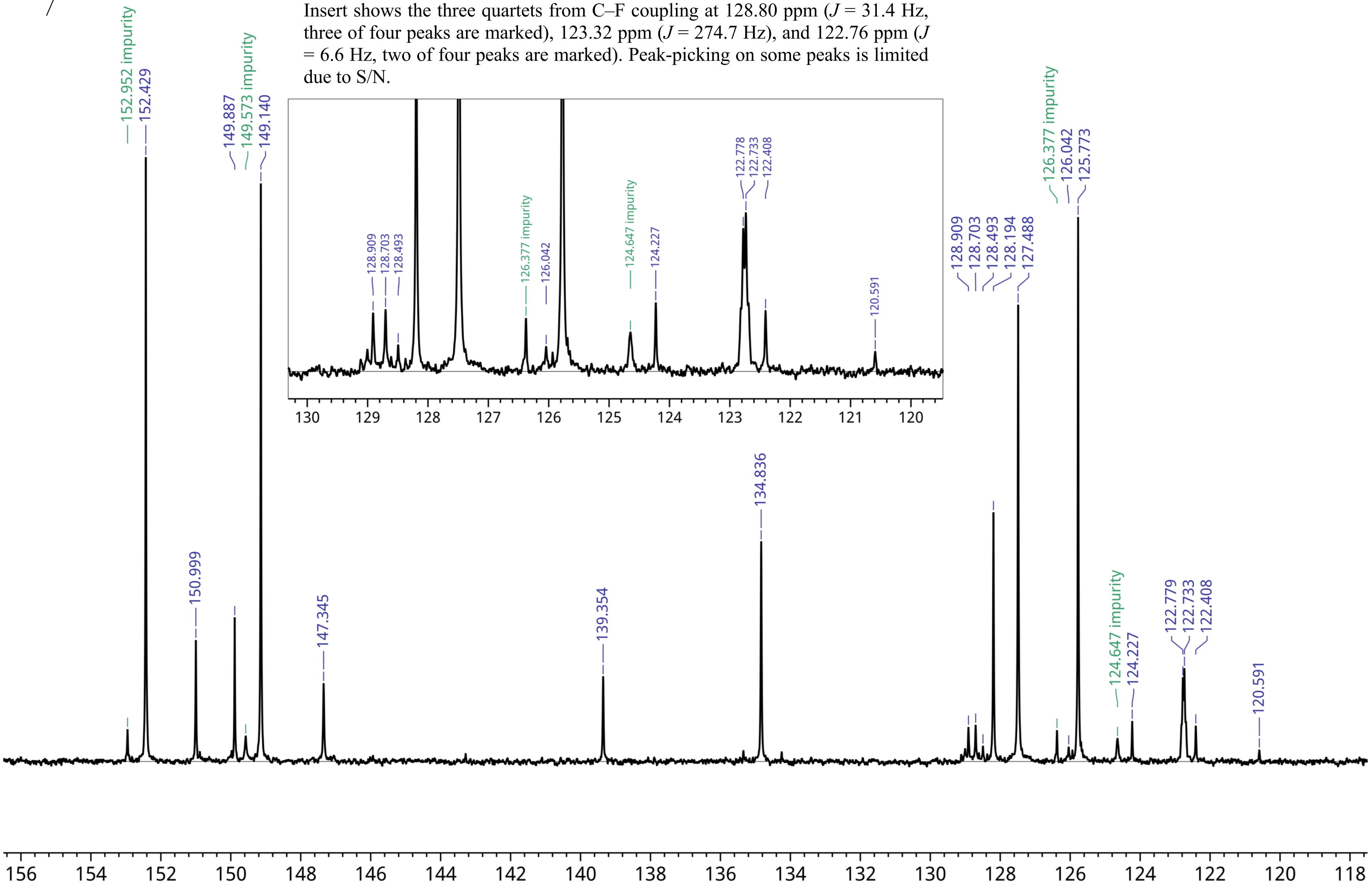

Insert shows the three quartets from C–F coupling at 128.80 ppm (J = 31.4 Hz, three of four peaks are marked), 123.32 ppm (J = 274.7 Hz), and 122.76 ppm (J = 6.6 Hz, two of four peaks are marked). Peak-picking on some peaks is limited due to S/N.

4-methylpyridine supported 5-membered palladacycle (**1a-PdCycle-MePy**)

^{19}F NMR (564 MHz, $CDCl_3$) δ –58.49

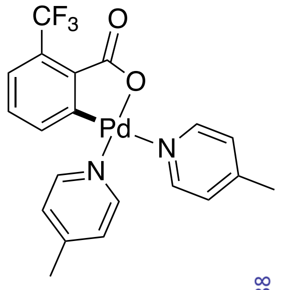

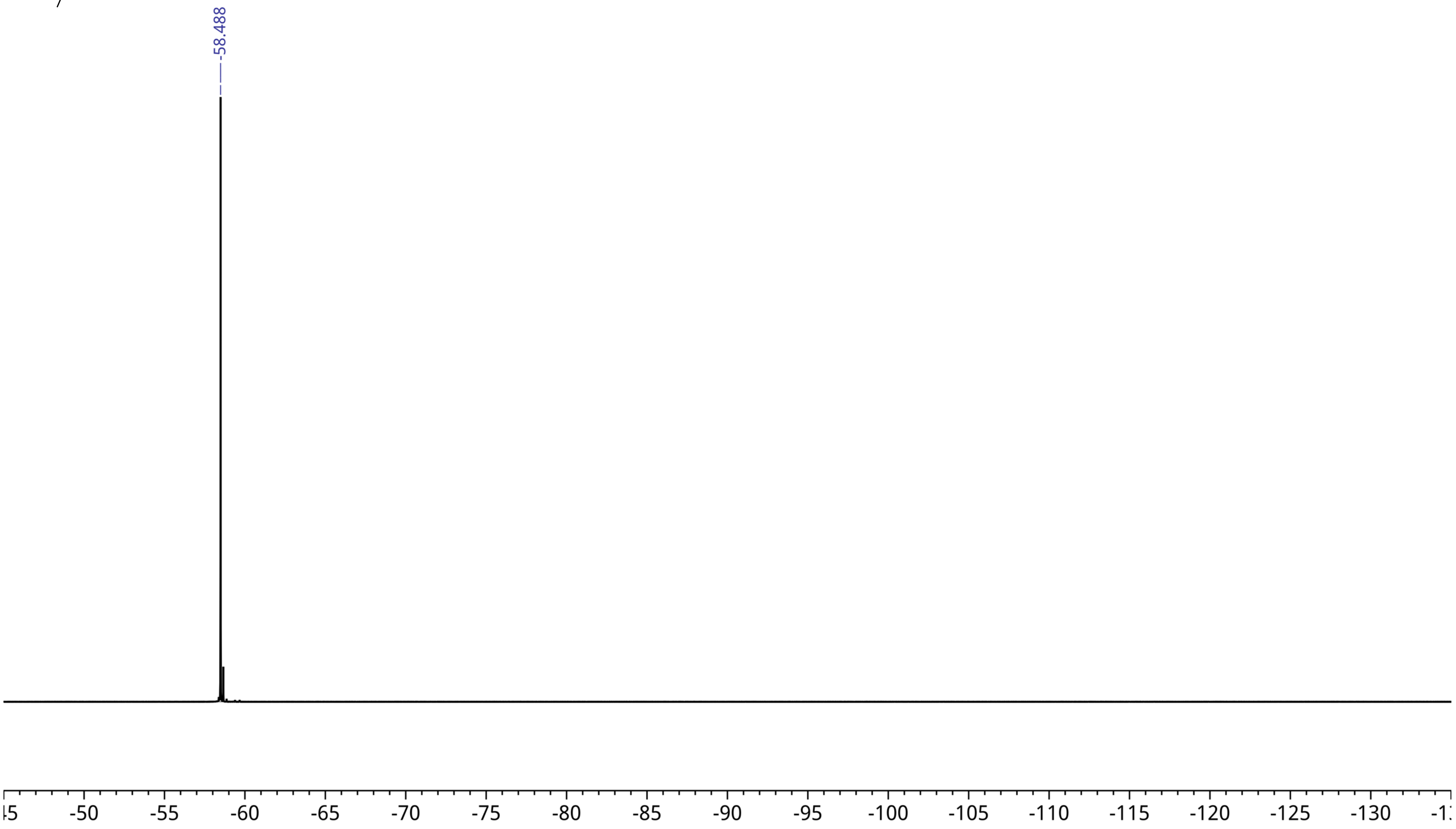

4-*tert*-butylpyridine supported 5-membered palladacycle (**1a-PdCycle-^{t}BuPy**)

^{1}H NMR (600 MHz, $CDCl_3$) δ 8.85 – 8.81 (m, 2H), 8.20 – 8.16 (m, 2H), 7.50 – 7.45 (m, 2H), 7.36 (dd, J = 7.8, 1.1 Hz, 1H), 7.21 – 7.17 (m, 2H), 6.89 (t, J = 7.7 Hz, 1H), 6.17 (dd, J = 7.7, 1.1 Hz, 1H), 1.38 (s, 9H), 1.24 (s, 9H).

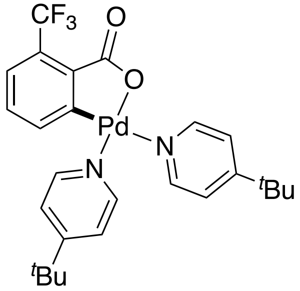

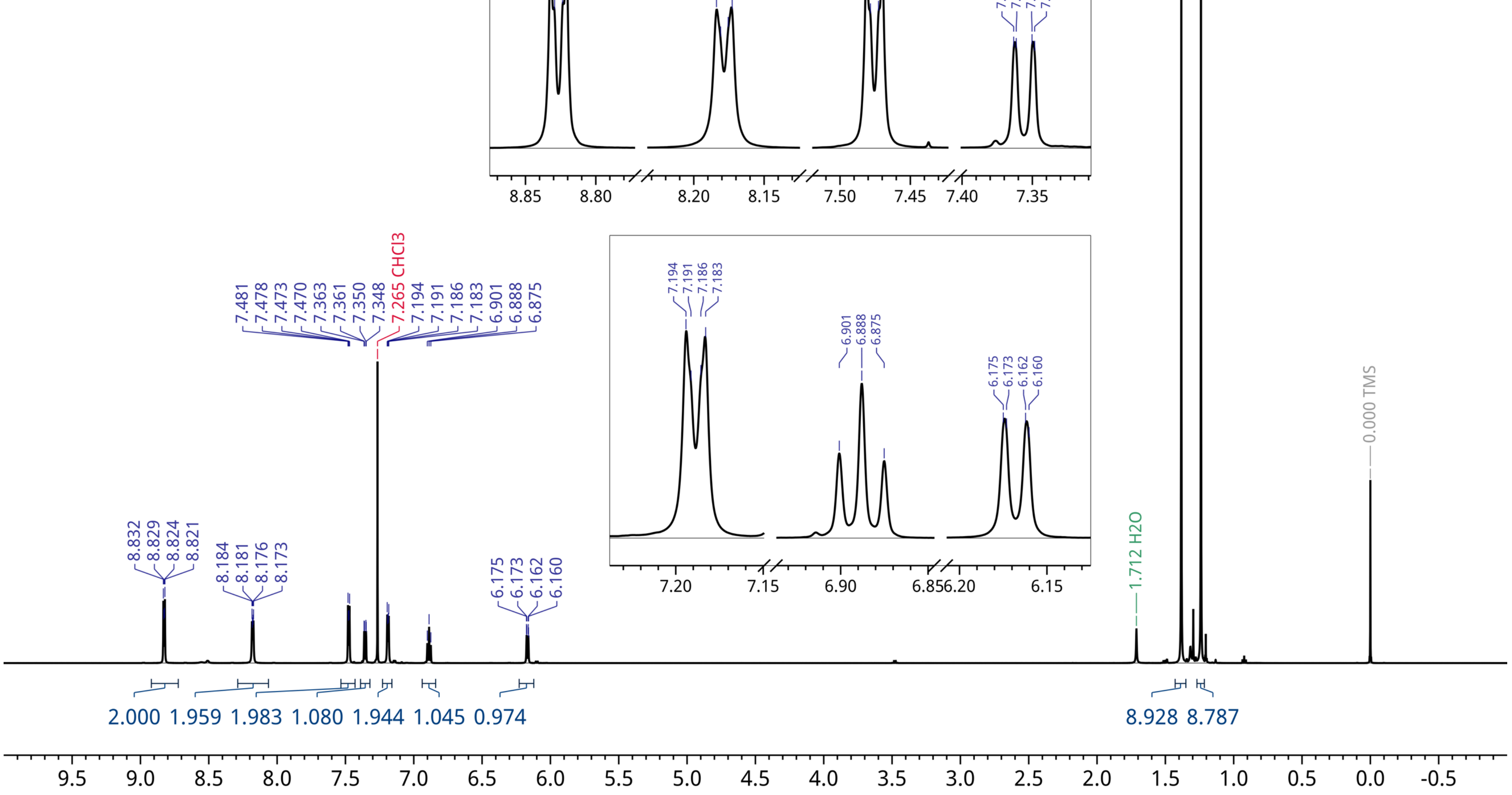

4-*tert*-butylpyridine supported 5-membered palladacycle (**1a-PdCycle-^{t}BuPy**)

^{13}C NMR (151 MHz, CDCl$_3$) δ 176.61, 163.69, 162.59, 152.49, 149.37, 147.45, 139.42, 134.88, 128.86 (q, J = 31.0 Hz), 128.22, 123.67, 123.34 (q, J = 273.8 Hz), 122.82 (q, J = 6.7 Hz), 122.08, 35.37, 34.98, 30.27, 30.22.

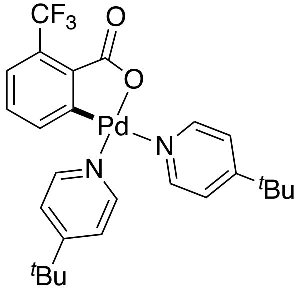

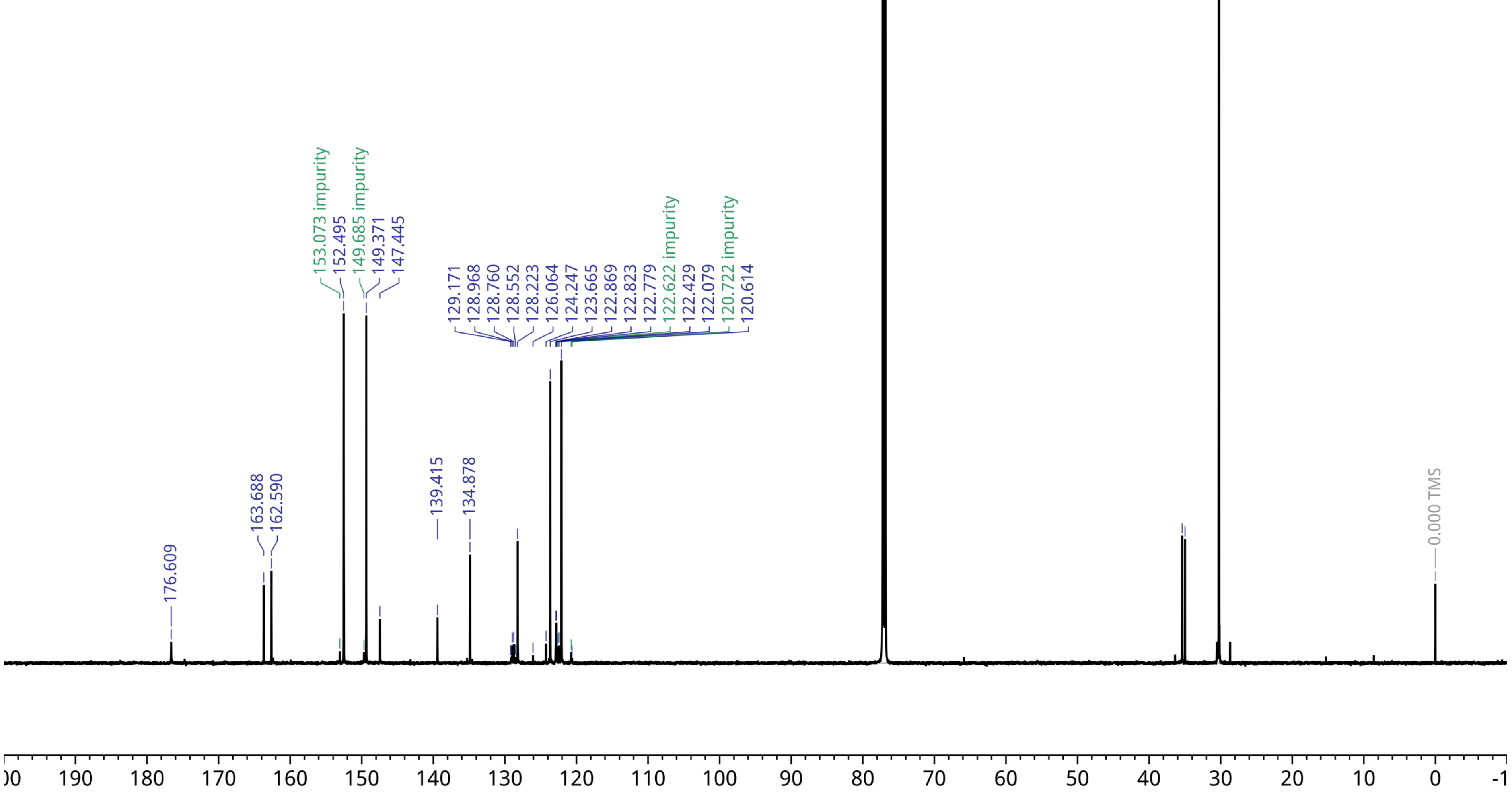

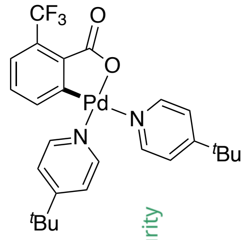

Insert shows the three quartets from C–F coupling at 128.86 ppm (J = 31.0 Hz), 123.34 ppm (J = 273.8 Hz), and 122.82 ppm (J = 6.7 Hz, three of four peaks are marked). Peak-picking on some peaks is limited due to S/N.

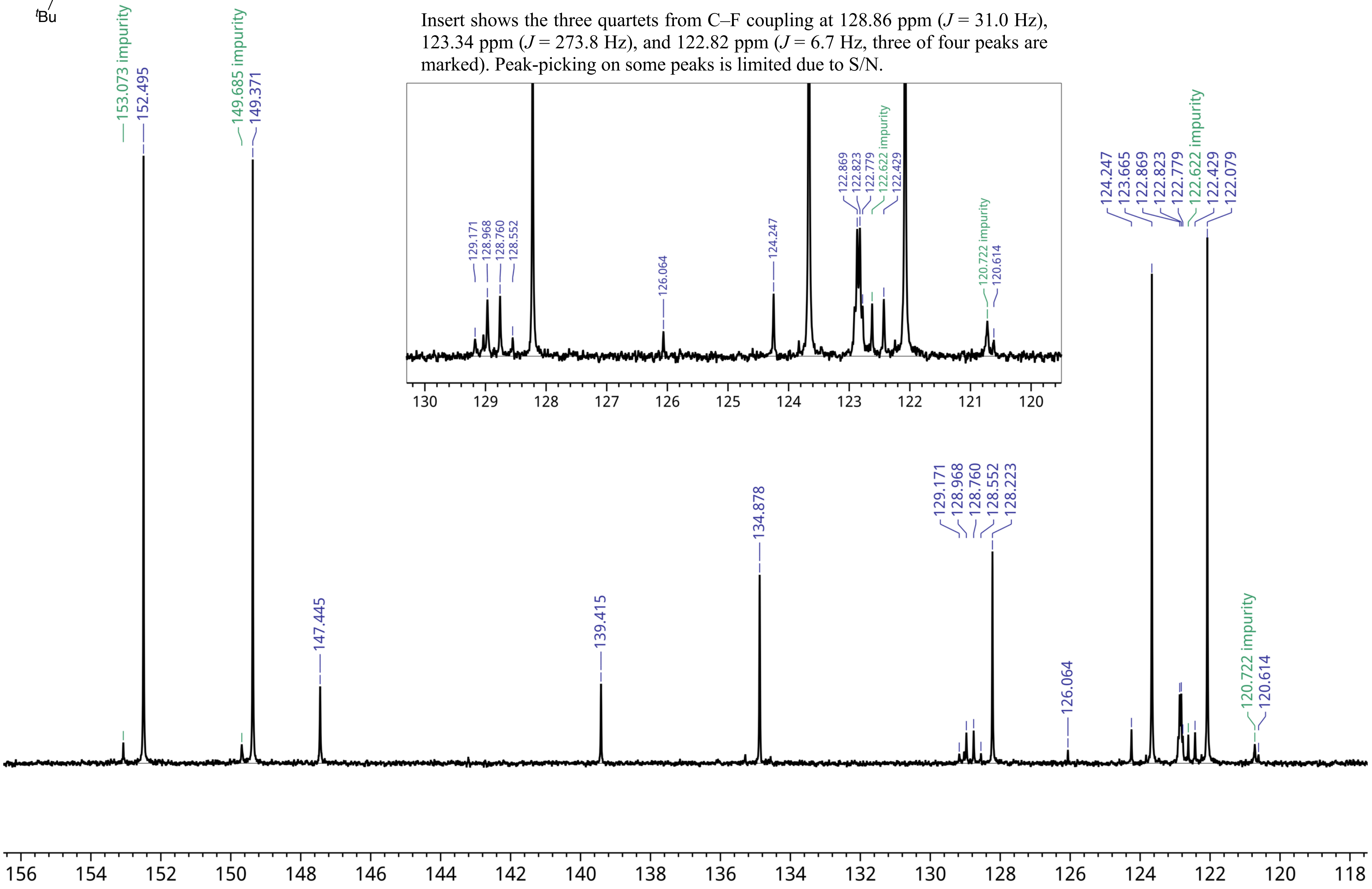

4-*tert*-butylpyridine supported 5-membered palladacycle (**1a-PdCycle-^{t}BuPy**)

^{19}F NMR (564 MHz, $CDCl_3$) δ –58.37.

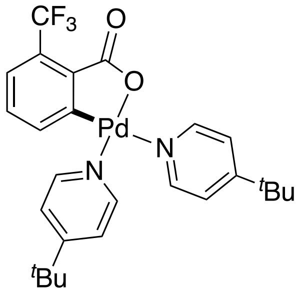

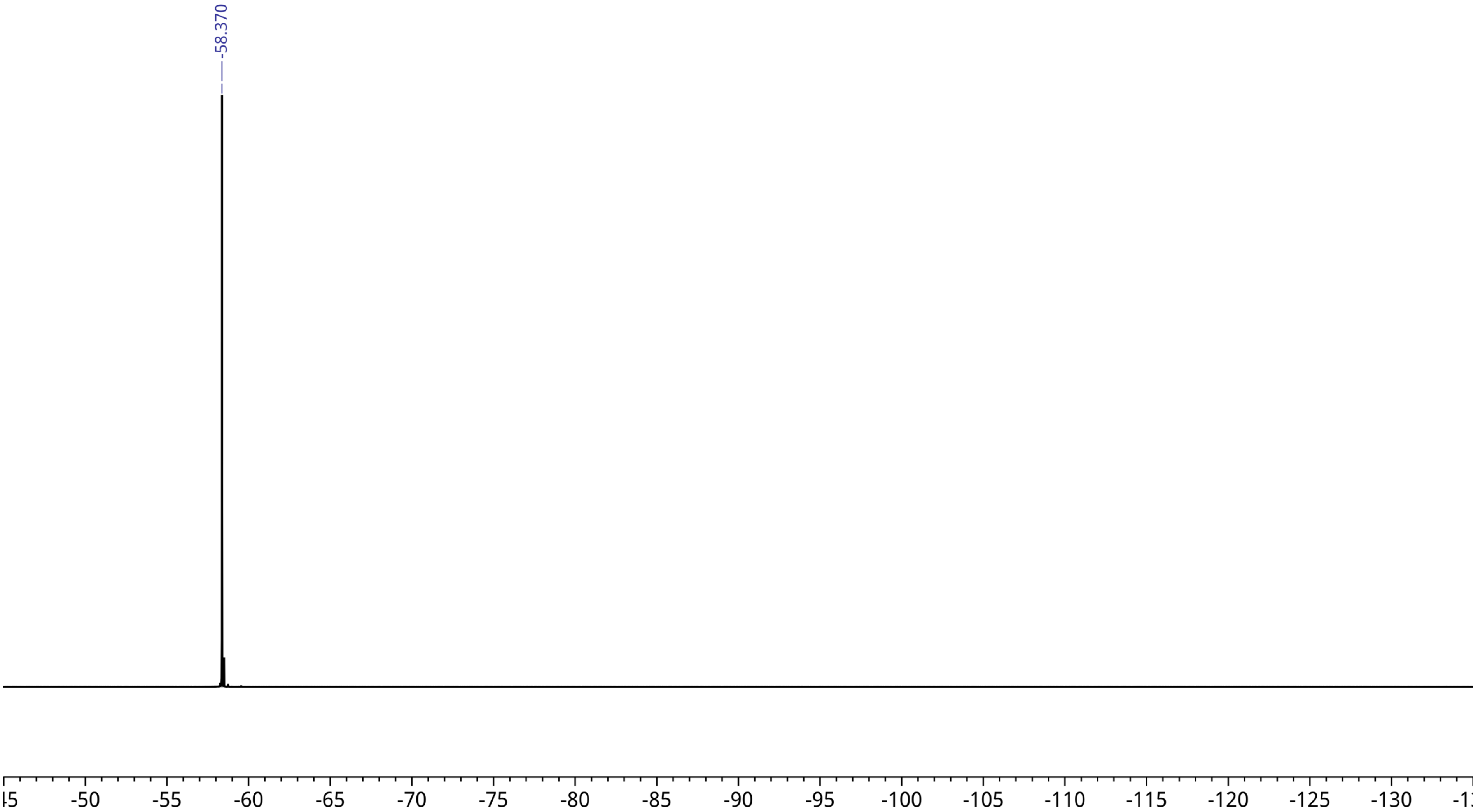

4-methylpyridine supported 6-membered palladacycle (**1b-PdCycle-MePy**)

^{1}H NMR (600 MHz, $CDCl_3$) δ 8.47 (d, J = 5.3 Hz, 2H), 8.14 (d, J = 5.4 Hz, 2H), 7.23 (d, J = 7.7 Hz, 1H), 7.19 (d, J = 5.8 Hz, 2H), 7.12 – 7.08 (m, 2H), 6.68 (t, J = 7.7 Hz, 1H), 6.50 (d, J = 7.6 Hz, 1H), 4.27 (s, 2H), 2.44 (s, 3H), 2.34 (s, 3H).

* Note: ^{t}AmylOH peaks not reported.

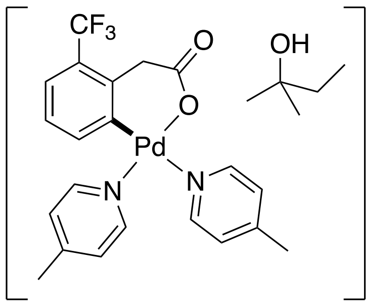

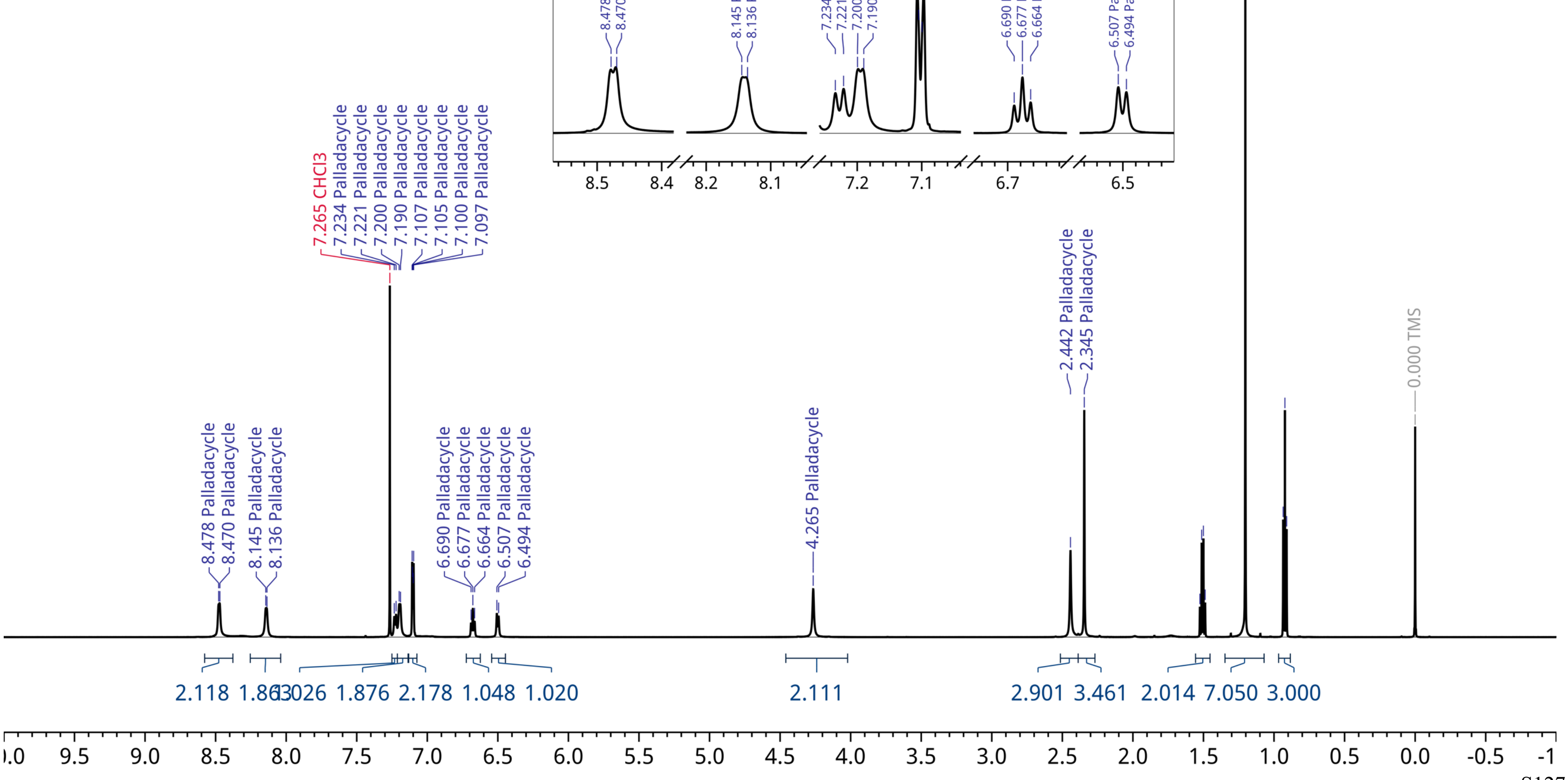

4-methylpyridine supported 6-membered palladacycle (**1b-PdCycle-MePy**)

^{1}H NMR (600 MHz, $CDCl_3$) δ 8.47 (d, J = 5.3 Hz, 1H), 8.14 (d, J = 5.4 Hz, 1H), 7.23 (d, J = 7.7 Hz, 1H), 7.19 (d, J = 5.8 Hz, 1H), 7.12 – 7.08 (m, 1H), 6.68 (t, J = 7.7 Hz, 1H), 6.50 (d, J = 7.6 Hz, 1H), 4.27 (s, 1H), 2.44 (s, 2H), 2.34 (s, 2H).

* Note: ^{t}AmylOH peaks not reported.

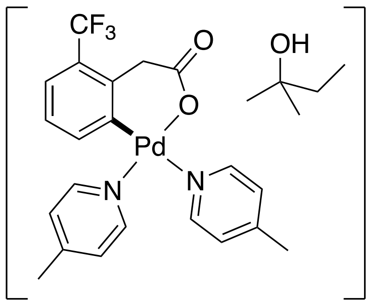

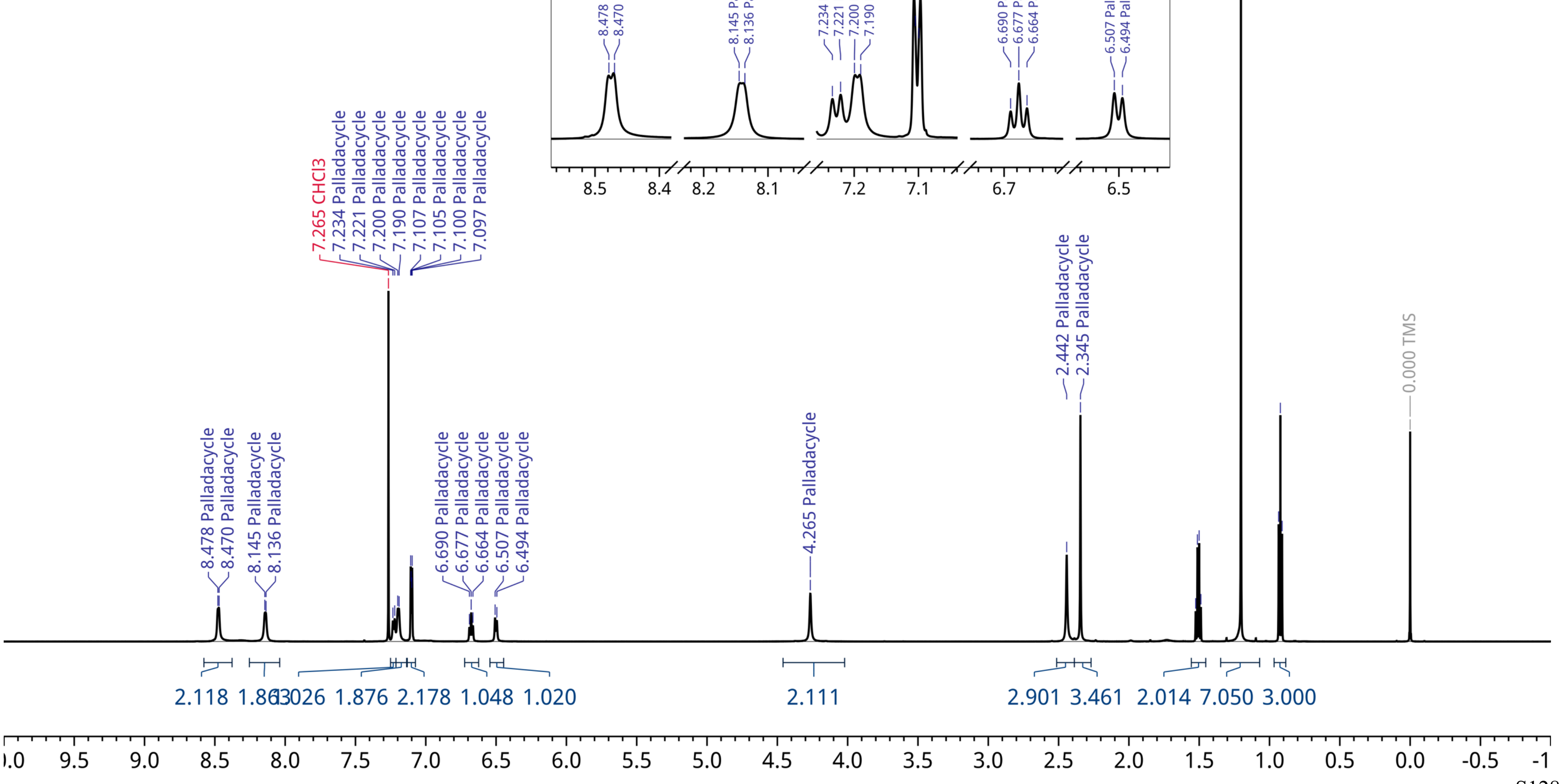

4-methylpyridine supported 6-membered palladacycle (**1b-PdCycle-MePy**)

^{1}H NMR (600 MHz, $CDCl_3$) δ 176.35, 152.60, 150.91, 150.03, 138.70, 127.02, 125.33, 123.55, 123.51, 121.78, 46.51, 21.31, 21.13.

* Note: *t*AmylOH peaks not reported.

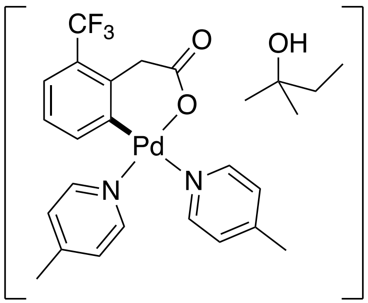

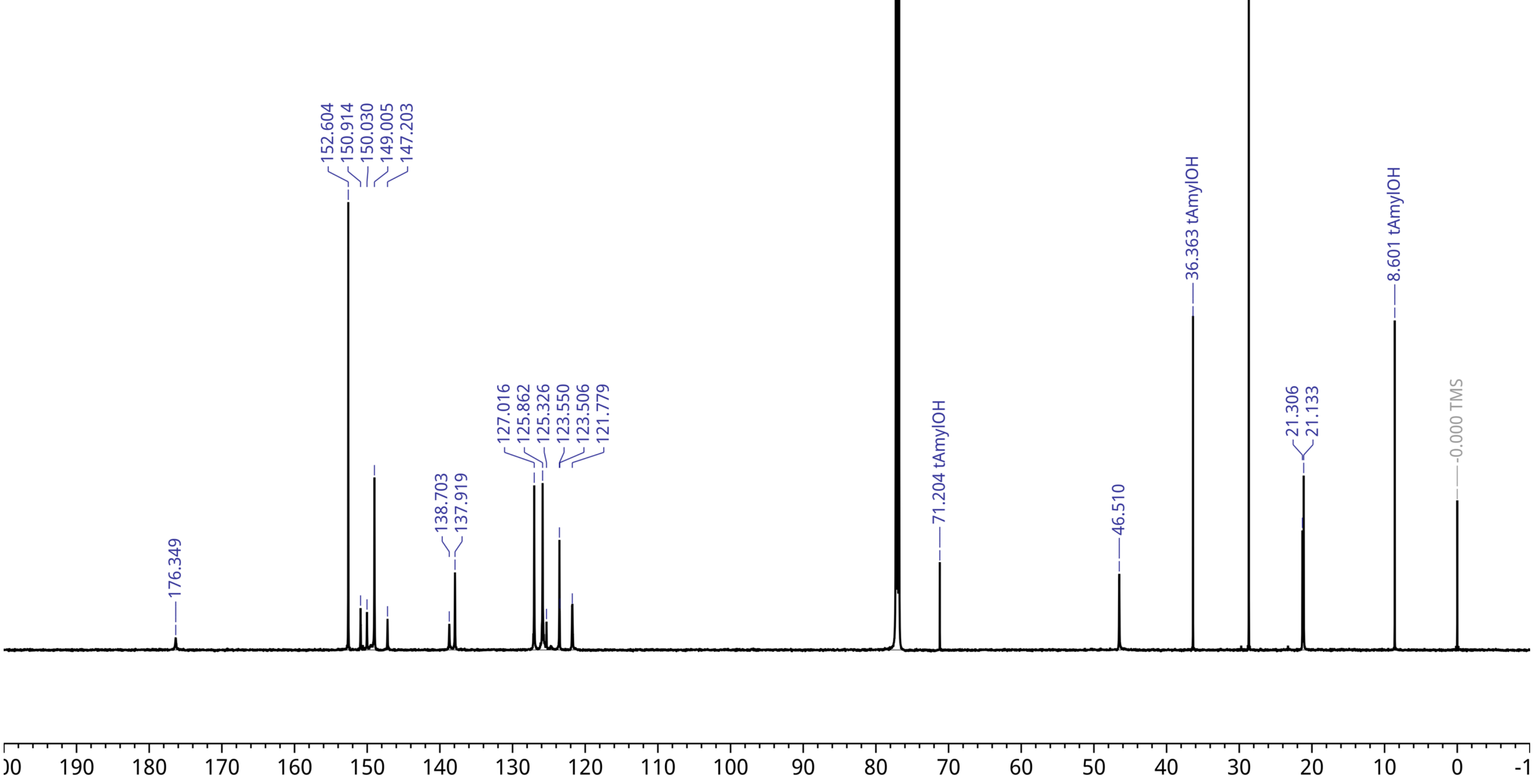

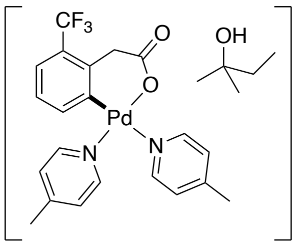
CF3
O
O
Pd
N
N
OH

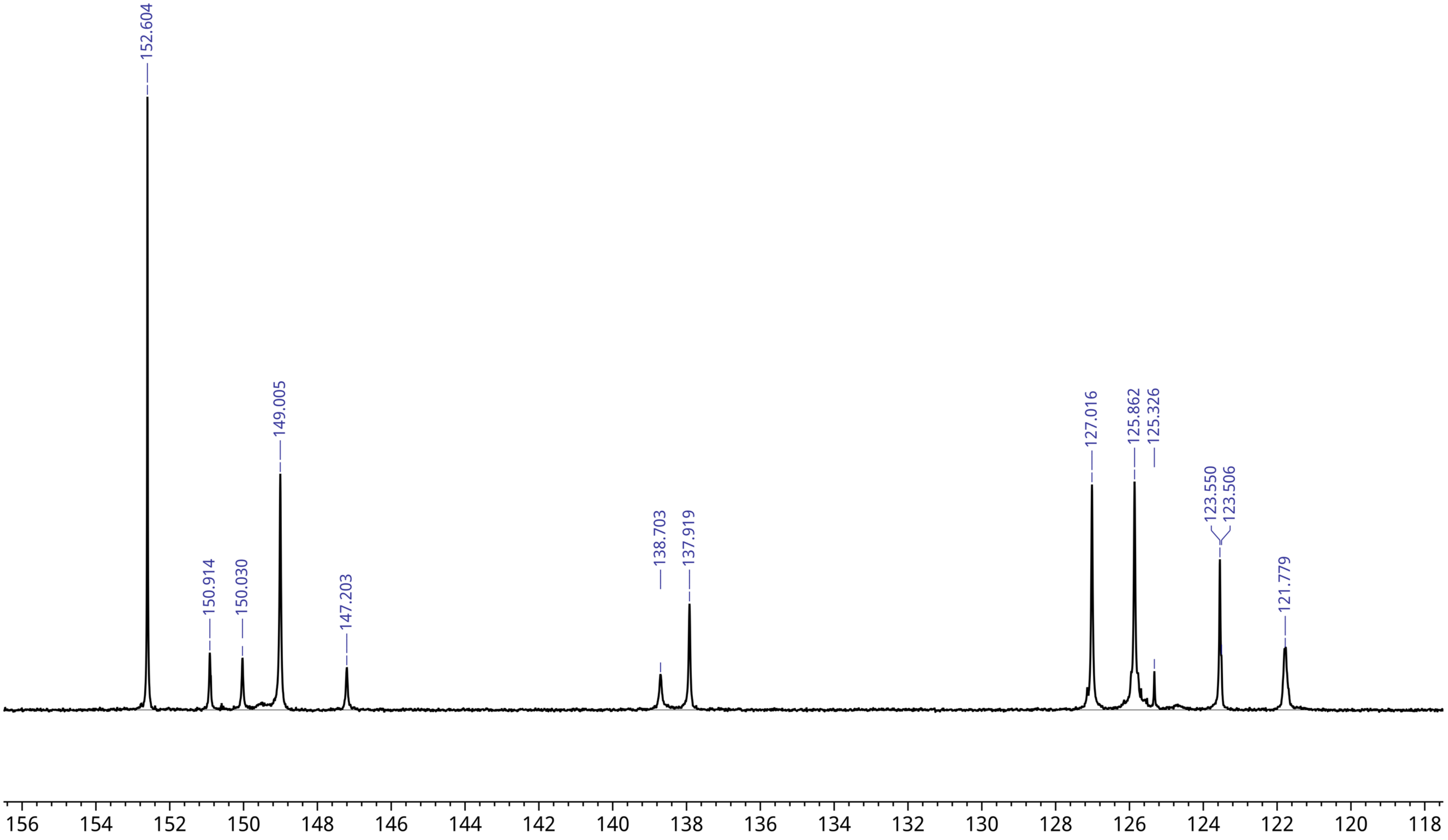
152.604
150.914
150.030
149.005
147.203
138.703
137.919
127.016
125.862
125.326
123.550
123.506
121.779
156
154
152
150
148
146
144
142
140
138
136
134
132
130
128
126
124
122
120
118

4-methylpyridine supported 6-membered palladacycle (**1b-PdCycle-MePy**)

^{19}F NMR (564 MHz, $CDCl_3$) δ –58.57.

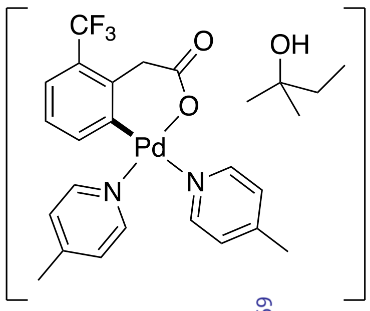

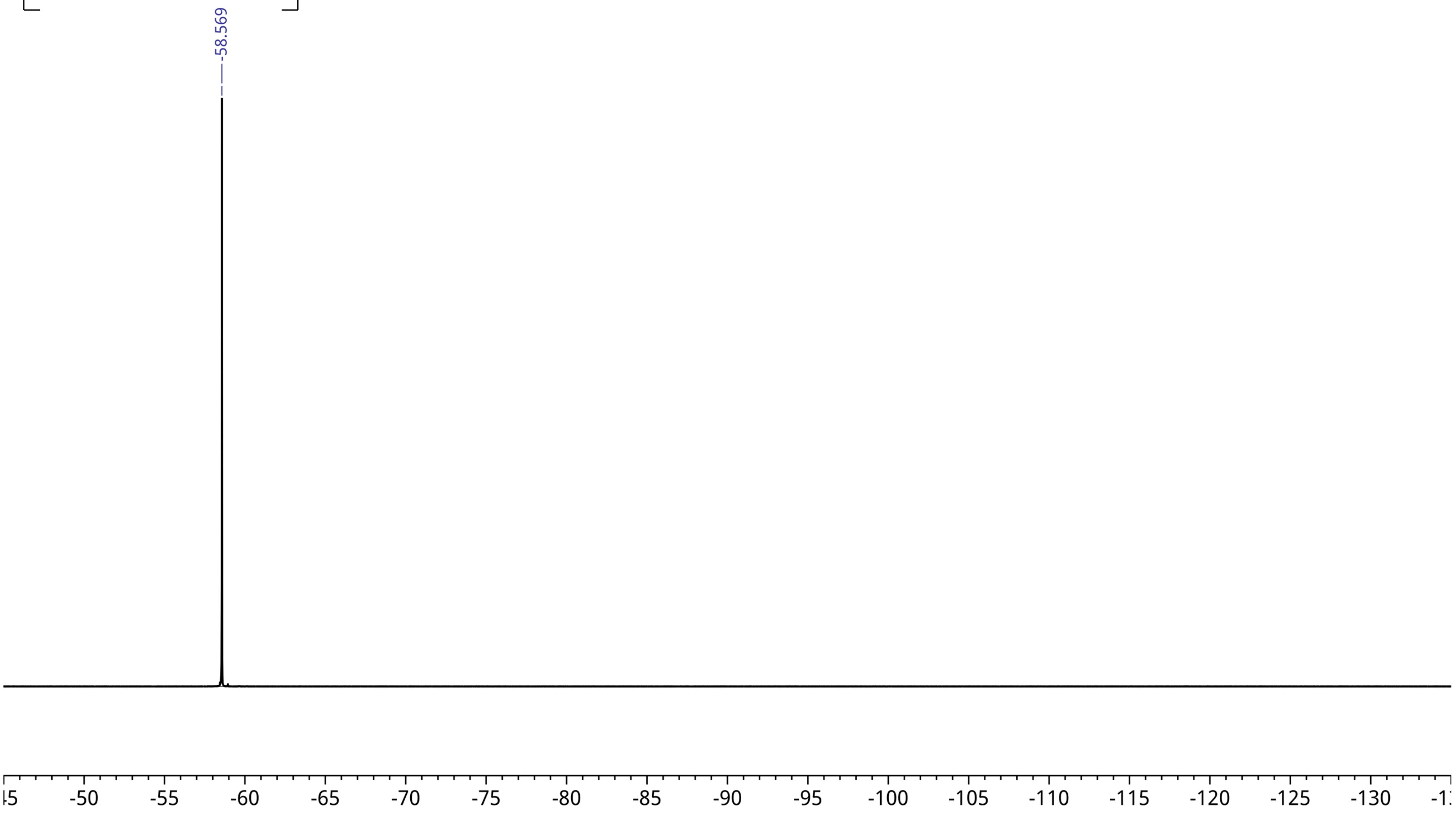

2-(4-fluorophenyl)benzoic acid (**P5-1**)

^{1}H NMR (600 MHz, $CDCl_3$) δ 11.63 (s, 1H), 7.96 (dd, *J* = 7.8, 1.4 Hz, 1H), 7.43 (td, *J* = 7.6, 1.3 Hz, 1H), 7.33 (dd, *J* = 7.7, 1.3 Hz, 1H), 7.30 – 7.26 (m, 2H), 7.10 – 7.04 (m, 2H).

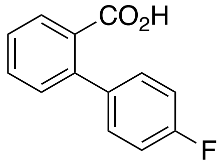

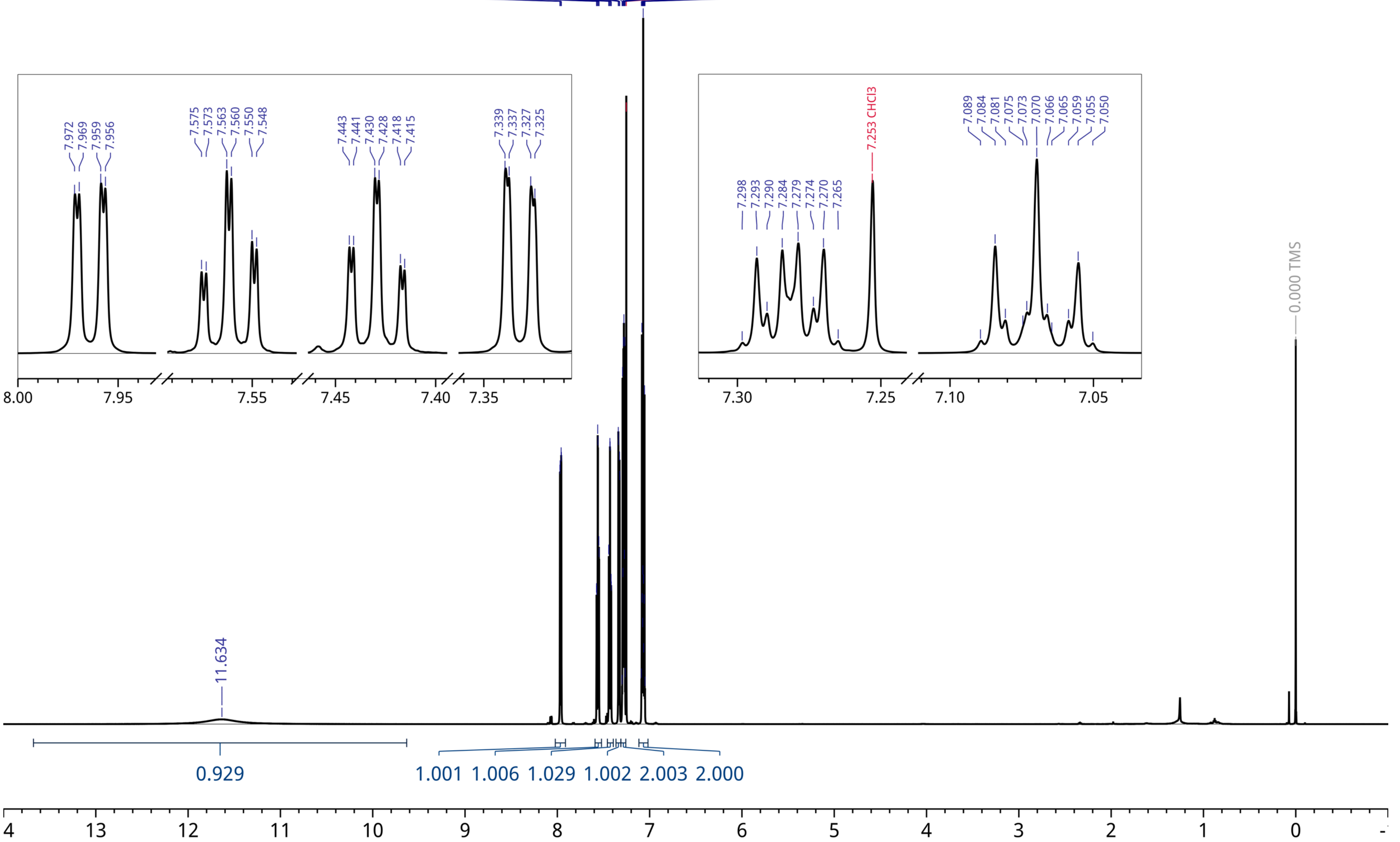

2-(4-fluorophenyl)benzoic acid (**P5-1**)

$^{13}C\{^{1}H\}$ NMR (151 MHz, $CDCl_3$) δ 173.26, 162.37 (d, J = 246.3 Hz), 142.51, 137.02 (d, J = 3.4 Hz), 132.27, 131.26, 130.87, 130.08 (d, J = 8.1 Hz), 129.09, 127.39, 115.00 (d, J = 21.5 Hz).

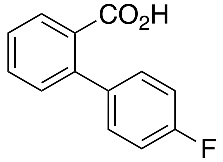

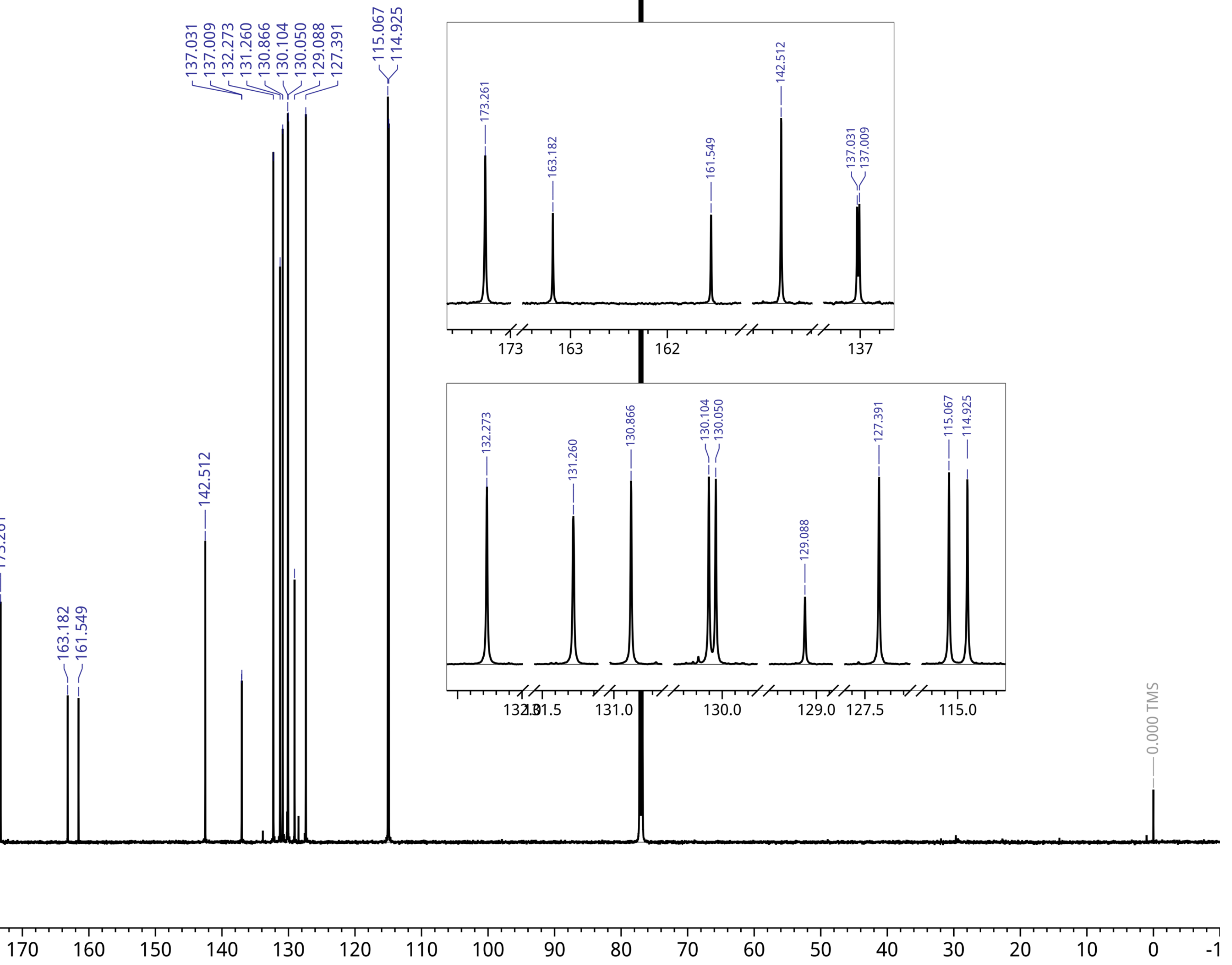

2-(4-fluorophenyl)benzoic acid (**P5-1**)

^{19}F NMR (564 MHz, $CDCl_3$) δ –115.34 (tt, J = 8.7, 5.3 Hz).

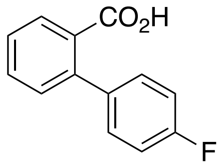

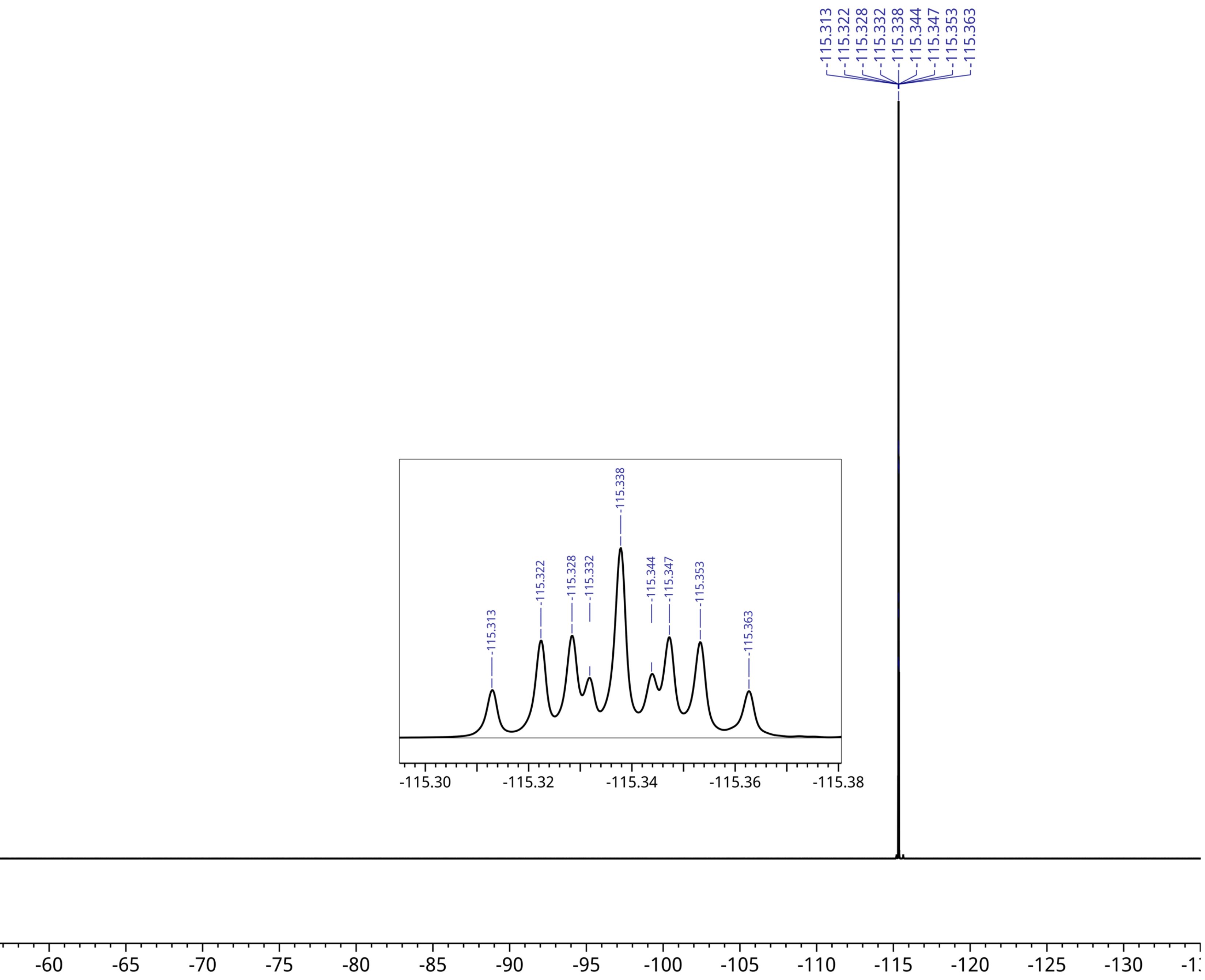

2-(4-fluorophenyl)-6-methylbenzoic acid (**P5-2**)

^{1}H NMR (600 MHz, $CDCl_3$) δ 11.06 (s, 1H), 7.40 – 7.33 (m, 3H), 7.25 – 7.23 (m, 1H), 7.18 (dd, J = 7.8, 1.2 Hz, 1H), 7.11 – 7.04 (m, 2H), 2.45 (s, 3H).

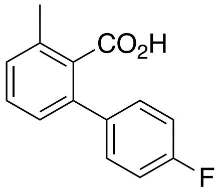

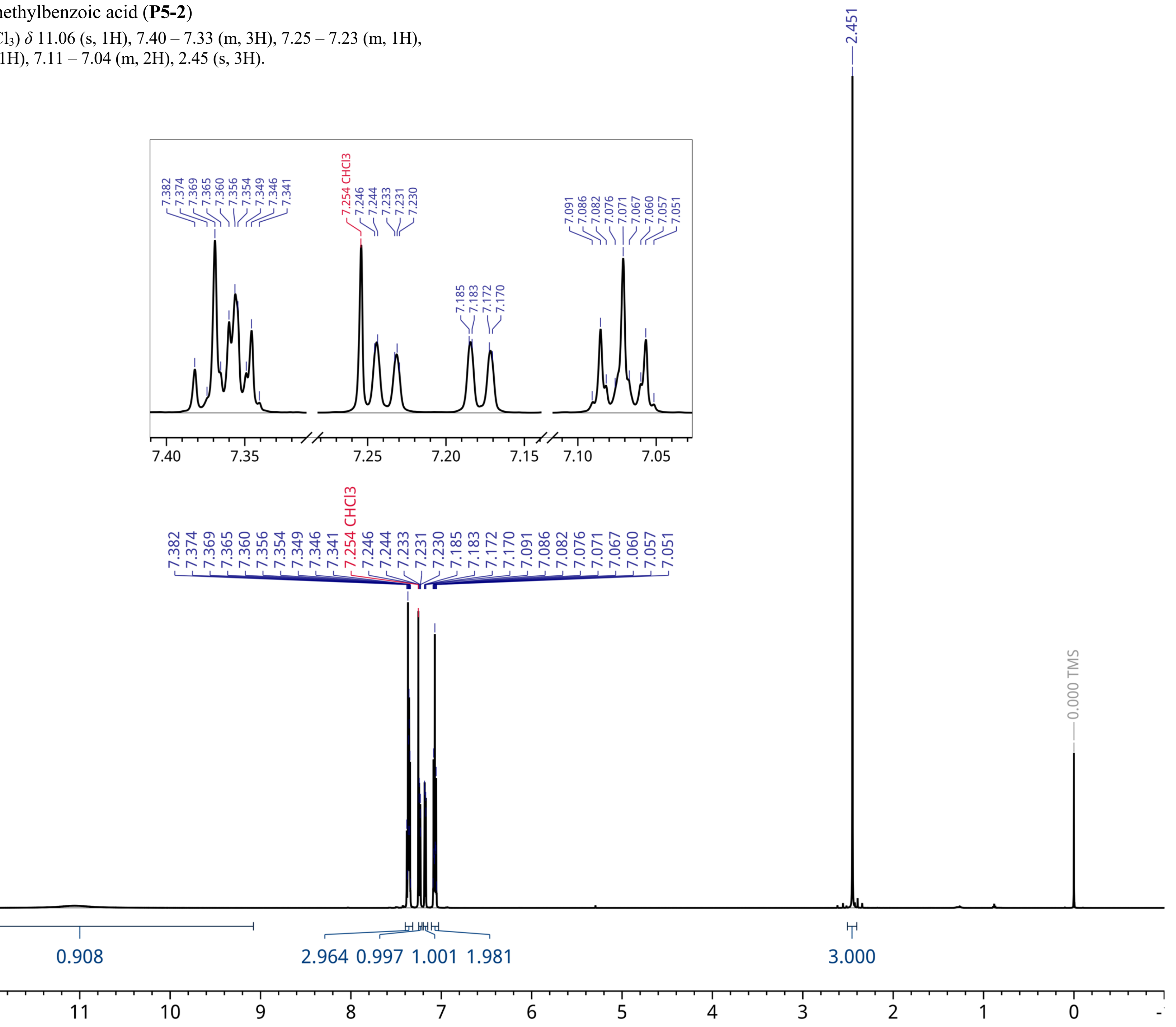

2-(4-fluorophenyl)-6-methylbenzoic acid (**P5-2**)

$^{13}C\{^{1}H\}$ NMR (151 MHz, $CDCl_3$) δ 174.76, 162.47 (d, J = 246.8 Hz), 139.14, 136.60 (d, J = 3.3 Hz), 135.50, 132.07, 130.03 (d, J = 8.1 Hz), 129.82, 129.37, 127.43, 115.30 (d, J = 21.4 Hz), 19.88.

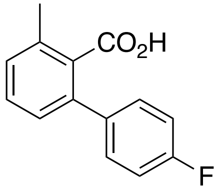

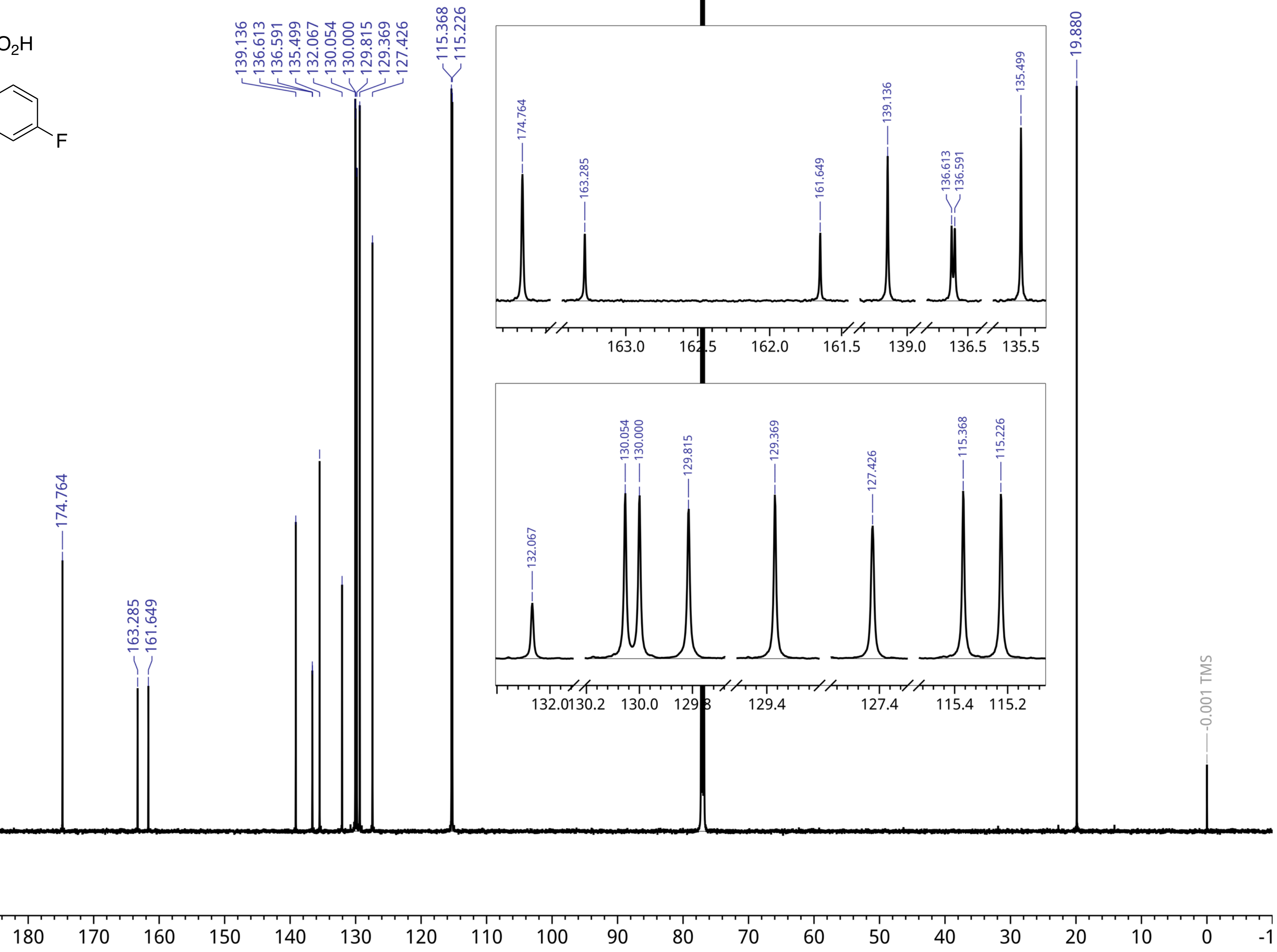

2-(4-fluorophenyl)-6-methylbenzoic acid (**P5-2**)

^{19}F NMR (564 MHz, $CDCl_3$) δ –114.91 (tt, J = 8.7, 5.3 Hz).

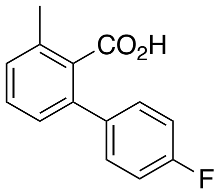

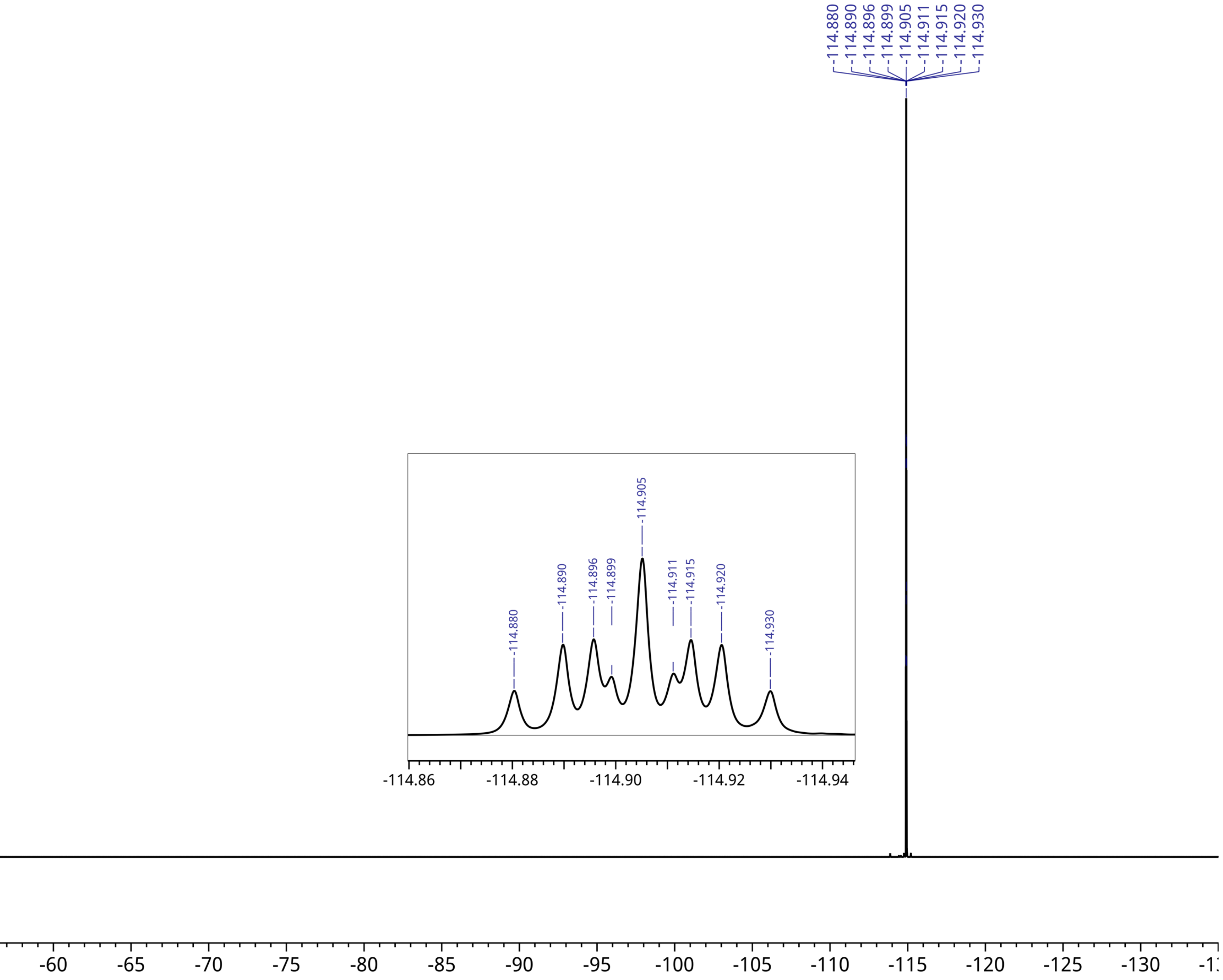

2-(4-fluorophenyl)-4-methylbenzoic acid (**P5-3**)

^{1}H NMR (600 MHz, $CDCl_3$) δ 10.52 (s, 1H), 7.73 (dd, *J* = 7.9, 1.2 Hz, 1H), 7.61 (td, *J* = 7.8, 1.0 Hz, 1H), 7.55 (dd, *J* = 7.8, 1.2 Hz, 1H), 7.39 – 7.33 (m, 2H), 7.14 – 7.07 (m, 2H).

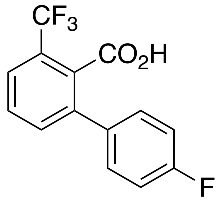

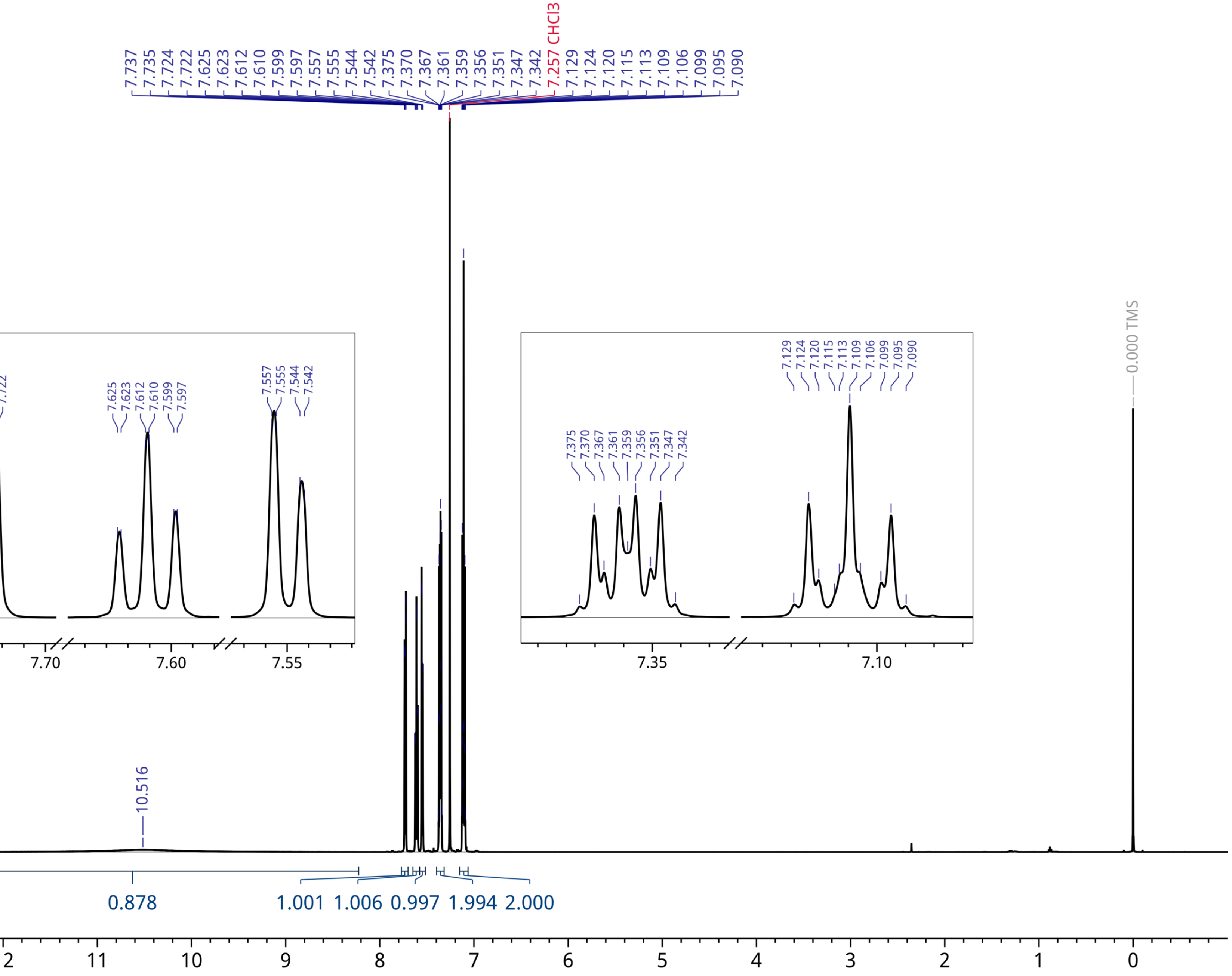

2-(4-fluorophenyl)-4-methylbenzoic acid (**P5-3**)

$^{13}C\{^{1}H\}$ NMR (151 MHz, $CDCl_3$) δ 172.21, 162.88 (d, J = 248.1 Hz), 140.20, 134.66 (d, J = 3.4 Hz), 133.66, 130.31 (d, J = 8.3 Hz), 130.23 (q, J = 2.2 Hz), 129.99, 127.88 (q, J = 32.3 Hz), 125.24 (q, J = 4.8 Hz), 123.30 (q, J = 273.9 Hz), 115.61 (d, J = 21.7 Hz).

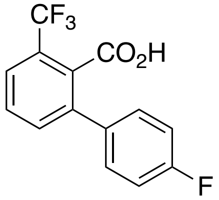

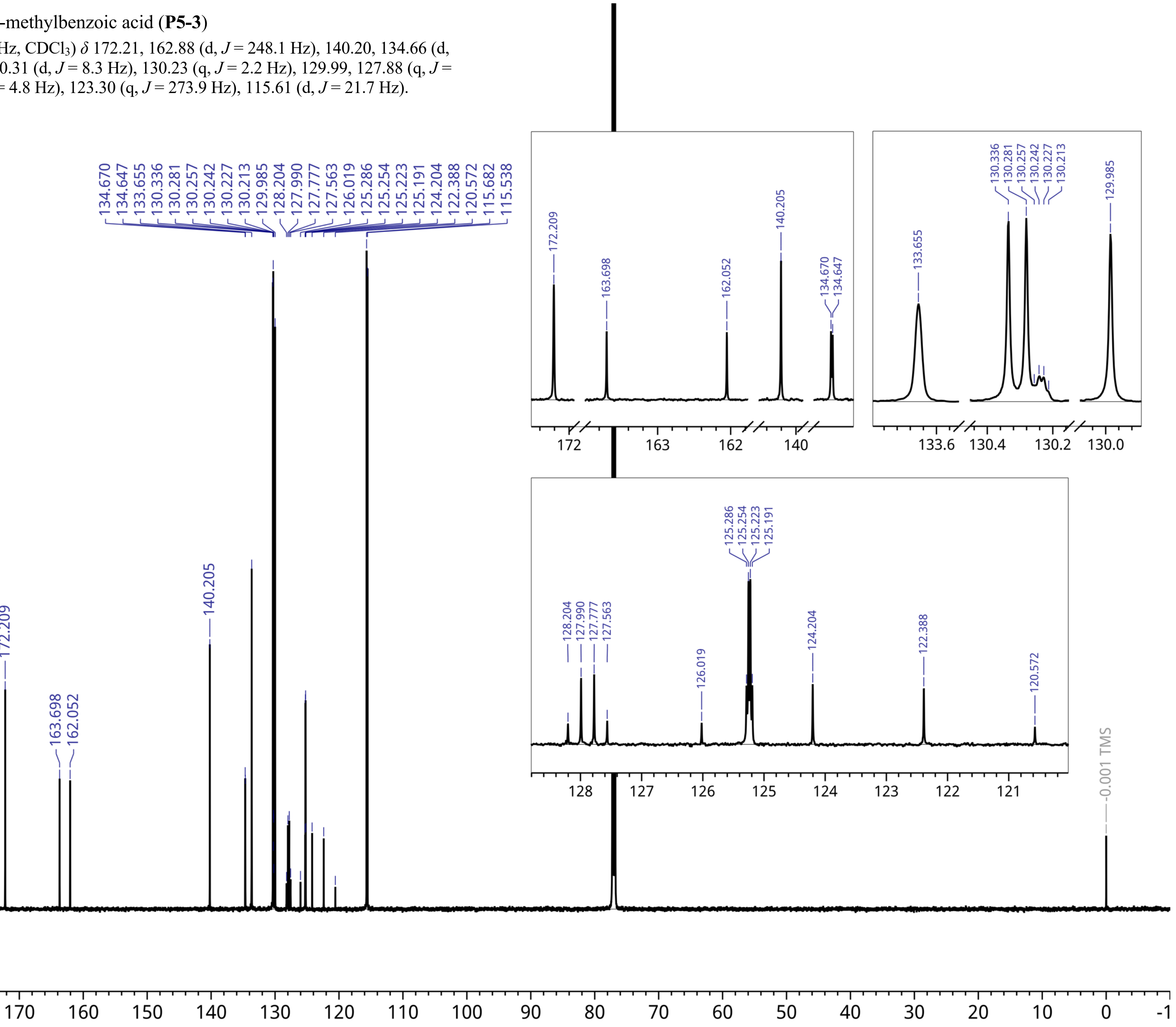

2-(4-fluorophenyl)-4-methylbenzoic acid (**P5-3**)

^{19}F NMR (564 MHz, $CDCl_3$) δ –59.40, –113.48 (tt, J = 8.6, 5.2 Hz).

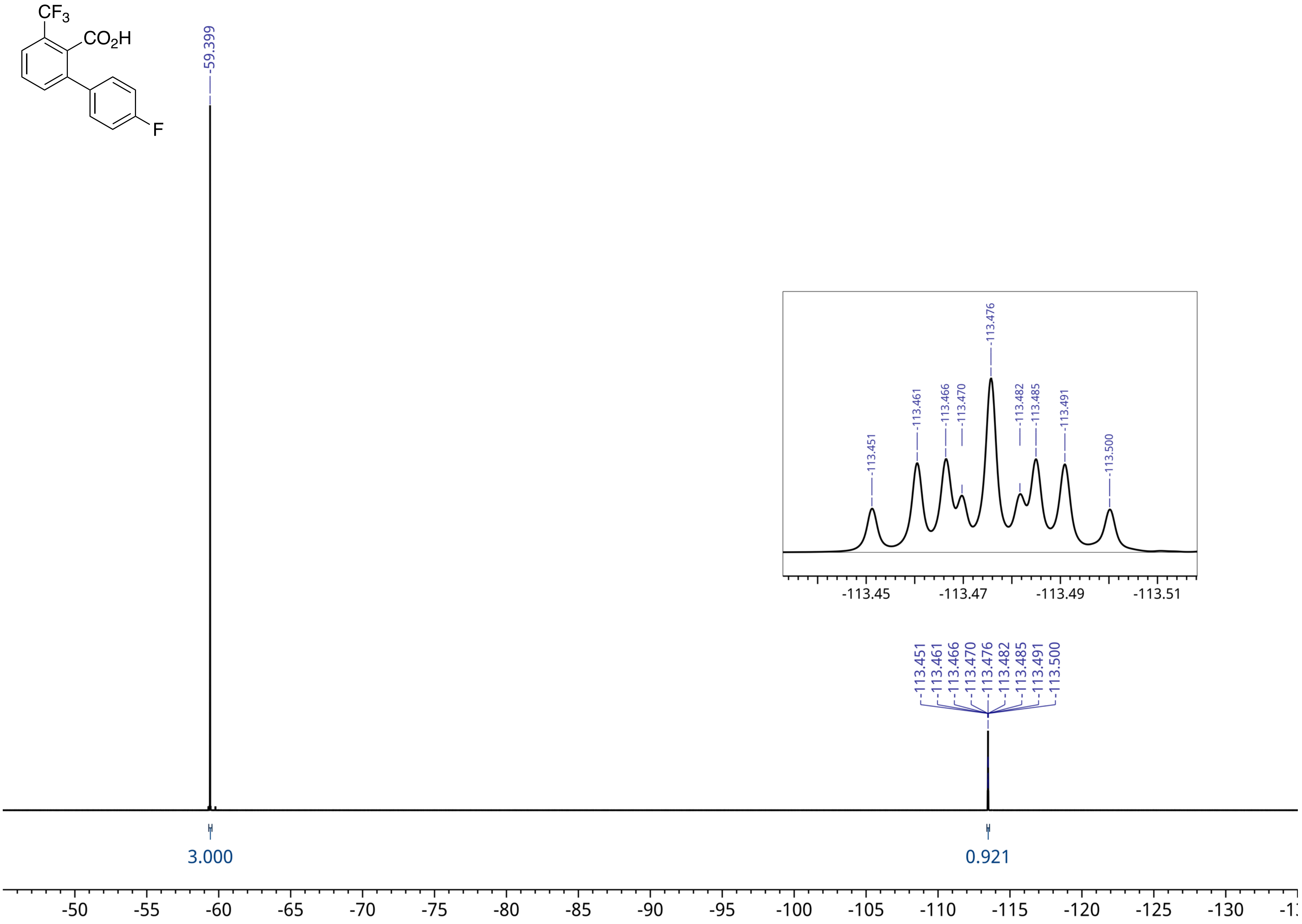

2-(4-fluorophenyl)-6-(trifluoromethyl)benzoic acid (**P5-4**)

^{1}H NMR (600 MHz, $CDCl_3$) δ 11.77 (s, 1H), 7.89 (d, J = 8.0 Hz, 1H), 7.28 – 7.24 (m, 2H), 7.24 – 7.21 (m, 1H), 7.14 – 7.10 (m, 1H), 7.09 – 7.02 (m, 2H), 2.42 (s, 3H).

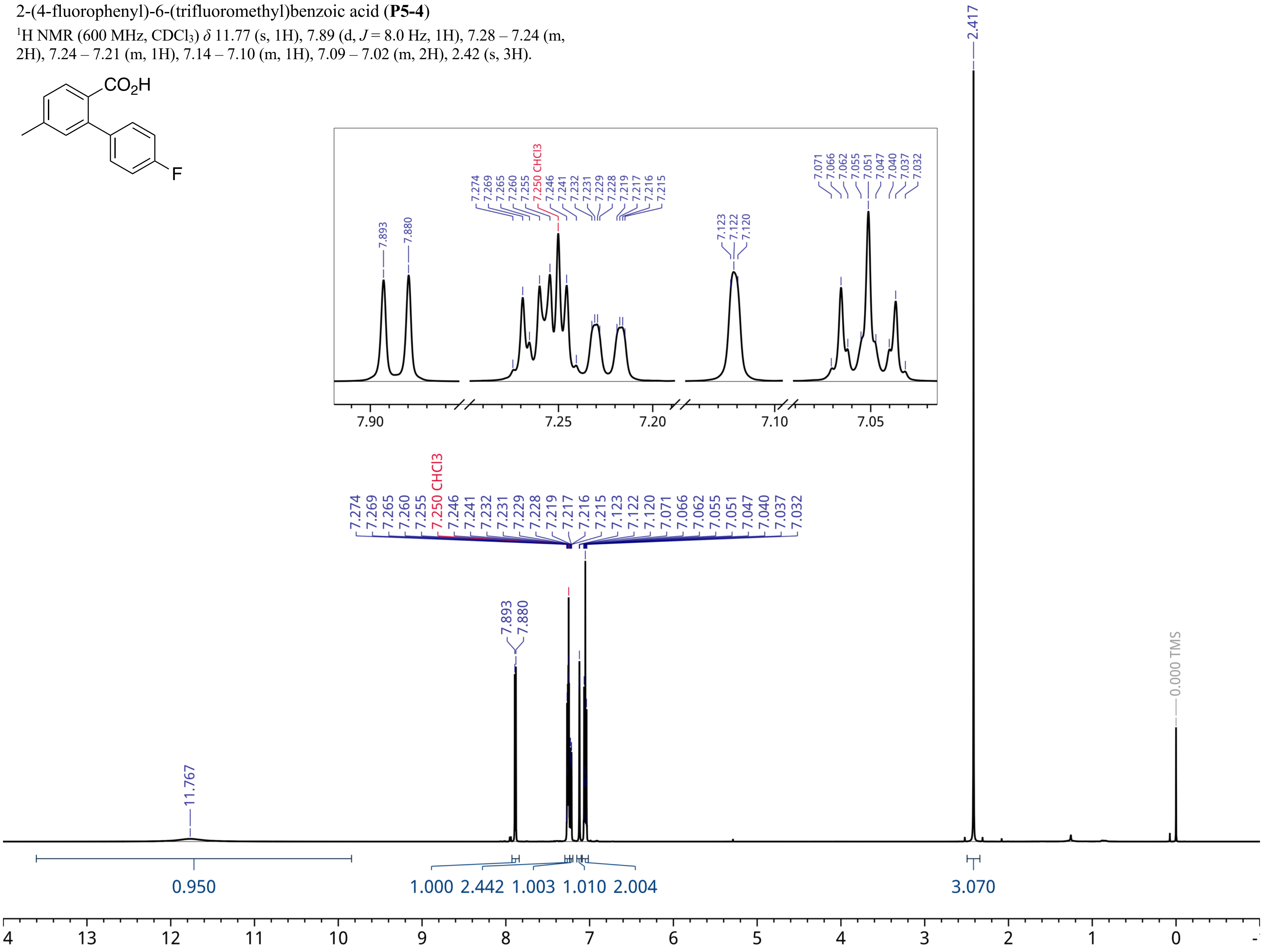

2-(4-fluorophenyl)-6-(trifluoromethyl)benzoic acid (**P5-4**)

$^{13}C\{^{1}H\}$ NMR (151 MHz, $CDCl_3$) δ 173.16, 162.27 (d, J = 246.1 Hz), 143.05, 142.80, 137.28 (d, J = 3.3 Hz), 132.15, 131.23, 130.05 (d, J = 8.1 Hz), 128.10, 126.07, 114.85 (d, J = 21.4 Hz), 21.50.

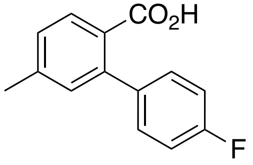

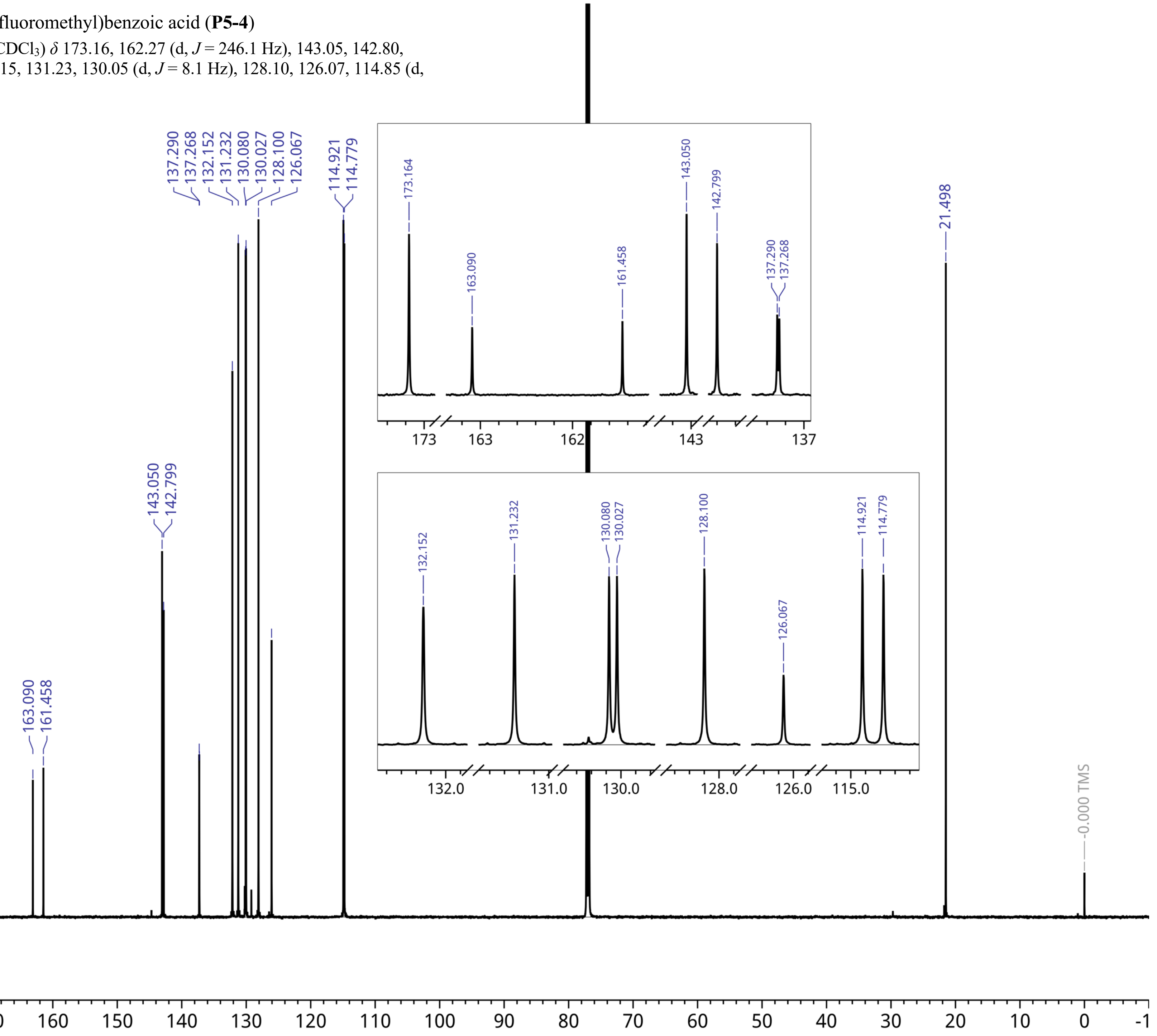

2-(4-fluorophenyl)-6-(trifluoromethyl)benzoic acid (**P5-4**)

^{19}F NMR (564 MHz, $CDCl_3$) δ –115.60 (tt, J = 8.7, 5.3 Hz).

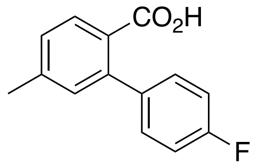

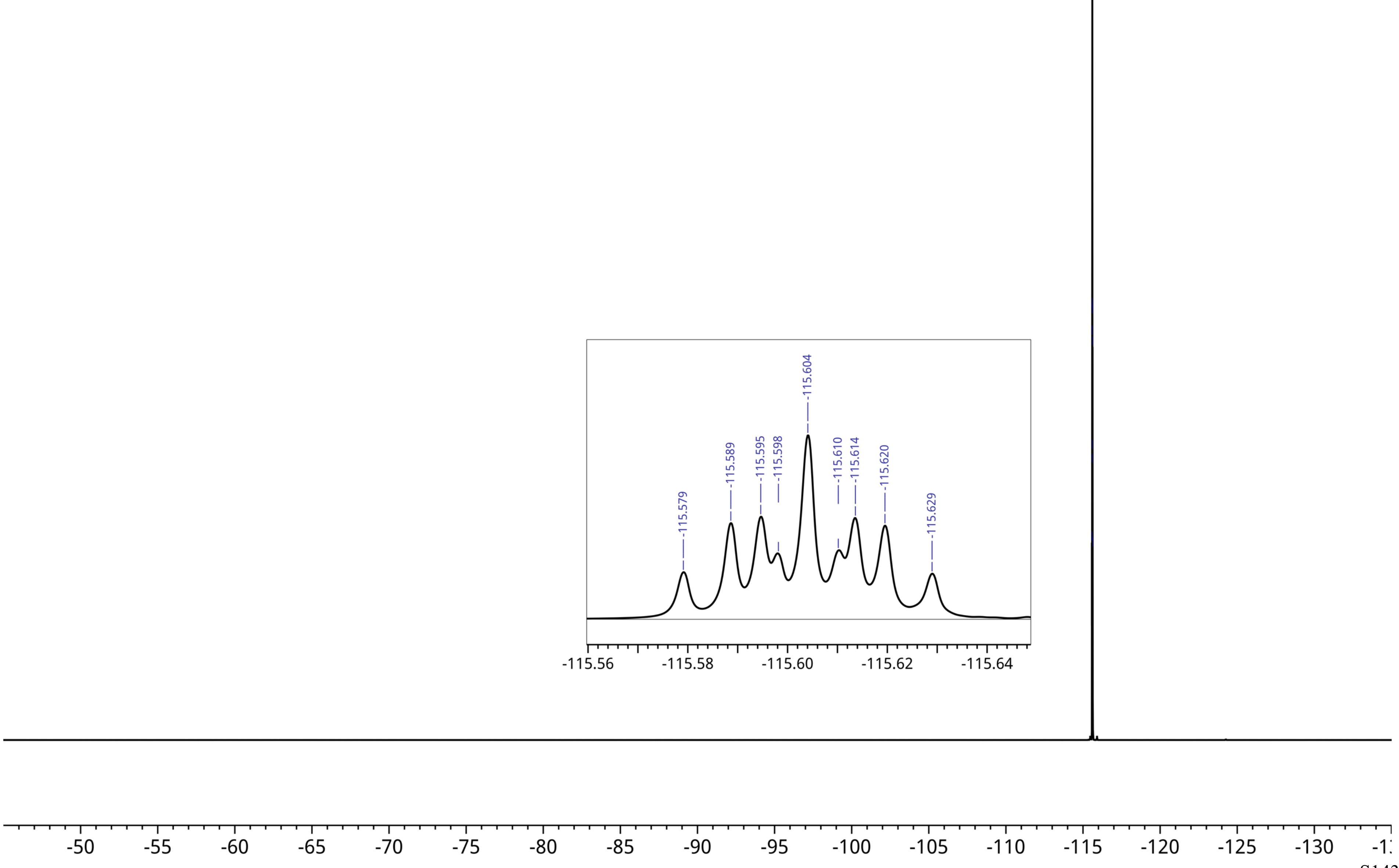

2-(4-fluorophenyl)-4-(trifluoromethyl)benzoic acid (**P5-5**)

^{1}H NMR (600 MHz, $CDCl_3$) δ 11.39 (s, 1H), 8.06 (d, J = 8.2 Hz, 1H), 7.70 (dd, J = 8.3, 1.8 Hz, 1H), 7.61 (d, J = 1.9 Hz, 1H), 7.33 – 7.27 (m, 2H), 7.14 – 7.08 (m, 2H).

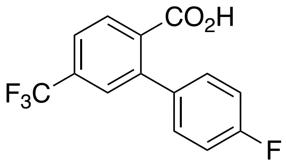

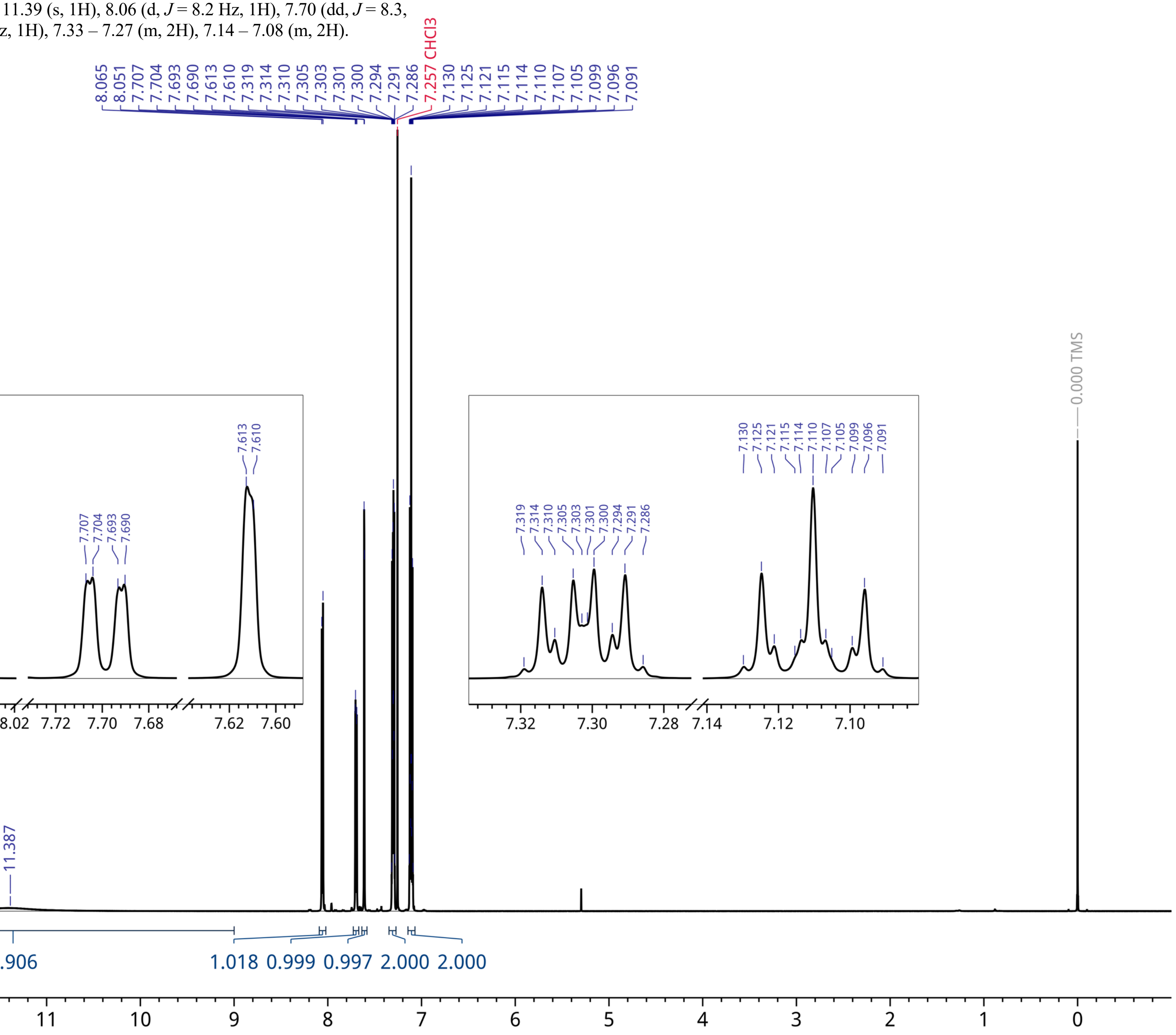

2-(4-fluorophenyl)-4-(trifluoromethyl)benzoic acid (**P5-5**)

$^{13}C\{^1H\}$ NMR (151 MHz, $CDCl_3$) δ 172.14, 162.76 (d, J = 247.8 Hz), 143.10, 135.51 (d, J = 3.5 Hz), 133.94 (q, J = 32.9 Hz), 132.33, 131.27, 130.11 (d, J = 8.1 Hz), 128.06 (q, J = 3.7 Hz), 124.23 (q, J = 3.6 Hz), 123.05 (q, J = 273.1 Hz), 115.39 (d, J = 21.7 Hz).

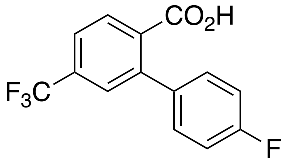

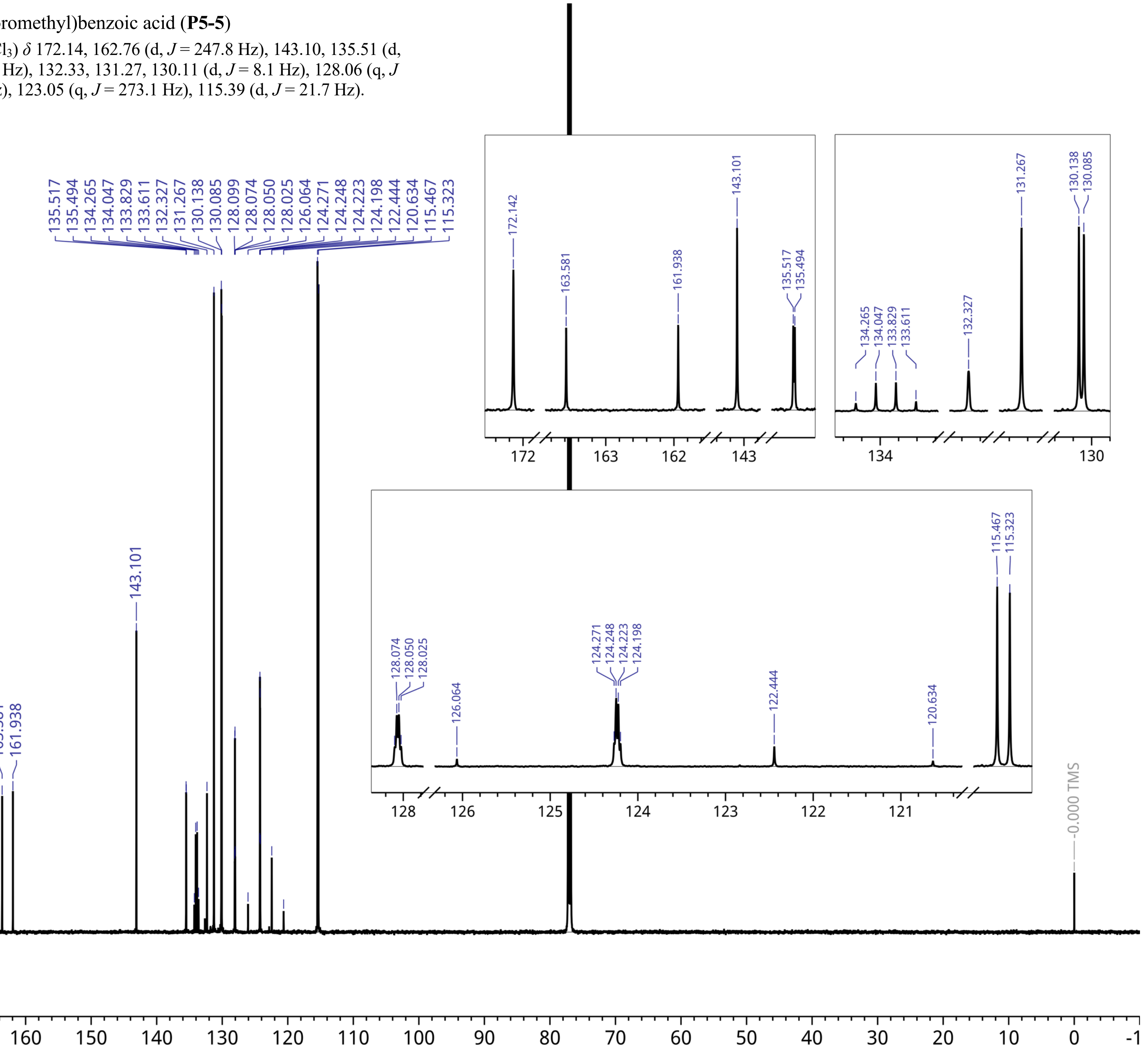

2-(4-fluorophenyl)-4-(trifluoromethyl)benzoic acid (**P5-5**)

^{19}F NMR (564 MHz, $CDCl_3$) δ –63.15, –113.89 (tt, J = 8.6, 5.2 Hz).

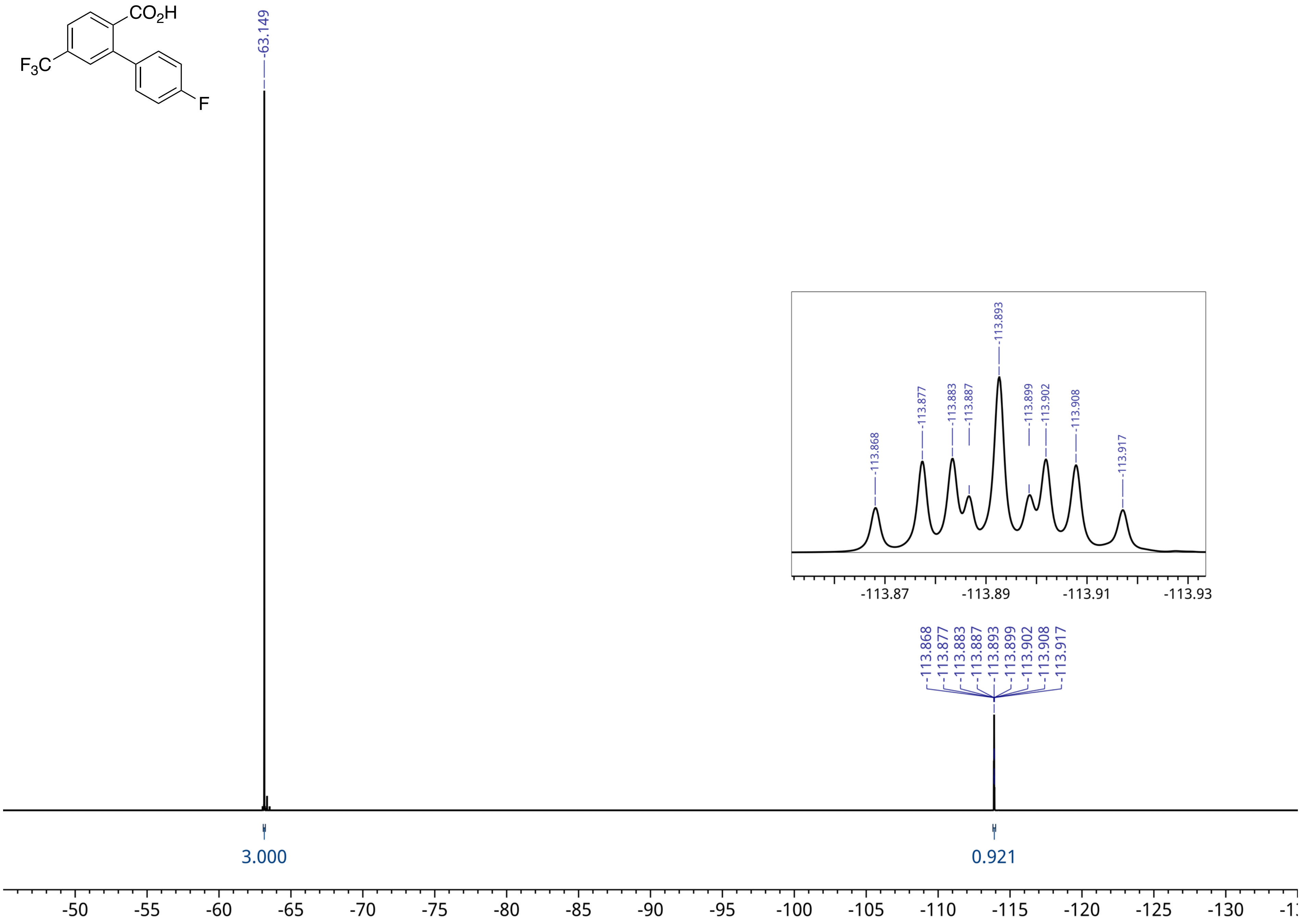

[2,6-di-(4-fluorophenyl)phenyl]acetic acid (**P6-1-2**)

^{1}H NMR (600 MHz, $CDCl_3$) δ 10.67 (s, 1H), 7.40 – 7.35 (m, 1H), 7.31 – 7.22 (m, 6H), 7.12 – 7.06 (m, 4H), 3.50 (s, 2H).

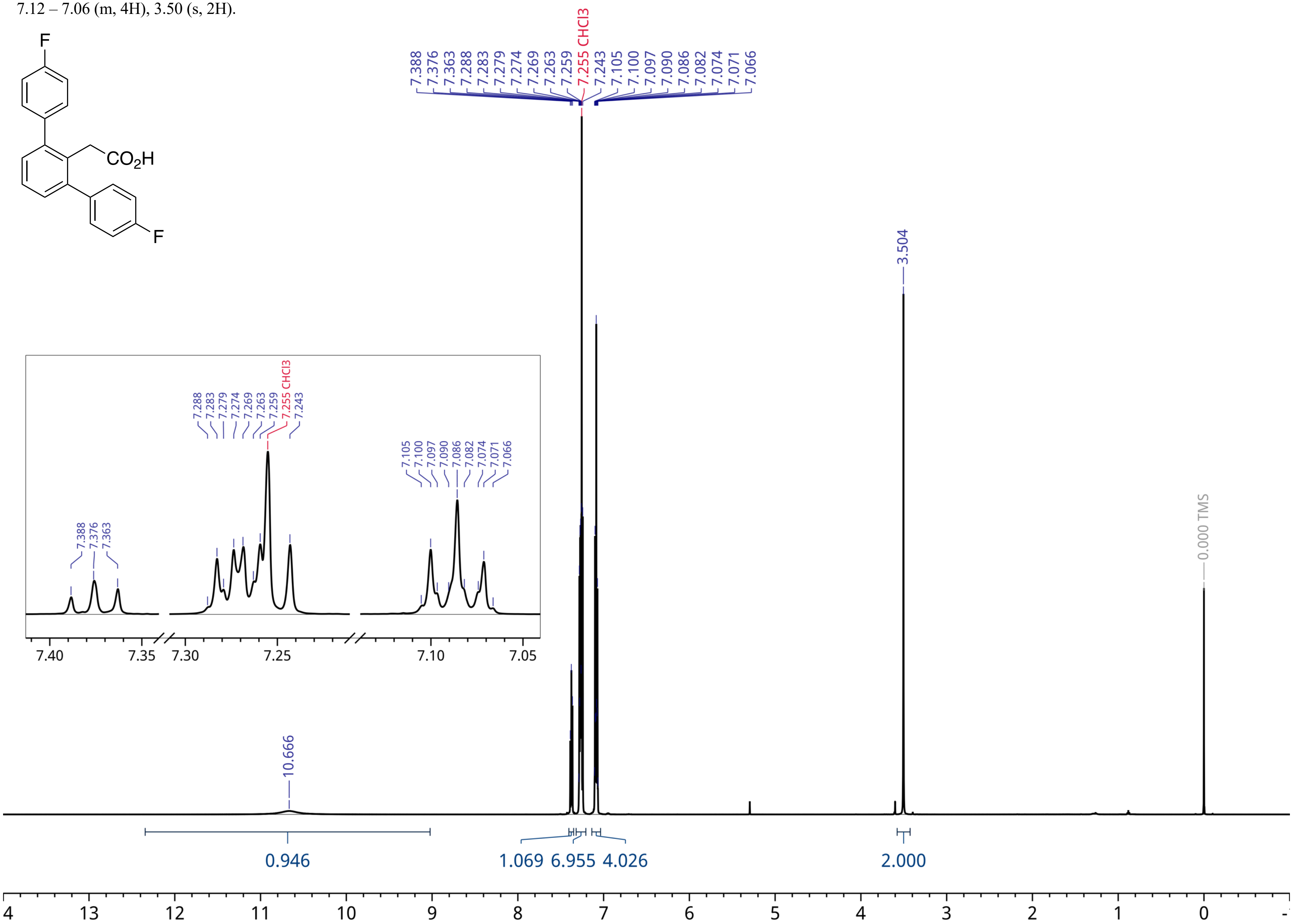

[2,6-di-(4-fluorophenyl)phenyl]acetic acid (**P6-1-2**)

$^{13}C\{^{1}H\}$ NMR (151 MHz, $CDCl_3$) δ 177.53, 162.23 (d, J = 246.6 Hz), 142.55, 137.21 (d, J = 3.4 Hz), 130.69 (d, J = 8.0 Hz), 129.64, 129.49, 115.25 (d, J = 21.3 Hz), 36.43.

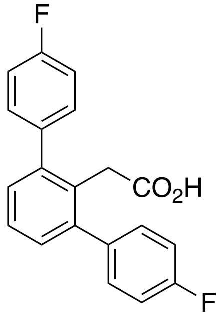

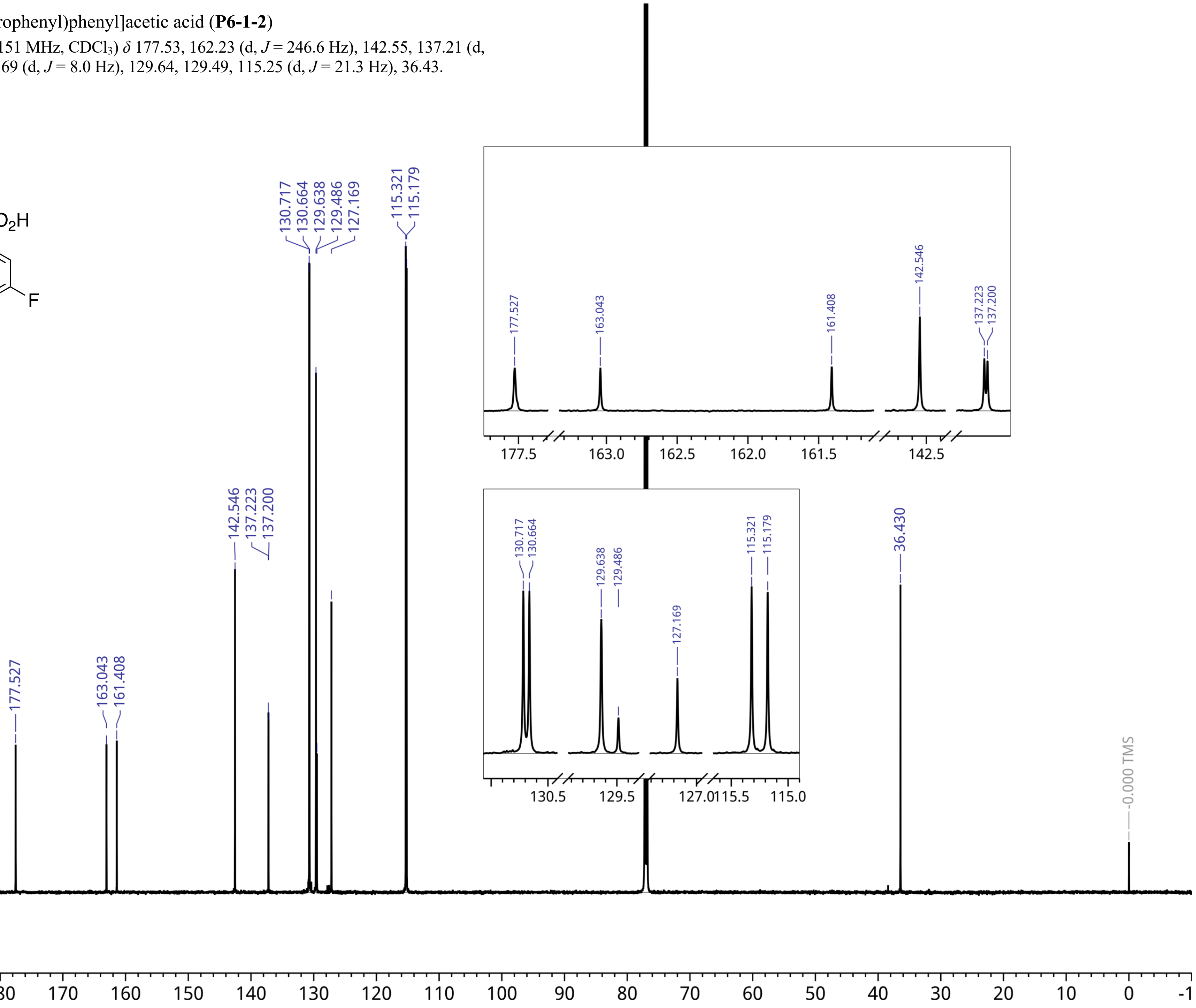

[2,6-di-(4-fluorophenyl)phenyl]acetic acid (**P6-1-2**)

^{19}F NMR (564 MHz, CDCl$_3$) δ –114.88 (tt, J = 8.7, 5.4 Hz).

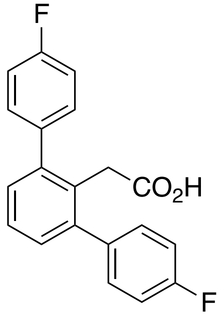

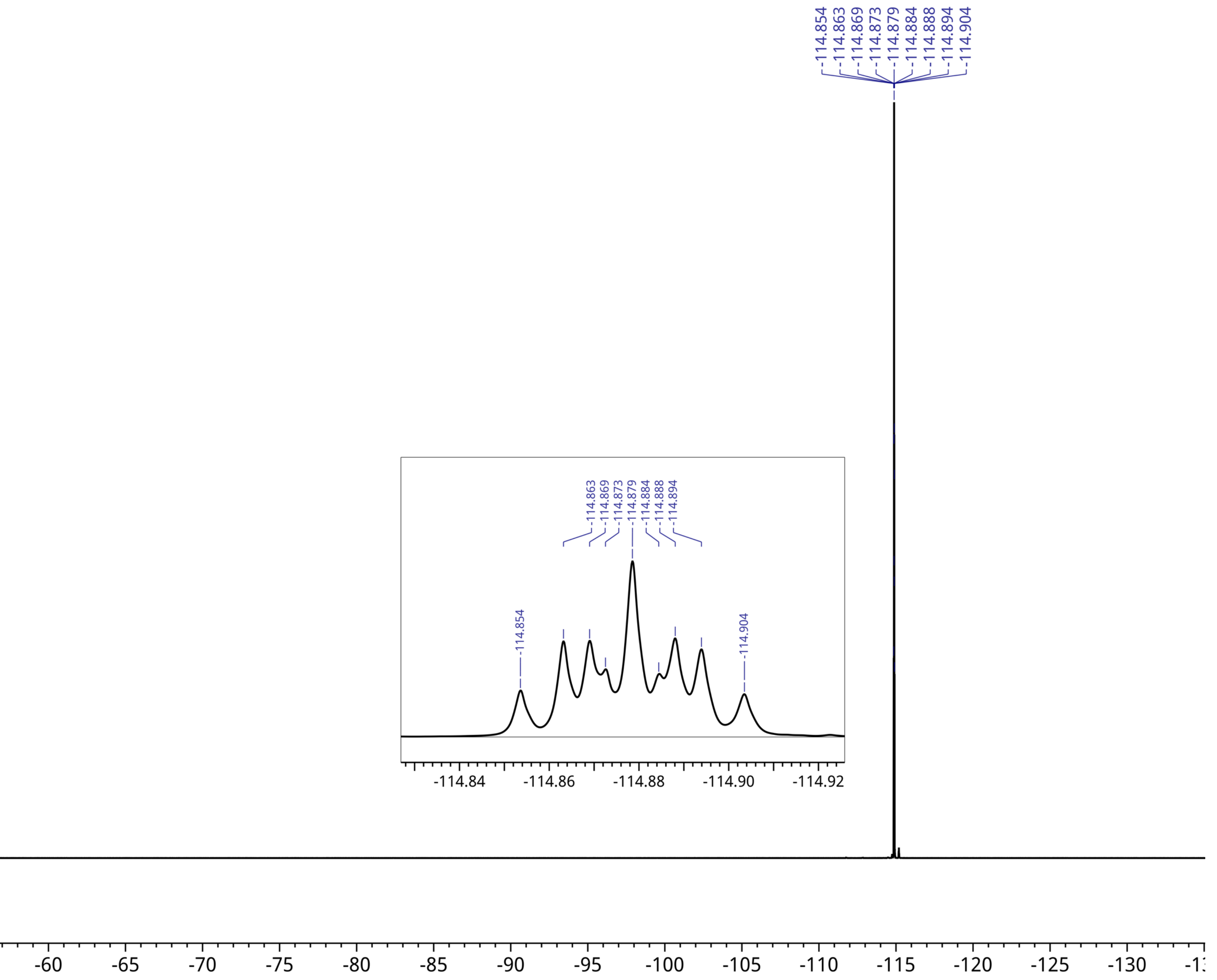

[2-(4-fluorophenyl)-6-methylphenyl]acetic acid (**P6-2**)

^{1}H NMR (600 MHz, $CDCl_3$) δ 11.28 (s, 1H), 7.28 – 7.20 (m, 4H), 7.13 – 7.05 (m, 3H), 3.62 (s, 2H), 2.35 (s, 3H).

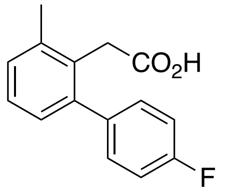

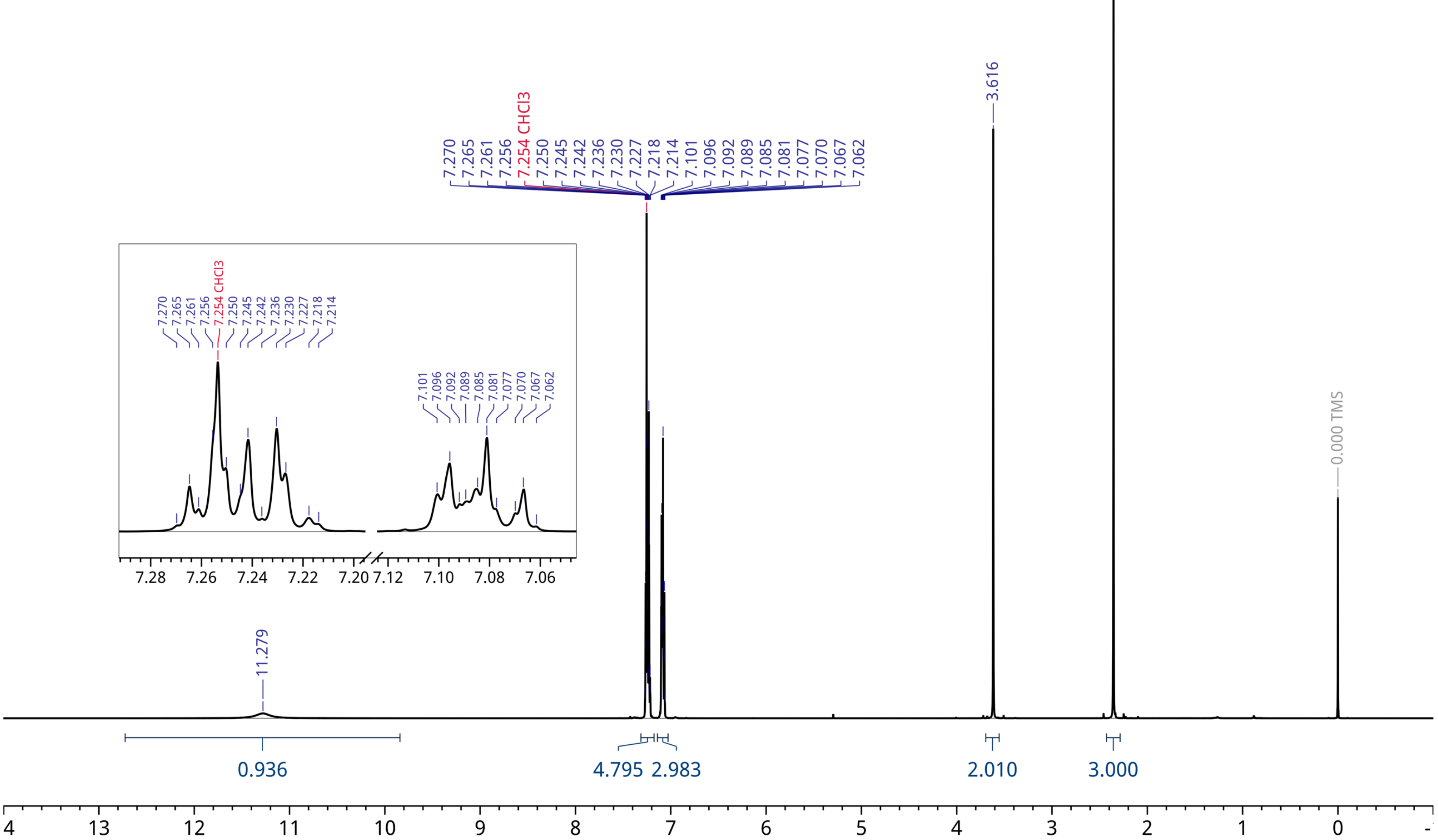

[2-(4-fluorophenyl)-6-methylphenyl]acetic acid (**P6-2**)

$^{13}C\{^{1}H\}$ NMR (151 MHz, $CDCl_3$) δ 177.72, 162.15 (d, J = 246.2 Hz), 142.30, 137.74, 137.58 (d, J = 3.4 Hz), 130.72 (d, J = 7.9 Hz), 130.07, 129.71, 128.03, 127.24, 115.15 (d, J = 21.4 Hz), 36.01, 20.21.

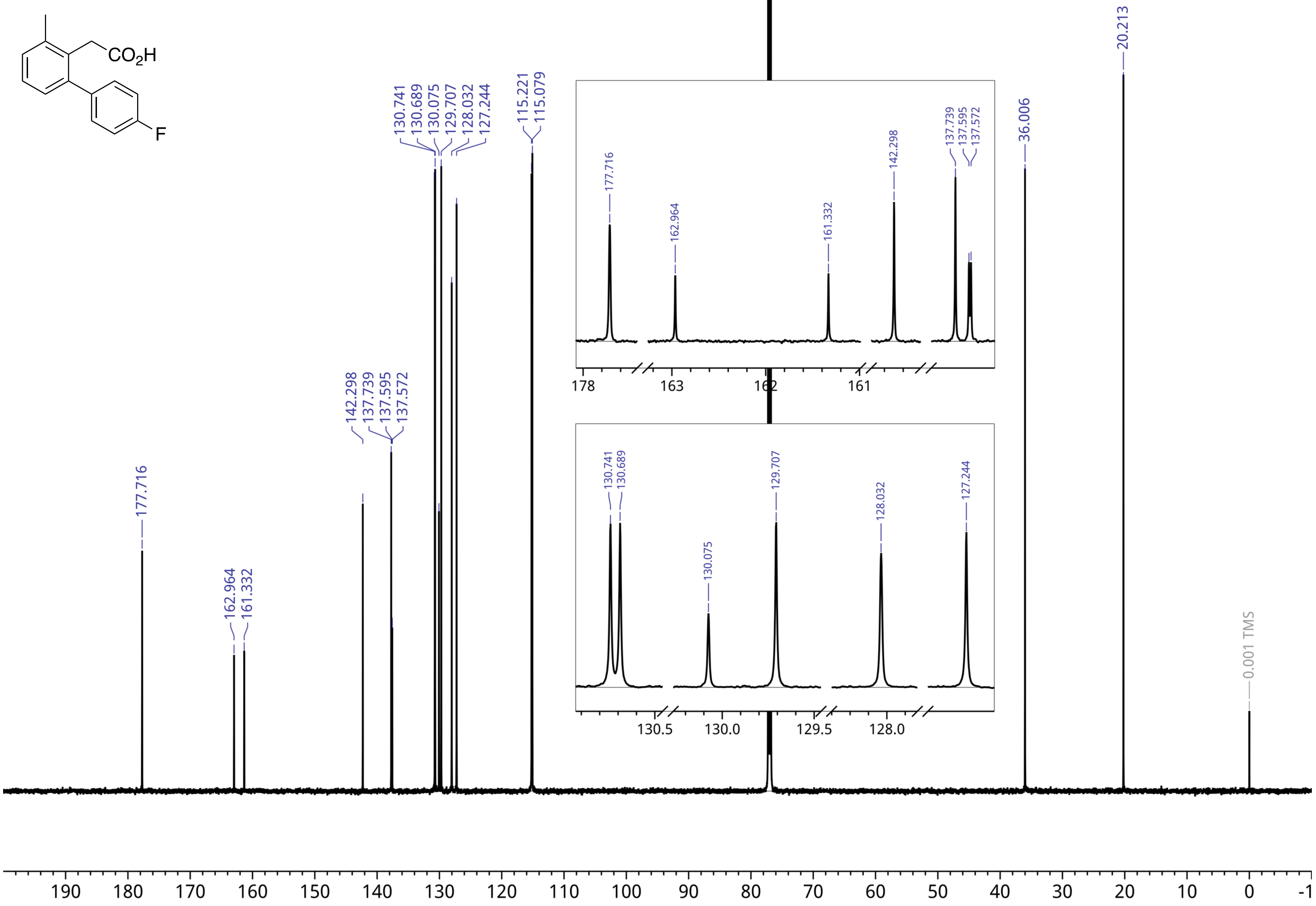

[2-(4-fluorophenyl)-6-methylphenyl]acetic acid (**P6-2**)

^{19}F NMR (564 MHz, $CDCl_3$) δ –115.41 (tt, J = 8.7, 5.4 Hz).

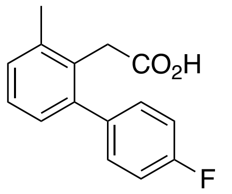

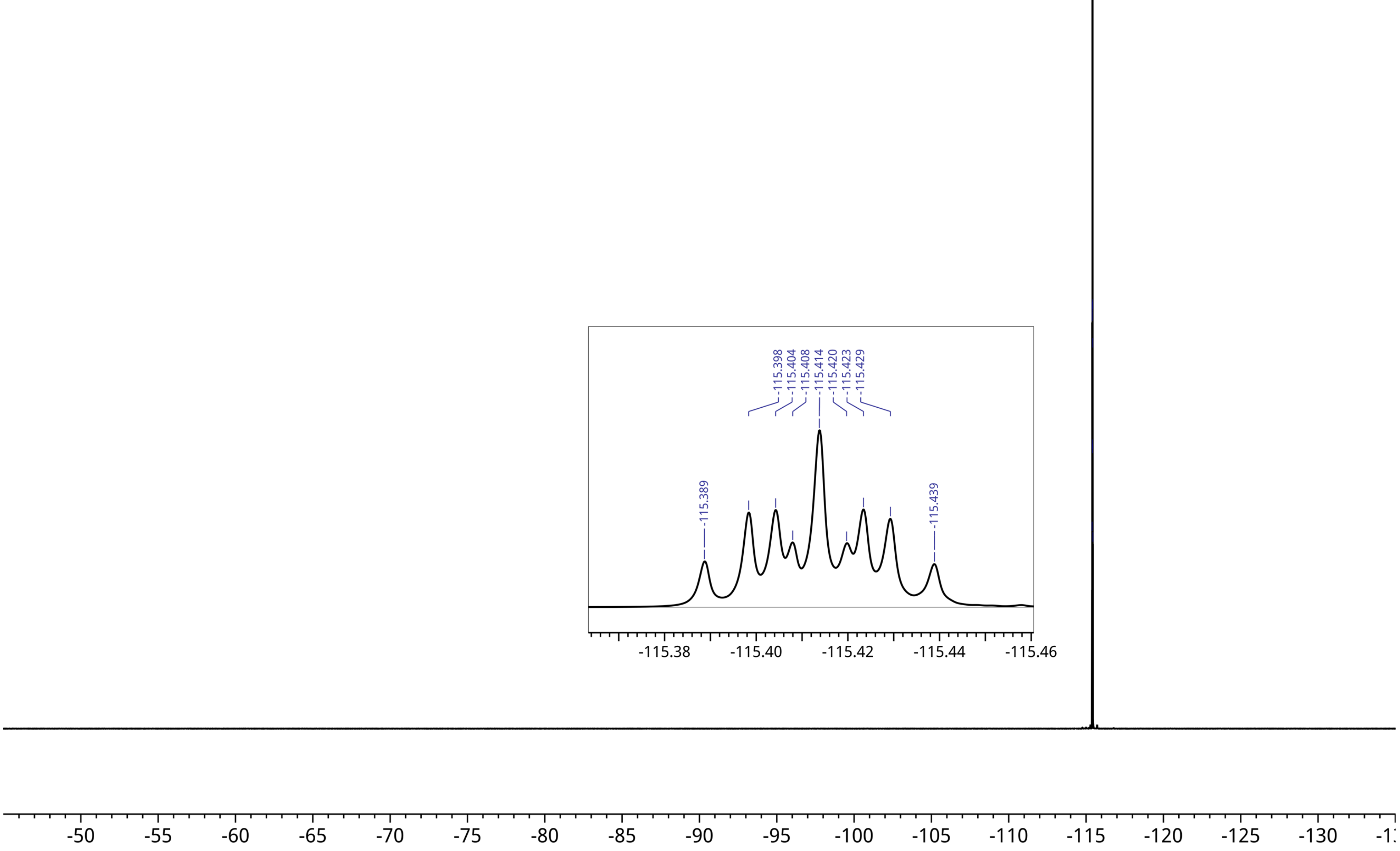

[2-(4-fluorophenyl)-6-(trifluoromethyl)phenyl]acetic acid (**P6-3**)

^{1}H NMR (600 MHz, $CDCl_3$) δ 11.12 (s, 1H), 7.72 (dd, J = 7.5, 1.8 Hz, 1H), 7.45 (t, J = 7.6 Hz, 1H), 7.42 (dd, J = 7.8, 1.8 Hz, 1H), 7.25 – 7.21 (m, 2H), 7.13 – 7.08 (m, 2H), 3.77 (s, 2H).

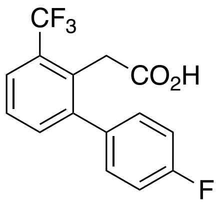

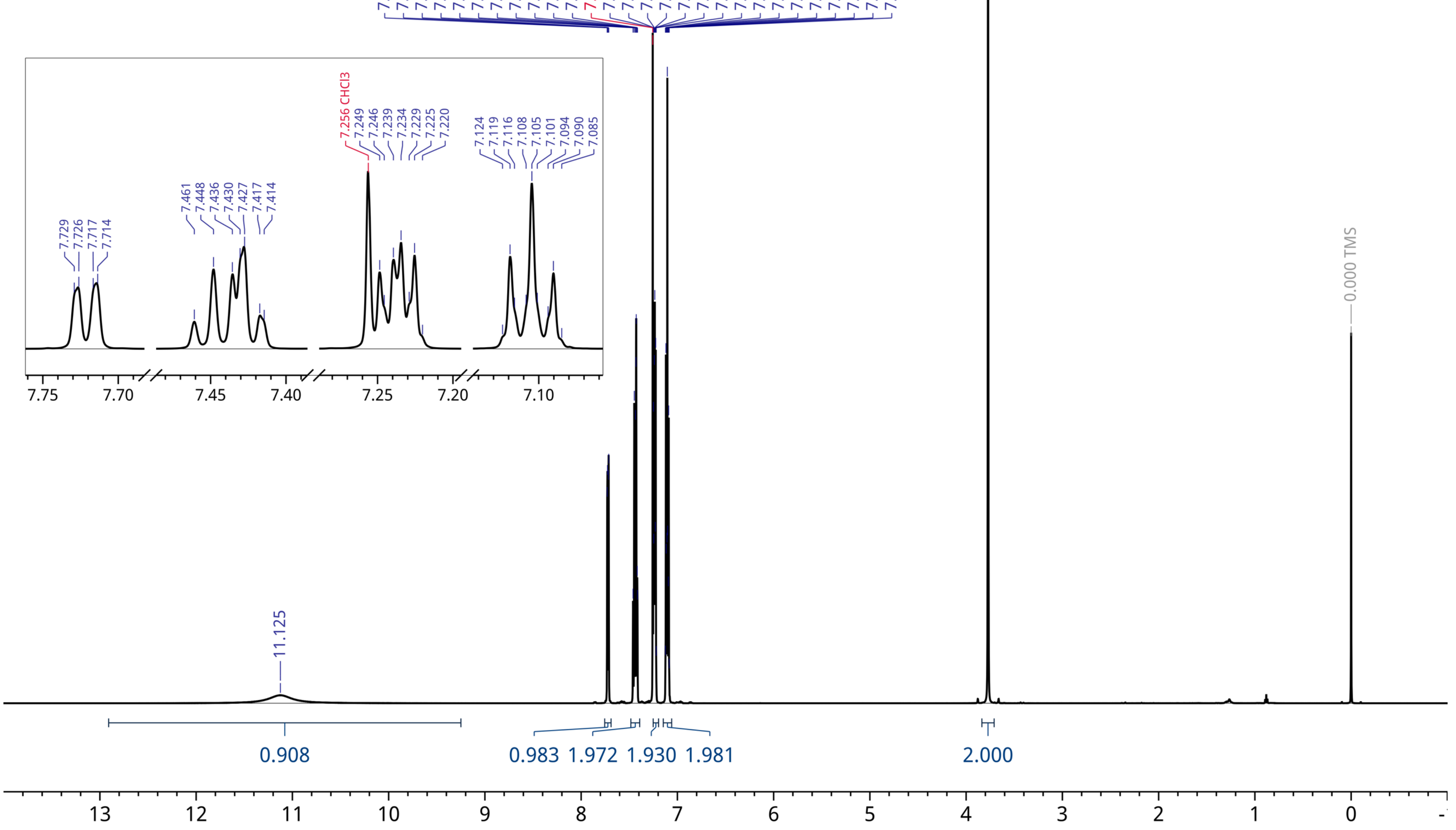

[2-(4-fluorophenyl)-6-(trifluoromethyl)phenyl]acetic acid (**P6-3**)

$^{13}C\{^{1}H\}$ NMR (151 MHz, $CDCl_3$) δ 176.80, 162.51 (d, J = 247.7 Hz), 144.42, 135.85 (d, J = 3.3 Hz), 133.93, 130.65 (d, J = 8.1 Hz), 129.79, 129.69 (q, J = 29.7 Hz), 127.46, 125.69 (q, J = 5.7 Hz), 124.33 (q, J = 274.0 Hz), 115.50 (d, J = 21.5 Hz), 35.21 (q, J = 2.0 Hz).

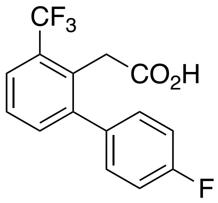

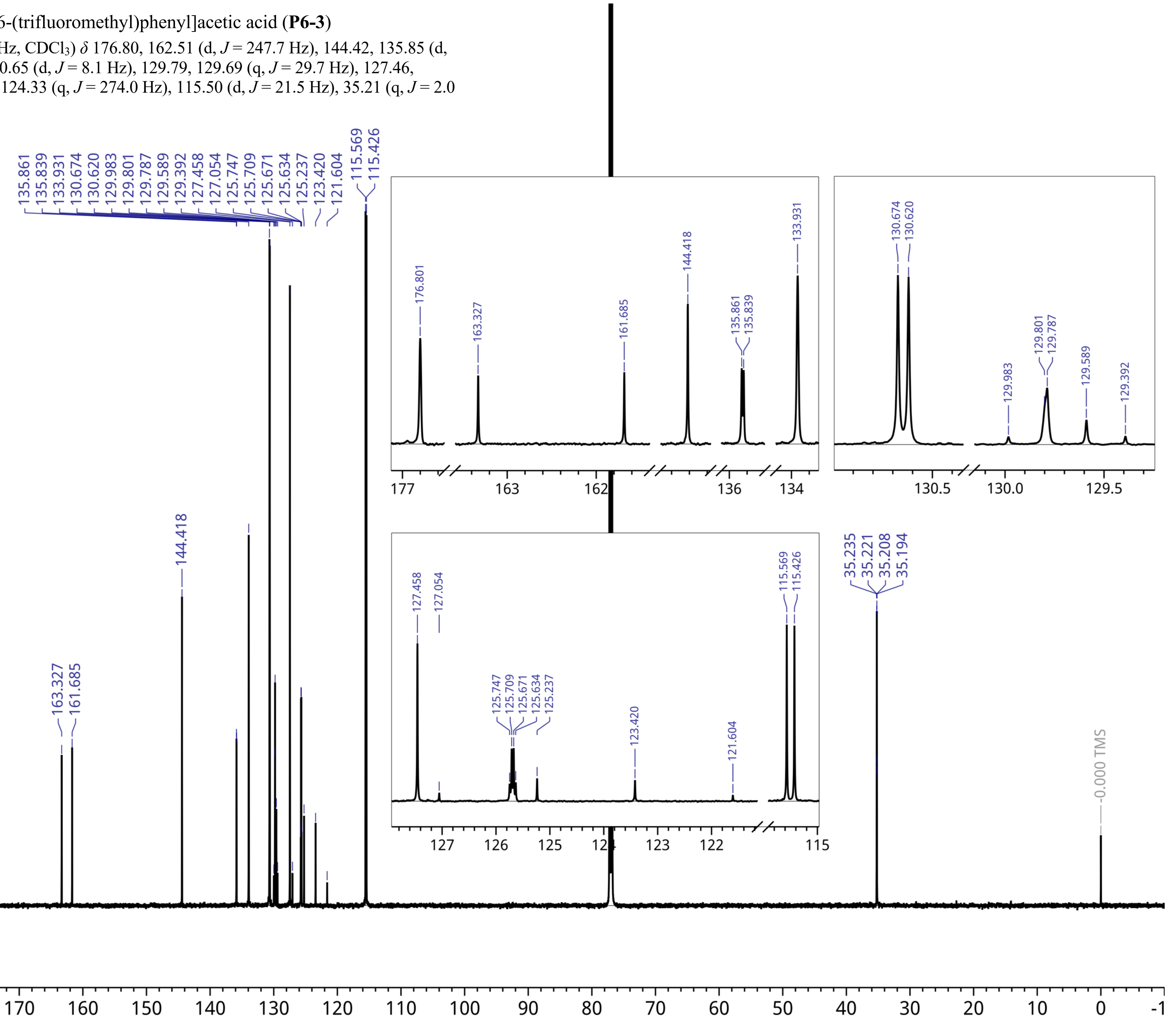

[2-(4-fluorophenyl)-6-(trifluoromethyl)phenyl]acetic acid (**P6-3**)

^{19}F NMR (564 MHz, $CDCl_3$) δ –59.99, –114.02 (tt, J = 8.6, 5.3 Hz).

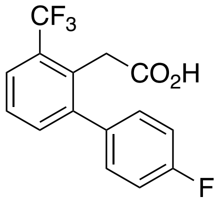

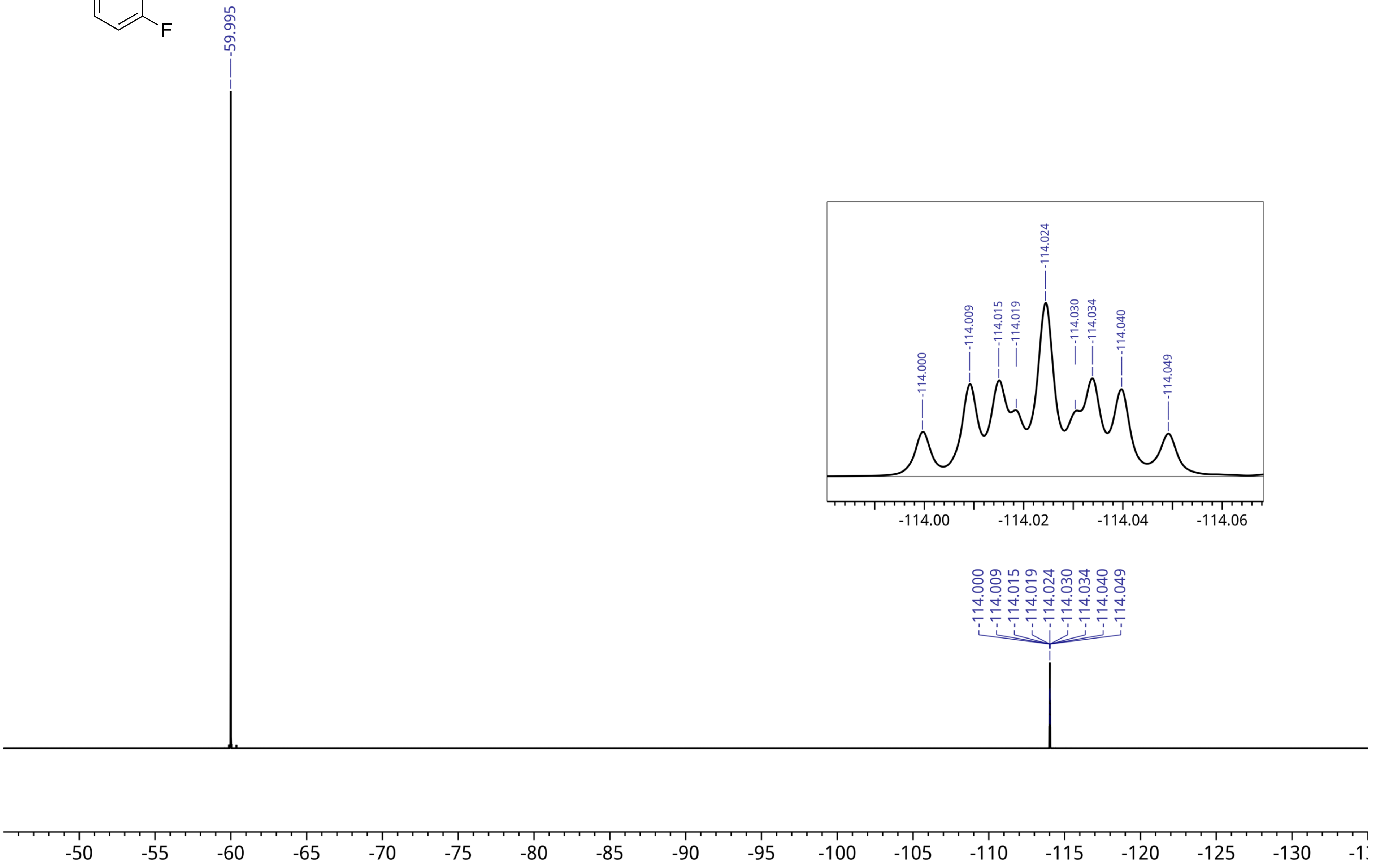

[2,6-di-(4-fluorophenyl)-4-methylphenyl]acetic acid (**P6-4-2**)

^{1}H NMR (600 MHz, $CDCl_3$) δ 10.57 (s, 1H), 7.26 (dd, J = 8.6, 5.3 Hz, 4H), 7.12 – 7.04 (m, 6H), 3.46 (s, 2H), 2.39 (s, 3H).

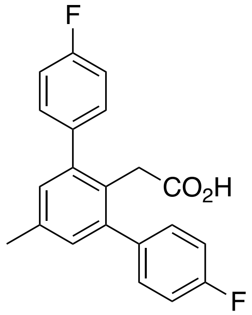

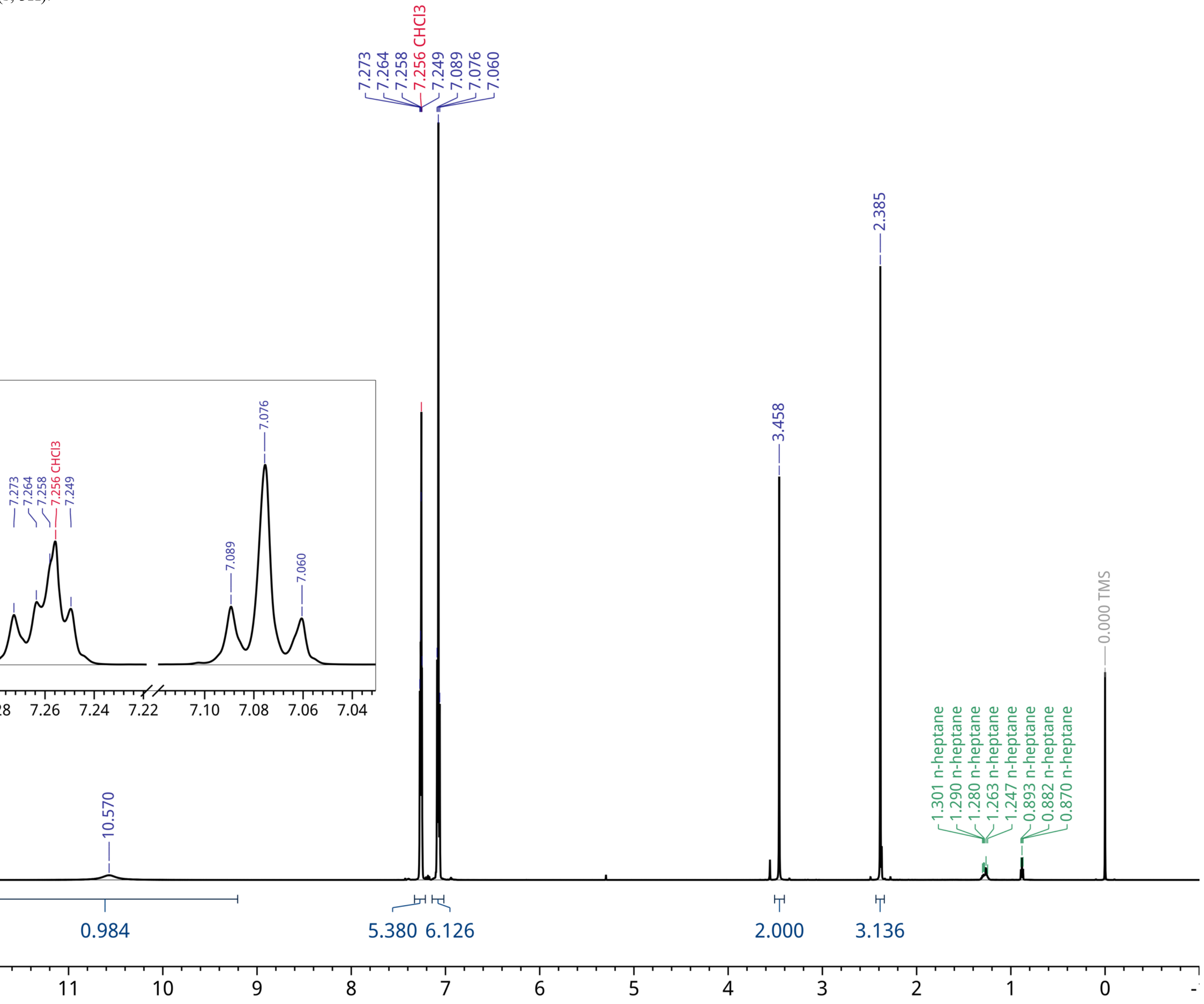

[2,6-di-(4-fluorophenyl)-4-methylphenyl]acetic acid (**P6-4-2**)

$^{13}C\{^{1}H\}$ NMR (151 MHz, $CDCl_3$) δ 177.62, 162.17 (d, J = 246.4 Hz), 142.40, 137.35 (d, J = 3.3 Hz), 136.87, 130.65 (d, J = 8.0 Hz), 130.43, 126.46, 115.19 (d, J = 21.3 Hz), 36.02, 20.98.

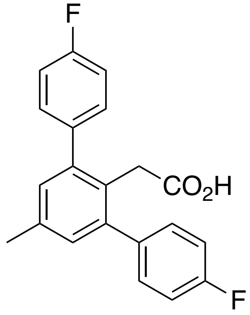

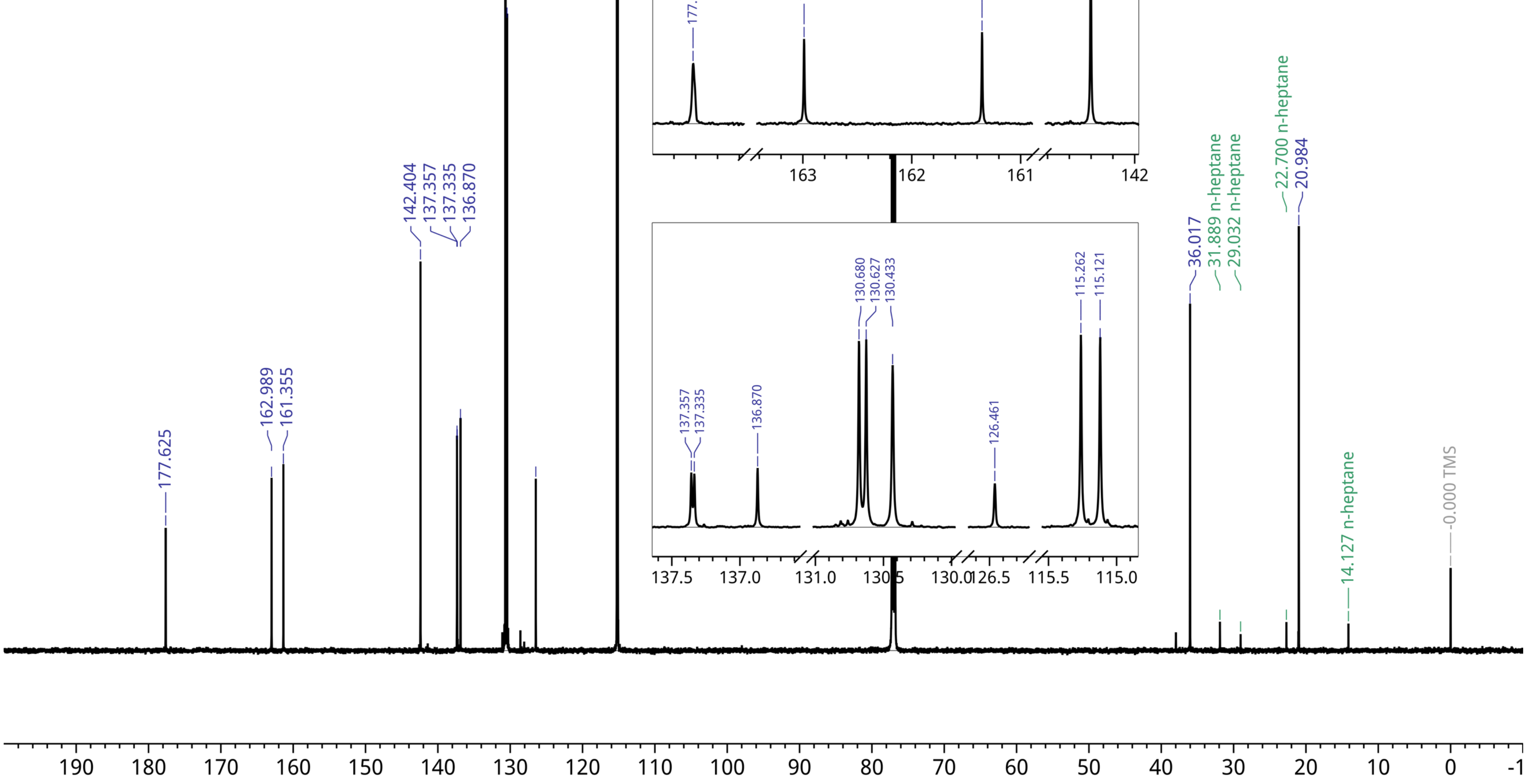

[2,6-di-(4-fluorophenyl)-4-methylphenyl]acetic acid (**P6-4-2**)

^{19}F NMR (564 MHz, $CDCl_3$) δ –115.05 (tt, J = 8.6, 5.4 Hz).

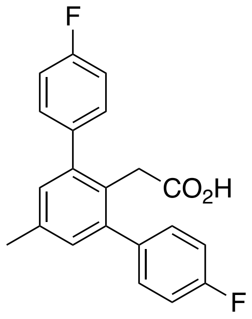

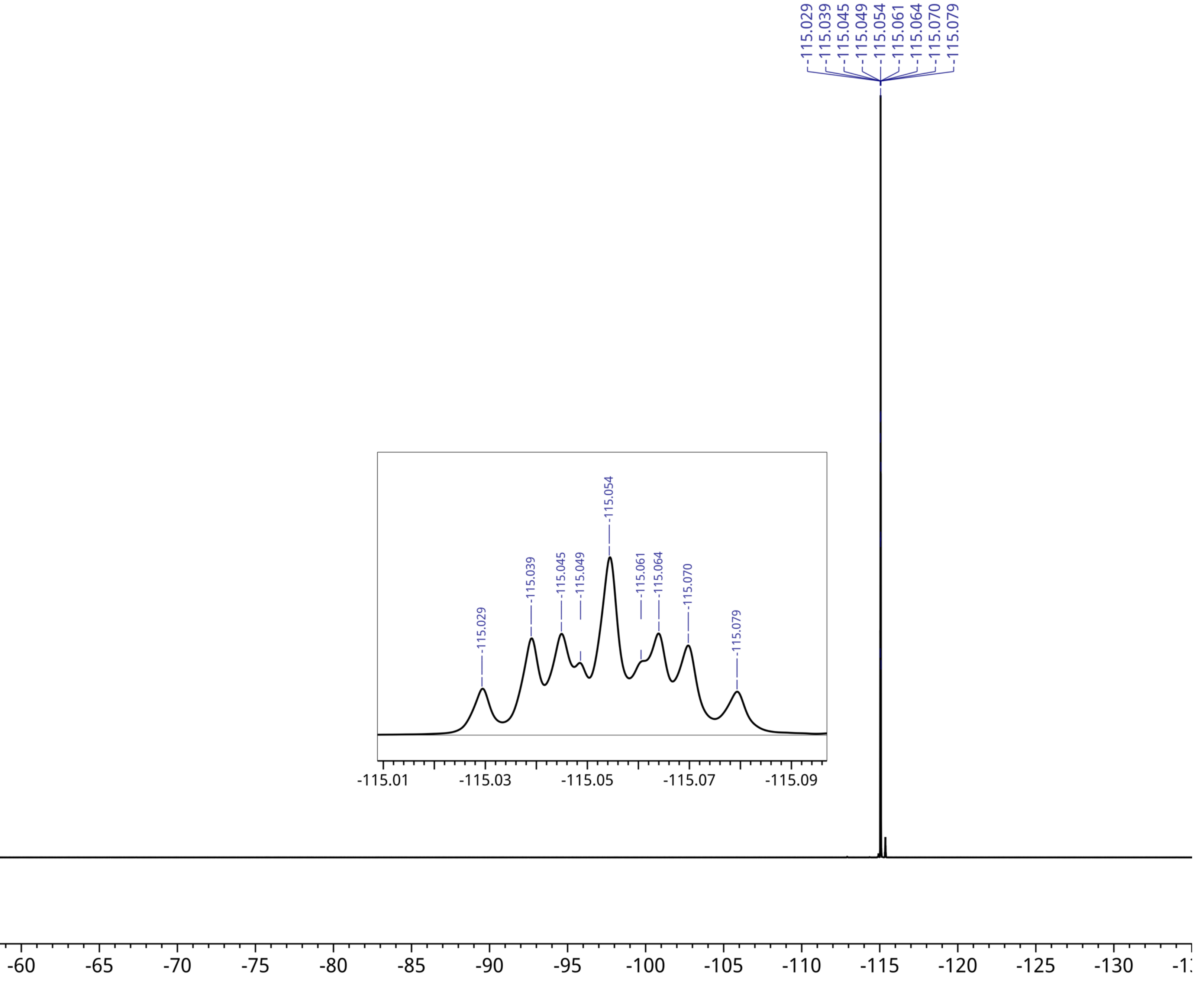

[2,6-di-(4-fluorophenyl)-4-(trifluoromethyl)phenyl]acetic acid (**P6-5-2**)

^{1}H NMR (600 MHz, $CDCl_3$) δ 10.58 (s, 1H), 7.51 (s, 2H), 7.31 – 7.26 (m, 4H), 7.16 – 7.09 (m, 4H), 3.55 (s, 2H).

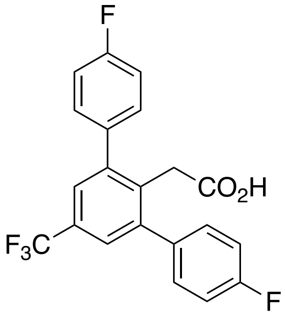

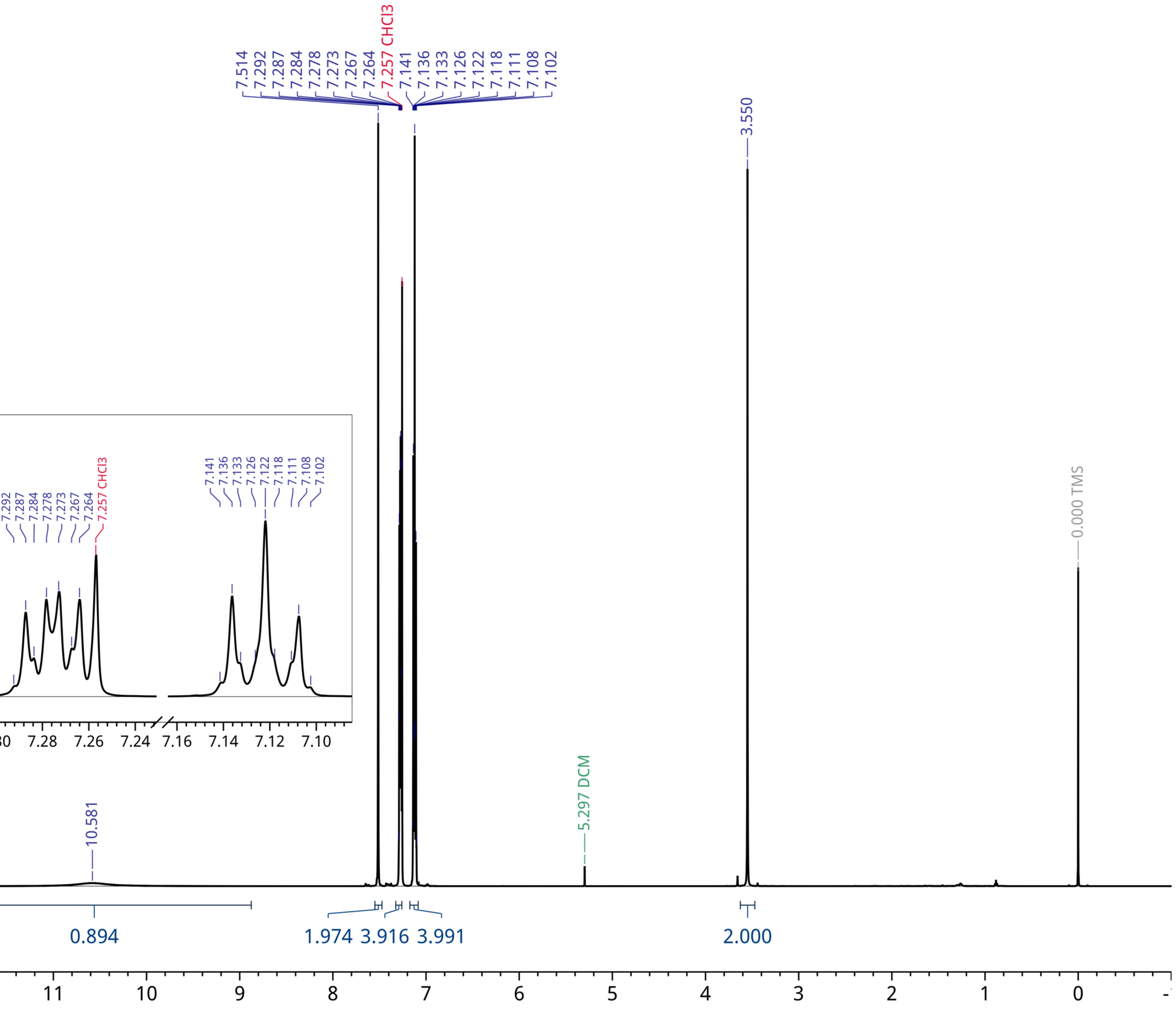

[2,6-di-(4-fluorophenyl)-4-(trifluoromethyl)phenyl]acetic acid (**P6-5-2**)

$^{13}C\{^{1}H\}$ NMR (151 MHz, $CDCl_3$) δ 176.72, 162.55 (d, J = 247.9 Hz), 143.35, 135.85 (d, J = 3.5 Hz), 133.55, 130.64 (d, J = 8.1 Hz), 129.50 (q, J = 32.7 Hz), 126.28 (q, J = 3.7 Hz), 123.79 (q, J = 272.6 Hz), 115.61 (d, J = 21.6 Hz), 36.40.

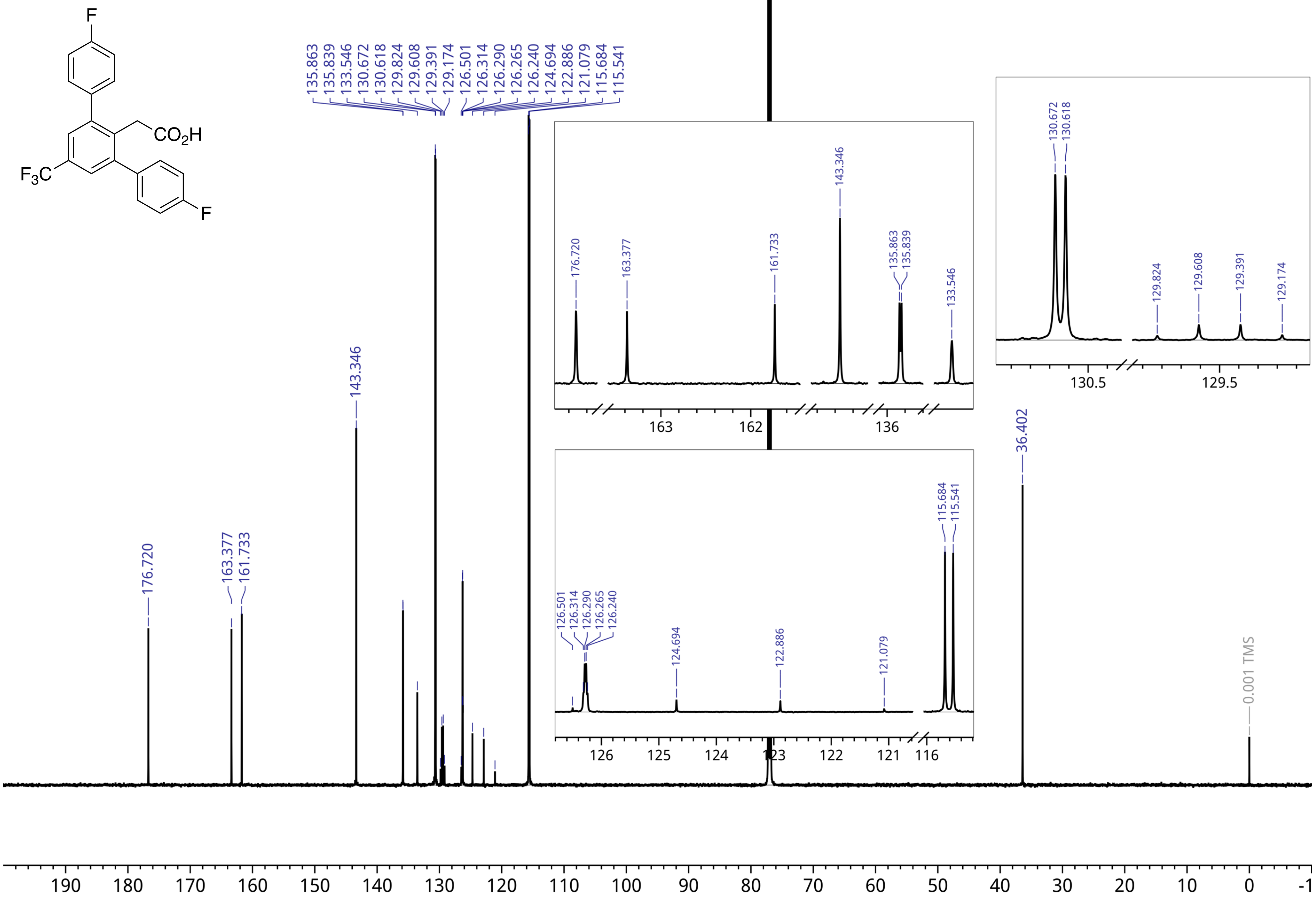

[2,6-di-(4-fluorophenyl)-4-(trifluoromethyl)phenyl]acetic acid (**P6-5-2**)

^{19}F NMR (564 MHz, $CDCl_3$) δ –62.56, –113.61 (tt, J = 8.6, 5.3 Hz).

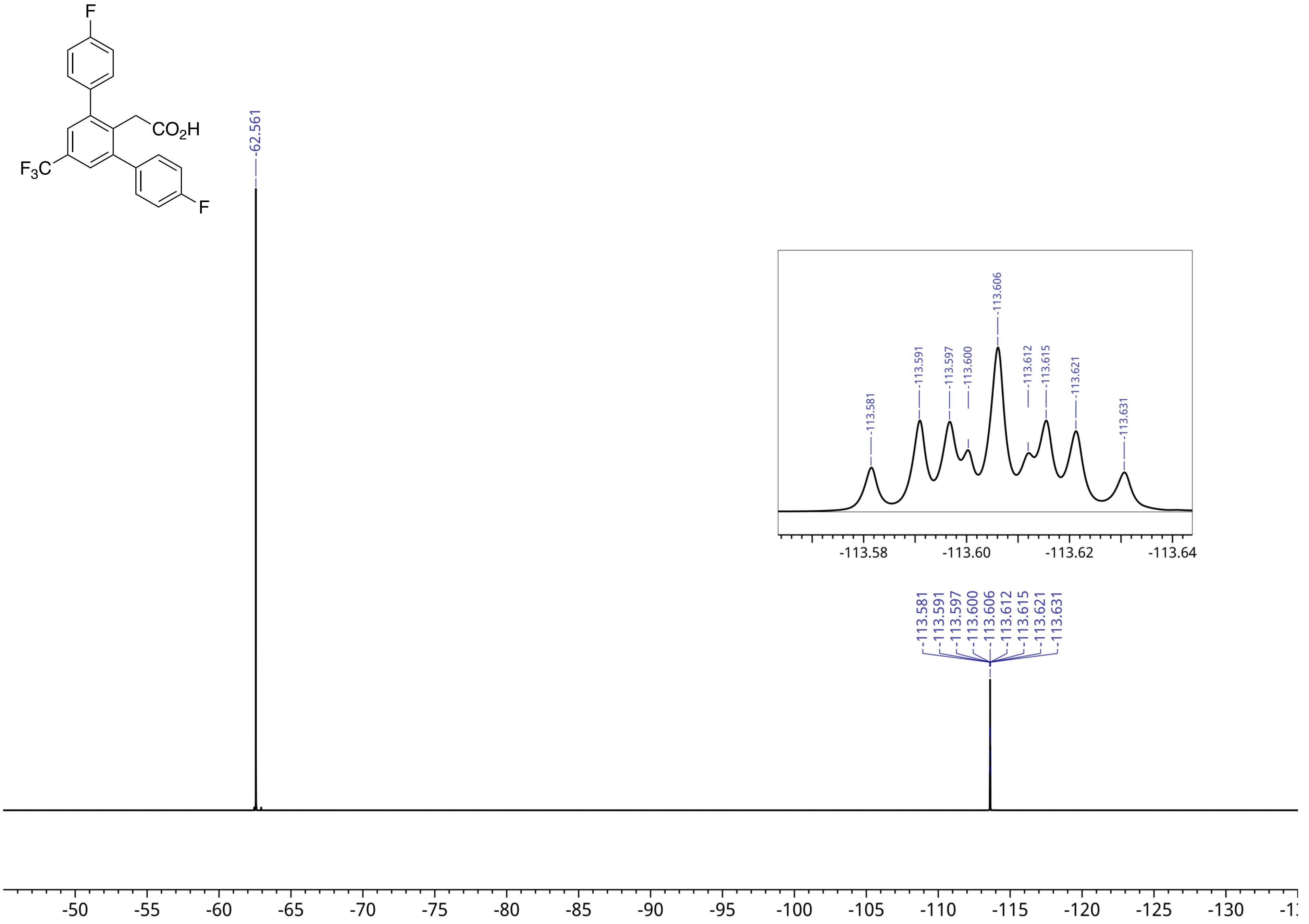

Supplement: Supplementary file 1 [file ja5c02735_si_001.pdf]
